# ﻿A phylogenetic and morphological study of the genus *Dermoloma* (*Agaricales*, *Tricholomataceae*) in Europe and North America exposes inefficiency of opportunistic species descriptions

**DOI:** 10.3897/imafungus.16.157337

**Published:** 2025-07-10

**Authors:** Katarína Adamčíková, Munazza Kiran, Miroslav Caboň, Brandon P. Matheny, Marisol Sánchez-García, Eef Arnolds, Michaela Caboňová, Gilles Corriol, Bálint Dima, Gernot Friebes, Gareth W. Griffith, Django Grootmyers, David Harries, Alexander Karich, Armin Mešić, Martin Mihaljevič, Pierre-Arthur Moreau, Ana Pošta, Vasilii Shapkin, Zdenko Tkalčec, Alfredo Vizzini, Lenka Vondrovicová, Slavomir Adamčík, Soňa Jančovičová

**Affiliations:** 1 Department of Plant Pathology and Mycology, Institute of Forest Ecology, Slovak Academy of Sciences Zvolen, Akademická 2, 949 01 Nitra, Slovakia Slovak Academy of Sciences Zvolen Nitra Slovakia; 2 Laboratory of Molecular Ecology and Mycology, Institute of Botany, Plant Science and Biodiversity Center, Slovak Academy of Sciences, Dúbravská cesta 9, 845 23 Bratislava, Slovakia Slovak Academy of Sciences Bratislava Slovakia; 3 Department of Botany, Division of Science & Technology, University of Education, Lahore, Pakistan University of Education Lahore Pakistan; 4 Department of Ecology and Evolutionary Biology, University of Tennessee, Knoxville, Tennessee, USA University of Tennessee Knoxville United States of America; 5 Department of Forest Mycology and Plant Pathology, Swedish University of Agricultural Sciences, Box 7026, 750 07 Uppsala, Sweden Swedish University of Agricultural Sciences Uppsala Sweden; 6 Department of Forest Ecology, Wageningen University, P.O. Box 9101, 6700 HB Wageningen, Netherlands Wageningen University Wageningen Netherlands; 7 Conservatoire botanique national des Pyrénées et de Midi-Pyrénées, Vallon de Salut. B.P. 315, 65203, Bagnères-de-Bigorre Cedex, France Conservatoire botanique national des Pyrénées et de Midi-Pyrénées Bagnères-de-Bigorre Cedex France; 8 Department of Plant Anatomy, Institute of Biology, Eötvös Loránd University, Pázmány Péter sétány 1/C, H-1117 Budapest, Hungary Eötvös Loránd University Budapest Hungary; 9 Studienzentrum Naturkunde, Universalmuseum Joanneum, Weinzöttlstraße 16, 8045 Graz, Austria Universalmuseum Joanneum Graz Austria; 10 Department of Life Sciences, Pont Cledwyn, Aberystwyth University, Aberystwyth SY23 3DD, Wales, UK Aberystwyth University Aberystwyth United Kingdom; 11 Somerton Cottage, Hundleton, Pembroke, Wales, UK Somerton Cottage, Hundleton Pembroke United Kingdom; 12 Technische Universität Dresden - Internationales Hochschulinstitut Zittau, Markt 23, 02763 Zittau, Germany Technische Universität Dresden Zittau Germany; 13 Laboratory for Biological Diversity, Ruđer Bošković Institute, Bijenička cesta 54, HR-10000 Zagreb, Croatia Ruđer Bošković Institute Zagreb Croatia; 14 Institute of Geochemistry, Mineralogy and Mineral Resources, Faculty of Science, Charles University, Prague 2, Albertov 6, Czech Republic Charles University Prague Czech Republic; 15 Laboratoire de Génie Civil et géo-Environnement, University of Lille, ULR 4515 – LGCgE, 3 rue du Pr Laguesse, F-59000 Lille, France University of Lille Lille France; 16 Department of Life Sciences and Systems Biology, University of Torino, Vale P.A. Mattioli 25, 10125 Torino, Italy University of Torino Torino Italy; 17 Department of Botany, Faculty of Natural Sciences, Comenius University Bratislava, Révová 39, 811 02 Bratislava, Slovakia Comenius University Bratislava Bratislava Slovakia

**Keywords:** Agarics, CHEGD fungi, chromatic analysis, stable isotypes, systematics, taxonomy

## Abstract

*Dermoloma* is traditionally known as a small genus of agarics classified in the family *Tricholomataceae*. This study implemented a multilocus phylogeny of six DNA regions to recognize phylogenetic species within the genus. The species concept is reinforced by observations of well-defined morphological characters enhanced by long term sampling effort in Europe and North America. Thirty European *Dermoloma* species are described, including 16 new species from Europe and three from North American. These species are classified into two subgenera morphologically distinguished by spores with positive or negative amyloid reaction. A new genus *Neodermoloma* is introduced for the *Dermoloma*-like species *N.campestre*. Localized or continental-scale species endemicity was confirmed based on studied material, but more inclusive phylogenetic clustering supported a mixture of North American species among the European clades. Of the 22 names validly published from Europe prior to this study, 11 could be assigned to well-defined *Dermoloma* species recognized here. Of the remaining 11 names, two were considered representing *Dermoloma* species not recorded since their description, and nine were established as later synonyms of other species. Morphological studies of *Dermoloma* are challenging due to the relatively low number of characters suitable for identification of species. The majority of morphological characters showed continuous variation with high overlap throughout the genus. For this reason, species identification requires an awareness of morphological variability within species, and multiple distinguishing characters need to be combined, and furthermore, often a barcode sequence is needed for a certain identification. Stable isotope analysis in *Dermoloma* of δ^13^C and δ^15^N revealed an ecological signature similar to known CHEGD fungi, i.e. *Clavariaceae* and *Hygrocybe* s.l. This indicates that *Dermoloma* species are biotrophic but neither ectomycorrhizal nor saprotrophic and may form mutualistic root endophytic associations with vascular plants.

## ﻿﻿Introduction

*Dermoloma* (J.E. Lange) Singer ex Herink is an insufficiently known genus of small-sized agarics classified in the family *Tricholomataceae* R. Heim ex Pouzar (*Tricholomatineae*, *Agaricales*, *Agaricomycotina*, *Basidiomycota*) (Sánchez-García and Matheny 2017; Vizzini et al. 2024). A morphological and phylogenetic circumscription of the genus was recently made by Sánchez-García et al. (2021). *Dermoloma* is characterized by prevailing gray-brown colors of the basidiomata, fragile context with a typical farinaceous odor, and a hymeniderm or epithelium type of pileipellis formed by inflated obpyriform or sphaeropedunculate terminal cells. The genus comprises two subgenera, D.subgen.Dermoloma with inamyloid spores and D.subgen.Amylospora Adamčík with amyloid spores. European members are typically known to inhabit semi-natural grassland ecosystems as part of CHEGD (acronym of *Clavariaceae*, *Hygrocybe* s.l., *Entoloma*, *Geoglossaceae*, *Dermoloma*) fungi, which have a biotrophic lifestyle and probably form an unspecified symbiosis with vascular plants (Halbwachs et al. 2013, 2018; Tello et al. 2014).

**Figure 1.**
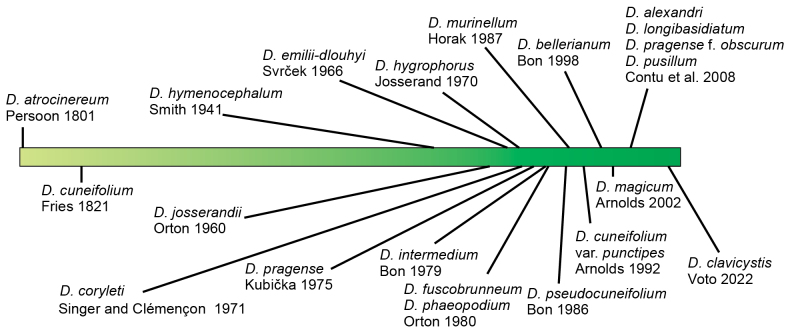
Chronological timeline of species description in the genus *Dermoloma* from Europe and North America.

The first two species historically placed in the genus, *D.atrocinereum* (Pers.) P.D. Orton and *D.cuneifolium* (Fr.) Singer ex Bon (Orton 1960; Bon 1986), were initially described in *Agaricus* L. during the early 19^th^ century (Persoon 1801; Fries 1821). Due to morphological similarities among *Dermoloma* members, there was a gap of 120 years between Friesian names and acceptance of subsequent species such as *D.hymenocephalum* (A.H. Sm.) Singer. The majority of European taxa were described during the past sixty years (Fig. 1). Forty species and lower rank taxa have been described thus far in *Dermoloma* globally, among them 26 from Europe (Sánchez-García et al. 2021; Voto 2022). However, only three (Arnolds 1992, 1993) to 11 taxa (Contu et al. 2008) were accepted in the modern European literature. In contrast, a recent genus-level phylogeny (Sánchez-García et al. 2021) supported 25 European species-rank clades and six North American species-rank clades. From these, only 11 clades were identified by sequences from type or authenticated material, leaving many species clades unpaired with available names. The current situation with unstable taxonomy and naming, is a consequence of the obscurity of morphological characters used for species delimitation, with some species, for example, distinguished only by subtle variation in basidiome color (Orton 1980; Contu et al. 2008).

Another character frequently used for species identification is spore dimension. Sánchez-García et al. (2021) demonstrated the importance of spore size for distinguishing some species but not for all. Well-elaborated studies dealing with other genera of similar agarics with inconspicuous field characters usually rely on additional morphological observations and differences of the pileipellis, hymenial cystidia and caulocystidia (Vašutová et al. 2008; Song and Bau 2023). *Dermoloma* lacks distinct hymenial cystidia, and most species descriptions provide little information about microscopic elements other than spores and basidia. The use of elements on lamellar edges and caulocystidia for species delimitation is rare and not supported by observations on other *Dermoloma* species (Contu et al. 2008; Voto 2022).

Our study expands the taxon sampling for phylogenetic analyses and revises morphological criteria by which species can be recognized. The previous study by Sánchez-García et al. (2021) included 15 potential species represented only by a single sample. Nevertheless, considerable higher diversity of the genus was observed in Europe compared to North America, with no species detected from both continents. Our aim is to achieve phylogenetic and morphological congruence by gathering more reliable *Dermoloma* samplings provided by an extended dataset of six DNA regions and statistically supported morphological differences based on measurements from multiple collections per species. Taxonomic and nomenclatural stability established by providing genetic barcodes and morphological differences between species are essential to globally understand the ecological significance of *Dermoloma* diversity in terrestrial ecosystems.

## ﻿﻿Material and methods

### ﻿﻿Sampling

The study was based on material used by Sánchez-García et al. (2021), supplemented by additional, mainly more recent, *Dermoloma* collections. All collections were putatively identified as belonging to this genus by field appearance and their identification was later confirmed by sequencing of the ITS region. In total, the studied material includes more than 350 samples, including type and authenticated material of several *Dermoloma* species. The authors provided 208 new collections, mainly from *Dermoloma*. Approximately half of the fungal collections (165 specimens) were collected in 2005–2022 and deposited at the herbarium of Slovak Academy of Sciences (SAV). A part of the studied material was deposited in the following herbaria ABS, AMB, AQUI, BBF, C, CNF, E, ELTE, G, GDOR, GLM, H, L, LIP, MICH, O, PRM, SLO, TENN, TUR and UPS (abbreviations follows Index Herbariorum, Thiers, updated continuously).

### ﻿﻿DNA extractions and sequencing

A small piece of dried material (10–30 mg) was sampled and grinded in a 1.5-ml tube with sterilised sand or liquid nitrogen and a micropestle. Genomic DNA was extracted using the E.Z.N.A. Fungal DNA Mini Kit (Omega Bio-Tek, Inc., Norcross, GA, USA) and the E.Z.N.A. HP Fungal DNA Kit (Omega Bio-Tek) following the manufacturer’s instructions. DNA amplification was performed using a PCR mix consisting of approximately 2 ng/μl of template DNA, molecular grade water, forward and reverse primers (10 pmol/μl), 5× HOT FIREPol® Blend Master Mix (Solis BioDyne, Tartu, Estonia) or using a mixture of 5× buffer, GoTaq and dNTPs supplied by Invitrogen Corp. (Carlsbad, CA, USA) or GoTaq® G2 Green Master Mix (Promega, Madison, WI, USA). The following loci were targeted: (i) the internal transcribed spacer regions of nuclear ribosomal DNA (ITS), (ii) the nuclear ribosomal large subunit (LSU), (iii) the first largest subunit of RNA polymerase II (*rpb1*), (iv) the most variable region between domains 6 and 7 of the nuclear gene encoding the second largest subunit of RNA polymerase II (*rpb2*), (v) the DNA replication licencing factor, *mcm7* component (*mcm7*) and (vi) a portion of transcription elongation factor 1-alpha (*tef1a*). The ITS was amplified with the primers ITS1F-ITS4 (White et al. 1990; Gardes and Bruns 1993). In cases where the ITS region was not amplified successfully using these two primers, partial regions ITS1 and ITS2 were sequenced using the primers ITS1-ITS2 and ITS3-ITS4 respectively (White et al. 1990). The LSU was amplified using the primer pairs LR0R/LR16, LR0R/LR5 or LR0R/LR7 (Vilgalys and Hester 1990, https://sites.duke.edu/vilgalyslab/rdna_primers_for_fungi/). The primers bRPB2-6F and bRPB2-7.1R (Matheny 2005) and the primers gRPB1-A for and fRPB1-C rev (Matheny et al. 2002) were used to amplify the *rpb2* and *rpb1* regions, respectively. The *mcm7* gene was amplified using *mcm7*-709for and *mcm7*-1348rev primers (Schmitt et al. 2009). For amplification of *tef1a* primers 983F and 2218R or additional internal primers 1567R and 1577F (Rehner and Buckley 2005) were used. Additionally, the forward primer Ef1-Derm2_F (CAGGATGTTTACAAAATTGGYGG), designed in the course of this study, was also used. The targeted amplified fragments were purified using a PCR Purification Kit (Qiagen, Hilden, Germany) or using ExoSAP-IT™ (Thermo Fisher Scientific, Waltham, MA, USA). Sequencing was performed at the University of Tennessee Genomics Core, at the SEQme sequencing company (Dobříš, Czech Republic) or Macrogen Europe (Amsterdam, The Netherlands).

For some specimens originating from Denmark, Hungary and Norway, the ITS region was amplified directly using the Phire® Plant Direct PCR Kit (Thermo Scientific, USA). A small piece of lamella was lysed in 20 μl Dilution Buffer. The samples were incubated at room temperature for 10 min and then 1 μl of the lysate was added as template for PCR amplification reactions. The primers ITS1F and ITS4 (White et al. 1990; Gardes and Bruns 1993) were used for amplifications, which were performed in 20 μl volume containing 6.6 μl nuclease freewater, 1 μl sample (from dilution protocol), 0.5 μM of each primer, 10 μl of 2× Phire Plant PCR Buffer and 0.4 μl of Phire Hot Start II DNA Polymerase. The PCR reactions were performed using the following program: 5 min at 98 °C for initial denaturation, 40 cycles of 5 s denaturation at 98 °C, 5 s annealing at 55 °C, and 20 s extension at 72 °C, followed by a final extension for 1 min at 72 °C. Amplicons were sequenced at LGC Genomics (Berlin, Germany) with the same primers as those applied in the PCR reactions. Sequences were assembled and edited with the CodonCodeAligner 4.1. (CodonCode Corporation, USA). Some of the Norwegian sequences were generated within the Norwegian Barcode of Life (NorBOL) project.

### ﻿﻿Sequence editing and phylogenetic analyses

Sequence files were edited in Geneious (version R10) (Kearse et al. 2012). Intra-individual polymorphic sites having more than one signal were marked with NC-IUPAC ambiguity codes. The majority of sequence data used here were generated by the authors and this allowed detailed inspection of the original sequence chromatograms. Sequence files of individual species or small groups of related species were aligned in Geneious and ambiguous low-quality positions were additionally checked within these alignments. All sequences used in the phylogenetic studies with accession numbers are listed in Suppl. material 1.

For the phylogeny of the genus *Dermoloma* a multilocus alignment of all six loci (ITS, LSU, *rpb1*, *rpb2*, *mcm7* and *tef1a*) was constructed adding *Pseudotricholoma* (Singer) Sánchez-García & Matheny sequences as an ingroup (some of them newly sequenced for the purpose of this study) and *Tricholomaterreum* (Schaeff.) P. Kumm. and *T.portentosum* (Fr.) Quél. as an outgroup (retrieved from Ding et al. 2023).

For the phylogenetic placement of the genus *Dermoloma* within the family *Tricholomataceae*, we used selected *Dermoloma* samples representing distinct clades within the genus, supplemented with sequences from the following genera published by Sánchez-García et al. (2014) and Sánchez-García and Matheny (2017): *Albomagister* Sánchez-García, Birkebak & Matheny; *Corneriella* Sánchez-García; *Dennisiomyces* Singer; *Leucopaxillus* Boursier; *Porpoloma* Singer; *Pseudobaeospora* Singer; *Pseudotricholoma*; and *Tricholoma* (Fr.) Staude. For the broader phylogenetic placement, multilocus alignments of five DNA regions were used (ITS, LSU, *rpb1*, *rpb2* and SSU) because *mcm7* and *tef1a* were not available from published sources Suppl. material 1. *Singerocybeadirondackensis* (Peck) Zhu L. Yang & J. Qin and *Entolomaasterosporum* (Coker & Couch) T.J. Baroni & Matheny were used as outgroups for the family-level phylogeny.

Alignments of individual loci in both multi-loci datasets were aligned with the MAFFT 7 online service (Katoh et al. 2019) using the E-INS-i settings (Katoh and Standley 2013) and then manually curated in Geneious R10 (Kearse et al. 2012) or Aliview 1.28 (Larsson 2014). Divergent and ambiguously aligned positions of ITS and LSU were removed with Gblocks (Castresana 2000) using the least stringent parameters. Due to the high level of heterogeneity, intronic regions of single copy genes (*rpb1*, *rpb2*, *mcm7* and *tef1a*) were manually removed. The resulting alignments of individual loci were concatenated into a single multi-loci dataset to be further analyzed with two different methods: Maximum Likelihood (ML) and Bayesian Inference (BI). The final alignment files (Suppl. material 2: *Dermoloma* multilocus alignment, Suppl. material 3: *Tricholomataceae* multilocus alignment, Suppl. material 4: ITS alignment) were deposited in Figshare (https://figshare.com/s/903834519a46fcd6190b).

For the *Tricholomataceae* dataset, the ML analysis was done with RAxML 8.2.10 (Stamatakis 2014) and the BI was performed with MrBayes 3.2.6 (Ronquist et al. 2012) using the high-performance computer cluster UPPMAX (Uppsala University). The phylogenetic analyses for the *Dermoloma* dataset were undertaken in the CIPRES Science Gateway (Miller et al. 2010; https://www.phylo.org/), using RAxML-HPC2 on XSEDE (8.2.12) (Stamatakis 2014) and MrBayes 3.2.7a (Ronquist et al. 2012). The concatenated alignments were uploaded as FASTA files and analyzed as a six-partitioned dataset under the GTR + GAMMA model with 1000 bootstrap iterations. For the BI analysis, the dataset was divided into six partitions: ITS, LSU, *rpb1*, *rpb2*, *mcm7* and *tef1a*. The best substitution model for each partition was computed jointly in PartitionFinder 1.1.1 (Lanfear et al. 2012). The aligned FASTA datasets were converted to nexus format using Mesquite 3.61 (Maddison and Maddison 2019) or Aliview and further analysed using MrBayes 3.2.7a. Bayesian runs were computed twice independently with four MCMC chains for 10 million generations until the standard deviation of split frequencies fell below the 0.01 threshold. The convergence of runs was visually assessed using the trace function in Tracer 1.6 (Rambaut et al. 2014). The trees were visualized and annotated with TreeGraph 2 (Stöver and Müller 2010) and graphically improved in CorelDRAW X5 (Ottawa, Canada).

All ITS sequences pooled into an extended dataset were analysed as a single partition with Maximum Likelihood using RAxML-HPC2 on XSEDE (8.2.12) (Stamatakis 2014) under the GTR + GAMMA model with 1000 bootstrap iterations. The trees were visualized and annotated with TreeGraph 2 (Stöver and Müller 2010) and graphically improved in CorelDRAW X5 (Ottawa, Canada). Clades with ML bootstrap support values greater or equal to 90% and Bayesian posterior probabilities greater or equal to 0.98 are considered as strongly supported.

### ﻿﻿Morphology

From the recent material, only collections with confirmed affinity to the genus *Dermoloma* by ITS sequence analysis (in a few cases also LSU) were included. Field descriptions were prepared from fresh material shortly after the sampling and most observations were registered using a description form (Suppl. material 5). The number of full-length lamellae is labelled in the species descriptions as “L” and the number of short lamellulae between each pair of full-length lamellae is labelled as “l” (Vellinga 1988). Color nomenclature standards followed Kornerup and Wanscher (1978). Color variation of basidiomata was visualized on charts prepared in CorelDRAW X5 software (Ottawa, Canada). Microscopic structures were examined on fungarium specimens in a solution of Congo red with ammonia after a short treatment in aqueous 10% KOH. The amyloid reaction of spores was assessed in Melzer’s reagent and observed both using the standard approach with initial observation immediately after mounting the object in the reagent (Melzer 1924), and also following prolonged (30 min) incubation (with pre-heating) in Melzer’s reagent, as described by Vizzini et al. (2020). Pileipellis elements near the pileus margin and the pileus center were observed and measured separately.

All microscopic observations were observed with an Olympus CX-41 microscope with an oil-immersion lens at a magnification of 1000×. All drawings of microscopic structures, with the exception of basidiospores, were made with a camera lucida using an Olympus U-DA drawing attachment at a projection scale of 2000×. Spores on lamella surfaces not attached to basidia were observed and illustrated using QuickPHOTO MICRO 3.2 software visualizing images captured by a Promicra 3-3CP camera. Enlarged scanned pictures of spores were used for measuring with an accuracy of 0.1 μm and for preparation of line drawings. Terminology of spore shapes follows Vellinga (1988). All other elements were measured with an accuracy of 0.5 μm. Thirty spores, terminal elements in pileipellis and caulocystidia were measured per collection, and 20 basidia and marginal cells were measured. For each species at least four specimens were selected, when available, from distant areas in order to represent morphological variation within the species. Both macro- and micromorphological observations are available in Suppl. materials 6, 7. The microscopic dimensions presented here are based on 30 measurements per collection and stated as a mean value plus/minus standard deviation; with values in parentheses showing measured minimum or maximum values. Q is the length/width ratio of spores. Statistics for morphological observations are presented in charts processed in the R statistical environment version 4.2.0 (R Core Team 2023). Non-scaled heatmap with taxon clustering using Hclust was calculated in *pheatmap* package version 1.0.12 (Kolde 2018). Boxplots with species order based on median value and color gradient and density plots with Kernel Density Estimations were calculated in *ggplot2* package version 3.4.4 (Wickham 2016). All species, including those published prior to our study, are presented with short descriptions in order to present a brief visualization of species morphology.

### ﻿﻿Typification and nomenclature

The type specimens of newly described taxa were selected from collections with successfully sequenced DNA regions used for phylogeny as the major criterion. Other criteria were quality of dried fungal specimens, quality of photos and presence of detailed micromorphological observations. Epitypes are proposed for species described prior to this study without successful sequencing of type collections. Selection of epitypes combine proximity to type collecting area with quality of sequence data. Epitypes, new names and other nomenclatural novelties were registered in MycoBank (https://www.MycoBank.org/).

### ﻿﻿Determination of δ^13^C and δ^15^N in samples

The elementary and stable isotopic compositions of nitrogen (N) and carbon (C) were determined from dried *Dermoloma* basidiomata using a Thermo Flash 2000 elemental analyser connected to a Thermo Delta V Advantage (Thermoscientific, Germany) isotope ratio mass spectrometer in a Continuous Flow IV system in the isotopic laboratory of the Institute of Geochemistry, Mineralogy and Mineral Resources (Charles University, Prague).

Samples wrapped in tin capsules were combusted, with the released gases (CO_2_, N_2_) being separated in a GC column and transferred to the MS source through a capillary. The isotope ratios are reported as delta (δ) values and expressed relative to the VPDB (Vienna Pee Dee Belemnite) reference for δ^13^C and to atmospheric nitrogen for δ^15^N.

The raw δ^13^C and δ^15^N values were normalized to the scale using a multiple-point linear regression based on certified international reference materials IAEA-CH-6, IAEA-CH-3 and IAEA-600 (International Atomic Energy Agency, Vienna) for carbon and IAEA-N-2, IAEA-N-1 and IAEA-NO-3 (International Atomic Energy Agency, Vienna) for nitrogen, run during the same sequence. The analytical precision, expressed as the long reproducibility for the homogenous standards, was within ± 0.2 ‰ for both δ^13^C and δ^15^N. The biplot with density curves visualization and ANOVA Tukey test were performed in the R statistical environment version 4.2.0 (R Core Team 2023) using *ggplot2* package version 3.4.4 (Wickham 2016).

## ﻿﻿Results

### ﻿﻿Phylogenetic analyses

**Figure 2.**
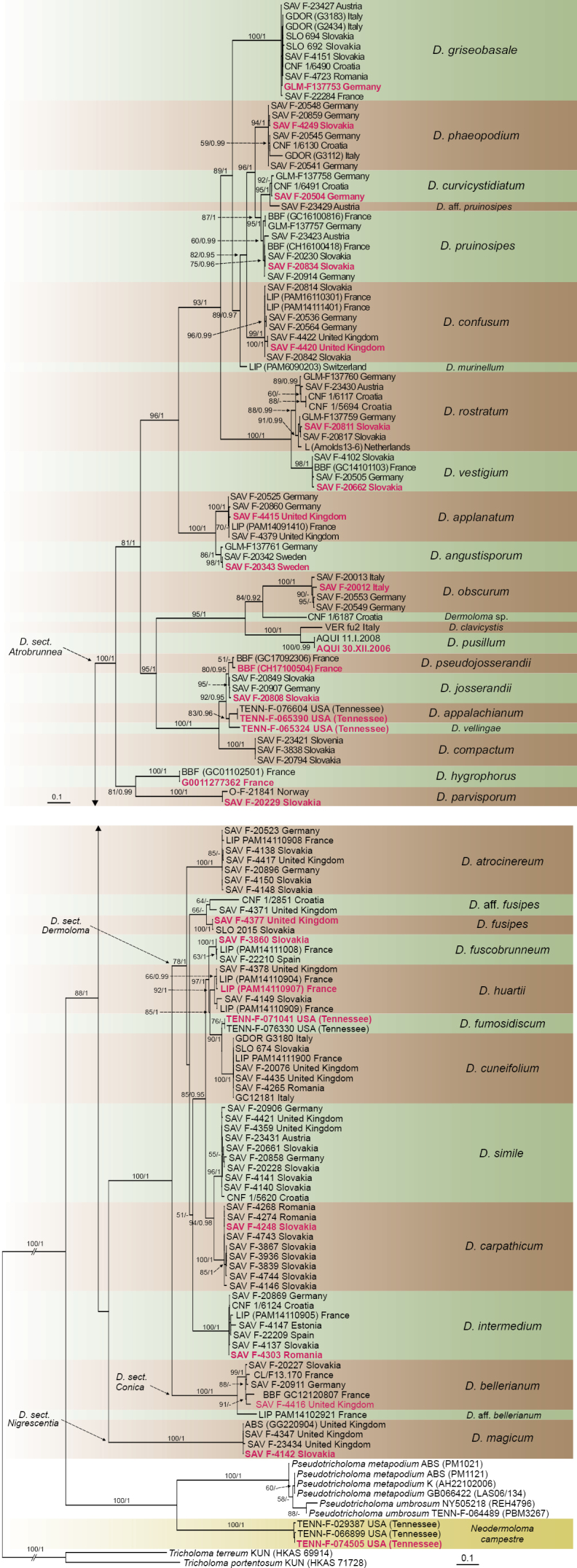
Phylogram generated by ML analysis based on combined sequence data of ITS, LSU, *rpb1*, *rpb2*, *mcm7* and *tef1a* showing phylogenetic relationships within genus *Dermoloma*. ML bootstrap support values greater or equal to 50% and Bayesian posterior probabilities greater or equal to 0.90 are indicated above or below the nodes. Sequences originating from type collections are labelled in red and bold.

Multilocus phylogenetic analysis was based on 147 samples putatively morphologically identified as *Dermoloma*, six *Pseudotricholoma* samples and two *Tricholoma* samples as an outgroup (Fig. 2). ITS region is represented by 153, LSU by 134, *rpb1* by 93, *rpb2* by 107, *mcm7* by 71 and *tef1a* by 92 sequences (Suppl. material 1). The majority of species are placed in a monophyletic lineage corresponding to the genus *Dermoloma*, with exception of three samples from the USA which are supported as a sister group to *Pseudotricholoma*. These samples were classified within a new genus *Neodermoloma* (erected below) with a single new species *N.campestre*. To resolve broader taxonomic relationships within *Tricholomataceae*, both the new genus and *Dermoloma* were analyzed with an expanded dataset containing additional genera from this family (see below). The branch support at notes above the species rank was relatively high and more than 80% of these nodes received strong support in both ML and BI analyses. All four sections previously proposed by Sánchez-García et al. (2021) were supported in our phylogeny. The two subgenera defined morphologically by spore amyloidity were not supported by our phylogeny due to the unresolved position of *D.magicum* Arnolds, which is the only species of D.sectionNigrescentia Adamčík. The phylogenetic grouping of other taxa corresponded to previous classifications. Members with inamyloid spores are placed in a well-supported lineage corresponding to D.subgen.Dermoloma (ML = 100, BI = 1.00). Remaining members of D.sectionAtrobrunnea Contu with amyloid spores received the same high support.

In total, within the genus *Dermoloma*, we recognized 28 species rank clades with at least two samples included and eight additional singletons. Ten sequences originating from *Dermoloma* type specimens were analyzed and placed in the phylogeny, all of them obtained from previous studies. In some cases, sequences from types are not included in the multilocus alignment due to low quality and short length, but they are included in phylogenetic analyses of the ITS region (Suppl. material 8). Based on the position of type sequences, *D.atrocinereum*, *D.cuneifolium*, *D.hygrophorus* Joss. and *C.phaeopodium* P.D. Orton were names assigned to European species clades with at least one recent collection by authors of this study. Additionally, *D.clavicystis* Voto, *D.hymenocephalum* and *D.pusillum* Contu represented well-supported species which were not collected by the authors. In agreement with Sánchez-García et al. (2021), the type sequence of *D.emilii-dlouhyi* Svrček was placed in the *D.cuneifolium* species clade and the type sequence of D.pragensef.obscurum Consiglio & Contu in the *D.atrocinereum* species clade. Consequently, both these names are treated as synonyms. Based on the morphological match, the species clade with the type sequence of *D.alexandri* Consiglio is identified as *D.intermedium* Bon. Other species-rank clades with names that are assigned based on morphology are *D.bellerianum* Bon, *D.fuscobrunneum* P.D. Orton, *D.josserandii* Dennis & P.D. Orton, *D.magicum* and *D.murinellum* E. Horak.

Sixteen European species-rank clades represented by at least two samples from different countries could not be assigned to any available published name and therefore are described here as new species: *D.angustisporum*, *D.applanatum*, *D.carpathicum*, *D.compactum*, *D.confusum*, *D.curvicystidiatum*, *D.fusipes*, *D.griseobasale*, *D.huartii*, *D.obscurum*, *D.parvisporum*, *D.pruinosipes*, *D.pseudojosserandii*, *D.rostratum*, *D.simile* and *D.vestigium*. The type sequence of *D.hymenocephalum*, the only *Dermoloma* species previously described from North America, did not match any of our recent collections, and additionally three new North American species clades are described here as *D.appalachianum*, *D.fumosidiscum* and *D.vellingae*.

Almost all the species mentioned above received strong ML and BI support, but there are a few cases where we decided after careful checking of original sequence data, not to accept the supported clades as independent species. The most notable of these are *D.huartii* and *D.rostratum*, which both included pairs of supported subclades, but the support for these subclades was not congruent among the individual loci which were analyzed. In contrast, *D.josserandii* and *D.pseudojosserandii* are recognized as different but closely related species, despite the lack of statistical support in the multilocus tree. In these two cases, the samples represented in multilocus analyses showed consistent differences in all analysed regions, except for *rpb1*, and this conclusion is also supported by differences in spore shape.

Names were assigned also to three singletons in the multilocus tree. First, *D.clavicystis* is represented by only a single ITS sequence originating from the Italian type collection. It was confirmed as a good species, distinct from all other species collected by the authors. Second, *D.murinellum* from Switzerland is supported as a distinct species. This is based on a recent collection from the same area as the type in Switzerland, with morphology of the type specimen and the protologue in Horak (1987). Lastly, the American collection TENN-F-065324 was recognized as a well-defined phylogenetic species by four loci (ITS, LSU, *rpb1*, *rpb2*), and therefore, combined with the morphological differences observed, we describe it below as *D.vellingae*. Three other singleton samples, supported as different from closest relatives by multilocus phylogenetic analyses, were provisionally labeled as D.aff.pruinosipes, D.aff.fusipes and D.aff.bellerianum. They represent potentially independent species, but because of limited material and close affinity to sister species, we did not describe them here as new species. Finally, two Croatian singleton samples in the tree are labelled also as *Dermoloma* sp. because they are only represented by single ITS sequences.

**Figure 3.**
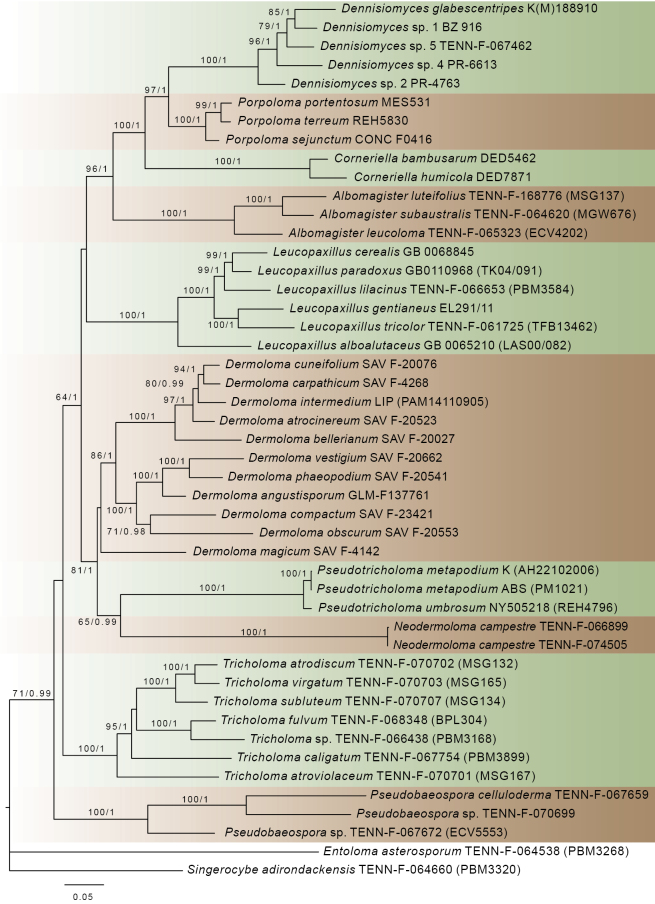
Phylogram showing phylogenetic relationships within the family *Tricholomataceae* generated by ML analysis based on combined sequence data of ITS, LSU, *rpb1*, *rpb2* and SSU. ML bootstrap support values greater or equal to 50% and Bayesian posterior probabilities greater or equal to 0.90 are indicated above or below the nodes.

Results of multilocus phylogenetic analyses at the genus level, with broader sampling across *Tricholomataceae*, showed identical topology in ML and BI (Fig. 3). There were five major supported lineages within the family, from the top: the first clade contains the genera *Albomagister*, *Corneriella*, *Dennisiomyces* and *Porpoloma*; the second clade is represented by *Leucopaxillus*; the third by *Dermoloma*, *Neodermoloma* and *Pseudotricholoma*; the fourth by *Tricholoma*; and the fifth by *Pseudobaeospora*. The first three clades form a supported lineage within *Tricholomataceae* (ML = 64, BI = 1.00). All *Dermoloma* species are clustered together, but the D.subgenusDermoloma is not supported as a monophyletic clade because the position of *D.magicum* is unresolved. *Pseudotricholoma* and *Neodermoloma* are supported (ML = 65, BI = 0.99) as sister genera within this clade.

### ﻿﻿Morphology

**Figure 4.**
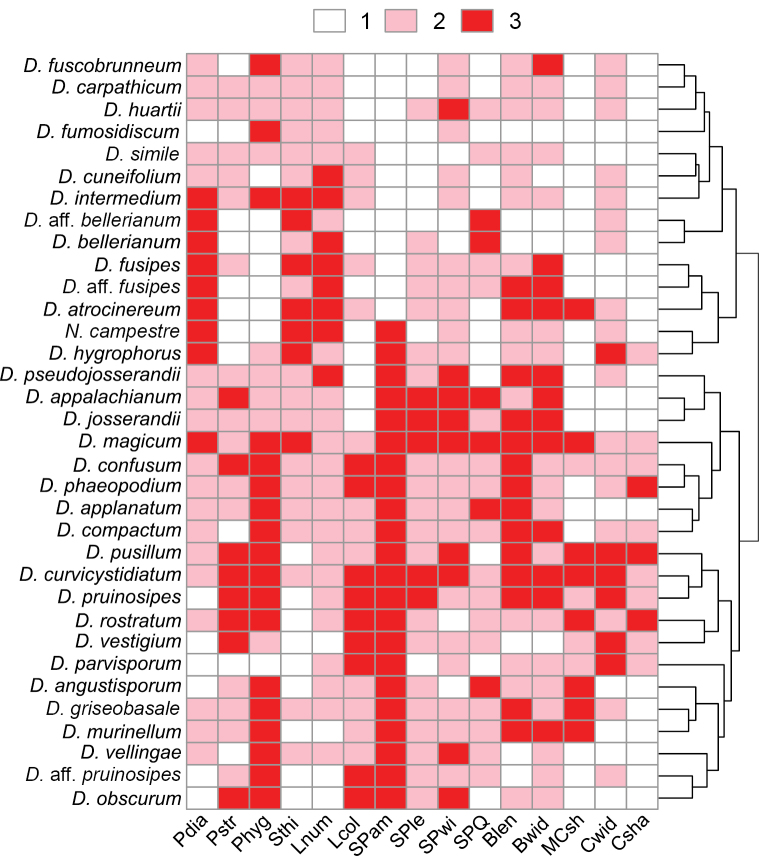
Heatmap showing clustering based on important morphological differences in Dermoloma (D.) and Neodermoloma (N.). Characters are classified into three categories. **Pdia** – pileus diameter: 1 = up to 12 mm, 2 = 13–25 mm, 3 = more than 25 mm; **Pstr** – striation near pileus margin: 1 = none, 2 = indistinct, 3 = distinct; **Phyg** – pileus hygrophaneity: 1 = none, 2 = indistinct, 3 = distinct; **Sthi** – stipe thickness: 1 = up to 2 mm, 2 = 2–5 mm, 3 = more than 5 mm; **Lnum** – number of lamellae near stipe: 1 = up to 20, 2 = 21–30, 3 = more than 30; **Lcol** – lamellae color: 1 = white to ochraceous-gray, 2 = brownish gray to brownish ochraceous, 3 = brown to dark gray-brown; **SPam** – spore amyloidity: 1 = inamyloid, 3 = amyloid; **SPle** – spore length: 1 = up to 5.5 µm, 2 = 5.6–6.5 µm, 3 = more than 6.5 µm; **SPwi** – spore width: 1 = up to 3.6 µm, 2 = 3.7–4.2 µm, 3 = more than 4.2 µm; **SPQ** – spore length/width ratio: 1 = less than 1.4, 2 = 1.4–1.6, 3 = more than 1.6; **Blen** – basidia length: 1 = up to 25 µm, 2 = 26–28 µm, 3 = more than 28 µm; **Bwid** – basidia width: 1 = less than 6 µm, 2 = 6–6.5 µm, 3 = more than 6.5 µm; **MCsh** – shape of marginal cells: 1 = cylindrical or clavate and apically obtuse, 2 = lageniform and apically constricted or attenuated, 3 = lobate and diverticulate or branched; **Cwid** – caulocystidia width: 1 = less than 5.5 µm, 2 = 5.5–8 µm, 3 = more than 8 µm; **Csha** – shape of caulocystidia: 1 = cylindrical or narrowly clavate and apically obtuse, 2 = broadly clavate or sphaeropedunculate or obpyriform, 3 = lobate or diverticulate or branched.

All samples collected by the authors were assigned to a species based on phylogenetic analysis only, using ITS sequences and where available additional loci (Suppl. material 8). Collections grouped in this way were used in further morphological analyses. We observed only a few species that can be morphologically identified in the field, for instance *D.magicum* characterized by large basidiomata with wounded parts of the basidiome turning black, or *D.hygrophorus* with pale grayish cream colors and distant lamellae. Our detailed morphological analysis, guided by phylogenetic information, identified several additional morphological characters not previously mentioned in the literature. Analysis of these characters shows morphological differentiation between all species (Suppl. materials 6, 7; Fig. 4). The morphological groupings were only partially congruent with phylogenetic analyses, for example subgenus Dermoloma is split into three morphological groups, but the morphological grouping of the core clade with *D.cuneifolium* and D.sectionConica corresponded to our phylogeny. In the majority of cases it was necessary to combine morphological and phylogenetic information to circumscribe individual species. Below, we present details of the new morphological features which are described in the species descriptions of the Taxonomy part.

**Figure 5.**
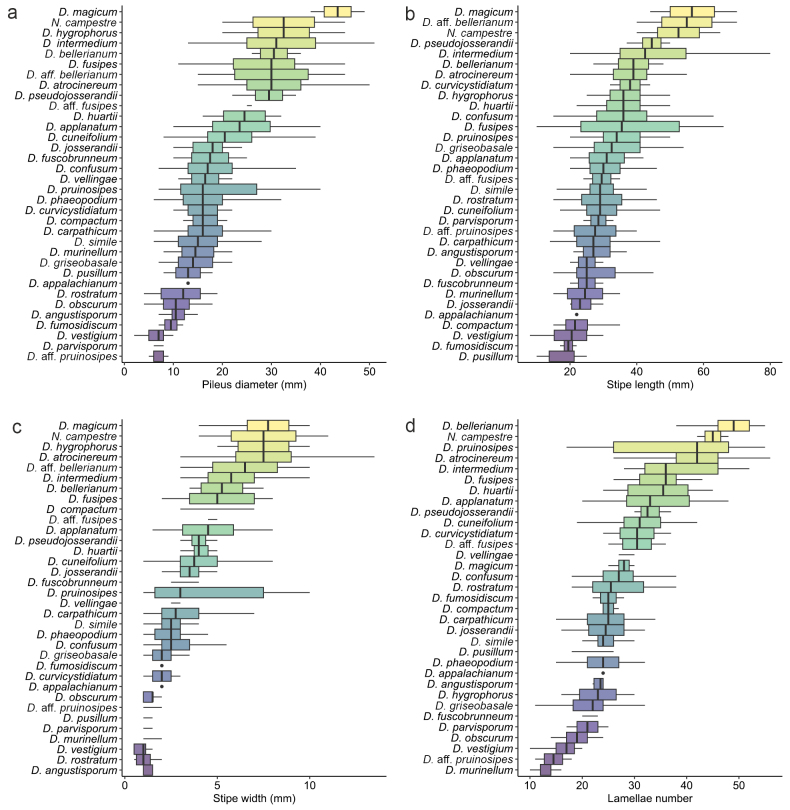
Boxplots showing statistical differences in macromorphological characters observed on Dermoloma (D.) and Neodermoloma (N.). **a** Pileus diameter; **b** stipe length; **c** stipe width; **d** lamellae number. Observations are presented in Suppl. material 6.

**Figure 6.**
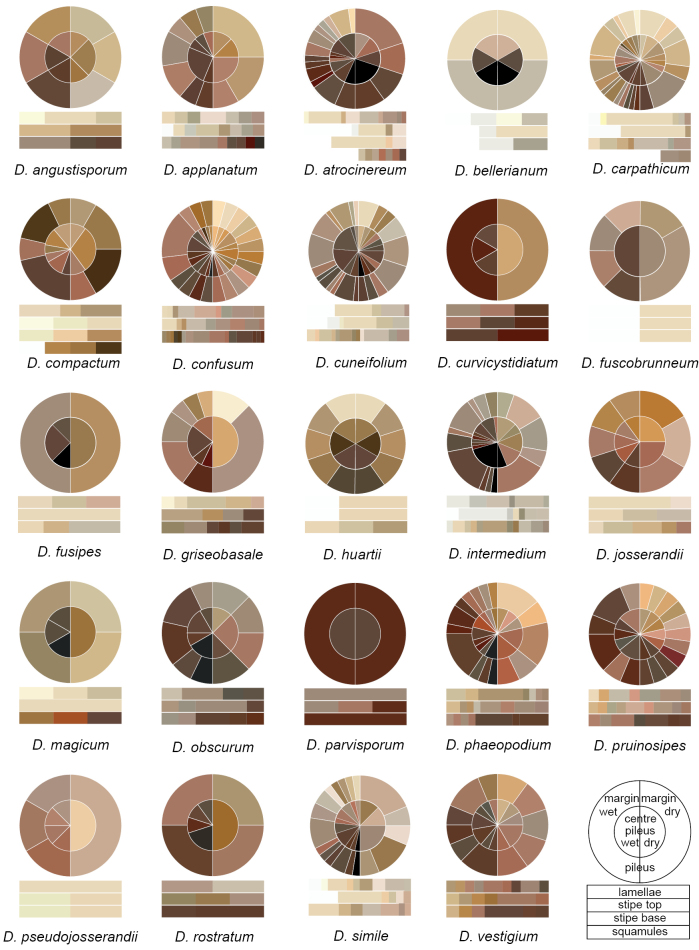
Chromatic diagram showing variation of pileus, lamellar and stipe surfaces on *Dermoloma*. Colors observed on fresh basidiomata were coded and transferred from Kornerup and Wanscher (1978). These observations are presented in Suppl. material 6.

**Figure 7.**
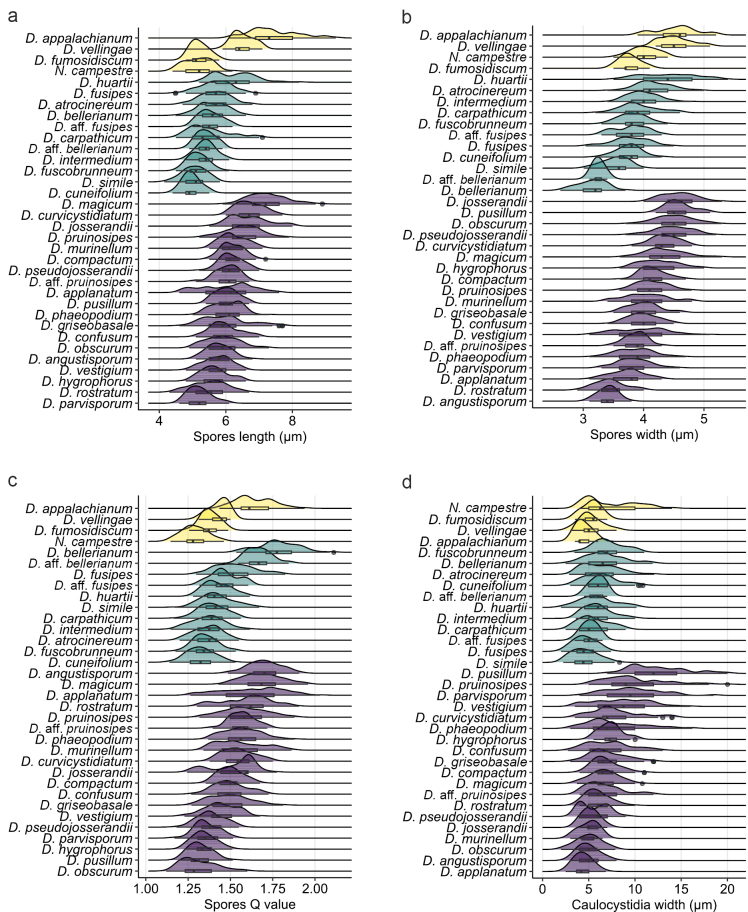
Density plots showing statistical differences in micromorphological characters observed in Dermoloma (D.) and Neodermoloma (N.). North American species are indicated in yellow, European species with inamyloid spores in green and European species with amyloid spores in purple. **a** Spore length; **b** spore width; **c** spore Q; **d** caulocystidia width. Average values are presented in Suppl. material 7.

**Figure 8.**
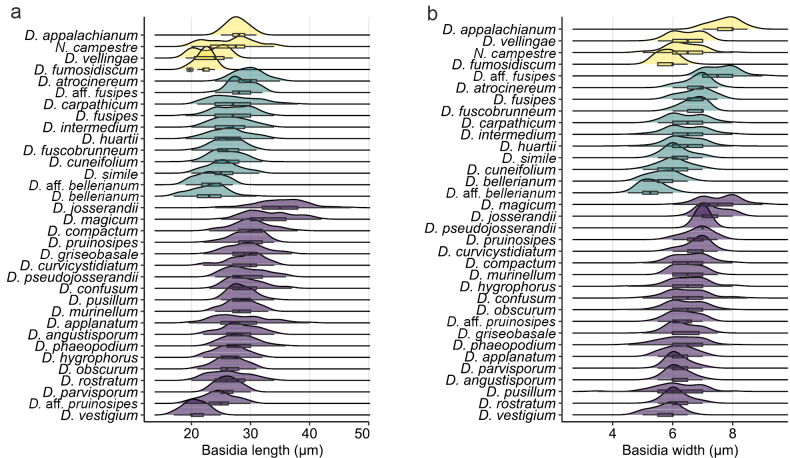
Density plots showing statistical differences in micromorphological characters observed on Dermoloma (D.) and Neodermoloma (N.). North American species are indicated in yellow, European species with inamyloid spores in green and European species with amyloid spores in purple. **a** Basidia length; **b** basidia width. Average values are presented in Suppl. material 7.

(1) ***Pileus***. Pileus diameter was very variable within species, but for some species pilei were consistently only up to 10 mm in diam. (e.g. *D.vestigium* and *D.parvisporum*), while pilei of other species exceeded 20 mm. The species with the largest basidiomata, *D.magicum*, had mature pilei usually larger than 40 mm diam. (Fig. 5). This character was also included in the key but should be used with caution, especially when a collection comprises only one or few basidiomata. *Dermoloma* species showed little variation in pileus shape, and most of the variation seen falls into the range observed within a single species. For example, we observed both pilei with an umbo and a central depression among different collections of *D.intermedium*. A few species had a distinctly striate pileus margin which is also used in our key (e.g. *D.simile* and *D.rostratum*). The pileus surface was always glabrous, cracking under exposure to dry air and often with a rough or rugulose relief. However, these features were not taxonomically relevant at the species level. In contrast, the color change of the pileus surface following drying to a paler color (hygrophanous pilei) was identified as a useful separating character. The colors usually faded first at the margin, but in the *D.josserandii* lineage they typically first faded near the pileus center. Colors of pilei are often used as distinguishing characters in the literature, but it is dependent on the hygrophanous state, as well as differences between the pileus margin and center (Fig. 6). We also observed differences between young and old stages; for this reason, we excluded very young basidiomata and we recommend to collect sufficient material represented by several basidiomata at different stages. Some species showed a distinct color contrast between the pileus margin and center (e.g. *D.bellerianum*), while others showed more or less homogenous colors (e.g. *D.compactum*).

(2) **Stipe**. The stipe length was very variable and was not identified as a taxonomically relevant character; however, stipe width had a crucial importance for species identification (Fig. 5). In combination with pileus diameter, stipe width can be used to sort basidiomata into more robust (tricholomatoid), intermediate (collybioid) or small/tiny (mycenoid) types. The diameter of the pileus and stipe usually agrees with these categories, and there were only a few cases of disproportioned dimensions (e.g. *D.compactum* has a relatively wide stipe but moderately large pileus). The stipe surface is usually pruinose near the lamellae and glabrous towards the base, but some species are distinctly pruinose all along the stipe (e.g. *D.pruinosipes*) or have white or brownish squamules near the stipe base (e.g. *D.atrocinereum*). Members with inamyloid spores (D.subgenusDermoloma) usually had paler stipe colors, especially near the lamellae attachment compared to more uniform and darker colored stipes of members with amyloid spores (D.subgenusAmylospora) (Fig. 6).

(3) **Lamellae**. Lamellar density (L), defined as number of lamellae at the point of attachment to the stipe, was an important character. It was, for example, used in combination with pileus diameter to distinguish species with inamyloid spores with higher and lower L (Fig. 5) and it may also help to differentiate species pairs (e.g. *D.josserandii* and *D.pseudojosserandii*). Lamellar attachment may be variable among individual collections of a species. The majority of species usually have narrowly adnate and emarginate lamellae, but a few species can have broadly adnate to decurrent lamellae (e.g. *D.vestigium*). Lamellar color usually corresponds to the color of the stipe near the lamellar attachment point (Fig. 6). Pale-colored lamellae defined for example *D.josserandii* and related taxa. We did not observe any differences in lamellar edges that were useful for species recognition.

(4) **Context**. The context of *Dermoloma* members was typically fragile at least in the pileus, which typically cracked radially when handling mature basidiomata. The odor was farinaceous in all *Dermoloma* species, but may be indistinct in small species (e.g. *D.parvisporum*) and only appeared when the context was freshly crushed. *Neodermoloma* has an unpleasant component in the odor. The color of the context usually corresponded to the color of the surface but was somewhat paler. Context characteristics can be used for species recognition only in a few cases, for example in *D.magicum*, where the context turned yellow inside the stipe and became black after longer air exposure. *Dermolomahygrophorus* can be easily recognized from other *Dermoloma* species by the unusually thick context in the pileus.

(5) **Spores.** These are the most frequently used microscopic structures for *Dermoloma* identification in the literature. Previous phylogenetic analysis combined with spore dimensions (length, width and Q value) demonstrated that only some closely related and/or similar species can be distinguished by spore size (Sánchez-García et al. 2021). However, we recommend to supplement spore dimensions with additional supporting characters due to the variability of spore size within species usually caused by the presence of two-spored basidia (Fig. 10). For example, collections with prevailingly two and four sterigmata were observed in *D.bellerianum* and *D.vestigium*. The majority of spores were ellipsoid to narrowly ellipsoid, but they also often showed slightly asymmetric shapes in transition to ovoid or amygdaloid. Since the visibility of this asymmetric shape depends on spore orientation and can also vary among spores of individual collections, we did not describe it for the majority of species. However, a few species, like *D.magicum*, had distinctly amygdaloid spores. Spore amyloidity had a strong phylogenetic significance. Spores of D.subgenusDermoloma were consistently inamyloid while spores of D.subgenusAmylospora and the genus *Neodermoloma* were always distinctly amyloid. Spores released from hymenia and observed in Melzer’s reagent without any other elements in the background sometimes appeared to be darker. For reliable recognition of spore amyloidity, we recommend to search the hymenium for collapsed spores which are completely black in case of an amyloid reaction. Prolonged mounting or preheating in Melzer’s reagent (following Vizzini et al. 2020) did not result in any different results compared to direct observation. Spores with dextrinoid, thick walls were observed multiple times. They were more frequent in some species like *D.carpathicum*, but were not present in all collections and usually they were more frequent towards the lamellae edges. Dextrinoid thick walls were also observed in mounts of lamellae from several collections of species with amyloid spores, e.g. *D.magicum* and *D.vestigium*.

(6) **Basidia**. Basidia showed relatively low variability in size among *Dermoloma* species, but in a few cases, they can be combined with other characters to support species identifications (Fig. 8). For example, among common and similar species with inamyloid spores, *D.cuneifolium* had on average narrower basidia than *D.atrocinereum* and *D.intermedium*, whereas the last two species were different in basidia length. Basidia with four sterigmata were observed in most collections, but basidia with one, two or three sterigmata were sporadically observed in some collections. There were a few species in which basidia predominantly bore two sterigmata, including *D.bellerianum*, *D.huartii*, *D.obscurum*, *D.rostratum* and *D.vestigium*, but all these collections had clamp connections present on the base of the basidia. The only exception was D.aff.bellerianum which lacked clamp connections on basidia with two sterigmata.

(7) **Lamellar edges**. Marginal cells near lamellar edges were usually undifferentiated and similar to the basidioles in D.subgenusDermoloma and the genus *Neodermoloma*. These elements showed a higher variation among members of D.subgenusAmylospora (Suppl. material 9), but it is sometimes difficult to locate them under a microscope and to make good microscopic preparations. Unfortunately, marginal cells were the only morphological structures showing apparent differences between *D.confusum* and *D.phaeopodium*. When possible, we recommend to use observations of marginal cells in combination with other microscopic structures when they appear in the key.

(8) **Pileipellis**. The pileipellis of all studied taxa had a similar structure forming a transition between a hymeniderm and an epithelium. Inflated cells in the pileipellis were predominantly obpyriform or sphaeropedunculate and were arranged in one to three layers. We did not observe differences between the pileus margin and the center within individual pilei. Older basidiomata showed bigger terminal cells in the pileipellis with occasionally thickened walls, especially near the septa. The only taxonomically significant difference was observed in *Neodermoloma* which differed from all *Dermoloma* members by smaller terminal cells (Suppl. material 9). We observed brownish to dark brown parietal and incrusted pigments with an intensity corresponding to field appearance of basidiomata. Incrusted pigments were present especially at the base of terminal cells and on subterminal cells. In general, pileipellis characters were either variable within collections or show low variability among species. Therefore, they were not used for species circumscriptions.

(9) **Caulocystidia**. These were present in all species and collections and were usually one-celled. They usually occur in clusters, close to the hymenium. They can completely cover the stipe surface and also contain elements similar to basidioles. Towards the stipe base they become more scattered and may even be completely absent. In D.subgenusDermoloma, clavate or subcylindrical caulocystidia had yellow, granulose incrustations. DermolomasubgenusAmylospora had no incrustations but showed high variability of caulocystidia shapes and sizes (Fig. 7, Suppl. material 9). Within D.subgenusDermoloma, caulocystidia were used for species recognition in only one case (*D.fuscobrunneum*). Within D.subgenusAmylospora, caulocystidia were among the most important elements for species identifications.

### ﻿﻿Stable isotopes δ^13^C and δ^15^N

**Figure 9.**
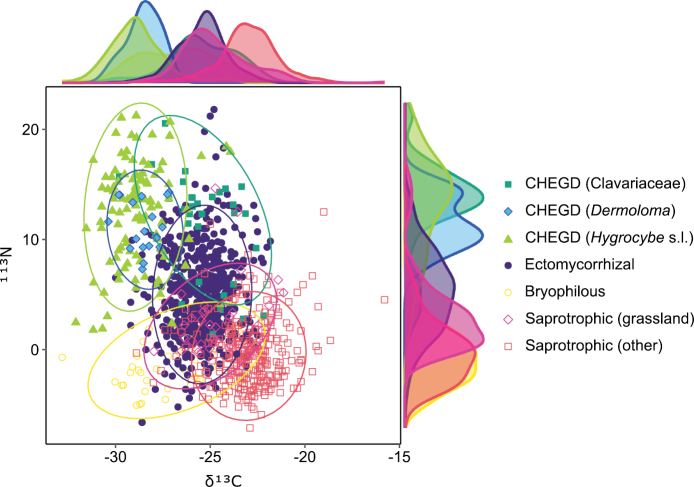
Biplot diagram showing differences in δ^13^C and δ^15^N between fungi with different trophic strategies. Isotopic measurements with data sources are in (Suppl. material 10). Ellipses indicate 95% confidence intervals. Curve plots show the density of observations per group on the N and C stable isotope axis separately.

The final data matrix includes 19 ratios of stable isotopes recorded as δ^13^C and δ^15^N values from 15 *Dermoloma* species and an additional 1084 measurements (Suppl. material 10) retrieved from the literature (Taylor et al. 2003; Trudell et al. 2004; Hart et al. 2006; Zeller et al. 2007; Mayor et al. 2009; Seitzman et al. 2011; Tedersoo et al. 2012; Birkebak et al. 2013; Sánchez-García and Matheny 2017; Halbwachs et al. 2018; Korotkin et al. 2018) and classified into CHEGD groups *Clavariaceae*, *Dermoloma* and *Hygrocybe* (biotrophic grassland fungi), ectomycorrhizal fungi (ECM), bryophilous fungi and grassland and other saprotrophs. Distribution of *Dermoloma* isotopic values in a two-dimensional chart was very similar and highly overlapping with *Hygrocybe* s.l. (Fig. 9). High δ^15^N ratios were also observed in *Clavariaceae*. Trophic mode had a significant effect on the proportion of both δ^13^C (ANOVA, F = 268.14, p < 0.05) and δ^15^N (ANOVA, F = 240.38, p < 0.05) isotopes values. *Dermoloma* δ^13^C ratios were similar to *Hygrocybe* s.l. but different from all other trophic groups (ANOVA Tukey test p < 0.05). *Dermoloma* δ^15^N ratios were similar to other CHEGD fungi and significantly different from all other groups (Suppl. material 11).

**Figure 10.**
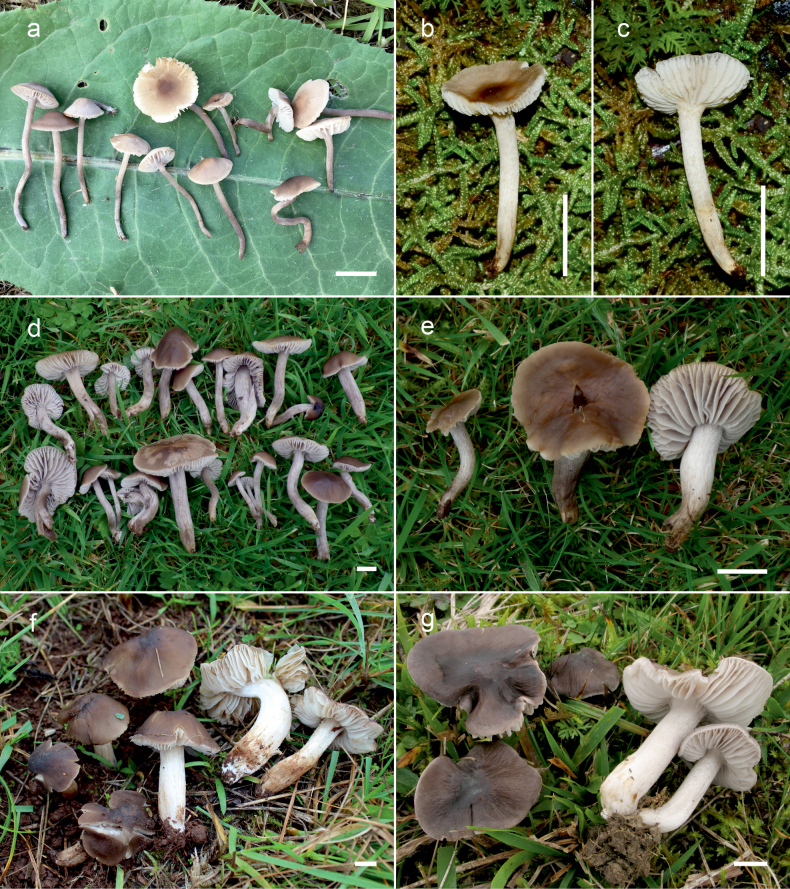
Basidiomata of *Dermoloma* in field appearance. **a***Dermolomaangustisporum* (SAV F-20343, holotype), photo S. Jančovičová; **b, c***Dermolomaappalachianum* (TENN-F-076604), photo D. Grootmyers; **d***Dermolomaapplanatum* (SAV F-4415, holotype), photo D. Harries; **e***Dermolomaapplanatum* (SAV F-4419), photo D. Harries; **f***Dermolomaatrocinereum* [BBF (*GC16112316*)], photo G. Corriol; **g***Dermolomaatrocinereum* (SAV F-20523), photo S. Jančovičová. Scale bar: 10 mm.

### ﻿﻿Taxonomy

#### ﻿﻿Key to *Dermoloma* and *Neodermoloma* species

**Table:** 

1	Spores inamyloid (D.subgenusDermoloma)	**2**
–	Spores amyloid (D.subgenusAmylospora)	**12**
2	Spores on average < 3.4 µm wide, Q ≥ 1.6 (D.sectionConica)	**3**
–	Spores on average ≥ 3.4 µm wide, Q < 1.6 (D.sectionDermoloma)	**4**
3	Spore Q ≥ 1.7	***D.bellerianum***
–	Spore Q < 1.7	**D.aff.bellerianum**
4	Stipes less than 4 mm wide; L < 30; pileus diameter usually < 20 mm	**5**
–	Basidiomata usually larger; stipe more than 4 mm wide; L ≥ 30; pileus diameter ≥ 20 mm	**8**
5	Pilei up to 12 mm in diam., strongly rugulose near center; North America	***D.fumosidiscum***
–	Pilei usually wider than 12 mm, often not distinctly rugulose near center; Europe	**6**
6	Lamellae and stipes pale ochraceous-gray to almost white; pileus margins not striate when wet; caulocystidia wider than 5.5 µm	***D.fuscobrunneum***
–	Lamellae often darker; stipes near base usually darker brownish to grayish brown; pileus margins usually translucently striate when wet; caulocystidia usually narrower than 5.5 µm	**7**
7	Spores on average up to 3.6 µm wide and/or Q ≥ 1.4; stipe bases never with darker fibrils or squamules	***D.simile***
–	Spores on average wider than 3.6 µm and/or Q < 1.4; stipe bases often with darker fibrils or squamules	***D.carpathicum***
8	Stipes up to 5 mm wide; pilei usually < 30 mm in diam; lamellae pale ochraceous-gray to white; spores on av. longer than 5.5 µm and often wider than 4 µm	***D.huartii***
–	Basidiomata larger, or lamellae darker brownish, or spores smaller	**9**
9	Stipes up to 5 mm wide; pilei usually < 30 mm in diam; spores on av. shorter than 5.2 µm long; basidia on average narrower than 6.5 µm	***D.cuneifolium***
–	Basidiomata larger, or spores longer, or basidia wider	**10**
10	Stipes often wider than 6 mm, often with darker brown fibrils or squamules near bases; spores on average 4 µm or wider, Q on average ≤ 1.45; basidia on average longer than 28 µm	***D.atrocinereum***
–	Stipes usually narrower and without darker fibrils or squamules near bases; spores on average narrower than 4 µm or Q on average > 1.45, or basidia shorter than 28 µm	**11**
11	Pilei distinctly hygrophanous	***D.intermedium***
–	Pilei not hygrophanous	***D.fusipes*** or **D.aff.fusipes**
12	Basidiomata relatively robust, tricholomatoid; stipes usually ≥ 5 mm wide; pilei usually ≥ 25 mm in diam.; pileus and stipe surface with black spots or pale colored	**13**
–	Basidiomata smaller, usually collybioid or mycenoid; either stipes < 5 mm wide or pilei < 25 mm in diam.; pileus and stipe surface not with black spots and always with darker brown colored parts	**15**
13	Surface of basidiomata spotted black with drying or maturing; pileus margins brown; spores on average ≥ 7 µm long (D.sectionNigrescentia)	***D.magicum***
–	Surface of basidiomata without black spots or patches; pileus margins orange-gray, gray, grayish to almost white; spores < 7 µm (D.sectionAtrobrunnea)	**14**
14	Lamellae distant, L up to 30; hyphal terminations in pileipellis mainly ≥ 12 µm wide; Europe	***D.hygrophorus***
–	Lamellae relatively crowded, L ≥ 30; hyphal terminations in pileipellis mainly < 12 µm wide; North America	***Neodermolomacampestre***
15	Basidiomata collybioid to tricholomatoid; stipes usually > 2 mm wide; lamellae and stipes pale ochraceous-gray to almost white, stipes only slightly darker towards bases; pilei hygrophanous and color fading first near the center	**16**
–	Basidiomata mycenoid or if basidiomata collybioid or tricholomatoid; then lamellae and stipes darker brown and stipes brown to dark brown near bases; if pilei hygrophanous then pallescent first near the margin	**19**
16	Mature stipes towards the base with distinct brown fibrils or squamules; pilei brown to dark brown; spores on average up to 4.2 µm wide	***D.compactum***
–	Stipes without distinctly darker fibrils or squamules; pileus sometimes light brown at least when dry; spores usually > 4.2 µm wide	**17**
17	Pileus mainly brown to dark brown; basidia ≤ 25 µm long and ≤ 6.5 µm wide; North America	***D.vellingae***
–	Pileus when dry light brown; basidia > 25 µm long and > 6.5 µm wide; Europe	**18**
18	Usually L < 30; stipes sometimes with distinctly darker bases; spores sometimes ≥ 6.5 µm long; marginal cells up to 8 µm wide	***D.josserandii***
–	Usually L ≥ 30 lamellae; stipes with evenly pale colors along entire length; spores < 6.5 µm long; marginal cells > 8 µm wide	***D.pseudojosserandii***
19	Basidiomata collybioid; stipes usually ≥ 2 mm wide or pilei ≥ 15 mm in diam.	**20**
–	Basidiomata mycenoid; stipes always < 2 mm wide, pilei usually < 15 mm in diam.	**25**
20	Stipes fusiform; pilei soon expanding to plane; caulocystidia ≤ 5 µm wide, narrowly clavate or cylindrical	***D.applanatum***
–	Shapes of pilei and stipes usually different; caulocystidia > 5 µm wide and distinctly clavate or even more inflated	**21**
21	Spores on av. > 6.5 µm long and > 4 µm wide; basidiomata brown to dark brown in all parts, only pilei becoming ochraceous-brown when dry	***D.curvicystidiatum***
–	Spores smaller or basidiomata when wet with pale brown to ochraceous-brown; colors on pilei, lamellae or on stipes near lamellae attachment pale brown to ochraceous-brown	**22**
22	Stipes distinctly pruinose along all length when young; marginal cells with long narrow, flexuous apical projections	***D.pruinosipes***
–	Stipes when young only pruinose near lamellae; marginal cells clavate or lageniform and without flexuous projections	**23**
23	Lamellae relatively pale, brownish ochraceous-white, ochraceous-gray, brownish gray to brownish ochraceous; marginal cells clavate and often lobate or with lateral projections	***D.griseobasale***
–	Lamellae often darker grayish brown; if marginal cells clavate, than not lobate and without projections	**24**
24	Marginal cells lageniform or cylindrical, apically usually constricted and often also mucronate	***D.confusum***
–	Marginal cells clavate, apically obtuse	***D.phaeopodium***
25	Lamellae pale colored, yellowish, ochraceous-gray, brownish gray; spores narrow, on av. Q ≥ 1.7	***D.angustisporum***
–	Lamellae not pale colored or if lamellae pale colored then spore Q < 1.7	**26**
26	Spores on av. ≥ 7 µm long; North America	***D.appalachianum***
–	Spores on av. < 7 µm long; Europe	**27**
27	Caulocystidia on av. > 8 µm wide, regularly broadly clavate, sphaeropedunculate or ellipsoid, obtuse, not diverticulate	**28**
–	Caulocystidia usually up to 8 µm wide, if wider than curved, lobate or diverticulate	**29**
28	Caulocystidia short, on av. ≤ 30 µm long, often ellipsoid or obpyriform; margins of pastures in temperate Europe	***D.vestigium***
–	Caulocystidia longer, on av. > 30 µm long, broadly clavate or sphaeropedunculate; in moss among scarce herbal vegetation in Mediterranean Europe	***D.pusillum***
29	Spores on av. ≥ 4 µm wide; lamellae young and fresh brown to dark brown	***D.obscurum***
–	If spores on av. > 4 µm wide; then lamellae paler gray when young	**30**
30	Pileus with distinctly striate margin when wet; caulocystidia and marginal cells lobate and diverticulate	***D.rostratum***
–	Pileus margin not distinctly striate when wet; caulocystidia not diverticulate, marginal cells attenuated or clavate and similar to basidioles	**31**
31	Spores on av. ≤ 5.5 µm long, Q ≤ 1.4; marginal cells with attenuated and flexuous apical part	***D.parvisporum***
–	Spores on av. > 5.5 µm long, Q > 1.4; marginal cells mainly clavate and similar to basidioles	**32**
32	Pileus up to 10 mm in diam.; known from a single collection in low altitude with temperate climate	**D.aff.pruinosipes**
–	Pileus more than 10 mm in diam.; known only from higher elevations in the Alps	***D.murinellum***

##### ﻿ Dermoloma

Taxon classificationAnimaliaAgaricalesTricholomataceae

(J. Lange) Singer, Mycologia 48: 724. 1956.

121C5369-72B5-57ED-A899-5F954B6EFF56

Tricholoma [II] *Dermoloma* J.E. Lange, Dansk Bot Ark 3: 35. 1933. Basionym.

###### Type species.

*Dermolomacuneifolium* (Fr.) Singer ex Bon

###### Notes.

The valid publication of the genus name was assigned to Herink (1958), who provided a Latin diagnosis with typification (Sánchez-García et al. 2021). However, Singer (1956) introduced the name as a new combination at genus rank based on the infrageneric name in *Tricholoma* described by Lange (1933). This has already been pointed out by Arnolds (1992), who, however, mistakenly thought Lange had used the rank “stirps”. Lange did not indicate a rank for *Tricholoma* [II] *Dermoloma*. Singer (1956) treated this basionym at the rank of a subgenus but this does not contradict the valid publication as a name at a new rank (see Turland et al. 2018; Art. 41.6). Donk’s (1962) statement that Lange’s name would not be valid is wrong under the current Code (see Art. 37.3).

##### ﻿ Dermoloma angustisporum

Taxon classificationAnimaliaAgaricalesTricholomataceae

Jančovič. & Karich, sp. nov.

746B27EF-4786-59B2-9027-A7EB874FBD1A

856329

Figs 10a , 11

###### Holotype.

Sweden • Medelpad, Börgsjö, Talja Gården, terrestrial on semi-natural, mown meadow, 27 Aug 2018, J. M. Traba-Velay (SAV F-20343).

###### Etymology.

With narrow spores compared to other *Dermoloma* species.

###### Diagnosis.

European species; basidiomata small and slender; lamellae pale yellowish white to brownish gray; spores amyloid and narrow, Q > 1.7.

***Pileus*** (7–)9–20 mm; convex, soon expanding to plane, sometimes indistinctly umbonate; margin indistinctly translucently striate when wet, when dry radially cracked; surface smooth, pruinose, hygrophanous; color near margin dark brown (6F4), brown (6E4) to light brown (5D4), when dry grayish brown (5D3), brownish ochraceous (5C3) to ochraeous-gray (5C2, 6C2), near center dark brown (6F4, 7F4) to brown (6E4), when dry light brown (5E4, 5E5), grayish brown (5E3) to light brown (5D4). ***Stipe*** 21–32(–37) × 1–1.5 mm; cylindrical, sometimes narrowed towards the base, flexuous; surface finely longitudinally striate, pruinose near lamellae, finely fibrillose towards the base; color near lamellae brownish gray (5C3, 5D3), near the base grayish brown (6E3), brown (6E4) to dark brown (6F3). ***Lamellae*** L = 21–28, l = 0–3; 1.5–3 mm wide; adnate-emarginate and decurrent with tooth; color yellowish white (4A2), ochraeous-gray (5B2) to brownish gray (5C2); edges entire or slightly irregular, rarely serrulate. ***Context*** fragile; odor weakly farinaceous.

**Figure 11.**
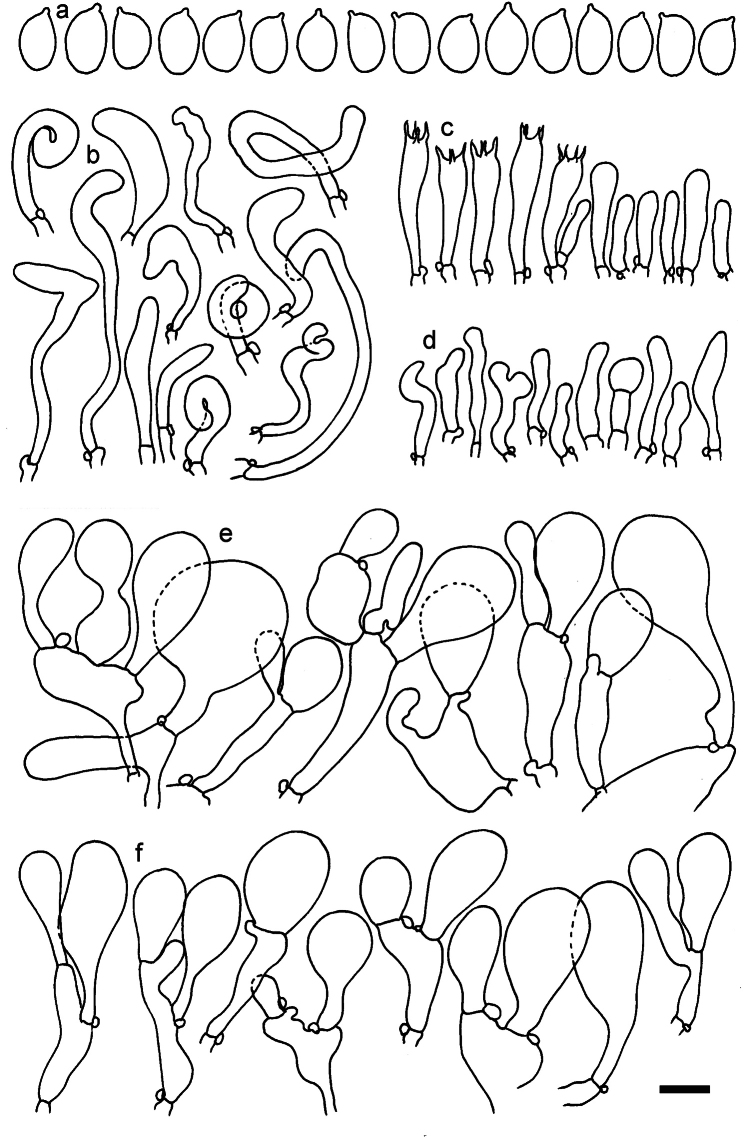
*Dermolomaangustisporum* (SAV F-20343, holotype), microscopic elements. **a** Spores; **b** caulocystidia; **c** basidia and basidioles; **d** marginal cells; **e** pileipellis elements near the pileus margin; **f** pileipellis elements near the pileus center. Scale bar: 5 µm for spores and 10 µm for other elements.

***Spores*** (5.1–)5.4–5.8–6.2(–6.8) × (2.9–)3.2–3.4–3.6(–3.9) μm; narrowly ellipsoid, oblong or narrowly amygdaloid, Q = (1.49–)1.60–1.70–1.80(–1.91); walls amyloid; hilar appendage 0.5–1 μm long. ***Basidia*** (22–)25–28.1–31(–34) × (5–)5.5–6.2–6.5(–7.5) μm; clavate; with 4 sterigmata. ***Basidioles*** first cylindrical, then clavate, ca. 3–6 μm wide. ***Marginal cells*** (10–)12.5–20.4–28(–40) × (3–)4–5.1–6.5(–7.5) μm; cylindrical or clavate, apically mainly obtuse or occasionally constricted, flexuous, occasionally nodulose lobate. ***Pileipellis*** 55–70 μm deep; suprapellis 35–45 μm deep, usually of one, rarely two layers of inflated, densely arranged, with some irregularly protruded terminal cells at surface; subpellis well differentiated, 20–30 μm deep, of densely packed, mainly horizontally oriented, 3–15 μm wide hyphae, gradually passing to horizontally oriented and intricate hyphae in trama; hyphal terminations with brownish parietal pigments, in subpellis darker yellow-brown pigments, walls of terminal cells thickened up to 1 μm, in subpellis up to 1.5 μm. Terminal cells near pileus margin (18–)28–34.9–42(–50) × (10–)13–15.7–18.5(–22) μm; usually obpyriform, clavate, occasionally hourglass-shaped or subcylindrical, occasionally lobate near septa; subterminal cells mainly branched, fusiform or clavate, rarely cylindrical, often lobate, with lateral swellings or projections. Terminal cells near pileus center (18–)26.5–35.8–45(–65) × (5–)10–15.1–20.5(–30) μm; clavate or obpyriform, less frequently lobate; subterminal cells narrower or equally wide, cylindrical, clavate or fusiform, occasionally branched, flexuous, occasionally with lateral swellings or nodulose. ***Caulocystidia*** (21–)34.5–53.5–72.5(–93) × (3–)3.5–5.1–6.5(–14) μm; clavate or subcylindrical, usually flexuous or twisted, often nodulose or lobate, repent and in small or larger clusters, often intermingled; usually with slightly thickened walls up to 0.5 μm, with yellowish parietal pigments. ***Clamp connections*** present.

###### Distribution and ecology.

Known from two localities; one in Germany and the type locality in Sweden (two collections); in semi-natural grassland, perhaps preferring neutral to alkaline, loamy soil, but insufficiently known.

###### Additional material studied.

Germany • Zittau, Kaiserfelder, coord. 50°52'26"N, 14°47'35"E, 16 Oct 2019, extensive sheep-grazed pasture, with loamy soil, A. Karich and R. Ullrich *IHI-19Der01* (GLM-F137761, as D.josserandiivar.phaeopodium). Sweden • Medelpad, Börgsjö, Talja Gården, terrestrial on meadow, 27 Aug 2018, S. Adamčík (SAV F-20342).

###### Notes.

*Dermolomaangustisporum* is a member of D.subgenusAmylospora, section Atrobrunnea. The narrow spores (Q = 1.6–1.8) make it morphologically distinct from other species with similar small mycenoid basidiomata. In the phylogeny it forms a distinct clade with *D.applanatum* (Fig. 2), a species with similar spores but with much larger collybioid basidiomata (see below).

##### ﻿ Dermoloma appalachianum

Taxon classificationAnimaliaAgaricalesTricholomataceae

Adamčík & Matheny sp. nov.

4C6E92B2-B163-5ADF-93D1-A7722BBBBE3A

856330

Figs 10b, c , 12

###### Etymology.

Type collected in the Appalachian Mts. of Tennessee, USA.

###### Holotype.

USA • Tennessee, Sevier County, Gatlinburg, on acidic soil in mixed forest including *Quercus*, *Carpinus*, Tsuga, 10 Jun 2010, J. M. Birkebak *JMB10061007* (TENN-F-065390).

###### Diagnosis.

North American species with small basidiomata and amyloid spores on average longer than 7 μm.

***Pileus*** 7–13 mm; convex, soon expanding to plane to slightly centrally depressed, indistinctly umbonate; margin even or striate up to 2 mm; surface slightly to strongly radially wrinkled, hygrophanous; color near margin brownish gray (5C2) to brownish ochraceous (5C3), near center dark brown (6F6), when dry fading to brownish gray (6C2) or grayish brown (6D3) at the margin and grayish brown (6E3) at the center. ***Stipe*** 17–22 × 2 mm; cylindrical, equal or tapering toward the base, at times curved; surface near lamellae slightly fibrillose-granulose, towards the base finely longitudinally striate-fibrillose; color near lamellae almost white, near the base brownish ochraceous (5C3) to brownish gray. ***Lamellae*** L = 22–28, l = 1–3; up to 2.5 mm wide; adnate to uncinate, slightly intervenose; color white to yellowish gray (4B2); edges even. ***Context*** when young elastic, later fragile; odor farinaceous.

***Spores*** (6.1–)6.7–7.4–8.2(–9.3) × (3.6–)4.1–4.5–4.9(–5.2) μm; narrowly ellipsoid to oblong, sometimes slightly amygdaloid, Q = (1.43–)1.53–1.64–1.75(–1.94); walls amyloid; hilar appendage 0.5–1 μm long. ***Basidia*** (25–)26–27.9–29.5(–31) × (7–)7.5–7.9–8.5(–9) μm; clavate; mainly with 4 sterigmata, rarely 1–2 sterigmata. ***Basidioles*** first cylindrical, then clavate, ca. 3.5–7 μm wide. ***Marginal cells*** (11–)14.5–19.7–24.5(–28) × (5–)6–7.3–8.5(–10) μm; clavate or capitate, often pedunculate, occasionally lobate or diverticulate, apically obtuse, subterminal cells often with lateral branches or swellings. ***Pileipellis*** 70–85 μm deep; suprapellis 40–50 μm deep, usually of one or two layers of inflated, densely arranged cells; subpellis 34–38 μm deep, of densely packed, irregularly oriented, 4–8(–15) μm wide hyphae, not sharply delimited from horizontally oriented hyphae in trama; hyphal terminations with brownish yellow parietal pigments, in subpellis darker yellow brown and near center also incrusted pigments, walls thickened up to 0.5 μm, near septa of terminal cells and in subpellis up to 1 μm. Terminal cells near pileus margin (25–)28.5–37.2–45.5(–66) × (11–)14–16.8–19.5(–25) μm; usually clavate, sphaeropedunculate or obpyriform, sometimes with narrowed, flexuous or lobate basal part; subterminal cells mainly branched, usually not inflated, fusiform, flexuous, often with branches or nodulose, occasionally inflated and with lateral swellings. Terminal cells near pileus center (18–)22.5–33.3–44(–57) × (8.5–)10–15.6–21(–28) μm; clavate, obpyriform, sphaeropedunculate, subglobose or ellipsoid, near basal parts often flexuous, lobate or with lateral swellings; subterminal cells similar to cells near margin. ***Caulocystidia*** (27–)35.5–49–62(–72) × 3.5–4.8–6(–8.5) μm; mainly narrowly clavate, flexuous, often twisted, apically mainly obtuse but often also constricted, rarely lobate, completely covering surface of stipe but loosely arranged, often intricate, repent or with ascending apices; thin-walled, with brownish yellow parietal pigments, occasionally partially with loose yellowish incrustations. ***Clamp connections*** present.

**Figure 12.**
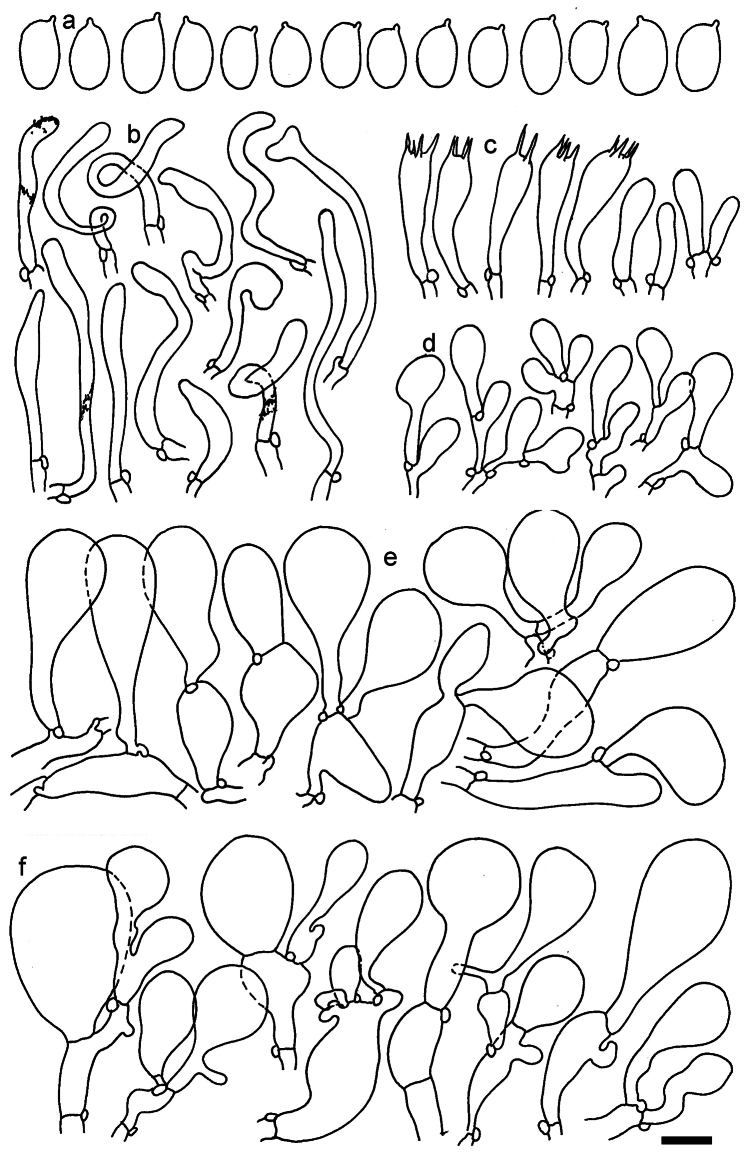
*Dermolomaappalachianum* (TENN-F-065390, holotype), microscopic elements. **a** Spores; **b** caulocystidia; **c** basidia and basidioles; **d** marginal cells; **e** pileipellis elements near the pileus margin; **f** pileipellis elements near the pileus center. Scale bar: 5 µm for spores and 10 µm for other elements.

###### Distribution and ecology.

Known from two localities in the Great Smoky Mountains, Tennessee, USA; in temperate, mixed deciduous forest.

###### Additional material studied.

USA • Tennessee, Cocke Co., Great Smoky Mountains National Park, Cosby Campground, elev. 625 m, coord. 35°45'22"N, 83°12'27"E, on acidic soil near *Acer* sp., *Carpinuscaroliniana*, *Carya* sp., *Liriodendrontulipifera*, *Magnoliaacuminata*, *M.tripetala*, *Oxydendronarboreum*, Quercussect.Lobatae, *Rhododendron* sp., *Tiliaamericana* and *Tsugacanadensis*, 29 Aug 2021, J. Kalichman and D. Grootmyers *DG21082913* (TENN-F-076604).

###### Notes.

*Dermolomaappalachianum* is a member of D.subgenusAmylospora, section Atrobrunnea. The lengthy spores (on average longer than 7 μm), in combination with the small mycenoid basidiomata, clearly distinguish this North American species from other similar European species. It is member of a distinct clade which includes *D.josserandii* and other taxa with relatively sturdy collybioid and pale colored basidiomata (Fig. 2). The North American species *D.vellingae* and *D.hymenocephalum* are also members of this clade but they both differ by shorter spores. This species was included in the phylogenetic study by Sánchez-García et al. (2021) as “*Dermoloma* sp. 10”. The collection TENN-F-065390 is selected as holotype because it contains multiple basidiomata but unfortunately it was not photographed.

##### ﻿ Dermoloma applanatum

Taxon classificationAnimaliaAgaricalesTricholomataceae

Corriol & P.-A. Moreau sp. nov.

0B976048-DE0E-5FC4-BAF6-4FEA101609B2

856331

Figs 10d, e , 13

###### Etymology.

The epithet *applanatum* refers to the characteristic applanate shape of the pileus.

###### Holotype.

United Kingdom • Wales, Pembrokeshire, Upton Castle, coord. 51°42'22"N, 04°51'57"E, terrestrial in lawn, 26 Oct 2014, S. Adamčík (SAV F-4415).

###### Diagnosis.

European species; basidiomata moderately large; pilei soon expanding to plane; stipes 2–5.5 mm wide, fusiform and dark brown near bases; spores amyloid, ellipsoid to oblong; caulocystidia 3.5–5.5 μm wide.

***Pileus*** 10–30(–40) mm; convex, soon expanding to plane, sometimes lobate; margin translucently striate to half of the radius or not striate, recurved when old; surface smooth near margin, rugulose and sometimes pitted near center, hygrophanous; color near margin dark brown (6F4, 6F5), brown (5E4, 6E4), grayish brown (5E3, 6D3, 6E3), when dry brownish ochraceous (5C3) or grayish brown (5D4, 6D4), near center dark brown (6F3, 6F4, 6F5) or brown (6E5), when dry light brown (5D4, 5D5, 6D4). ***Stipe*** (20–)22–37(–40) × (1.5–)2–5.5(–8) mm; fusiform or cylindrical, narrowed towards the base, flexuous; surface finely longitudinally striate, near lamellae pruinose or granulose, towards the base finely fibrillose or squamulose; color near lamellae when young pale gray (B1, C1), ochraceous-gray (5B2), when mature brownish gray (5C2, 5C2, 6C2, 6D2) or grayish brown (5D3, 6D3), near the base brownish gray (6C2, 6D2), grayish brown (6D3, 6E3), brown (6E4, 7F3, 7F4) to almost black (7F8). ***Lamellae*** L = 20–39(–48), l = (0–)1–3; 4–8 mm wide; adnate-emarginate and decurrent with tooth; color ochraceous-gray (6B2), brownish gray (5D2, 6C2, 6D2), grayish brown (7D3), towards edges paler ochraceous-gray (5B2), brownish gray (5C2); edges entire. ***Context*** when young compact, later fragile; odor farinaceous.

**Figure 13.**
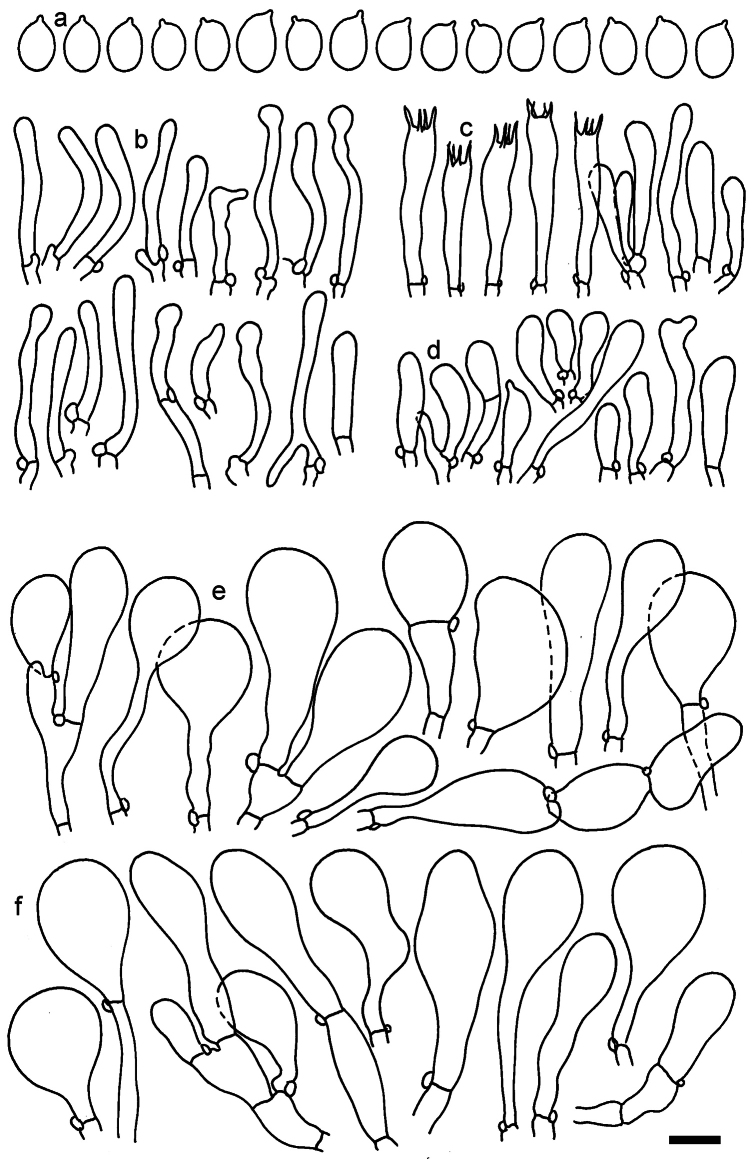
*Dermolomaapplanatum* (SAV F-4415, holotype), microscopic elements. **a** Spores; **b** caulocystidia; **c** basidia and basidioles; **d** marginal cells; **e** pileipellis elements near the pileus margin; **f** pileipellis elements near the pileus center. Scale bar: 5 µm for spores and 10 µm for other elements.

***Spores*** (3.6–)5.2–6–6.9(–8.4) × (3.1–)3.5–3.7–4(–4.3) μm; ellipsoid to oblong, Q = (1.26–)1.44–1.63–1.81(–2.03); amyloid; hilar appendage 0.5–1.5 μm long. ***Basidia*** (19.5–)23.5–28.3–33(–42) × (5–)5.5–6.2–7(–8) μm; clavate; with 4 sterigmata. ***Basidioles*** first cylindrical, then clavate, ca. 3–6 μm wide. ***Marginal cells*** (11–)16.5–23.1–29.5(–47) × (3–)6–8–10(–12) μm; clavate, sometimes apically constricted, rarely lobate. ***Pileipellis*** 70–85 μm deep; suprapellis mainly of two, occasionally one or three layers of inflated cells; subpellis 28–40 μm deep, well defined, of densely packed, irregularly oriented, intricate, 3­9(–15) μm wide hyphae, not sharply delimited from horizontally oriented hyphae in trama; hyphal terminations with brownish parietal pigments, hyphae in subpellis thickened up to 1 μm and locally with dark incrusted pigments. Terminal cells near pileus margin (18.5–)29.5–40.6–51.7(–80) × (8–)13–20–27(–43) μm; usually clavate, sphaeropedunculate or obpyriform, sometimes ellipsoid, towards septa occasionally flexuous or constricted; subterminal cells usually narrower, cylindrical or similar to terminal cells and clavate or obpyriform, occasionally with lateral swellings, usually not branched and not lobate. Terminal cells near pileus center (12.5–)26.5–37.6–49(–77) × (6–)11.5–18–24.5(–39) μm; similar to cells near margin; subterminal cells mainly narrower and cylindrical or clavate, occasionally with lateral swellings, not lobate, occasionally branched. ***Caulocystidia*** (19–)26.5–34.8–43(–57) × (2.5–)3.5–4.4–5.5(–7.5) μm; clavate, sometimes cylindrical or capitate, slightly flexuous, rarely apically constricted, often clustered in small ascending fascicules, sometimes individual and repent; usually with slightly thickened walls up to 0.5 μm, with yellowish parietal pigments. ***Clamp connections*** present.

###### Distribution and ecology.

Known from France, Germany and Wales (United Kingdom); in semi-natural grassland and (rarely) forests, on neutral to calcareous loamy soil.

###### Additional material studied.

France • Hautes-Pyrénées, Castet de Gerde, coord. 43°03'35"N, 00°09'54"E, mown meadow on neutral clay soil, 14 Nov 2010, G. Corriol *GC10111408* (BBF); • Pas-de-Calais, Baincthun, forêt domaniale de Boulogne-sur-Mer, maison forestière de Wirwignes, coord. 50°40'55"N, 01°44'26"E, on clay-calcareous ground under *Carpinusbetulus* and *Quercuspetraea*, 14 Sep 2014, E. Bastien *PAM14091410* (LIP). Germany • Baden-Württemberg, Justingen, Schachenheide, coord. 48°24'35"N, 09°40'25"E, terrestrial in grassland, 2 Oct 2021, S. Adamčík (SAV F-20860); • Rheinland-Pfalz, Sobernheim, elev. 215 m, coord. 49°47'18"N, 07°40'35"E, terrestrial in semi-natural grassland, 9 Nov 2019, C. Manz (SAV F-20525). United Kingdom • Wales, Pembrokeshire, Upton Castle, coord. 51°42'22"N, 04°51'57"E, terrestrial in lawn, 26 Oct 2014, D. Harries (SAV F-4419); • Wales, Powis Castle gardens, coord. 52°38'58"N, 03°09'34"E, terrestrial in lawn, 22 Oct 2014, D. Harries (SAV F-4379).

###### Notes.

*Dermolomaapplanatum* is a member of D.subgenusAmylospora, section Atrobrunnea (Fig. 2). In the field it is relatively well defined by a collybioid habit, typical plane pilei when mature and relatively dark colors. Similar species with collyboid basidiomata have caulocystidia wider than 5 μm. The most closely related *D.angustisporum* has much smaller mycenoid basidiomata (see above). This species was previously included in the phylogenetic study by Sánchez-García et al. (2021) as “*D.phaeopodium* like”. The majority of our collections were associated with multiple *Dermoloma* species, but not always on sites with an apparent conservation concern (collections from Wales in castle gardens).

##### ﻿ Dermoloma atrocinereum

Taxon classificationAnimaliaAgaricalesTricholomataceae

(Pers.) P.D. Orton, Trans. Br. mycol. Soc. 43(2): 175. 1960.

D812D840-BE1F-5EE9-9F94-FEE8AA144C00

329820

Figs 10f, g , 14

Dermolomalongibasidiatum Contu, Consiglio & Setti, Micol. Veg. Medit. 22(2): 110. 2008; Dermolomapragensef.obscurum Consiglio & Contu, in Contu, Consiglio & Setti, Micol. Veg. Medit. 22(2): 99. 2008. Syn.

###### Neotype.

(designated by Contu et al. 2008): Italy • Province of L’Aquila, Abruzzo, Villeta Barrea, under *Quercuscerris* and *Fagussylvatica* on basic soil, 19 Sep 2003, C. Baratti (AQUI).

###### Distinguishing characters.

European species; basidiomata large, tricholomatoid; stipes usually wider than 6 mm; spores inamyloid, on average 5.2–6.2 × 3.9–4.5 μm; basidia relatively long (27–33 μm).

***Pileus*** (15–)20–45(–50) mm; convex or broadly conical, sometimes indistinctly umbonate, when mature expanding to plane and sometimes with reflexed margin, often lobate; margin not striate; surface rugulose, radially veined and wrinkled, often pitted, towards margin usually smooth whitish pruinose, not hygrophanous; color near margin dark brown (6F4, 6F5, 6F6, 6F8, 7F4, 8F4), with age usually becoming brown (6E3, 6E4, 7E3), grayish brown (6D2, 6D3) to brownish gray (5C3, 6C3), near center usually darker, dark brown (6F3, 6F4, 6F5, 6F7, 7F3, 7F4, 8F3, 8F4) to black, rarely brown (6E5, 7E4). ***Stipe*** (20–)27–53(–63) × (3–)4.5–12(–15) mm; fusiform or cylindrical, usually narrowed towards the base, flexuous; surface sometimes finely longitudinally striate, near lamellae pruinose or granulose, towards the base finely to distinctly fibrillose or squamulose; color ochraceous-gray (5B2, 6B2), near lamellae usually paler to almost white, towards the base often with darker brownish gray (5C2, 6C2), grayish brown (5D3, 6D3, 6E3), light brown (6D5), brown (6E5) or dark brown (6F4) fibrils or squamules. ***Lamellae*** L = (26–)33–53, l = (0–)1–3(–7); 4–10 mm wide; adnexed or adnate-emarginate and decurrent with tooth; color ochraceous-gray (5B2, 6B2), brownish ochraceous (5C3, 6C3), brownish gray (6C2) paler towards edge, sometimes darker grayish brown (6D3, 6E3) to brown (6E4) near pileus context; edges entire or slightly irregular. ***Context*** when young compact, later fragile; odor farinaceous.

**Figure 14.**
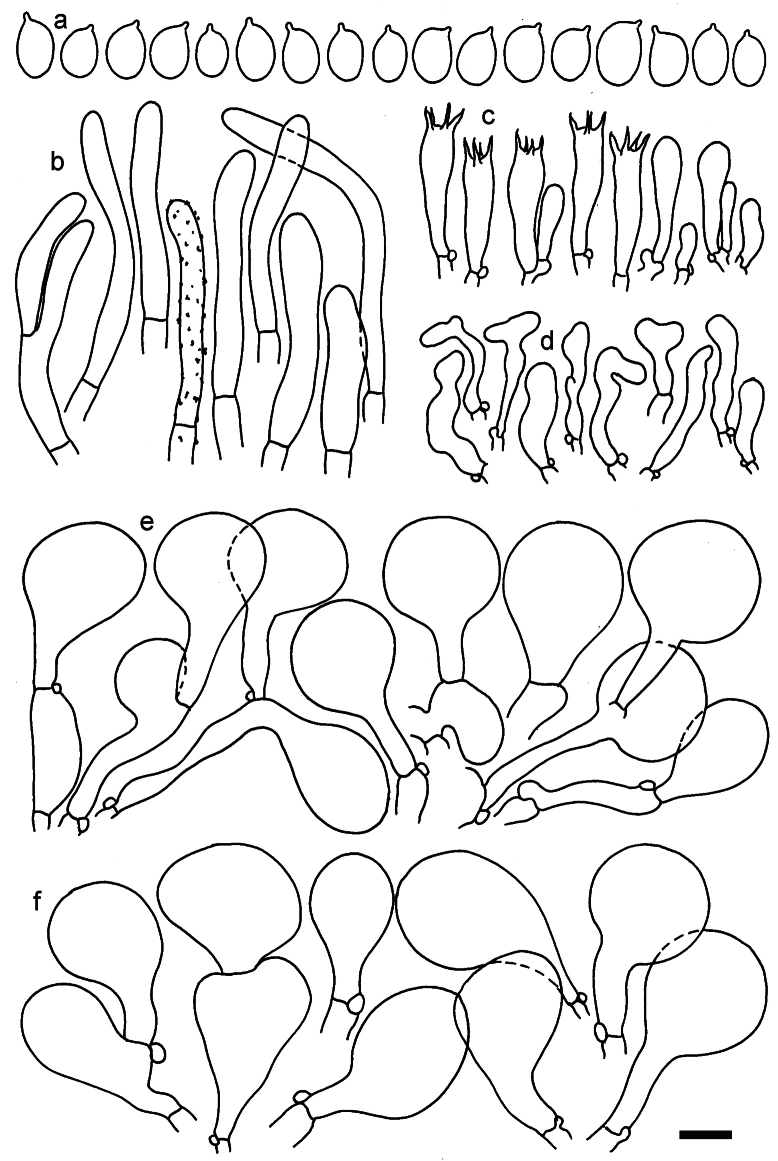
*Dermolomaatrocinereum* (AQUI 19.IX.2003, holotype), microscopic elements. **a** Spores; **b** caulocystidia; **c** basidia and basidioles; **d** marginal cells; **e** pileipellis elements near the pileus margin; **f** pileipellis elements near the pileus center. Scale bar: 5 µm for spores and 10 µm for other elements.

***Spores*** (4.7–)5.2–5.7–6.2(–7.3) × (3.4–)3.9–4.2–4.5(–5) μm; broadly ellipsoid to ellipsoid, Q = (1.20–)1.28–1.37–1.45(–1.59); walls inamyloid, rarely thick-walled and dextrinoid; hilar appendage ca. 1–1.5 μm long. ***Basidia*** (24–)27.1–29.9–32.8(–40) × (5.5–)6–6.9–7.5(–8) μm; clavate; with 4 sterigmata. ***Basidioles*** first cylindrical, then clavate, ca. 2.5–7 μm wide. ***Marginal cells*** (15–)17.5–25.7–34(–51) × (3.5–)5–5.7–6.5(–8) μm; clavate, flexuous, often moniliform, often apically lobate. ***Pileipellis*** 79–102 μm deep; suprapellis of one or two, rarely three layers of inflated cells, gradually passing to 30–45 μm deep subpellis of densely packed, irregularly oriented, puzzled, 3.5­–12 μm wide hyphae, relatively well delimited from horizontally oriented hyphae in trama; hyphal terminations with brown parietal pigments, thin-walled but in subpellis with thickened up to 1.3 μm walls, and with thick dark brown to black incrusted pigments especially near septa of terminal cells. Terminal cells near pileus margin (17–)29.5–41.1–52.5(–75) × (9–)16.5–22.1–27.5(–44) μm; usually obpyriform or sphaeropedunculate, sometimes ellipsoid, subglobose or clavate; subterminal cells narrower or equally wide, usually unbranched, clavate, ventricose, obpyriform or cylindrical, often with lateral swellings. Terminal cells near pileus center very variable in size, (10–)24.6–36.1–47.5(–67) × (6.5–)11.5–18.9–26(–47) μm; obpyriform, clavate, ellipsoid or subglobose; subterminal cells usually narrower and unbranched, clavate or obpyriform, sometimes equally wide and with lateral swellings, rarely irregularly lobate. ***Caulocystidia*** (13–)24–37.3–50.5(–75) × (3–)4–6.3–8.5(–12.5) μm; cylindrical or clavate, usually not or only slightly flexuous, clustered in repent fascicules; usually with slightly thickened walls up to 0.5 μm, often with granulose yellow incrustations. ***Clamp connections*** present.

###### Distribution and ecology.

Widely distributed and rather frequent in Europe; in semi-natural grasslands, deciduous and coniferous forests, on calcareous soil, occurring mainly in temperate regions, more rarely Mediterranean, documented north to a hemi-boreal site in the Oslofjord area of SE Norway.

###### Additional material studied.

Austria • Steiermark, Sausal, Plesch, Fastlkogel, elev. 383 m, coord. 46°47'07"N, 15°28'52"E, 14 Nov 2020, G. Friebes *GF20200262* (SAV F-23432). Croatia • Mljet island, Mljet National Park, 3 km E/E-SE of Goveđari village, coord. 42°46'52"N, 17°22'41"E, semi-natural grassland with shrubs of *Phillyrea* sp., *Pistacia* sp., *Viburnumtinus*, 9 Dec 2018, Z. Tkalčec (CNF 1/7750). France • Aveyron, Le Fel, Le Mas, coord. 44°39'27"N, 02°30'55"E, old grassland, 15 Nov 2016, C. Hannoire *CH16111508* (BBF, as *D.cuneifolium*); • Hautes-Pyrénées, Castet de Gerde, coord. 43°03'35"N, 00°09'54"E, *Brachypodiumrupestre* calcicolous fringe, 14 Nov 2012, G. Corriol *GC12111407* (BBF, as *D.cuneifolium*); • Hautes-Pyrénées, Sariac-Magnoac, coord. 43°18'43"N, 00°33'14"E, marly grazed pasture (*Mesobromion*), 23 Nov 2016, G. Corriol *GC16112316* (BBF); ibid., 23 Nov 2016, G. Corriol *GC16112315* (BBF); • Pas-de-Calais, Noeux-les-Auxi, Réserve Naturelle Régionale des Riez du Mont de Boffles, coord. 50°14'47"N, 02°11'55"E, grazed calcareous meadow, 15 Oct 2004, C. Lécuru *CL/F04.168* (LIP, as *D.cuneifolium*); • ibid., 6 Nov 2004, R. Courtecuisse *RC/F04.090* (LIP, as *D.cuneifolium*); • Pas-de-Calais, Neufchâtel-Hardelot, coord. 50°36'39"N, 01°36'35"E, réserve naturelle du Mont-Saint-Frieux, calcareous grassland, 9 Nov 2014, P.-A. Moreau *PAM14110906* (LIP, as D.cf.cuneifolium); • ibid., 9 Nov 2014, P.-A. Moreau *PAM14110908* (LIP, as D.cf.cuneifolium); • Pas-de-Calais, Wavrans-sur-l’Aa, Réserve naturelle nationale des Coteaux de Wavrans, coord. 50°41'16"N, 02°08'23"E, dry calcareous grassland, 9 Nov 2016, D. Huart and P.-A. Moreau *PAM16110902* (LIP, as D.cuneifoliumvar.punctipes); • Savoie, Billième, forêt de Lierre, coord. 49°19'57"N, 02°36'08"E, calcareous ground under *Buxus* with *Quercuspubescens*, 23 Oct 2000, M. Durand and P.-A. Moreau *PAM00102305* (LIP, as *D.atrocinereum*). Germany • Baden-Württemberg, Justingen, Schachenheide, coord. 48°24'35"N, 09°40'25"E, terrestrial in semi-natural grassland, 2 Oct 2021, S. Adamčík (SAV F-20861); • ibid., 2 Oct 2021, S. Adamčík (SAV F-20866); • ibid., 2 Oct 2021, S. Adamčík (SAV F-20877); • ibid., 2 Oct 2021, M. Caboň (SAV F-20883); • ibid., 3 Oct 2021, M. Caboň (SAV F-20896); Kalkbruch, Schöpsatal, coord. 51°13'00"N, 14°56'55"E, 5 Oct 2022, under deciduos trees (*Acer* sp., *Populustremula*, *Betulapendula*) on limestone, A. Karich and R. Ullrich *IHI-22Der04* (SAV F-23435); • Rheinland-Pfalz, Brauweiler, Wingertsberg, elev. 250 m, coord. 49°49'18"N, 07°29'16"E, terrestrial in seimi-natural grassland, 9 Nov 2019, H. Terlutte (SAV F-20565); • Rheinland-Pfalz, Heimberg, elev. 265 m, coord. 49°48'37"N, 07°44'06"E, terrestrial in semi-natural grassland, 10 Nov 2019, S. Adamčík (SAV F-20551); • ibid., 10 Nov 2019, S. Adamčík (SAV F-20552); Rheinland-Pfalz, Sobernheim, elev. 215 m, coord. 49°47'18"N, 07°40'35"E, terrestrial on semi-natural grassland, 9 Nov 2019, F. Hampe (SAV F-20523); • ibid., 9 Nov 2019, S. Adamčík (SAV F-20528). Hungary • Fejér, Magyaralmás, coord. 47°19'27"N, 18°18'42"E, in *Pinusnigra* plantation on dolomite bedrock, 8 Nov 2020, A. Koszka *FP-2020-11-08-1* (ELTE). Italy • Marche, Monte Grino, Piobbico (PU), grassy area close to *Cedrusatlantica* trees, 22 Oct 2006, G. Consiglio and M. Maletti *GC06186* (AMB, holotype of D.pragensef.obscurum); • Sardinia, Tempio Pausania, in a grassy area, 29 Dec 2003, M. Contu *AV 14562003* (TO, as *D.fuscobrunneum*); • Susà, Pegrine (TN), among grass, 30 Nov 1993, G. Consiglio, G. Marasca and B. Oss-Emer *GC93318* (AMB, holotype of *D.longibasidiatum*). Norway • Akershus, Asker, Elnestangen SW, coord. 59°47'58"N, 10°29'38"E, margin of *Tilia* forest on calcareous soil, 2 Oct 2015, T. E. Brandrud and B. Dima *DB5880* (ELTE), duplicate *TEB644-15* (O). Slovakia • Kremnické vrchy Mts., pasture 0.5 km W of Tajov, elev. 600 m, coord. 48°44'54"N, 19°03'31"E, terrestrial on pasture, 25 Oct 2020, M. Caboň (SAV F-20844); • Laborecká vrchovina Mts., 1.5 km NNE of Svetlice, elev. 390 m, coord. 49°11'03"N, 22°02'38"E, terrestrial on pasture, 21 Sep 2006, S. Adamčík (SAV F-4150); • ibid., 23 Oct 2007, S. Adamčík (SAV F-4138); • Levočské vrchy Mts., 1.5 km NEE of Bijacovce, elev. 650–700 m, coord. 49°01'49"N, 20°49'12"E, terrestrial under *Larixdecidua* and *Piceaabies*, 25 Sep 2004, S. Adamčík (SAV F-4148). United Kingdom • Wales, Pembrokeshire, Upton Castle, coord. 51°42'22"N, 04°51'57"E, terrestrial in lawn, 26 Oct 2014, D. Harries (SAV F-4417).

###### Notes.

*Dermolomaatrocinereum* is a member of D.subgenusDermoloma, section Dermoloma. It has the largest and sturdiest basidiomata within the subgenus with typically broadly conical and obtusely umbonate pilei features conducive to field identification. Other similar species have shorter basidia. The conical pilei are reminiscent of the field appearance of *D.bellerianum*, but the spores of this species are narrower (Q > 1.6). Members of section Dermoloma show relatively short genetic distances with low support at higher rank nodes in the tree (Fig. 2), which do not allow to specify further relationships for *D.atrocinereum*. The taxonomic concept of this species was recognized by Sánchez-García et al. (2021) by position of the neotype sequence in the phylogeny. *Dermolomaatrocinereum* was originally described from a pine forest in Germany. We show that it has a broader ecology, including deciduous forests and grasslands. It is one of the most common species in the genus.

##### ﻿ Dermoloma bellerianum

Taxon classificationAnimaliaAgaricalesTricholomataceae

Bon, Doc. Mycol. 28(109–110): 6. 1998.

E51177A8-9D29-57E2-804C-49CED158F4CD

444845

Figs 15a, b , 16

###### Holotype.

France • Pyrénées-Orientales, Ferrières, col de Spandelles, under *Buxus* (pastures?), 30 Sep 1972, Jean Beller *Beller 912* (LIP – only notes in M. Bon’s collection, material not located).

###### Epitype

(designated here MBT10022906): United Kingdom • Wales, Pembrokeshire, Upton Castle, coord. 51°42'22"N, 04°51'57"E, terrestrial in lawn, 26 Oct 2014, D. Harries (SAV F-4416).

###### Distinguishing characters.

European species; basidiomata moderately large to large; stipes and lamellae pale white; spores narrow (Q > 1.7), 2.9–3.4 μm wide.

***Pileus*** 13–31(–60) mm; convex to broadly conical; margin not striate, sometimes lobate; surface strongly radially rugulose and veined; color when young grayish brown (8F3), when mature near margin brownish gray (6C2) or ochraceous-gray (5B2), near center dark brown (6F3) to black, sometimes brownish ochraceous (6C3). ***Stipe*** 27–48(–70) × 3.5–8(–10) mm; cylindrical, narrowed towards the base, slightly flexuous; surface finely longitudinally striate, near lamellae pruinose, towards the base finely fibrillose to almost smooth, sometimes with fine darker gray squamules; color white to gray (B1) or brownish gray (6D2). ***Lamellae*** L = 38–50(–55), l = (0–)1–3(–7); 2.5–5 mm wide; adnexed or adnate-emarginate, sometimes decurrent with tooth; color almost white to yellowish white (4A2), sometimes gray (B1) to brownish gray (6C2); edges entire or serrulate, early eroded. ***Context*** first compact and elastic, later fragile in pileus; odor strongly farinaceous.

**Figure 15.**
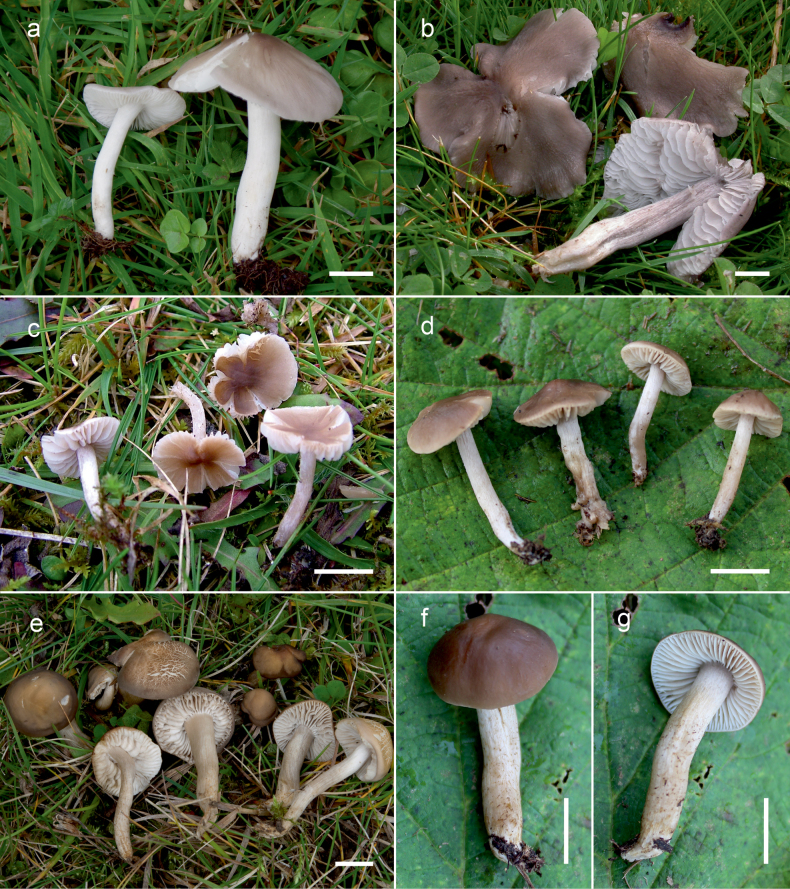
Basidiomata of *Dermoloma* in field appearance. **a***Dermolomabellerianum* (SAV F-4416, epitype), photo D. Harries; **b***Dermolomabellerianum* [LIP (*PAM05100303*)], photo P.-A. Moreau; **c***Dermolomacarpathicum* (SAV F-4268), photo S. Jančovičová; **d***Dermolomacarpathicum* (SAV F-20805), photo S. Jančovičová; **e***Dermolomacompactum* (SAV F-23421), holotype, photo G. Friebes; **f, g***Dermolomacompactum* (SAV F-20794), photo S. Jančovičová. Scale bar: 10 mm.

***Spores*** (4.3–)5.1–5.6–6.1(–6.9) × (2.5–)2.9–3.2–3.4(–3.9) μm; narrowly ellipsoid to oblong, Q = (1.42–)1.64–1.79–1.93(–2.3); walls inamyloid, sometimes thick-walled and dextrinoid, hilar appendage ca. 1–1.5 μm long. ***Basidia*** (17–)20–23–26(–31) × (4–)5–5.7–6.5(–7.5) μm; clavate; number of sterigmata variable, most often 2 or 4 sterigmata, or mixture of these, occasionally with 3 and 1 sterigmata. ***Basidioles*** first cylindrical, then clavate, ca. 2.5–5.5 μm wide. ***Marginal cells*** (10–)12–16–20(–23) × (2–)2.5–3.9–5(–6) μm; usually clavate, sometimes obpyriform or cylindrical, rarely fusiform, sometimes lobate. ***Pileipellis*** 45–55 μm deep; suprapellis of mainly one, occasionally two layers of inflated cells; subpellis 13–18 μm deep, hardly differentiated, with irregularly oriented, puzzled, 4–12(–15) μm wide hyphae, sharply delimited from horizontally oriented hyphae in trama; hyphal terminations with brownish parietal pigments and without incrusted pigments, in subpellis with thickened walls up to 1 μm. Terminal cells near pileus margin (25–)34.5–45–55(–76) × (11–)16–20.5–25(–32) μm; usually sphaeropedunculate, sometimes obpyriform or clavate, rarely lageniform, fusiform or clavate-lageniform; subterminal cells usually narrower and branched, clavate or obpyriform, often with lateral swellings. Terminal cells near pileus center (20–)36.5–50–63(–89) × (9.5–)1 5–21–26(–36) μm; usually sphaeropedunculate, sometimes clavate or obpyriform; subterminal cells narrower or equally wide, often with lateral swellings or irregularly lobate. ***Caulocystidia*** (19–)28–44.2–60(–118) × (2.5–)4.6–6.7–9(–12) μm; clavate or cylindrical, rarely fusiform, usually not or only slightly flexuous, often clustered in small ascending fascicules, sometimes individual and repent; usually with slightly thickened walls up to 0.5 μm, often with crystalline or granulose yellow incrustations. ***Clamp connections*** present.

**Figure 16.**
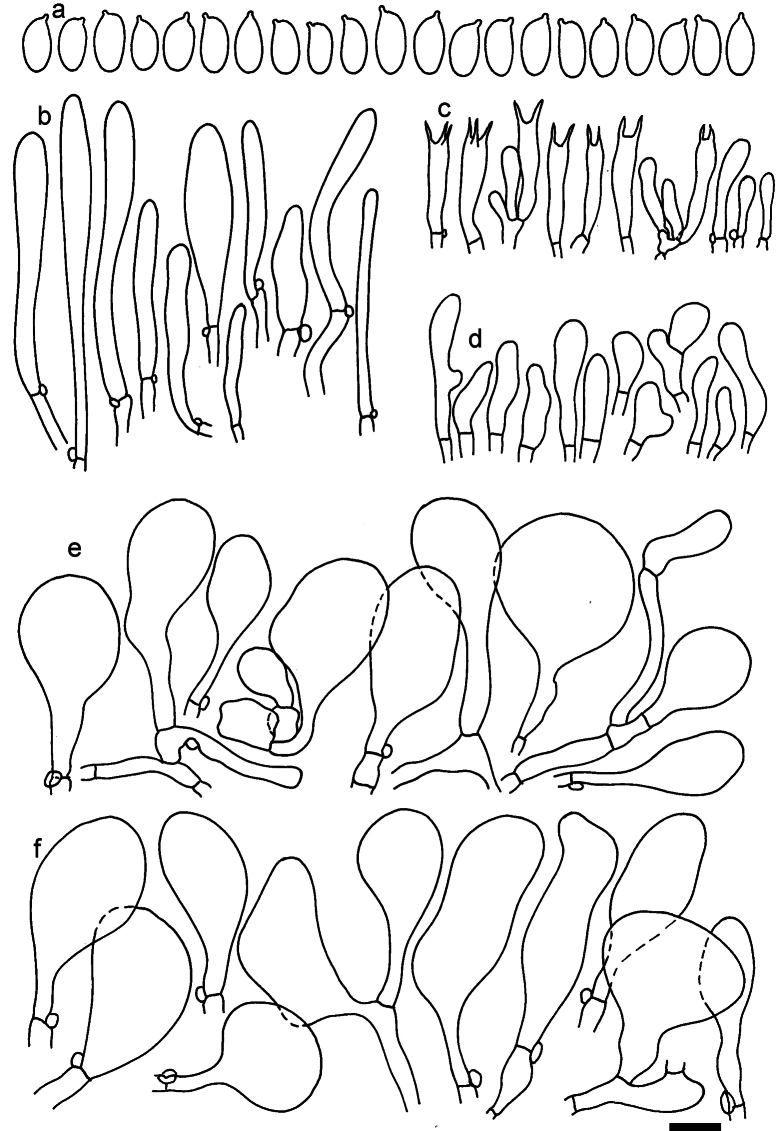
*Dermolomabellerianum* (SAV F-4416, epitype), microscopic elements. **a** Spores; **b** caulocystidia; **c** basidia and basidioles; **d** marginal cells; **e** pileipellis elements near the pileus margin; **f** pileipellis elements near the pileus center. Scale bar: 5 µm for spores and 10 µm for other elements.

###### Distribution and ecology.

Known from Croatia, France, Germany, Italy, Norway, Slovakia and Wales (United Kingdom); in semi-natural grasslands and deciduous forests on calcareous soil, mainly in temperate regions, so far sequence-verified north to the hemi-boreal SE Norway.

###### Additional material studied.

Croatia • Zagreb, Maksimir, coord. 45°50'11"N, 15°59'27"E, semi-natural grassland, 4 Nov 2003, M. Čerkez (CNF 1/3243). France • Hautes-Pyrénées, Castet de Gerde, coord. 43°03'35"N, 00°09'54"E, *Brachypodium* fringe, 8 Dec 2012, G. Corriol *GC12120807* (BBF, as *D.atrocinereum*); • Nord, Loos, parc de la Faculté de pharmacie de Lille, coord. 03°02'24"N, 50°36'16"E, calcareous grassland on rich soil, 3 Oct 2005, P.-A. Moreau *PAM05100303* (LIP); • Oise, Sacy-le-Grand, Marais de Sacy, sud de Saint Martin-Longueau, coord. 49°19'57"N, 02°36'08"E, 3 Oct 2013, P. Clowez *CL/F13.170* (LIP). Germany • Baden-Württemberg, Justingen, Schachenheide, coord. 48°24'35"N, 09°40'25"E, terrestrial in semi-natural grassland, 3 Oct 2021, M. Caboň (SAV F-20911). Italy • Emilia-Romagna, Poggio di Carviano (Grizzana Morandi BO), in an herbaceous clearing under *Cedrusatlantica*, 31 Oct 1993, G. Consiglio *GC93319* (AMB 15101, as *D.pragense*). Norway • Oppland, Gjøvik, Biri, Eriksrud NR, coord. 60°56'43"N, 10°38'20"E, in calcareous *Tilia* forest, 10 Sep 2019, T. E. Brandrud and B. Dima *TEB338b-19* (O); • Østfold, Moss, Jeløy, Refsnesskogen, coord. 59°26'51"N, 10°36'49"E, on rich soil with *Hygrocybe* spp., 6 Sep 2011, T. Læssøe *NOBAS2392-16* (O-F-21095). Slovakia • Biele Karpaty Mts., 1.5 km E of Nová Bošáca, Blažejová Natural Monument, elev. 415 m, coord. 48°52'33"N, 17°49'03"E, terrestrial on meadow, 27 Nov 2005, S. Jančovičová (SAV F-4143); • Poloniny Mts., 4 km N of Stakčín, pastures above the water reservoir Starina, elev. 380–420 m, coord. 49°02'43"N, 22°14'56"E, terrestrial, 25 Sep 2017, M. Caboň (SAV F-20227).

###### Notes.

*Dermolomabellerianum* is a member of D.subgenusDermoloma, section Conica. In the field the species is defined by pale colors especially on the lamellae and stipes, in addition to the typically conical pilei. It is morphologically well-defined by narrow spores (Q > 1.6). The only similar and closely related species is D.aff.bellerianum documented below based on a single collection. The concept of this species was based on the morphology matching the original description (Bon 1998). The original description only mentioned bisporic basidia but the number of sterigmata was variable among studied collections. The only remaining part of the original material of *D.bellerianum* (collection of Beller no. 912) is M. Bon’s drawings on his unpublished sheet. The material is lost, but we hesitated to select M. Bon’s drawings as the lectotype because Jean Beller’s herbarium might still be preserved. The collection SAV F-4416 was designated as an epitype, because it is geographically closest to the type collection area among collections with good sequence representation and documentation. The phylogenetic concept of the species presented here agrees with a previously published study by Sánchez-García et al. (2021).

##### ﻿ Dermoloma aff. bellerianum

Taxon classificationAnimaliaAgaricalesTricholomataceae

F002B386-DCEF-5725-9597-41B441157505

Fig. 17

###### Description.

***Pileus*** 15–45 mm; convex with more or less persistent umbo; margin not striate; surface densely radially wrinkled, not hygrophanous; color when young and fresh anthracite black, with age and when drying fading from margin, becoming pale lilac-gray with white margin. ***Stipe*** 40–70 × 3–8(–10) mm, cylindrical, narrowed towards the base; surface longitudinally twisted-striate, near lamellae minutely pruinose when young, then furfuraceous all along; color white, with gray fibrils. ***Lamellae*** very broad; adnate-emarginate; color white to grayish; edges entire. ***Context*** white or hyaline gray; odor strongly farinaceous.

**Figure 17.**
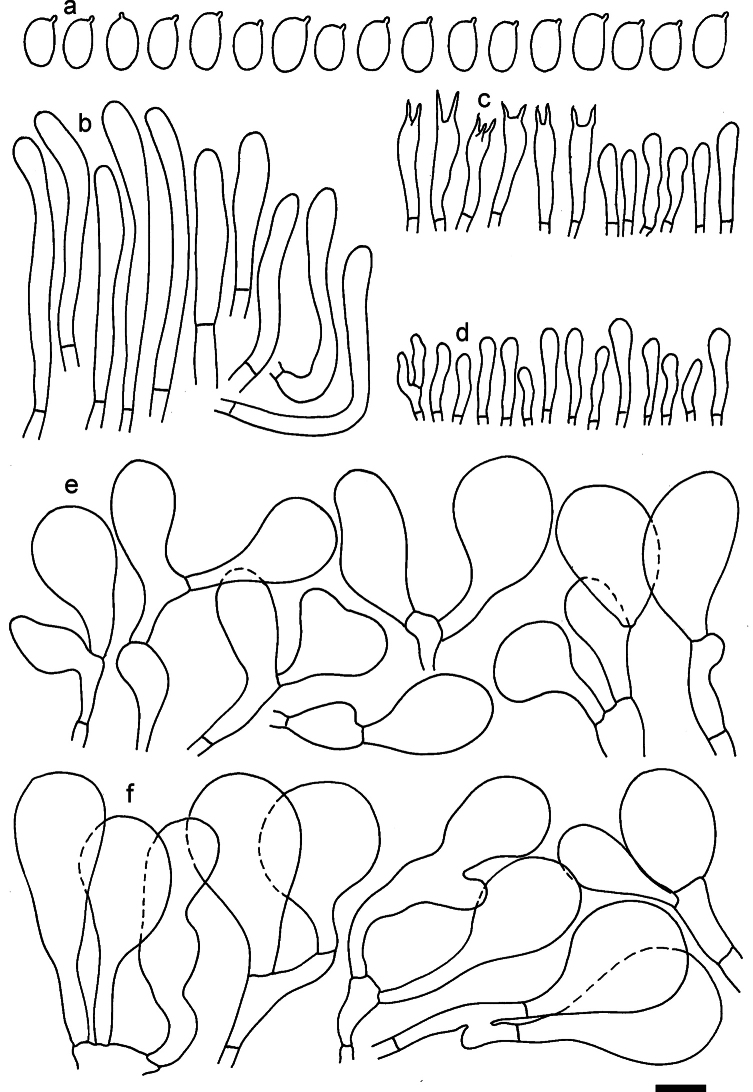
Dermolomaaff.bellerianum [LIP (*PAM14102921*)], microscopic elements. **a** Spores; **b** caulocystidia; **c** basidia and basidioles; **d** marginal cells; **e** pileipellis elements near the pileus margin; **f** pileipellis elements near the pileus center. Scale bar: 5 µm for spores and 10 µm for other elements.

***Spores*** (4.9–)5.1–5.5–5.8(–6.2) × (2.9–)3.1–3.3–3.4(–3.7) μm; narrowly ellipsoid to oblong, Q = (1.55–)1.60–1.67–1.74(–1.84); walls inamyloid, sometimes thick-walled and dextrinoid; hilar appendage ca. 0.5–1 μm long. ***Basidia*** (19–)20.5–23.3–26(–30) × (4.5–)5–5.3–5.5(–6) μm; clavate; with 2 sterigmata, occasionally with 1 sterigma. ***Basidioles*** first cylindrical, then clavate, ca. 3–5 μm wide. ***Marginal cells*** (11.5–)14–16.7–19.5(–21) × 2.5–3.4–4(–5) μm; not well-differentiated and similar to basidioles, clavate or cylindrical. ***Pileipellis*** 80–105 μm deep; suprapellis 55–70 μm deep, of one to three layers of densely arranged inflated cells; subpellis 15–35 μm deep, hardly differentiated, of almost horizontally oriented, puzzled, 4­–15(–25) μm wide hyphae, not sharply delimited from horizontally oriented hyphae in trama; hyphal terminations with brownish yellow parietal pigments, near septa of terminal cells and in subpellis often with darker brown and near center almost black incrusted pigments, terminal cells often with slightly thickened walls up to 0.5 μm, in subpellis thick-walled (walls up to 1 μm). Terminal cells near pileus margin (25–)34.5–46.1–58(–70) × (16–)19.5–24.1–29(–34) μm; obpyriform, clavate or sphaeropedunculate; subterminal cells branched or not, mainly inflated and with large lateral swellings, occasionally narrower fusiform or subcylindrical, often lobate or nodulose. Terminal cells near pileus center (27–)40.5–55.4–70(–95) × (13–)18–26.1–34.5(–49) μm; usually clavate or sphaeropedunculate, occasionally obpyriform or ellipsoid, occasionally lobate near septa; subterminal cells mainly branched, usually narrow, cylindrical or fusiform, implemented in intricate hyphae of subpellis. ***Caulocystidia*** (35–)41.5–51.8–62(–73) × (4.5–)5–6.1–7(–8.5) μm; clavate or cylindrical, usually not or only slightly flexuous, in dispersed fascicules, repent or ascending; thin-walled or occasionally with slightly thickened walls up to 0.5 μm, often with crystalline or granulose yellow incrustations. ***Clamp connections*** absent.

###### Distribution and ecology.

Known from a single locality in a deciduous forest in France.

###### Material studied.

France • Oise, Sacy-le-Grand, marais de Sacy, coord. 49°19'57"N, 02°36'08"E, thicket of *Acercampestre* and *Corylus* sp., 29 Oct 2014, P. Clowez, F. Petit and P.-A. Moreau *PAM14102921* (LIP).

###### Notes.

Dermolomaaff.bellerianum is a well-supported member of D.sectionConica and closely related but distinct from *D.bellerianum*. It has a very similar morphology to this species but differs from it by the wider spores (Q < 1.6). We do not formally describe it as a new species because it is represented only by a single poorly documented collection. This collection came from the very same place as a typical collection of *D.bellerianum* (CL/F13.170) one year later, with an extremely similar macromorphology. The presence of clampless basidia and the inamyloid, relatively narrow, ellipsoid spores (av. Q = 1.49) of *D.pseudocuneifolium* (based on the type) are unique for the genus, thus suggesting that D.aff.bellerianum can be identical with *D.pseudocuneifolium*. However, the name is well adopted in the literature and used for an amyloid *Dermoloma* species (Vesterholt 2012). To avoid further confusion, we recommend to treat the name *D.pseudocuneifolium* as dubious.

##### ﻿ Dermoloma carpathicum

Taxon classificationAnimaliaAgaricalesTricholomataceae

Adamčíková & Jančovič. sp. nov.

E5A45690-9DC8-5167-9484-44F9E19337EB

856332

Figs 15c, d , 18

###### Etymology.

The majority of the collections originate from the Carpathian Mts.

###### Holotype.

Romania • Vladeasa Mts., pasture 1.2 km N of Belis, elev. 1060–1085 m, coord. 46°41'47"N, 23°02'10"E, terrestrial, 5 Oct 2014, S. Adamčík (SAV F-4268).

###### Diagnosis.

European species; basidiomata small to moderately large; pileus hygrophanous and indistinctly striate near the margin; lamellae ochraceous-gray to almost white; stipe without darker squamules or fibrils; spores inamyloid and on average wider than 3.6 µm wide and/or Q < 1.4.

***Pileus*** (6–)10–41 mm; convex to plano-convex, soon expanding to plane, indistinctly umbonate, rarely broadly obtusely conical, rarely lobate; margin usually not striate, sometimes indistinctly translucently striate, recurved when old, when dry radially cracking; surface usually smooth near margin and rugulose or rough near center, sometimes completely smooth, rarely rough near margin and smooth near center or completely rough and rugulose, hygrophanous; color when young dark brown (6F3, 6F4, 7E3, 7F4), when mature gradually paler towards margin, brown (5E4, 6E4), light brown (5D4), grayish brown (5D3, 6E3), brownish ochraceous (5C3, 5C4), brownish gray (5C2), grayish ochraceous (5B3) to ochraceous-gray (5B2), near center dark brown (5F4, 6F3, 6F4, 6F5, 7F4, 7F5), brown (5E4, 5E5, 6E4, 6E6) to grayish brown (5D3, 5E3, 6D3, 6E3). ***Stipe*** (14–)18–46(–48) × 1–5(–7) mm; cylindrical, rarely narrowly fusiform, usually narrowed towards the base, usually flexuous; surface finely longitudinally striate, finely pruinose or granulose near lamellae, towards the base finely fibrillose or squamulose; color near lamellae paler ochraceous-gray (5B2) to almost white, towards the base brownish gray (5C2, 6C2), brownish ochraceous (5C3), grayish brown (6D3, 6E3), brown (6E4) to dark brown (6F3, 6F4), often with darker fibrils or squamules on paler background. ***Lamellae*** L = (18–)20­–34, l = (0–)1–3(–7); 2–7 mm wide; adnate-emarginate and decurrent with tooth; color ochraceous-gray (5B2) to almost white; edges entire or slightly irregular. ***Context*** when young compact, later fragile; odor farinaceous.

**Figure 18.**
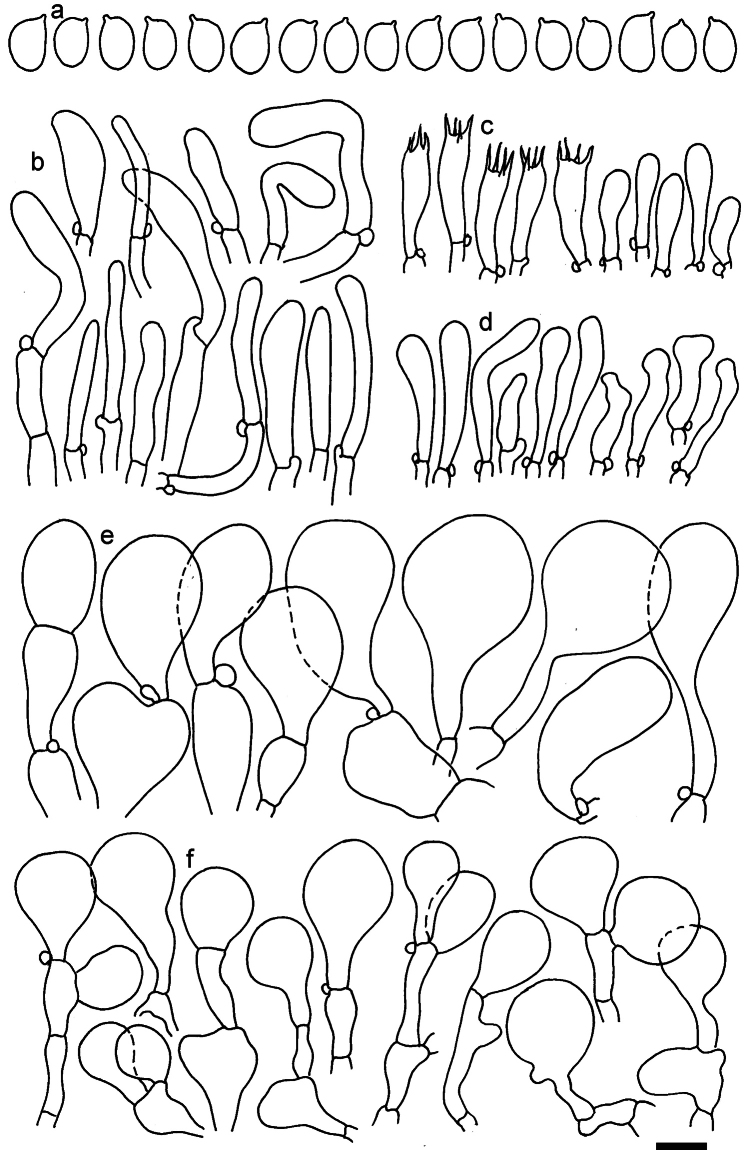
*Dermolomacarpathicum* (SAV F-3867), microscopic elements. **a** Spores; **b** caulocystidia; **c** basidia and basidioles; **d** marginal cells; **e** pileipellis elements near the pileus margin; **f** pileipellis elements near the pileus center. Scale bar: 5 µm for spores and 10 µm for other elements.

***Spores*** (4.5–)4.8–5.5–6.2(–8) × (3.2–)3.6–4–4.6(–5.6) μm; broadly ellipsoid to ellipsoid, Q = (1.16–)1.31–1.40–1.49(–1.74); walls inamyloid, often dextrinoid; hilar appendage ca. 0.5–1.5 μm long. ***Basidia*** (21–)23.5–27.3–31(–38) × (5.5–)6–6.5–7(–8) μm; clavate; with 4 sterigmata. ***Basidioles*** first cylindrical, then clavate, ca. 3.5–6 μm wide. ***Marginal cells*** (17–)21.5–26.8–32(–38) × (3.5–)5–6.2–7.5(–10.5) μm; not well-differentiated, clavate, rarely cylindrical, flexuous, sometimes constricted or lobate. ***Pileipellis*** 60–70 μm deep; suprapellis 35–50 μm deep, of one or two layers of inflated, densely arranged cells; subpellis well-differentiated, 20–27 μm deep, of densely packed, irregularly oriented, intricate, 3–13 μm wide hyphae, gradually passing to horizontally oriented hyphae in trama; hyphal terminations with brownish parietal pigments, and occasionally with darker brown incrusted pigments near septa of terminal cells and in subpellis, walls thickened especially in subpellis up to 1.2 μm. Terminal cells near pileus margin (20.5–)32–42.3–53(–88) × (8.5–)16–20.9–26(–40) μm; usually obpyriform, sphaeropedunculate or clavate, sometimes ellipsoid or subglobose; subterminal cells usually narrower and branched, flexuous, often lobate or with lateral swellings. Terminal cells near pileus center (14–)28–41.5–55(–82) × (5–)14–20.1–26(–38.5) μm; similar to cells near margin; subterminal cells narrower, cylindrical or ventricose, strongly flexuous or nodulose, usually branched, often with lateral swellings or irregularly lobate, more frequently with brown incrusted pigments. ***Caulocystidia*** (15–)23.5–35.2–46(–75) × (2.5–)4–5.5–7(–9) μm; clavate or cylindrical, usually not or only slightly flexuous, often clustered in small ascending fascicules, sometimes individual and repent; usually with slightly thickened walls up to 0.5 μm, often with crystalline or granulose yellow incrustations. ***Clamp connections*** present.

###### Distribution and ecology.

Known from Germany, Romania and Slovakia; in semi-natural grasslands, ancient extensively grazed pastures and meadows.

###### Additional material studied.

Germany • Baden-Württemberg, Justingen, Schachenheide, coord. 48°24'35"N, 09°40'25"E, terrestrial in semi-natural grassland, 3 Oct 2021, F. Hampe (SAV F-20895). Romania • Vladeasa Mts., pasture 1.2 km N of Belis, elev. 1060–1085 m, coord. 46°41'47"N, 23°02'10"E, terrestrial, 5 Oct 2014, S. Adamčík (SAV F-4268); • ibid., 5 Oct 2014, S. Adamčík (SAV F-4274); • ibid., 6 Oct 2014, S. Jančovičová (SAV F-4304); • ibid., 7 Oct 2014, S. Adamčík (SAV F-4336). Slovakia • Javorie Mts., Slatinské Lazy, Jombíkovci, meadow near Matúšov hájik cottage, elev. 460 m, coord. 48°29'47"N, 19°19'08"E, terrestrial, 22 Oct 2020, S. Adamčík (SAV F-20774); • Javorníky Mts., 2 km S of Vysoká nad Kysucou, Vrchrieka, elev. 780–810 m, coord. 49°21'42"N, 18°33'05"E, terrestrial in semi-natural grassland, 12 Oct 2012, S. Adamčík (SAV F-3867); • Kremnické vrchy Mts., 3 km W of Tajov, elev. 710 m, coord. 48°44'53"N, 19°01'35"E, terrestrial in a extensively grazed pasture, 24 Oct 2020, S. Adamčík (SAV F-20805); • ibid., 24 Oct 2020, S. Adamčík (SAV F-20806); • Laborecká vrchovina Mts., pasture 1 km NW of Vyšná Jablonka, elev. 400–450 m, coord. 49°09'31"N, 22°06'18"E, terrestrial, 21 Sep 2006, S. Adamčík (SAV F-4146); • Malé Karpaty Mts., 0.5 km SE of Sološnica, Božia muka, coord. 48°27'27"N, 17°14'45"E, terrestrial in semi-natural grassland, 12 Jun 2013, V. Kučera (SAV F-3936); • Podbeskydská vrchovina Mts., pasture on N margin of the village Mútňanská píla, elev. 780–830 m, coord. 49°28'26"N, 19°17'19"E, terrestrial, 10 Oct 2012, S. Adamčík (SAV F-3839); • Štiavnické vrchy Mts., 2.5 km NW of Prenčov, Horné Majere, elev. 420–460 m, coord. 48°22'54"N, 18°54'01"E, terrestrial in semi-natural grassland, 12 Oct 2015, M. Caboň (SAV F-4743); • ibid., 12 Oct 2015, M. Caboň (SAV F-4744); • Zvolenská kotlina Basin, pasture E of the village Bečov, elev. 400–450 m, coord. 48°38'48"N, 19°14'49"E, terrestrial, 28 Aug 2014, M. Caboň (SAV F-4247); • ibid., 28 Aug 2014, S. Adamčík (SAV F-4248).

###### Notes.

*Dermolomacarpathicum* is a member of D.subgenusDermoloma, section Dermoloma. It is sister to *D.simile* (Fig. 2), with which it shares a very similar field aspect: it reminds of small individuals of *D.cuneifolium*. Another similar species, *D.fuscobrunneum* is also a member of D.sectionDermoloma, but is not closely related. Morphological delimitation of these three species is problematic, and we recommend to sequence them for verification. In the field *D.carpathicum* has lamellae and the stipe with brownish or beige colors, sometimes with darker fibrils towards the base of the stipe and a translucently striated pileus margin when wet. The striate pileus margin is a good character to distinguish it from *D.cuneifolium*, which can sometimes have basidiomata of similar small sizes. *Dermolomacarpathicum* was included in the phylogenetic study by Sánchez-García et al. (2021) as “*Dermoloma* sp. 3”. According to our data, this species is common in the Carpathian Mts. (13 collections from Romania and Slovakia), but is rare or absent in Western Europe represented by only a single collection from Germany.

##### ﻿ Dermoloma compactum

Taxon classificationAnimaliaAgaricalesTricholomataceae

Friebes & Karich sp. nov.

1E44BA36-5258-5640-8B16-B9424AF1C7FE

856333

Figs 15e, f , 19

###### Etymology.

Basidiomata are bulky and short.

###### Holotype.

Slovenia • Zgornje Gorje, Goreljek, slope next to Šport Hotel Pokljuka, elev. 1260 m, coord. 46°20'15"N, 13°57'40"E, soil among moss and grass, 10 Oct 2021, G. Friebes *GF20211010* (SAV F-23421).

###### Diagnosis.

European species; basidiomata relatively robust but medium to small; pilei usually up to 25 mm in diameter, brown to dark brown; stipe 3–6 mm wide with brown fibrils or squamules; spores amyloid, on average ca. 4.2 µm wide.

***Pileus*** 10–21(–30) mm; convex to almost plane, broadly conical; margin not striate, deflexed; surface finely rough to almost smooth near margin, distinctly radially rugulose near center, sometimes pruinose, hygrophanous and discoloring first in center; color near margin brown (5E4, 5F5, 5E7, 6E4, 7E5) to dark brown (6F4), when dry brown (6E5), dark blond (5D4) to grayish brown (5D3), near center dark brown (6F4), when dry brown (5E5, 5F7, 6E7) to light brown (5D4, 5D5). ***Stipe*** (16–)21–40 × 3–6(–9) mm; usually fusiform, sometimes cylindrical or flexuous, narrowed towards the base; surface pruinose near lamellae, towards the base fibrillose-squamulose; color near lamellae yellowish white (4A2) to ochraceous-gray (paler than 5B2), towards the base grayish ochraceous (paler than 5B3) to light brown (5D4), with darker fibrils or squamules (dark brown 5F5, 5F7). ***Lamellae*** L = 22–36, l = (1–)3; up to 4 mm wide; adnexed to adnate-emarginate; color ochraceous-gray (5B2) to brownish ochraceous (5C3); edges entire. ***Context*** when young compact, later in pileus fragile; odor farinaceous.

**Figure 19.**
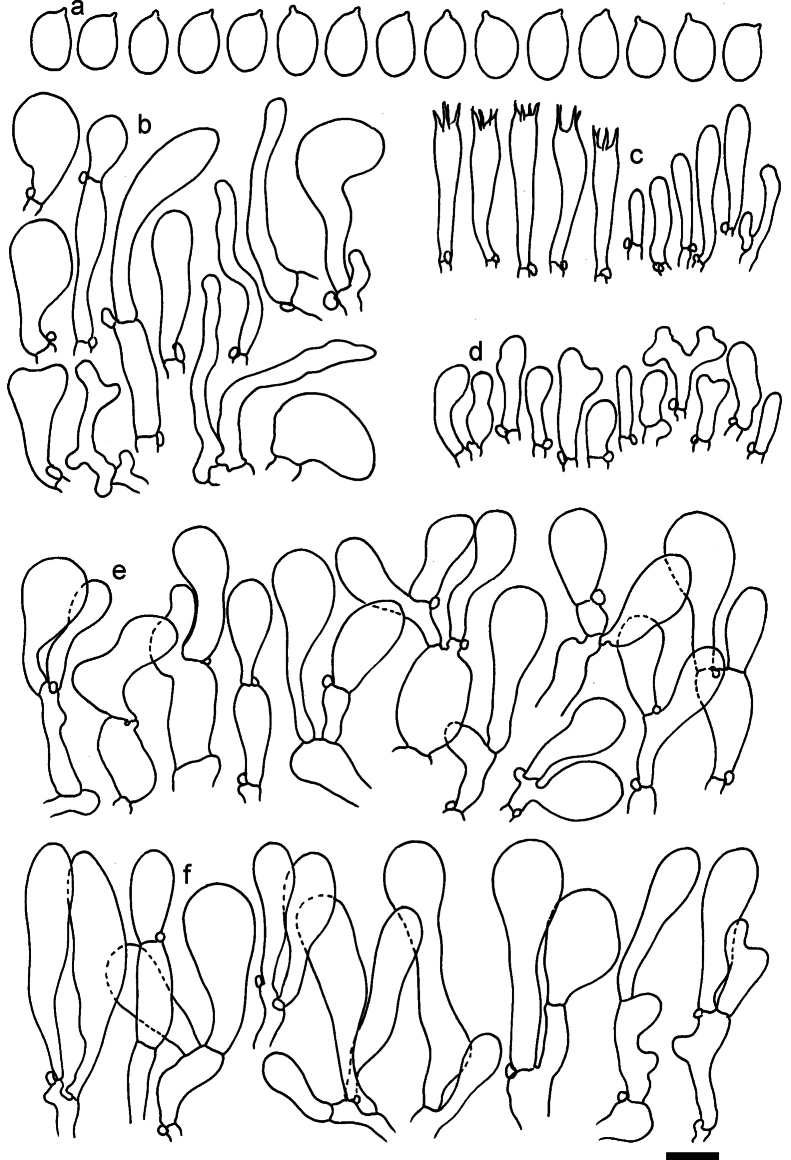
*Dermolomacompactum* (SAV F-3838), microscopic elements. **a** Spores; **b** caulocystidia; **c** basidia and basidioles; **d** marginal cells; **e** pileipellis elements near the pileus margin; **f** pileipellis elements near the pileus center. Scale bar: 5 µm for spores and 10 µm for other elements.

***Spores*** (5.5–)5.8–6.2–6.8(–7.4) × (3.6–)3.9–4.2–4.5(–5.1) μm; ellipsoid, Q = (1.26–)1.41–1.5–1.6(–1.67); walls amyloid, thin-walled; hilar appendage ca. 0.5–1.5 μm long. ***Basidia*** (23–)26–30.4–35(–41) × (5–)6–6.6–7(–8) μm; clavate; mainly with 4 sterigmata, occasionally with 2 sterigmata, rarely 1 sterigma. ***Basidioles*** first cylindrical, then clavate, ca. 3–6.5 μm wide. ***Marginal cells*** (8–)13–18.9–24(–30) × (3–)4.5–6.1–8(–10) μm; not well-differentiated, clavate, occasionally lobate or flexuous, rarely bifurcate. ***Pileipellis*** 75–95 μm deep; suprapellis 40–47 μm deep, usually of one or two layers of inflated, densely arranged cells; subpellis well-differentiated, 30–50 μm deep, of densely packed, mainly horizontally oriented, intricate, 3–10 μm wide hyphae, gradually passing to horizontally oriented hyphae in trama; hyphal terminations with brownish parietal pigments, near septa of terminal cells with dark brown parietal and locally also finely incrusted pigments, walls thickened especially in subpellis up to 1 μm. Terminal cells near pileus margin (12–)24–34.5–46(–55) × (8–)11–16.2–21(–36) μm; sphaeropedunculate or clavate, rarely obpyriform; subterminal cells mainly narrowly cylindrical, occasionally inflated and clavate, often flexuous, nodulose or with lateral swellings, occasionally branched. Terminal cells near pileus center (18–)24–34.4–46(–59) × (8–)10–14.9–20(–26) μm; mainly clavate and less frequently sphaeropedunculate, occasionally flexuous, nodulose or lobate; subterminal cells mainly narrower and subcylindrical, usually flexuous and lobate or with lateral swellings, occasionally branched. ***Caulocystidia*** (14–)19–31.5–46(–82) × (4–)5–7.1–9(–15) μm; mainly cylindrical and flexuous, occasionally obpyriform, clavate or sphaeropedundulate, occasionally lobate or nodulose, clustered in repent or ascending fascicules; usually with slightly thickened walls up to 0.5 μm, near septa with brownish parietal pigments. ***Clamp connections*** present.

###### Distribution and ecology.

Known from France, Germany, Slovakia, Slovenia and Wales (United Kingdom); in semi-natural grassland on neutral to alkaline soil.

###### Additional material studied.

France • Pyrénées-Atlantiques, Portalet, coord. 42°48'59.7"N, 00°24'53.74"E, terrestrial on pasture, 7 Oct 2022, M. Caboň (SAV F-22285). Germany • Sachsen, Adorf, NSG Zeidelweide, coord. 50°18'01.22"N, 12°13'17"E, regularly mowed semi-natural grassland, 23 Sep 2022, A. Karich and R. Ullrich *IHI-22Der01* (GLM-F137756, as D.cf.pseudocuneifolium). Slovakia • Kremnické vrchy Mts., 3 km W of Tajov, elev. 710 m, coord. 48°44'53"N, 19°01'35"E, terrestrial in extensively grazed pasture, 24 Oct 2020, V. Shapkin (SAV F-20794); • Podbeskydská vrchovina Mts., 1.5 km SW of Beňadovo, Beňadovské rašelinisko, elev. 720–750 m, coord. 49°25'16"N, 19°19'55"E, terrestrial on meadow, 10 Oct 2012, S. Adamčík (SAV F-3838). United Kingdom • Wales, Graig, Llanymddyfri, coord. 51°58'41"N, 03°42'51"E, 24 Sep 2021, G. Griffith *DM0921* (ABS, as *D.magicum*).

###### Notes.

*Dermolomacompactum* is a member of D.subgenusAmylospora, section Atrobrunnea. It shares a distinctive morphology with two other related European species, *D.josserandii* and *D.pseudojosserandii*, as their basidiomata are also sturdy and reminiscent of small individuals of *Tricholoma*, but the pileus size is relatively small. They form a well-supported clade together with two additional North American species (Fig. 2). *Dermolomacompactum* can be distinguished in the field from *D.josserandii* and *D.pseudojosserandii* by the darker brown pilei and stipes with distinct brown fibrils toward the base. In addition, it differs by narrower spores (on average ca. 4.2 μm). This species was included in the phylogenetic study by Sánchez-García et al. (2021) as “*Dermoloma* sp. 11”.

##### ﻿ Dermoloma confusum

Taxon classificationAnimaliaAgaricalesTricholomataceae

P.-A. Moreau & Courtec. sp. nov.

7BAA391D-3C9E-5AD9-BD5E-CD54924DDF9A

856334

Figs 20a, b , 21

###### Etymology.

The epithet refers to previous confusion with *D. pseudocuneifolium*.

###### Holotype.

United Kingdom • Wales, Pembrokeshire, Upton Castle, coord. 51°42'22"N, 04°51'57"E, terrestrial in lawn, 26 Oct 2014, S. Adamčík (SAV F-4420).

###### Diagnosis.

European species; basidiomata medium sized, collybioid; pilei and stipes brown; lamellae usually darker brownish gray or grayish brown; marginal cells clavate, lageniform or cylindrical and without projections; caulocystidia clavate or fusiform.

***Pileus*** (7–)10–35(–40) mm; convex to plane, sometimes indistinctly umbonate, sometimes lobate, rarely obtusely conical; margin translucently striate to half of the radius, recurved when old; surface near margin smooth, near center rugulose, rough or pitted, hygrophanous; color when young dark brown (6F6), when mature near margin dark brown (6F3, 6F4, 7F3), brown (5E5, 5E6, 6E4, 6E5), light brown (6D4), when dry brown (5E4), grayish brown (6D3), brownish ochraceous (5C3, 5C4, 6C4), light brown (5D4, 5D6), grayish ochraceous (5B3), pale ochraceous (5A3), near center dark brown (5F6, 6F3, 6F4, 6F5, 6F6, 7F3, 7F4, 7F5), brown (5E4, 6E4), light brown (5D5), when dry brown (5E4, 5E5), light brown (5D4, 5D5, 5D6), brownish ochraceous (5C4, 5C5), rarely grayish brown (6D3) or dark brown (6F5). ***Stipe*** (10–)17–55(–63) × (1–)2–6(–7) mm; cylindrical, not distinctly narrowed towards the base, flexuous, grooved; surface finely longitudinally striate, pruinose near lamellae, towards the base shiny and glabrous; color near lamellae ochraceous-gray (5B2), brownish ochraceous (5C3), light brown (5D4), brownish gray (6C2, 6D2), grayish brown (6D3, 6E3), light brown (6D4), rarely brown (6E4), near the base dark brown (5F3, 5F5, 6F3, 6F4, 6F5, 7F4, 7F6), brown (5E4, 5E5, 6E4, 6E5), grayish brown (5E3, 6D3, 6E3), rarely brownish gray (6D2). ***Lamellae*** L = (18–)21–32(–38), l = 1–3(–7); 2–6 mm wide; adnate-emarginate, sometimes decurrent with tooth; color brownish gray (5C2, 6C2, 6C2, 6D2), grayish brown (5D3, 6D3, 7D3), brownish ochraceous (6C3, 6C4); edges entire. ***Context*** when young elastic, later fragile; odor farinaceous.

**Figure 20.**
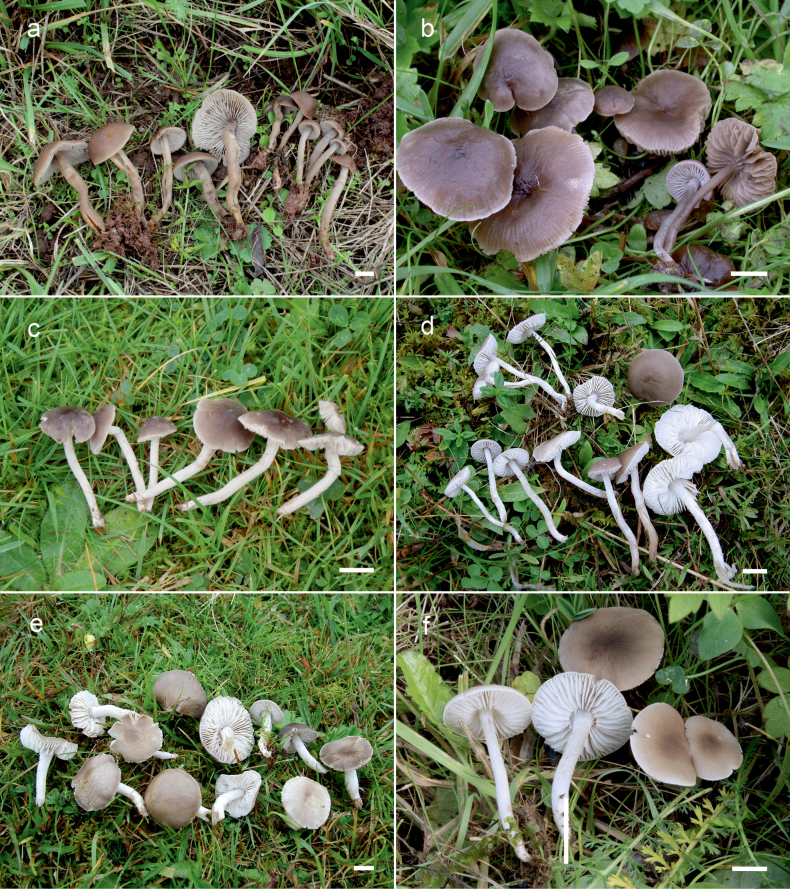
Basidiomata of *Dermoloma* in field appearance. **a***Dermolomaconfusum* (SAV F-20536), photo S. Jančovičová; **b***Dermolomaconfusum* [LIP (*PAM05102801*)], photo P.-A. Moreau; **c***Dermolomacuneifolium* (SAV F-4395), photo D. Harries; **d***Dermolomacuneifolium* (SAV F-4702), photo S. Jančovičová; **e***Dermolomacuneifolium* (SAV F-4265), photo S. Jančovičová; **f***Dermolomacuneifolium* (SAV F-23426), photo G. Friebes. Scale bar: 5 µm.

**Figure 21.**
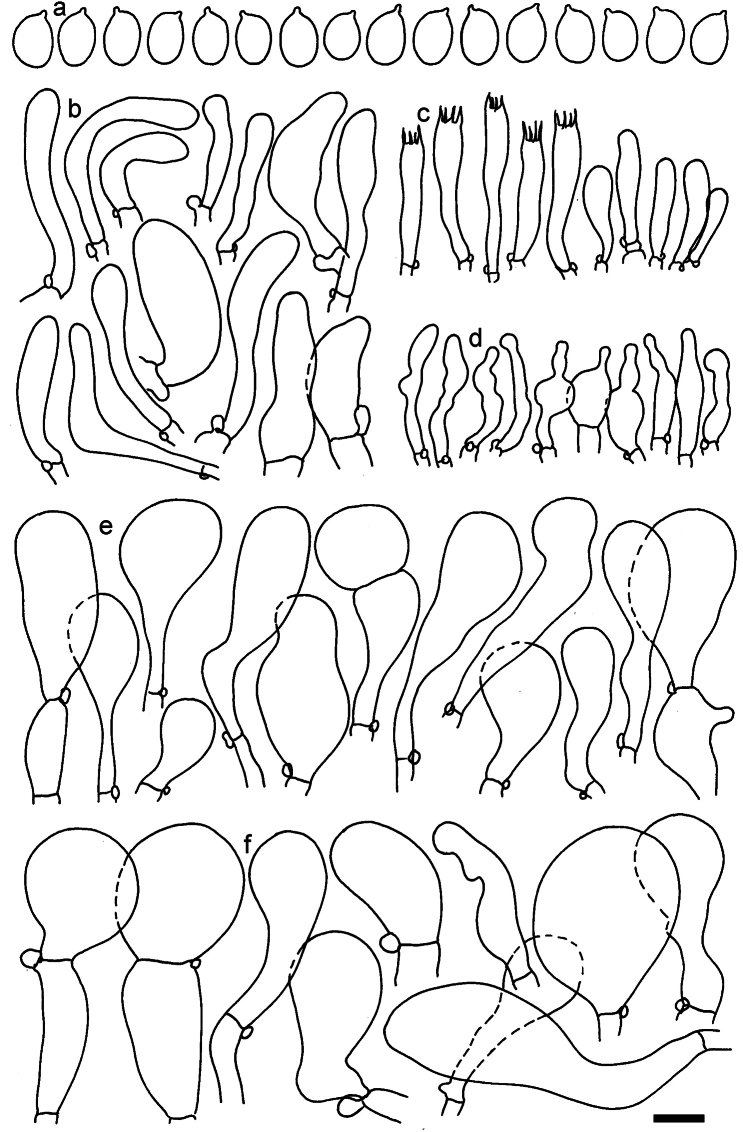
*Dermolomaconfusum* (SAV F-4420, holotype), microscopic elements. **a** Spores; **b** caulocystidia; **c** basidia and basidioles; **d** marginal cells; **e** pileipellis elements near the pileus margin; **f** pileipellis elements near the pileus center. Scale bar: 5 µm for spores and 10 µm for other elements.

***Spores*** (5.1–)5.5–6–6.5(–7.7) × (3.4–)3.7–4–4.3(–5.4) μm; ellipsoid to narrowly ellipsoid, Q = (1.27–)1.40–1.50–1.61(–1.85); walls amyloid; hilar appendage 0.5–1.5 μm long. ***Basidia*** (23–)25–29.4–34(–60) × (5–)6–6.5–7(–8.5) μm; clavate; with 4 sterigmata. ***Basidioles*** first cylindrical, then clavate, ca. 3–6 μm wide. ***Marginal cells*** (13–)21–30.7–40.5(–53) × (3.5–)4.5–6–7.5(–9.5) μm; clavate, lageniform or cylindrical, usually flexuous, apically often constricted or appendiculate. ***Pileipellis*** 45–50 μm deep; suprapellis of mainly two layers of inflated cells; subpellis 13–20 μm deep, not well-differentiated, of densely packed, irregularly oriented, puzzled, 3–10 μm wide hyphae, not sharply delimited from horizontally oriented hyphae in trama; hyphal terminations with brownish parietal pigments, thin-walled, near septa and in subpellis with dark brown and sometimes also slightly incrusted pigments and occasionally with thickened walls up to 0.7 μm. Terminal cells near pileus margin (20.5–)33.5–43.9–54(–82) × (10–)14–19.5–24.5(–33.5) μm; usually clavate or obpyriform, sometimes sphaeropedunculate, rarely ellipsoid or subglobose; subterminal cells usually narrower and branched, cylindrical or fusiform, occasionally inflated, sometimes with lateral swellings. Terminal cells near pileus center (14.5–)31–46–60(–109) × (7.5–)13.5–20.3–27(–43.5) μm; similar to cells near margin but often flexuous and constricted towards septa; subterminal cells similar to cells near margin. ***Caulocystidia*** (10–)23–33.8–45(–62) × (3–)4.5–7.4–10.5(–17) μm; clavate or fusiform-ventricose, often flexuous, often clustered in small ascending fascicules, sometimes individual and repent; usually with slightly thickened walls up to 0.5 μm, with brownish to brown parietal pigments. ***Clamp connections*** present.

###### Distribution and ecology.

Mainly in temperate-montane regions of Central and Western Europe. So far known from France, Germany, Slovakia and Wales (United Kingdom); in grasslands on calcareous soil.

###### Additional material studied.

France • Baives, Réserve naturelle régionale des Monts de Baives, coord. 04°11'24"E, 50°03'53"N, Oct 2008, C. Lécuru *CL/F08.245* (LIP, as *D.pseudocuneifolium*); Boulogne-sur-Mer, Cimetière de l’Est, grassy alley, 23 Oct 2006, A. Flahaut *CL/F06.248* (LIP, as D.cuneifoliumvar.pragensis); • Lille, jardin botanique de la Faculté de pharmacie, coord. 49°19'57"N, 02°36'08"E, urban grassland on rich calcareous soil, 13 Nov 2016, R. Courtecuisse and P.-A. Moreau *PAM16110301* (LIP); • ibid., 14 Nov 2014, P.-A. Moreau *PAM14111401* (LIP); • ibid., 28 Oct 2005, P.-A. Moreau *PAM05102801* (LIP); • Dannes, Dunes du Mont Saint-Frieux, coord. 50°36'05"N, 01°35'17"E, 24 Oct 2013, A. Flahaut *CL/F13.234* (LIP, as D.intermediumvar.coniferarum). Germany • Baden-Württemberg, Justingen, Schachenheide, coord. 48°24'35"N, 09°40'25"E, terrestrial in semi-natural grassland, 3 Oct 2021, S. Adamčík (SAV F-20913); • Rheinland-Pfalz, Sobernheim, elev. 215 m, coord. 49°47'18"N, 07°40'35"E, terrestrial in semi-natural grassland, 9 Nov 2019, C. Manz (SAV F-20536); • ibid., 9 nov 2019, F. Hampe (SAV F-20564); • Schleswig-Holstein, Fehmarn, Deich, 17 Nov 2015, T. Böhning *AG152* (ELTE). Slovakia • Kremnické vrchy Mts., pasture 0.5 km W of Tajov, elev. 600 m, coord. 48°44'54"N, 19°03'31"E, terrestrial, 24 Oct 2020, S. Adamčík (SAV F-20814); • ibid., 24 Oct 2020, S. Adamčík (SAV F-20824); • ibid., 25 Oct 2020, M. Caboň (SAV F-20837); • ibid., 25 Oct 2020, M. Caboň (SAV F-20838); • ibid., 25 Oct 2020, S. Jančovičová (SAV F-20833); • ibid., 25 Oct 2020, S. Jančovičová (SAV F-20842). United Kingdom • Wales, Pembrokeshire, Upton Castle, coord. 51°42'22"N, 04°51'57"E, terrestrial in lawn, 26 Oct 2014, S. Adamčík (SAV F-4418); • ibid., 26 Oct 2014, S. Adamčík, (SAV F-4422).

###### Notes.

*Dermolomaconfusum* corresponds to the widely accepted concept of *D.pseudocuneifolium* (Arnolds 1993; Contu et al. 2008) and, as a species with amyloid spores, it is a member of D.subgenusAmylospora, section Atrobrunnea. It belongs to a larger clade of species with mainly collyboid basidiomata that includes also *D.curvicystidiatum*, *D.griseobasale*, *D.phaeopodium* and *D.pruinosipes* (Fig. 2). In the field it is very difficult to distinguish from other members of this clade, and especially from the very similar *D.phaeopodium*. A useful distinguishing character seems to be the presence of marginal cells that are cylindrical or lageniform and apically constricted or mucronate. *Dermolomapseudocuneifolium* was first introduced by Herink (1958) as an invalid name (no Latin description) and later adopted by Bon (1986). Their concept was based on a misapplication of *D.cuneifolium* as a species with amyloid spores by Josserand (1943) based on French material. Bon (1986) designated his collection as the type and the protologue. Bon‘s notes attached to the type specimen both describe the spores as amyloid, 7.5–9 × 4–5 μm. Our type sequencing failed, but the type specimen (a single basidiome) showed bisporic basidia without clamp connections and inamyloid narrow spores on av. 5.2 × 3.5 μm, Q = 1.49. These inamyloid spores and two-spored basidia are consistent with members of the *D.bellerianum* complex and are contrary to the current use for a species with amyloid spores (Wilhelm 1992; Arnolds 1993, 1995; Contu et al. 2008; Sánchez-García et al. 2021). Therefore, we here consider *D.pseudocuneifolium* a dubious name. *Dermolomaconfusum* was included in the previous phylogenetic study by Sánchez-García et al. (2021) as “*D.pseudocuneifolium*”.

##### ﻿ Dermoloma cuneifolium

Taxon classificationAnimaliaAgaricalesTricholomataceae

(Fr.) Singer ex Bon, Doc. Mycol. 17(65): 51. 1986.

4352AB79-4C4E-5816-94B8-707D97251E0C

129540

Figs 20c–f , 22

Dermolomaemilii-dlouhyi Svrček, Česká Mycol. 20(3): 147. 1966. Syn.

###### Neotype.

(designated by Arnolds 1992): Sweden • Småland, Femsjö, the slope east of Avaberget, amongst mosses, low *Hieracium* sp., *Trifoliumpretense* etc., in disused, partly *Calluna* grown pasture-land, 19 Sep 1948, S. Lundell and G. Haglund *5101* (UPS-F-631065).

###### Distinguishing characters.

European species; basidiomata medium sized; pilei up to 35 mm in diameter; stipes 2–6 mm wide; lamellae ochraceous-gray to brownish gray; spores inamyloid, 4.7–5.3 × 3.5–4 μm; basidia up to 6.5 μm wide.

***Pileus*** (8–)12–35(–44) mm; convex, soon expanding to plane, often weakly depressed, sometimes indistinctly umbonate, sometimes lobate; margin usually not striate, indistinctly translucently striate when wet; surface near margin smooth, near center radially rugulose and veined, sometimes pitted or rough, not hygrophanous; color when young dark brown (6F2, 6F3, 6F4, 7F3, 7F4), when mature near margin usually grayish brown (5D3, 6D3, 6E3, 7E3), often brownish gray (5D2, 6C2), brown (5E4, 6E4, 7E4) to dark brown (6F3, 6F4, 7F3), rarely grayish ochraceous (5B3), brownish ochraceous (5C3) or dark blond (5D4), towards margin often gradually passing to narrow paler brownish gray (6C2), ochraceous-gray (5B2) to almost white zone, near center usually dark brown (6F2, 6F3, 6F4, 7F3, 7F4, 7F5) to black, sometimes brown (5E4, 6E4, 7E4), rarely grayish brown (6E3). ***Stipe*** (17–)21–50(–55) × (1–)2–6(–8) mm; cylindrical or fusiform, usually narrowed towards the base, flexuous; surface near lamellae pruinose or granulose, towards the base granulose or finely fibrillose, finely longitudinally striate; color near lamellae white, pale gray (B1), ochraceous-gray (5B2), brownish gray (5C2, 6C2), near the base usually brownish gray (5C2, 5D2, 6C2, 6D2) or grayish brown (5D3, 5E3, 6D3), sometimes ochraceous-gray (5B2, 6B2), rarely grayish ochraceous (5B3), brownish ochraceous (5C3) or brown (5E4). ***Lamellae*** L = (19–)23–38(–42), l = 1–7; 4–9 mm wide; adnate-emarginate, sometimes decurrent with tooth or adnexed; color usually ochraceous-gray (5B2) or brownish gray (5C2, 6C2), often almost white, rarely grayish brown (6D3); edges entire, rarely serrulate. ***Context*** fragile; odor farinaceous.

**Figure 22.**
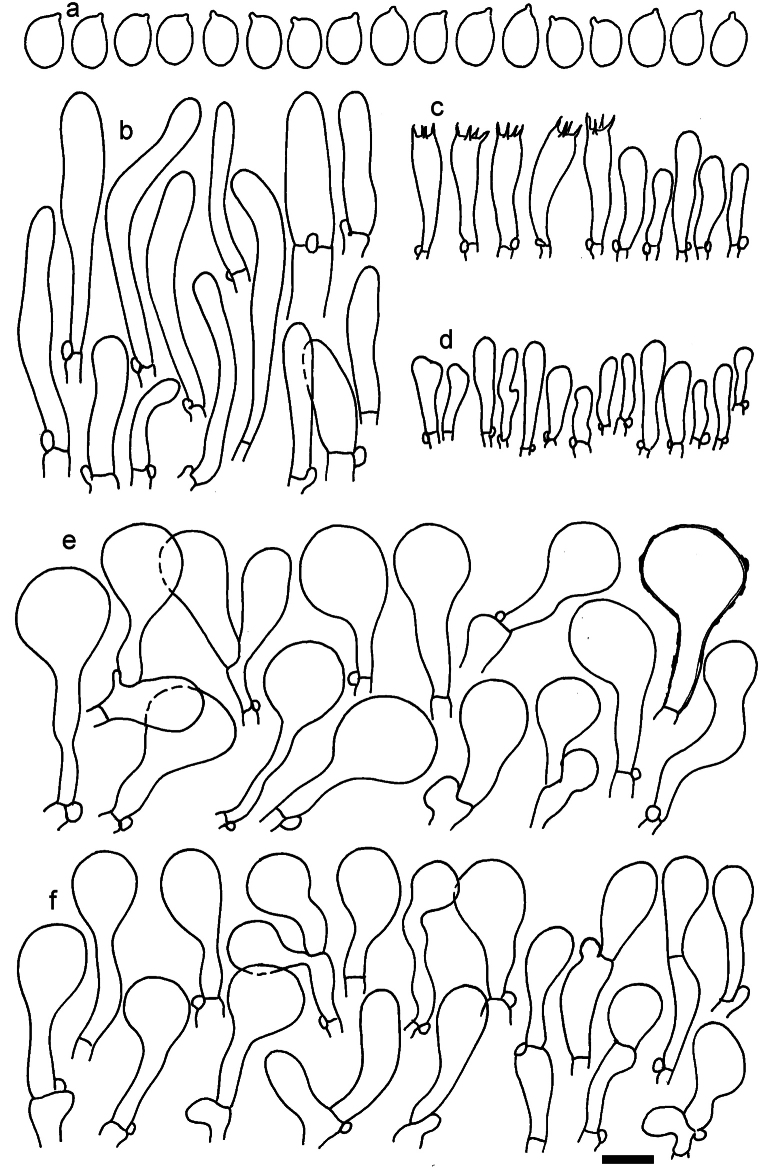
*Dermolomacuneifolium* (UPS-F-631065, neotype), microscopic elements. **a** Spores; **b** caulocystidia; **c** basidia and basidioles; **d** marginal cells; **e** pileipellis elements near the pileus margin; **f** pileipellis elements near the pileus center. Scale bar: 5 µm for spores and 10 µm for other elements.

***Spores*** (4.2–)4.7–5–5.3(–6.1) × (3.2–)3.5–3.8–4(–4.4) μm; broadly ellipsoid to ellipsoid, Q = (1.14–)1.24–1.32–1.40(–1.53); walls inamyloid, sometimes thick-walled and dextrinoid; hilar appendage 0.5–1 μm long. ***Basidia*** (20–)23.5–25.9–28.5(–32) × (5–)5.5–6–6.5(–7) μm; clavate; with 4 sterigmata. ***Basidioles*** first cylindrical, then clavate, ca. 3–6.5 μm wide. ***Marginal cells*** (10–)14–19.1–24(–28) × (2–)3.5–4.7–6(–8) μm; cylindrical or clavate. ***Pileipellis*** 55–65 μm deep; suprapellis of mainly one, occasionally two layers of inflated cells, gradually passing to 18–25 μm deep subpellis of densely packed, irregularly oriented, puzzled, 3–8 μm wide hyphae, not sharply delimited from horizontally oriented hyphae in trama; hyphal terminations with brownish parietal pigments, thin-walled or occasionally thickened up to 1 μm and with brown incrusted pigments especially near septa of terminal cells. Terminal cells near pileus margin (11–)28–37.4–46.5(–68) × (6–)13.5–17.9–22(–33) μm; usually sphaeropedunculate, sometimes obpyriform or clavate; subterminal cells usually narrower and implemented in subpellis, often branched, sometimes ventricose and short, rarely with lateral swellings. Terminal cells near pileus center (13–)27–37–47.5(–59) × (5.5–)11–16.4–21.5(–32) μm; usually obpyriform, clavate or sphaeropedunculate, rarely ellipsoid or clavate-lageniform, sometimes with one or two central constrictions; subterminal cells similar to cells near margin. ***Caulocystidia*** (10–)25–37.6–50.5(–81) × (3–)4–6.6–9(–12) μm; clavate or cylindrical, usually not or only slightly flexuous, often clustered in small ascending fascicules, sometimes individual and repent; occasionally with slightly thickened walls up to 0.5 μm, often with crystalline or granulose yellow incrustations. ***Clamp connections*** present.

###### Distribution and ecology.

Probably the most common *Dermoloma* species, widely distributed in Europe; in semi-natural grasslands, dunes, forests, cemeteries and parks, often collected in habitats with low conservation value as the single *Dermoloma* species.

###### Additional material studied.

Austria • Burgenland, Oberwart, Rechnitz, Galgenberg, elev. 344 m, coord. 47°17'52"N, 16°25'09"E, semi-dry grassland, soil among mosses and grass, 17 Nov 2019, G. Friebes *GF20190133* (SAV F-23422); • Steiermark, Südoststeiermark, Straden, Stradner Kogel, Berghölzer, elev. 411 m, coord. 46°49'53"N, 15°55'02"E, soil among mosses and grass, 6 Oct 2019, G. Friebes *GF20190080* (SAV F-23426). Croatia • Gorski kotar area, Crni Lug – Velika Voda, coord. 45°24'36"N, 14°42'15"E, mowed grassland with short grass and mosses, 4 Oct 1998, A. Mešić (CNF 5/204); • 2.2 km SE/S-SE of Brlog Ozaljski village, near Ozalj, coord. 45°36'33"N, 15°25'11"E, mowed grassland with short grass and mosses, *Callunavulgaris*, *Juniperuscommunis*, *Pteridiumaquilinum*, 16 Oct 2010, Z. Tkalčec and I. Tkalčec (CNF 1/6057). Czech Republic • Brdské hřebeny Mts., Vižina, mossy forest meadow, 28 Sep 1965, E. Dlouhý (PRM610931, holotype of *D.emilii-dlouhyi*). France • Aveyron, Le Fel, Le Mas, coord. 44°39'27"N, 02°30'55"E, old grassland, 15 Nov 2016, C. Hannoire *CH16111509* (BBF, as *D.fuscobrunneum*); • Corse du Sud, Bastelica, pont de Zipitoli, coord. 41°57'38"N, 09°00'08"E, *Quercusilex* thicket on mineral-rich clay soil, 19 Nov 2014, P.-A. Moreau *PAM14111900* (LIP, as D.cf.atrocinereum); • Hautes-Pyrénées, Payolle, coord. 42°55'57"N, 00°16'03"E, acidophilic grazed grassland (*Nardion*), 15 Oct 2016, G. Corriol *GC16101502* (BBF, as *D.fuscobrunneum*); • Hautes-Pyrénées, Tramezaïgues, Fontaine des Usclats, coord. 42°47'32"N, 00°17'26"E, grazed grassland, 24 Sep 2018, C. Hannoire *CH18092415* (BBF, as *D.fuscobrunneum*); • Pas-de-Calais, Merlimont, Réserve biologique, coord. 50°26'52"N, 01°35'10"E, fixed dune, open grassland on sand, 10 Nov 2014, P.-A. Moreau *PAM14111009* (LIP, as D.cf.pseudocuneifolium). Germany • Gottesacker, Herrnhut, coord. 51°01'11"N, 14°44'53"E, ancient graveyard, 25 Aug 2016, A. Karich and R. Ullrich *IHI-16Der01* (GLM-F137754); • Niedersachsen, Meppen, Mepper Kuhweide, poor grassland on dry calcareous sand, 5 Nov 2015, E. Arnolds *Arnolds 15-95* (L, as *D.phaeopodium*); • Schleswig-Holstein, Winderatter See, 11 Nov 2015, T. Böhning *AG110* (ELTE). Italy • Marche, Cantiano (PU), Bosco di Tecchie, 17 Oct 2012, G. Consiglio, M. Maletti and L. Polidori *GC12181* (AMB 15102); • Sardinia, Tempio Pausania, Monti Avoni, 28 Dec 2008, M. Contu *28.XII.2008* (AQUI, as *D.fuscobrunneum*); • Savona, Sassello, Periaschi, 2 Nov 2013, F. Bocianolo *G3180* (GDOR). Romania • Cindrel Mts., pasture , 0.5 km SW of Poiana Sibiului, elev. 940–950 m, 45°47'52"N, 23°42'38"E, terrestrial, 26 Sep 2015, S. Jančovičová (SAV F-4702); • Gilau Mts., pasture 750 m E of Someşu Rece, elev. 500–530 m, coord. 46°43'21"N, 23°22'20"E, terrestrial, 4 Oct 2014, S. Adamčík (SAV F-4265). Slovakia • Považský Inovec Mts., 2 km SE of Kálnica, coord. 48°45'18"N, 17°56'55"E, elev. 300 m, terrestrial in semi-natural grassland, 24 Oct 2014, S. Jančovičová (SLO 674); • ibid., 24 Oct 2014, S. Jančovičová (SLO 675); • ibid., 24 Oct 2014, S. Jančovičová (SLO 676); • ibid., 24 Oct 2014, S. Jančovičová (SLO 677); • ibid., 24 Oct 2014, S. Jančovičová (SLO 678); • ibid., 24 Oct 2014, S. Jančovičová (SLO 679). Sweden • Småland, Femsjö par., “Haggårds tomter”, NW of Skattegårten, 6 Sep 1948, S. Lundell and G. Haglund (UPS-F-631060); • Småland, Femsjö par., Källebo, mossy grassland, 4 Sep 1948, S. Lundell and G. Haglund (UPS-F-631061); • Småland, Femsjö par., the slope east of Arvaberget towards Artvamaden, pasture, among *Hieraciumpilosella*, 20 Sep 1948, S. Lundell and G. Haglund (UPS-F-631063); • Småland, Femsjö par., Femsjö village, Rysslandsåkern, mossy grassland, 13 Sep 1948, S. Lundell and G. Haglund (UPS-F-631064). United Kingdom • England, Shropshire, Shrewsbury cemetery, coord. 52°41'46"N, 02°45'32"E, terrestrial in lawn, 21 Oct 2014, S. Adamčík (SAV F-4361); • Wales, Dugoed, 2 Nov 2004, G. W. Griffith *SH804522* (ABS); • Wales, Mynachdy, Brignant grassland, terrestrial, 26 Oct 2016, G. W. Griffith (SAV F-20076); • Wales, Montgomery, Newtown cemetery, coord. 52°31'06"N, 03°18'06"E, terrestrial in lawn, 24 Oct 2014, P. David (SAV F-4393); • ibid., 24 Oct 2014, D. Harries (SAV F-4394); • ibid., 24 Oct 2014, D. Harries (SAV F-4395); • Wales, Powys, Gregynog grounds, coord. 52°34'04"N, 03°21'06"E, terrestrial in lawn, 24 Oct 2014, D. Harries (SAV F-4396); • ibid., 24 Oct 2014, D. Harries (SAV F-4397); • Wales, Powys, Hay Common, coord. 52°02'34"N, 03°06'19"E, terrestrial in semi-natural grassland, 28 Oct 2014, S. Adamčík (SAV F-4435); • ibid., 28 Oct 2014, D. Harries (SAV F-4436); • ibid., 28 Oct 2014, D. Harries (SAV F-4437); • ibid., 28 Oct 2014, D. Harries (SAV F-4438); • Wales, Pembrokeshire, Somerton farm, coord. 51°39'50"N, 04°59'29"E, terrestrial in pasture, 25 Oct 2014, S. Adamčík (SAV F-4407); • ibid., 25 Oct 2014, D. Harries (SAV F-4408); • Wales, Pembrokeshire, Tufton cemetery, coord. 51°55'02"N, 04°51'07"E, terrestrial in lawn, 27 Oct 2014, S. Adamčík (SAV F-4423); • ibid., 27 Oct 2014, leg. S. Adamčík (SAV F-4425).

###### Notes.

*Dermolomacuneifolium* has inamyloid spores as a member of D.subgenusDermoloma, section Dermoloma. The section forms a crown clade in the *Dermoloma* phylogeny with short branch lengths at species rank clades indicating short genetic distances (Fig. 2). Morphologically, *D.cuneifolium* exhibits intermediate characters within this group of very similar species. It has intermediate basidiomata sizes, spores which are relatively small but similar like in several other related species. It is similar, especially to *D.simile*, *D.huartii* and *D.intermedium*. Here, and in similar cases, we strongly suggest to use the key for identification, in combination with the heatmap diagram (Fig. 4), chromatogram (Fig. 6), plot diagrams (Figs 5–7) and other supplementary files (Suppl. materials 6, 7, 9) for the best score when combining multiple morphological characters. We believe that the probability of correct identification of this species may be high, but for precise identification we recommend to use sequences of the ITS region. The sister species to *D.cuneifolium* is the North American *D.fumosidiscum* (Fig. 2), which is distinctly smaller and has strongly rugulose pileus center. This species was included in the phylogenetic study by Sánchez-García et al. (2021) and the name was assigned by the position of the neotype sequence. It is the type species of *Dermoloma*.

##### ﻿ Dermoloma curvicystidiatum

Taxon classificationAnimaliaAgaricalesTricholomataceae

Mešić, Tkalčec, Brandrud & Dima sp. nov.

F926496E-56EA-58B3-B787-64F6E59A41CC

856335

Figs 23a, b , 24

###### Etymology.

The epithet refers to the flexuous caulocystidia and marginal cells.

###### Holotype.

Germany • Rheinland-Pfalz, Horbach, elev. 370 m, coord. 49°49'44"N, 07°31'17"E, terrestrial in semi-natural grassland, 8 Nov 2019, S. Adamčík (SAV F-20504).

###### Diagnosis.

European species; basidiomata collybioid, moderately large, brown to dark brown on all parts; pilei becoming ochraceous- brown when dry; spores amyloid, on average > 6.5 µm long and > 4 µm wide; marginal cells and caulocystidia usually flexuous and nodulose, occasionally with projections.

***Pileus*** (7–)10–22 mm; broadly conical to convex, indistinctly umbonate; margin translucently striate to half of the radius; surface smooth near margin, rugulose or rough near center, hygrophanous; color near margin dark brown (6F7), when dry light brown (5D4), near center dark brown to almost black (6F4, 6F7, 7F4), when dry brownish ochraceous (5C4). ***Stipe*** (23–)32–44 × 1–3 mm; cylindrical, flexuous; surface finely longitudinally striate, pruinose near lamellae, glabrous towards the base; color near lamellae brown (6E4) to dark brown (6F4, 6F6), near the base dark brown (6F4, 6F8). ***Lamellae*** L = 17–37, l = 1–3; 3–4 mm wide; adnexed to adnate-emarginate; color brown (6E3, 6E4), old dark brown (6F5); edges entire. ***Context*** elastic; odor farinaceous.

**Figure 23.**
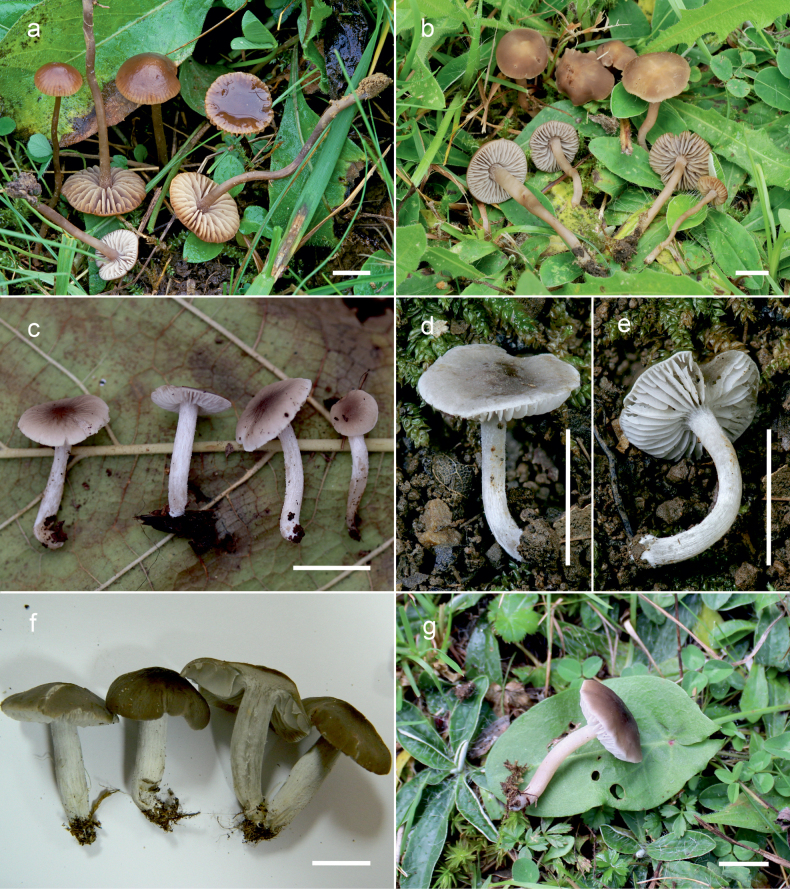
Basidiomata of *Dermoloma* in field appearance. **a**. *Dermolomacurvicystidiatum* (CNF 1/1963), photo A. Mešić; **b**. *Dermolomacurvicystidiatum* (GLM-F137758), photo A. Karich; **c**. *Dermolomafumosidiscum* (TENN-F-076389, holotype), photo M. G. Sánchez; **d, e***Dermolomafumosidiscum* (TENN-F-076330), photo J. Kalichman; **f***Dermolomafuscobrunneum* [LIP (*PAM14111008*), epitype], photo P.-A. Moreau; **g***Dermolomafuscobrunneum* (SAV F-22210), photo S. Jančovičová. Scale bar: 10 mm.

**Figure 24.**
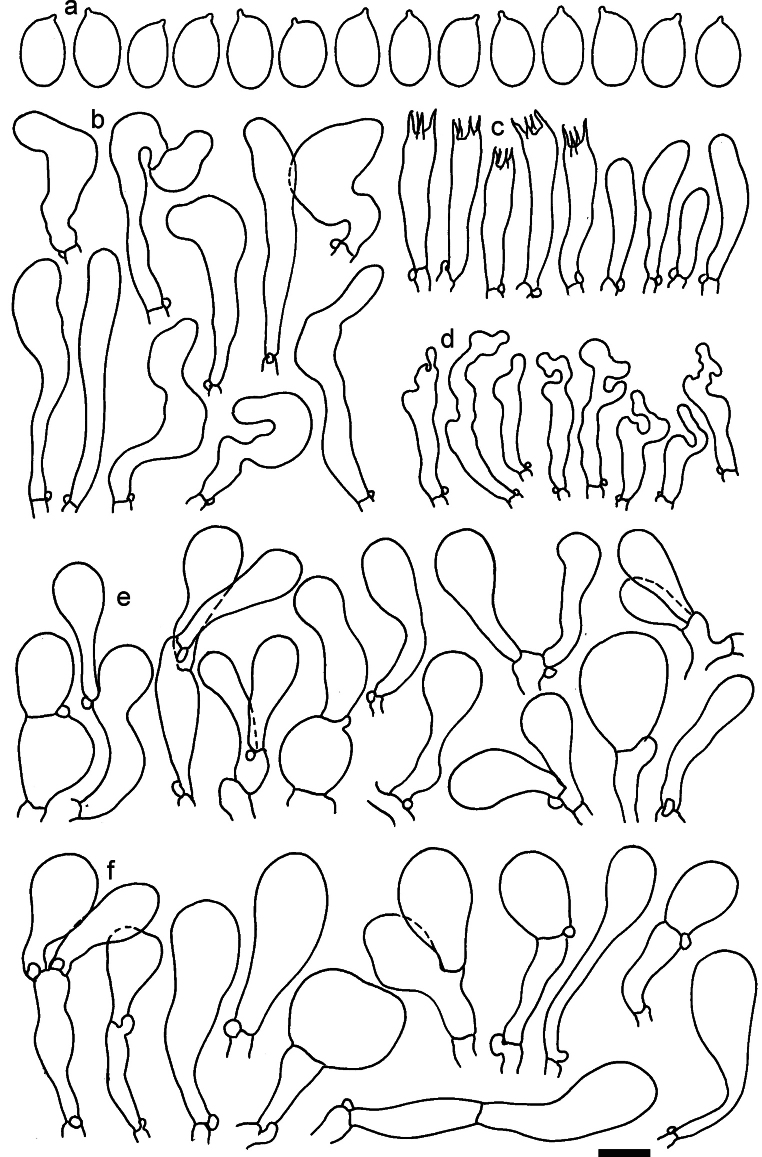
*Dermolomacurvicystidiatum* (SAV F-20504, holotype), microscopic elements. **a** Spores; **b** caulocystidia; **c** basidia and basidioles; **d** marginal cells; **e** pileipellis elements near the pileus margin; **f** pileipellis elements near the pileus center. Scale bar: 5 µm for spores and 10 µm for other elements.

***Spores*** (6–)6.3–6.7–7.1(–7.7) × (3.8–)4.1–4.3–4.6(–4.8) μm; narrowly ellipsoid to oblong, Q = (1.38–)1.45–1.55–1.64(–1.75); walls amyloid; hilar appendage 0.5–1 μm long. ***Basidia*** (22–)26–29.2–32.5(–34) × (5.5–)6.5–6.8–7.5(–8) μm; clavate; with 4 sterigmata. ***Basidioles*** first cylindrical, then clavate, ca. 4–7 μm wide. ***Marginal cells*** (18–)22.5–26.9–31.5(–38) × (3–)4–5.5–6.5(–8.5) μm; cylindrical or lageniform, rarely clavate, often very flexuous and nodulose, occasionally diverticulate or with lateral projections. ***Pileipellis*** 50–65 μm deep; suprapellis 33–43 μm deep, usually of one to three layers of inflated, loosely arranged cells; subpellis hardly defined, 20–30 μm deep, of densely packed, puzzled, 3–10(–15) μm wide, almost horizontally oriented hyphae and gradually passing to horizontally oriented hyphae in trama; hyphal terminations with brownish yellow to dark brown parietal pigments, and locally also with incrusted pigments in subpellis near the center, walls thickened up to 0.5 μm. Terminal cells near pileus margin (15–)27.5–37.8–48(–65) × (8–)12–16.8–21.5(–34) μm; usually obpyriform, sphaeropedunculate or clavate; subterminal cells occasionally branched, mainly fusiform and ventricose, often with lateral swellings. Terminal cells near pileus center (12–)26.5–35.5–44.5(–56) × (11–)13.5–16.7–20(–25) μm; similar to cells near margin but occasionally also ellipsoid; subterminal cells usually narrower, narrowly cylindrical or fusiform, often branched, occasionally irregularly lobate. ***Caulocystidia*** (25–)31–46.5–62(–102) × (3.5)5–8.3–11.5(–17) μm; very variable in shape and size, some broadly clavate or sphaeropedunculate, others filiform and with long attenuated tips, near lamellae also flexuous-filiform and similar to marginal cells, often flexuous, sometimes lobate, in clusters or occasionally also individual, repent or ascending; usually with slightly (0.5 m) thickened walls but near septa and on subterminal cells up to 1 μm, with brownish yellow, parietal pigments. ***Clamp connections*** present.

###### Distribution and ecology.

Known from temperate-boreal areas in, Croatia, Finland, Germany and Norway; in semi-natural grasslands on calcareous soils, once in a calcareous, thermophilous deciduous forest.

###### Additional material studied.

Croatia • Zagreb, Črnomerec, coord. 45°50'04"N, 15°57'03"E, mowed grassland with short grass and mosses, 22 Oct 1998, A. Mešić (CNF 5/269); • Zagreb, Črnomerec, coord. 45°50'01"N, 15°56'56"E, grassland with short grass and mosses, 27 Oct 2000, A. Mešić (CNF 1/1963); • Zagreb, Črnomerec, coord. 45°49'59"N, 15°56'58"E, grassland mowed twice a year, 25 Oct 2012, Z. Tkalčec (CNF 1/6491). Finland • Åland, Mariehamn, W of Dalen, near the road to Espholmen, coord. 60°04'08.4"N, 19°57'36.0"E, herb-rich forest with *Acerplatanoides*, *Betula* sp., *Corylusavellana*, *Fraxinusexcelsior*, *Piceaabies*, on calcareous ground, 21 Aug 2000, J. Vauras *FIPUT185-14* (TUR140054). Germany • Lückendorf, Kurwiese, coord. 50°49'53"N, 14°45'29"E, mesophilic semi-arid meadow (park-lawn), regularly mown, 1 Sep 2021, A. Karich and R. Ullrich *IHI-21Der01* (GLM-F137758, as *D.pseudocuneifolium*). Norway • Oppland, Lunner, Kjørvensætra, in semi-natural grazed pasture on calcareous soil, Aug 2015, T. E. Brandrud *TEB061-15* (O); • ibid., 10 Aug 2016, T. E. Brandrud and B. Dima *DB6031* (ELTE), duplicate *TEB157-16* (O); • Vestland, Bømlo, Spyssøya, Myra, coord. 59°43'44"N, 5°22'01"E, in semi-natural grassland on calcareous soil, 7 Oct 2022, J. B. Jordal, P. Fadnes and A. H. Abaz *NOBAS10390-23* (O-F-259833).

###### Notes.

*Dermolomacurvicystidiatum* is a member of D.subgenusAmylospora, section Atrobrunnea. It belongs to a larger clade of species with mainly collyboid basidiomata (see notes under *D.confusum*). In the field it is very difficult to distinguish it from other members of this clade. It is well-defined by combination of the darker brown colors of basidiomata and the larger spores. Very closely related are *D.pruinosipes* and D.aff.pruinosipes, of which the former has a more distinctly pruinose stipe and the latter with marginal cells that are not nodulose and flexuous. *Dermolomacurvicystidiatum* is for the first time included in a phylogeny in this study.

##### ﻿ Dermoloma fumosidiscum

Taxon classificationAnimaliaAgaricalesTricholomataceae

Adamčík & Matheny sp. nov.

4EAD3886-9138-56C7-94A7-094F5EAF3FD9

856336

Figs 23c–e , 25

###### Etymology.

In reference to the dark center of the pileus.

###### Holotype.

USA • Tennessee, Great Smoky Mountains National Park, Blount Co., Little Pigeon river, right riverbank, elev. 600 m, coord. 35°42'28"N, 83°22'57"E, terrestrial under *Acerrubrum*, *Liliodendrontulipifera*, *Liquidambar* sp. and *Quercus* sp., 2 Nov 2013, S. Adamčík (holotype TENN-F-071041, isotype SAV F-4094).

###### Diagnosis.

North American species; pilei up to 12 mm, rugulose near center; spores inamyloid.

***Pileus*** 7–12 mm; convex, indistinctly umbonate; margin not striate; surface rough near margin, strongly radially rugulose and veined, hygrophanous; color near margin brownish ochraceous (5C3) and with almost white outline, when dry brownish gray (6C2) to grayish brown (6D3), near center dark brown (6F3), when dry grayish brown (6E3). ***Stipe*** 17–22 × 2 mm; cylindrical, sometimes narrowed towards the base and curved; surface finely longitudinally striate, finely granulose near lamellae, towards the base fibrillose; color near lamellae white, near the base brownish-grayish. ***Lamellae*** L = 22–28, l = 1–3; up to 2.5 mm wide; adnate-emarginate and decurrent with tooth; color yellowish gray (4B2); edges irregularly serrulate. ***Context*** when young elastic, later fragile; odor farinaceous.

***Spores*** (4.8–)4.9–5.2–5.5(–5.8) × (3.5–)3.6–3.8–4(–4.2) μm; ellipsoid, Q = (1.26–)1.33–1.38–1.43(–1.48); walls inamyloid, sometimes thick-walled and dextrinoid; hilar appendage 0.5–1 μm long. ***Basidia*** (18–)20–22.6–25(–28) × 5.5–5.9–6.5(–7) μm; clavate; with 4 sterigmata. ***Basidioles*** first cylindrical, then clavate, ca. 3–6.5 μm wide. ***Marginal cells*** (11–)13.5–16.7–20(–22) × (4.5–)5–5.6–6(–6.5) μm; similar to basidioles, mainly clavate. ***Pileipellis*** 42–52 μm deep; suprapellis 32–38 μm deep, mainly of one layer of densely arranged inflated cells, gradually passing to 15–20 μm deep subpellis of densely packed, intricate, horizontally oriented, 3­–10 μm wide hyphae, not sharply delimited from horizontally oriented hyphae in trama; hyphal terminations with brownish yellow parietal pigments, thin-walled or occasionally thickened up to 1 μm, rarely with brownish incrusted pigments near septa of terminal cells. Terminal cells near pileus margin (23–)30–38.2–46.5(–52) × (12–)15–19.6–24(–29) μm; usually sphaeropedunculate, sometimes obpyriform or clavate; subterminal cells usually narrower and implemented in subpellis, often branched, rarely inflated and with lateral swellings. Terminal cells near pileus center (27–)33–43.2–53(–64) × (11–)15–18.3–22(–26) μm; mainly clavate, occasionally obpyriform or sphaeropedunculate, rarely ellipsoid, towards bases occasionally flexuous or lobate; subterminal cells similar to cells near margin. ***Caulocystidia*** (13–)20.5–28.2–36(–44) × (3.5–)4.5–5.4–6.5(–8) μm; clavate or subcylindrical, usually not or only slightly flexuous, often individual or in small fascicules, repent or ascending; thin-walled, with yellow parietal pigments and dispersed crystalline or granulose yellow incrustations. ***Clamp connections*** present.

**Figure 25.**
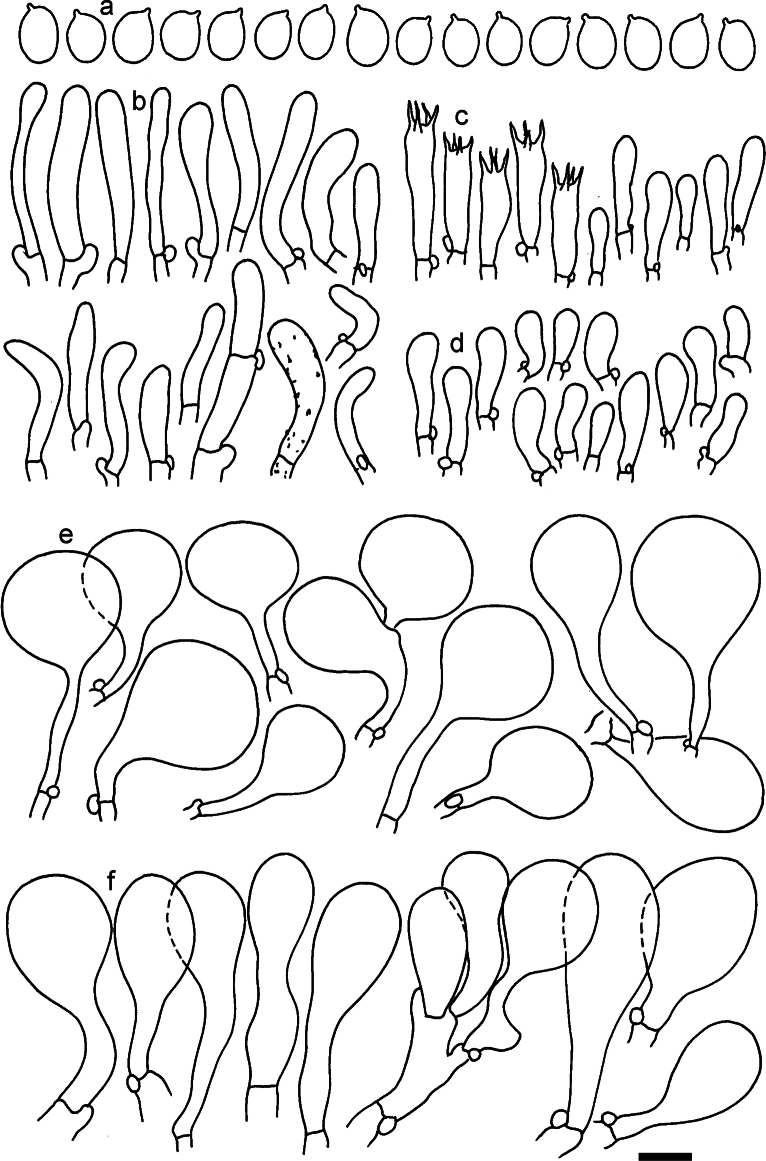
*Dermolomafumosidiscum* (SAV F-4094, isotype), microscopic elements. **a** Spores; **b** caulocystidia; **c** basidia and basidioles; **d** marginal cells; **e** pileipellis elements near the pileus margin; **f** pileipellis elements near the pileus center. Scale bar: 5 µm for spores and 10 µm for other elements.

###### Distribution and ecology.

Known from three localities in Smoky Mts., Tennessee, USA; in deciduous forests, two collections from park lawn areas.

###### Additional material studied.

USA • Tennessee, Great Smoky Mountains National Park, Oak Ridge, Haw Ridge Park, coord. 36°00'38"N, 84°09'46"E, terrestrial under *Acer* sp., *Carpinus* sp., *Cerasus* sp., *Liliodendrontulipifera* and *Quercus* sp., 10 Oct 2021, J. Kalichman *JK079* (TENN-F-076330); • Tennessee, Union County, Big Ridge State Park, 36°00'58"N, 83°58'54"E, in yard under hardwoods, *Acer* sp., *Carya* sp., *Cornus* sp., *Quercus* sp., 23 Oct 2021, J. Kalichman *JK139* (TENN-F-076389).

###### Notes.

*Dermolomafumosidiscum* is a North American species with inamyloid spores which belongs to D.subgenusDermoloma, section Dermoloma. It is sister to the type species of the genus, *D.cuneifolium* (Fig. 2). Unlike European members of section Dermoloma, this species is easily distinguishable by the combination of very small basidiomata and a strongly rugulose pileus center. Fortunately, this allows identification without a microscope, because the species does not have any unique microscopic characters distinguishing it from *D.cuneifolium* or other similar species. The sequence from the type of *D.fumosidiscum* was included in the phylogeny by Sánchez-García and Matheny (2017) as *Dermoloma* sp. and later in Sánchez-García et al. (2021) as “D.cf.cuneifolium”.

##### ﻿ Dermoloma fuscobrunneum

Taxon classificationAnimaliaAgaricalesTricholomataceae

P.D. Orton, Notes R. bot. Gdn. Edinb. 38(2): 326. 1980.

E5BF9F8C-20A2-5C92-A881-759EF0394A6B

111399

Figs 23f, g , 26

###### Holotype.

United Kingdom • England, Somerset, Bickham Wood, Crawley, in locis graminosis vel in silvis, 24 Oct 1975, P. D. Orton *4735* (E16876).

###### Epitype

(designated here MBT 10022913): France • Pas-de-Calais, Ambleteuse, Pré communal, coord. 50°48'28"N, 01°37'40"E, pastured grassland on sand, 10 Nov 2014, D. Huart and P.-A. Moreau *PAM14111008* (LIP).

###### Distinguishing characters.

European species; basidiomata medium sized; pileus margin not striate; lamellae and stipes ochraceous-gray to white; spores inamyloid; caulocystidia wider than 5.5 µm.

***Pileus*** 10–25 mm; convex, indistinctly umbonate; margin not striate; surface near margin smooth, near center radially rugulose or wrinkled, hygrophanous; color when young dark brown (6F4), when mature near margin brown (6E3), when dry grayish brown (6D3), near center dark brown (6F4), when dry grayish brown (6E3). ***Stipe*** 20–30(–50) × 2.5–4(–8) mm; cylindrical to slightly fusiform, narrowed towards the base, usually flexuous especially near the base; surface finely longitudinally striate, finely granulose near lamellae, finely fibrillose towards the base; color ochraceous-gray (5B2) to almost white. ***Lamellae*** L = 20–42, l = 3; up to 5 mm wide; adnate-emarginate and indistinctly decurrent with tooth; color ochraceous-gray (5B2) to almost white; edges entire or slightly irregular, rarely serrulate. ***Context*** when young elastic, later fragile; odor farinaceous.

**Figure 26.**
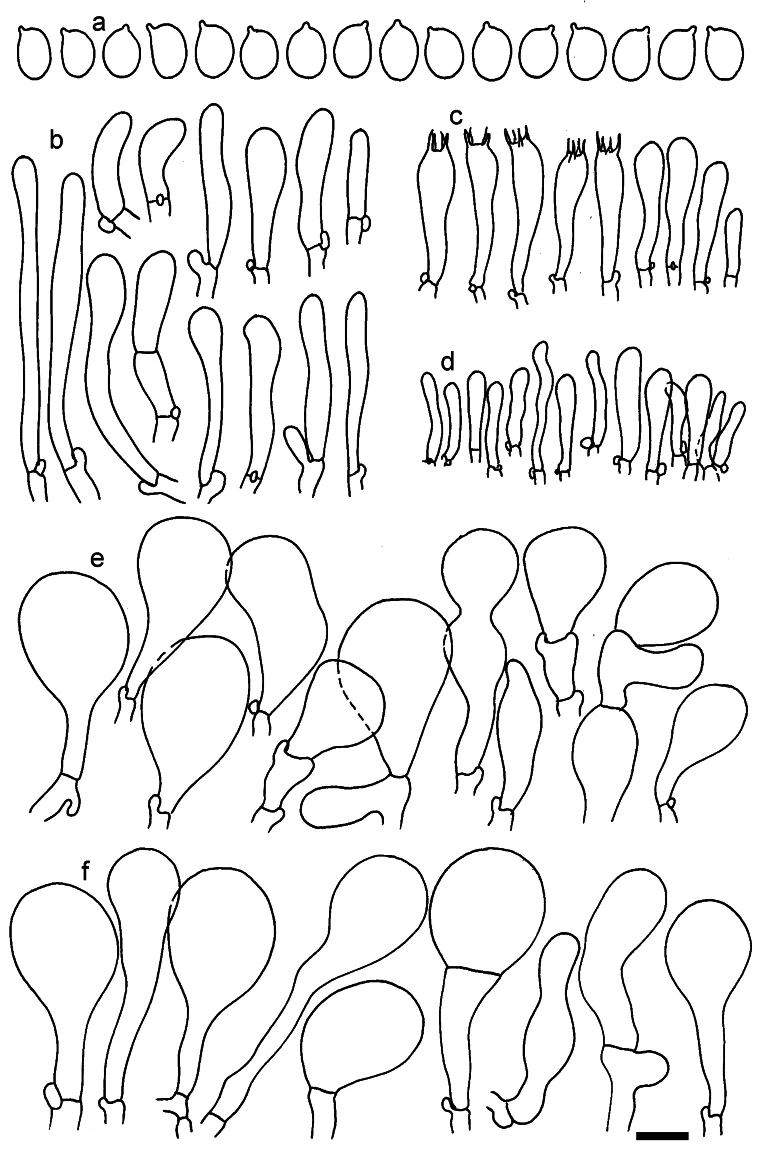
*Dermolomafuscobrunneum* (SAV F-3860), microscopic elements. **a** Spores; **b** caulocystidia; **c** basidia and basidioles; **d** marginal cells; **e** pileipellis elements near the pileus margin; **f** pileipellis elements near the pileus center. Scale bar: 5 µm for spores and 10 µm for other elements.

***Spores*** (4.5–)4.9–5.2–5.5(–6.1) × (3.2–)3.6–3.8–4.1(–4.7) μm; broadly ellipsoid to ellipsoid, Q = (1.21–)1.28–1.35–1.42(–1.53); walls inamyloid; hilar appendage ca. 0.5–1.5 μm long. ***Basidia*** (20–)23.5–26.1–29(–31) × (6–)6.5–6.8–7(–8) μm; clavate; with 4 sterigmata. ***Basidioles*** first cylindrical, then clavate, ca. 3–7.5 μm wide. ***Marginal cells*** (13–)15–18.2–21.5(–26) × 2.5–3.6–4.5(–5.5) μm; not well-differentiated, cylindrical or clavate, often flexuous. ***Pileipellis*** 65–76 μm deep; suprapellis of mainly one layer of inflated cells; subpellis 24–27 μm deep, hardly differentiated, of densely packed, irregularly oriented, puzzled, 3–12 μm wide hyphae, not sharply delimited from horizontally oriented hyphae in trama; hyphal terminations with brown parietal pigments, thin-walled or occasionally thickened up to 1 μm and with dark incrusted pigments especially near septa of terminal cells and in subpellis. Terminal cells near pileus margin (22–)30.5–38.4–46(–56) × (9–)16–20.4–24.5(–33) μm; usually obpyriform, sphaeropedunculate or clavate, sometimes ellipsoid; subterminal cells usually narrower and implemented into intricate hyphae of subpellis, sometimes with lateral swellings. Terminal cells near pileus center (16–)26–36–46(–54) × (8–)12–17–22(–31) μm; similar to cells near margin; subterminal cells usually narrower, occasionally with lateral swellings or irregularly lobate. ***Caulocystidia*** (19.5–)25.5–37.3–49(–69) × (3–)5–7.1–9(–12) μm; usually clavate, sometimes cylindrical, usually not flexuous, often clustered in small ascending fascicules, sometimes individual and repent; usually with slightly thickened walls up to 0.5 μm, often with crystalline or granulose yellow incrustations. ***Clamp connections*** present.

###### Distribution and ecology.

Known from four localities in France, Slovakia, Spain and United Kingdom; in grasslands or forests; habitat preferences insufficiently known.

###### Additional material studied.

Slovakia • Podbeskydská vrchovina Mts., NW margin of the village Zákamenné, terrestrial in a meadow, 11 Oct 2012, V. Kučera (SAV F-3860). Spain • Pyrénées Mts., Canfranc, Río de la Canal Roya, coord. 42°46'26"N, 00°30'56"E, terrestrial, under *Buxus* sp. and *Pinussylvestris*, 3 Oct 2022, S. Adamčík (SAV F-22210).

###### Notes.

*Dermolomafuscobrunneum* is a member of D.subgenusDermoloma, section Dermoloma. It has relatively small basidiomata which makes it similar to *D.carpathicum* and *D.simile*. It is distinguished from them by the paler or almost white lamellae and stipe, and the caulocystidia wider than 5.5 μm. The identification of species in D.sectionDermoloma can be problematic and requires special attention and multisource data (see notes under *D.cuneifolium*). *Dermolomafuscobrunneum* was included in the phylogenetic study by Sánchez-García et al. (2021) as “D.cf.cuneifolium”. The species was originally distinguished from other species with inamyloid spores by prevailingly brown and not gray colors (Orton 1980), however, our study revealed low taxonomic significance of pileus colors in D.subgenusDermoloma (Fig. 6). Sequencing of the holotype specimen failed, but our morphological analysis of the type specimen confirmed that *D.fuscobrunneum* has inamyloid spores on av. 4.6 × 3.5 μm in size. The small spores serve to distinguish *D.fuscobrunneum* from *D.atrocinereum*, *D.bellerianum*, D.aff.bellerianum, *D.fusipes*, D.aff.fusipes, *D.huartii* and *D.intermedium* (Fig. 7). *Dermolomasimile* has similar spores but smaller basidiomata. Among the studied species, *D.cuneifolium* and *D.carpathicum* have similar spores and basidiomata sizes, but we decided to assign the name *D.fuscobrunneum* to a phylogenetic species previously labelled as “D.aff.cuneifolium 2”, which has the colors most similar to those described in the protologue. No recent material from the country of the type origin (United Kingdom) was available, therefore we selected a collection from northwest France as the epitype. The original description (Orton 1980) mentioned grassy places as ecology of the type collection, but later in the description he wrote for this specimen: “In mixed deciduous wood of recent origin”.

##### ﻿ Dermoloma fusipes

Taxon classificationAnimaliaAgaricalesTricholomataceae

Arnolds & Harries sp. nov.

C80CF55C-A8FE-53DF-B97B-C449AE6CEE81

856338

Figs 27a, b , 28

###### Etymology.

The stipes of mature basidiomata are distinctly fusiform.

###### Holotype.

United Kingdom • Wales, Powis Castle gardens, coord. 52°38'58"N, 03°09'34"E, terrestrial in lawn, 22 Oct 2014, D. Harries (SAV F-4377).

###### Diagnosis.

European species; basidiomata relatively large; pilei 23–37 mm in diameter, not hygrophanous; stipes 3.5–7.5 mm wide, fusiform and narrowed at bases; spores inamyloid, on average narrower than 4 μm.

***Pileus*** (11–)23–37(–45) mm; convex, later almost plane, often indistinctly umbonate, lobate; margin not striate or translucently striate up to 10 mm; surface smooth, sometimes radially slightly rugulose or rough near center, not hygrophanous; color near margin first grayish brown (6E3), later light brown (5D4), near center dark brown (6F3, 6F4 to almost black), when old brown (5E4). ***Stipe*** (10–)20–58(–66) × (2–)3.5–7.5(–8) mm; cylindrical or fusiform, narrowed towards the base, usually flexuous especially near the base; surface longitudinally striate, granulose or pruinose near lamellae, fibrillose to finely squamulose towards the base; color near lamellae almost white to ochraceous-gray (5B2), near the base brownish ochraceous (5C3) to brownish gray (6C2), sometimes with darker fibrils on paler background. ***Lamellae*** L = (26–)31–41(–43), l = 1–3; 4–10 mm wide; adnexed or adnate-emarginate and decurrent with tooth; color ochraceous-gray (5B2), brownish gray (5C2) to brownish ochraceous (6C3), paler towards edges; edges entire or slightly serrulate. ***Context*** fragile; odor farinaceous.

**Figure 27.**
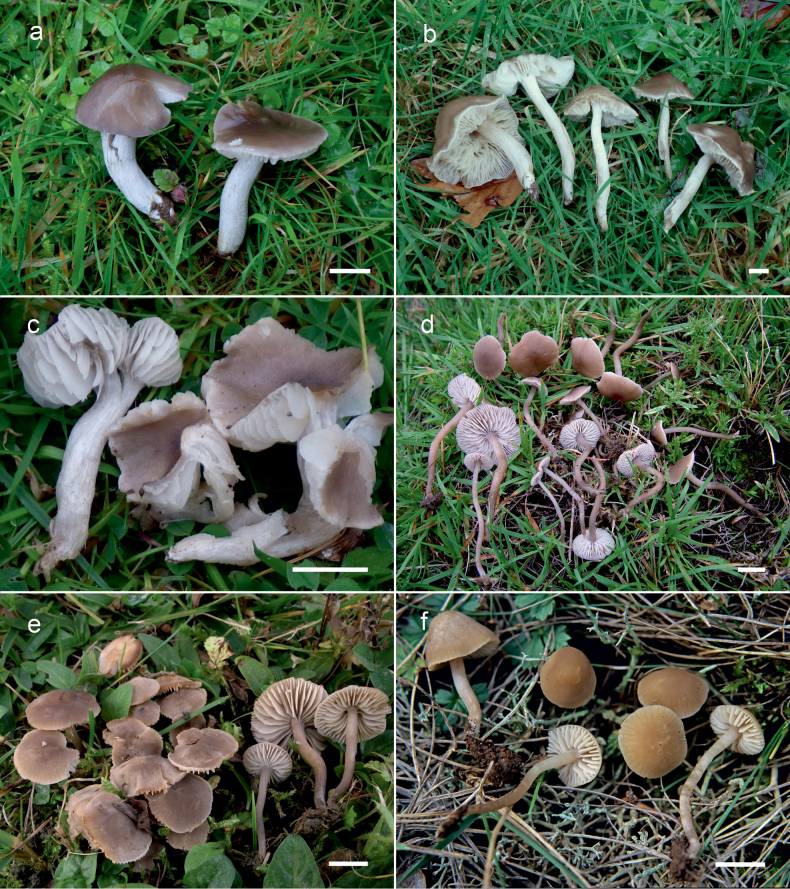
Basidiomata of *Dermoloma* in field appearance. **a***Dermolomafusipes* (SAV F-4376), photo D. Harries; **b***Dermolomafusipes* (SAV F-4377, holotype), photo D. Harries; **c**Dermolomaaff.fusipes (SAV F-4371), photo D. Harries; **d***Dermolomagriseobasale* (SAV F-4723, holotype), photo S. Jančovičová; **e***Dermolomagriseobasale* [BBF (*GC10110201*)], photo G. Corriol; **f***Dermolomagriseobasale* (CNF 5/287), photo A. Mešić. Scale bar: 10 mm.

**Figure 28.**
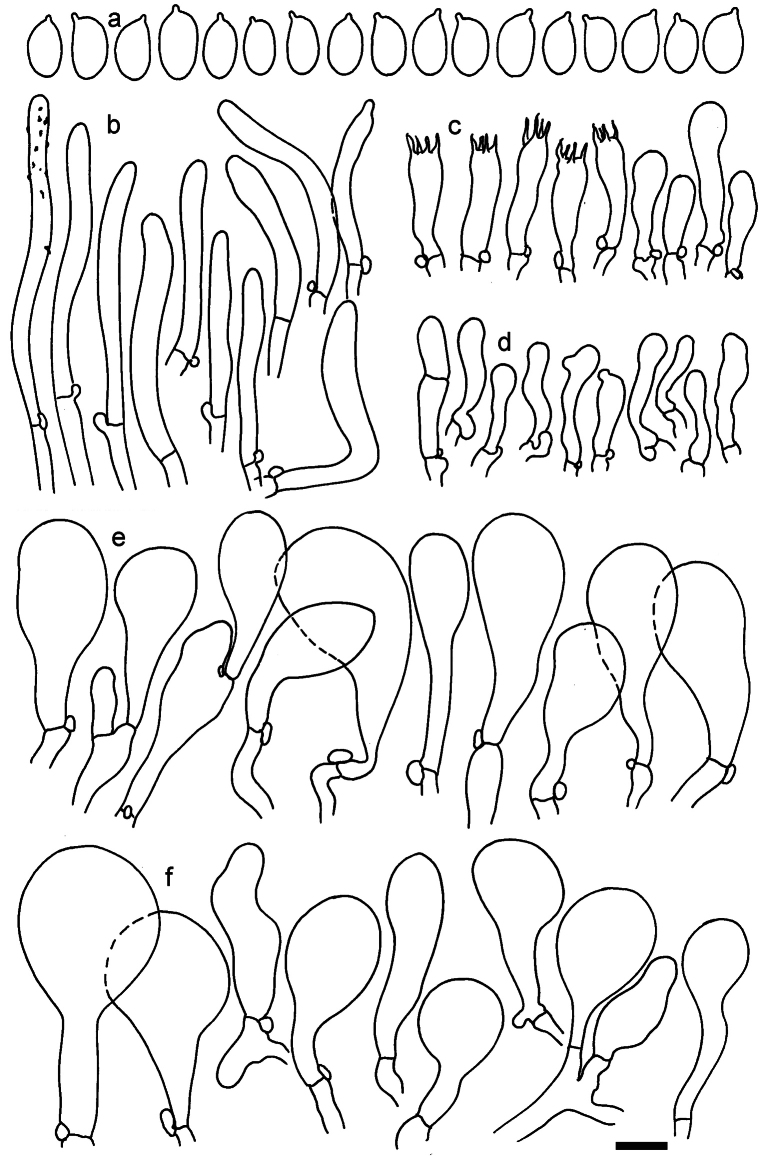
*Dermolomafusipes* (SAV F-4377, holotype), microscopic elements. **a** Spores; **b** caulocystidia; **c** basidia and basidioles; **d** marginal cells; **e** pileipellis elements near the pileus margin; **f** pileipellis elements near the pileus center. Scale bar: 5 µm for spores and 10 µm for other elements.

***Spores*** (4.5–)5.2–5.7–6.2(–7.2) × (2.5–)3.4–3.8–4.1(–4.5) μm; ellipsoid to narrowly ellipsoid, Q = (1.30–)1.41–1.52–1.64(–1.82); walls inamyloid; hilar appendage ca. 0.5–1.5 μm long. ***Basidia*** (19–)23.5–27.1–30.5(–34) × (5.5–)6–6.8–7.5(–8) μm; clavate; with 4 sterigmata. ***Basidioles*** first cylindrical, then clavate, ca. 3.5–7.5 μm wide. ***Marginal cells*** (11–)13.6–20.1–26.7(–35) × (4–)4.5–5.3–6(–8) μm; not well-differentiated, clavate, sometimes diverticulate or lobate, flexuous. ***Pileipellis*** 65–80 μm deep; suprapellis 40–60 μm deep, usually of two or three layers of inflated, densely arranged cells; subpellis well-differentiated, 25–35 μm deep, of densely packed, irregularly oriented, puzzled, 3–10 μm wide hyphae, gradually passing to horizontally oriented hyphae in trama; hyphal terminations with brown parietal pigments, near septa of terminal cells and on subterminal cells with dark brown incrusted pigments, walls thickened especially in subpellis up to 1 μm. Terminal cells near pileus margin (20–)28.5–39.5–50.5(–75) × (7–)12.5–17.5–22.5(–30) μm; usually obpyriform, sphaeropedunculate or clavate; subterminal cells usually inflated but smaller, lobate. Terminal cells near pileus center (18–)30–39.3–49(–68) × (7.5–)13.5–18.5–23.5(–31) μm; similar to cells near margin; subterminal cells less frequently inflated, and with more conspicuous dark brown to black incrustations than near margin. ***Caulocystidia*** (12.5–)22–32.7–43.2(–62.4) × (2–)3.5–4.7–6(–10.5) μm; cylindrical or clavate, usually not or only slightly flexuous, often clustered in small ascending fascicules, sometimes individual and repent; usually with slightly thickened walls up to 0.5 μm, sometimes with scarce yellow incrustations. ***Clamp connections*** present.

###### Distribution and ecology.

Known from three localities in The Netherlands, Slovakia and Wales (United Kingdom); in semi-natural grasslands; habitat preference insufficiently known.

###### Additional material studied.

The Netherlands • Elsloo, along Julianakanaal, 30 Oct 1982, J. Schreurs (L3988083, as D.cuneifoliumvar.punctipes). Slovakia • Bolešov, chata Gilianka, coord. 49°00'19"N, 18°08'20"E, terrestrial in semi-natural grassland, 7 Oct 2013, S. Jančovičová (SLO 2015). United Kingdom • Wales, Powis Castle gardens, coord. 52°38'58"N, 03°09'34"E, terrestrial in lawn, 22 Oct 2014, D. Harries (SAV F-4376).

###### Notes.

*Dermolomafusipes* is a member of D.subgenusDermoloma, section Dermoloma. It has relatively large basidiomata similar to *D.cuneifolium*, *D.huartii* and *D.intermedium*. The identification of species in D.sectionDermoloma can be problematic and requires special attention and multisource data (see notes under *D.cuneifolium*). *Dermolomafusipes* was included in the phylogenetic study by Sánchez-García et al. (2021) as “*Dermoloma* sp. 4”. Sequences from French (CL/F04.249) and Croatian (CLF 1/2851) samples were of poor quality. They may belong to this species or to D.aff.fusipes described below, but they were not used in our morphological study.

##### ﻿ Dermoloma aff. fusipes

Taxon classificationAnimaliaAgaricalesTricholomataceae

AE78E9E0-7060-5408-964F-01F2EFE4F9F6

Figs 27c , 29

###### Description.

***Pileus*** 25–26 mm; convex, soon expanding to plane, indistinctly umbonate; margin not striate, recurved when old, when dry radially cracking; surface smooth, slightly rough near center, not hygrophanous; color near margin grayish brown (more reddish than 5D3), near center dark brown (6F3). ***Stipe*** 24–35 × 4.5–5 mm; cylindrical, narrowed towards the base, flexuous; surface finely longitudinally striate, pruinose near lamellae, fibrillose-granulose towards the base; color near lamellae ochraceous-gray (5B2) to almost white, near the base brownish gray (5C2) with slightly more brown granules. ***Lamellae*** L = 25–36, l = 1–3; 4.5 mm wide; adnate-emarginate and decurrent with tooth; color ochraceous-gray (5B2) to almost white towards edges; edges entire. ***Context*** fragile; odor farinaceous.

***Spores*** (5–)5.2–5.6–5.9(–6.2) × (3.3–)3.5–3.8–4.1(–4.3) μm; ellipsoid to narrowly ellipsoid, Q = (1.32–)1.38–1.47–1.55(–1.61); walls inamyloid, often thick-walled and dextrinoid; hilar appendage ca. 0.5–1.5 μm long. ***Basidia*** (24–)26.5–28.5–30.6(–32) × 7–7.6–8(–9) μm; clavate; mainly with 4 sterigmata, occasionally with 2 sterigmata. ***Basidioles*** first cylindrical, then clavate, ca. 3–7 μm wide. ***Marginal cells*** not differentiated and similar to basidioles, mainly clavate. ***Pileipellis*** 68–78 μm deep; suprapellis 45–58 μm deep, of two to four layers of inflated, densely arranged cells; subpellis hardly defined, 12–25 μm deep, of densely packed, mainly horizontally oriented, puzzled, 3–12 μm wide hyphae, gradually passing to horizontally oriented hyphae in trama; hyphal terminations with brownish yellow parietal pigments, near septa of terminal cells and in subpellis also indistinctly incrusted but without darker pigments, near pileus center pigments in subpellis more apparently incrusted and darker, thin-walled or with slightly thickened walls up to 0.5 μm, in subpellis thick-walled up to 1 μm. Terminal cells near pileus margin (26–)34–44.5–55(–66) × (10–)15–19.3–23.5(–26) μm; usually sphaeropedunculate, obpyriform or clavate; subterminal cells usually equally wide and inflated, ventricose-fusiform, rarely narrowly cylindrical, often branched and irregularly lobate, occasionally nodulose and with lateral projections. Terminal cells near pileus center (30–)37–46.3–55.5(–71) × (10–)15.8–20.7–25.5(–30) μm; more frequently narrow and subcylindrical, more irregularly lobate and more frequently branched; subterminal cells more frequently narrowly cylindrical and branched, often flexuous and lobate, occasionally also inflated. ***Caulocystidia*** (12–)21.5–30–37.5(–48.5) × (3.5–)4.5–5.1–6(–6.5) μm; cylindrical or clavate, usually flexuous, often clustered in loose, small or large, ascending fascicules, sometimes individual and repent; thin-walled or with slightly thickened walls near septa, with yellowish parietal pigments, often with abundant crystalline or granulose yellow incrustations. ***Clamp connections*** present.

**Figure 29.**
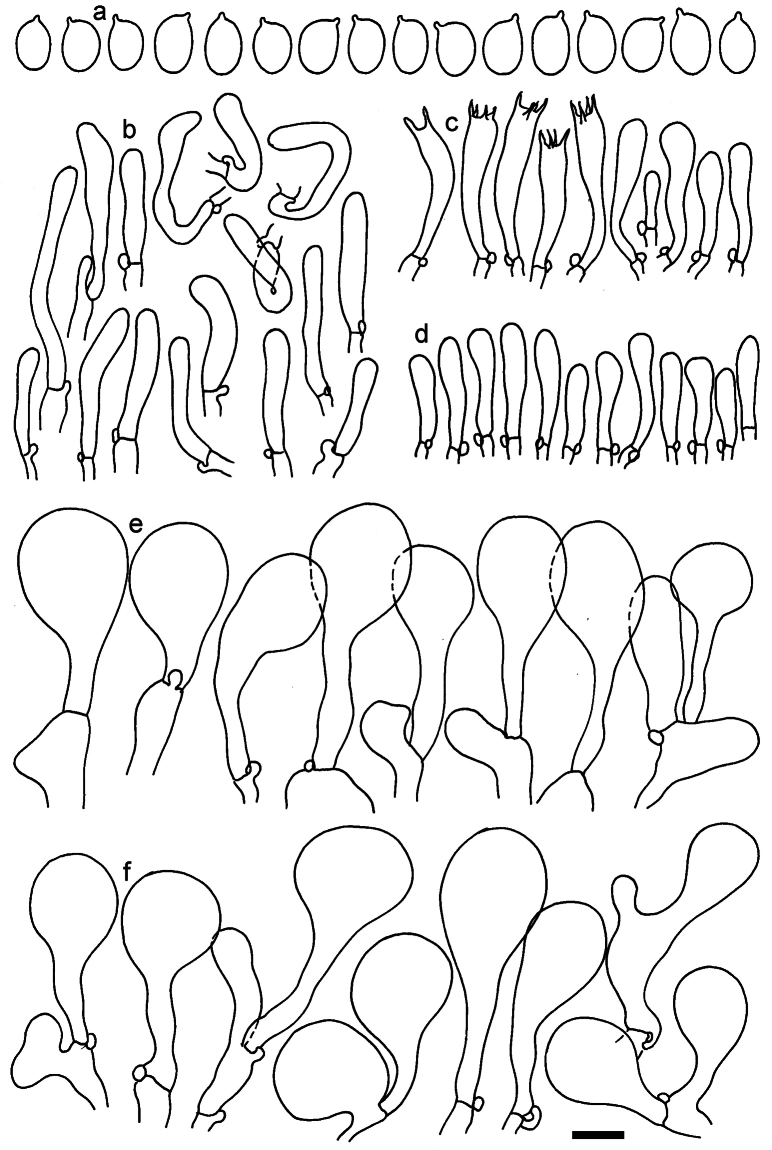
Dermolomaaff.fusipes (SAV F-4371), microscopic elements. **a** Spores; **b** caulocystidia; **c** basidia and basidioles; **d** marginal cells; **e** pileipellis elements near the pileus margin; **f** pileipellis elements near the pileus center. Scale bar: 5 µm for spores and 10 µm for other elements.

###### Distribution and ecology.

Known from one locality in Wales (United Kingdom).

###### Material studied.

United Kingdom • Wales, Powis Castle gardens, coord. 52°38'58"N, 03°09'34"E, terrestrial in lawn, 22 Oct 2014, S. Adamčík (SAV F-4371).

###### Notes.

This taxon is morphologically and phylogenetically very close to *D.fusipes*, but we had only one sample with good DNA quality. As mentioned above, sequences of French (CLF 04/249) and Croatian (CLF 01/2851) samples were of poor quality but clustered with this taxon. Dermolomaaff.fusipes was recognized by Sánchez-García et al. (2021) as “*Dermoloma* sp. 5”.

##### ﻿ Dermoloma griseobasale

Taxon classificationAnimaliaAgaricalesTricholomataceae

Mešić, Tkalčec & Corriol sp. nov.

FEA3702E-C1D6-566D-B888-2961AF240566

856339

Figs 27d–f , 30

###### Etymology.

In reference to the dark gray brown stipe base.

###### Holotype.

Romania • Trascaului Mts., pasture 1 km NE of Rimetea, elev. 550–650 m, coord. 46°27'44"N, 23°34'50"E, terrestrial under *Pyruscommunis*, 27 Sep 2015, S. Adamčík (SAV F-4723).

###### Diagnosis.

European species; basidiomata medium sized colybioid; pilei 7–20 mm in diameter; stipes 1–3.5 mm wide, pruinose near lamellae, grayish brown to dark brown near bases; lamellae relatively pale, brownish ochraceous-white, ochraceous-gray, brownish gray to brownish ochraceous; spores amyloid; marginal cells clavate and often lobate or with lateral projections; caulocystidia 5–10 μm wide.

***Pileus*** 7–20(–22) mm; convex to plano-convex, soon expanding to plane, indistinctly umbonate; margin not striate or sometimes translucently striate up to 2/3 of the radius; surface smooth, sometimes rugulose or slightly veined near center, hygrophanous; color when young dark brown (6F6, 6F7), when mature near margin brown (5E4, 5E6, 6E4), when dry grayish brown (5E5, 6D3, 6E3), near center dark brown (5F4, 6F4, 6F6, 6F8) or yellowish brown (5E5), when dry brownish ochraceous (5C4). ***Stipe*** (15–)17–37(–54) × 1–3.5 mm; cylindrical, narrowed towards the base, flexuous; surface pruinose near lamellae, finely fibrillose or smooth and shiny towards the base; color near lamellae light brown (5D4), grayish brown (5D3, 6E3), brown (5E6), near the base grayish brown (5E3, 6E3), dark brown (6F4, 6F5, 6F6, 7F4). ***Lamellae*** L = (11–)13–32, l = 1–3; 2–5 mm wide; adnate-emarginate and decurrent with tooth; color ochraceous-white (5A2), ochraceous-gray (5B2), brownish gray (5C2) to brownish ochraceous (5C3); edges entire or slightly irregular. ***Context*** when young elastic, later fragile; odor farinaceous.

***Spores*** (4.6–)5.3–6.1–7(–9.4) × (3.5–)3.7–4.1–4.4(–5) μm; ellipsoid to oblong, Q = (1.27–)1.37–1.49–1.61(–1.92); walls amyloid; hilar appendage ca. 0.5–1.5 μm long. ***Basidia*** (22–)26–29.3–32.5(–37) × (4–)6–6.3–7(–7.5) μm; clavate; with 4 sterigmata. ***Basidioles*** first cylindrical, then clavate, ca. 2–6.5 μm wide. ***Marginal cells*** (13–)16.5–21.2–25.5(–30) × (3.5–)6–7.8–9.5(–12) μm; usually clavate, often irregularly lobate, sometimes with short outgrowths. ***Pileipellis*** 50–60 μm deep; suprapellis of mainly one or two layers of inflated cells, loosely arranged and forming disconnected clusters near surface; subpellis not well-differentiated, of 3–15 μm wide hyphae gradually passing to horizontally oriented hyphae in trama; hyphal terminations with brownish and sometimes also dark brown parietal pigments, locally also with incrusted pigments, thin-walled or occasionally thickened up to 1 μm, especially near septa of terminal cells. Terminal cells near pileus margin (16–)35.5–46.7–58(–82) × (10–)16–24–32(–66) μm; usually sphaeropedunculate or obpyriform, sometimes with one or two constrictions near septa; subterminal cells usually narrower or more inflated and usually equally long, often branched. Terminal cells near pileus center (23.5–)36–46–56(–75) × (13–)17–23–28.5(–43) μm; usually sphaeropedunculate or clavate, sometimes obpyriform, rarely almost cylindrical with central constriction, often lobate or with lateral outgrowths; subterminal cells usually narrower, cylindrical or fusiform, usually not inflated, often branched. ***Caulocystidia*** (10.5–)19–30.1–41(–76) × (3–)5–7.3–10(–17) μm; usually clavate, sometimes cylindrical, often slightly flexuous, sometimes lobate, often clustered in small ascending fascicules, sometimes individual and repent; usually with slightly (1 μm) thickened walls especially near septa, with brownish parietal pigments. ***Clamp connections*** present.

**Figure 30.**
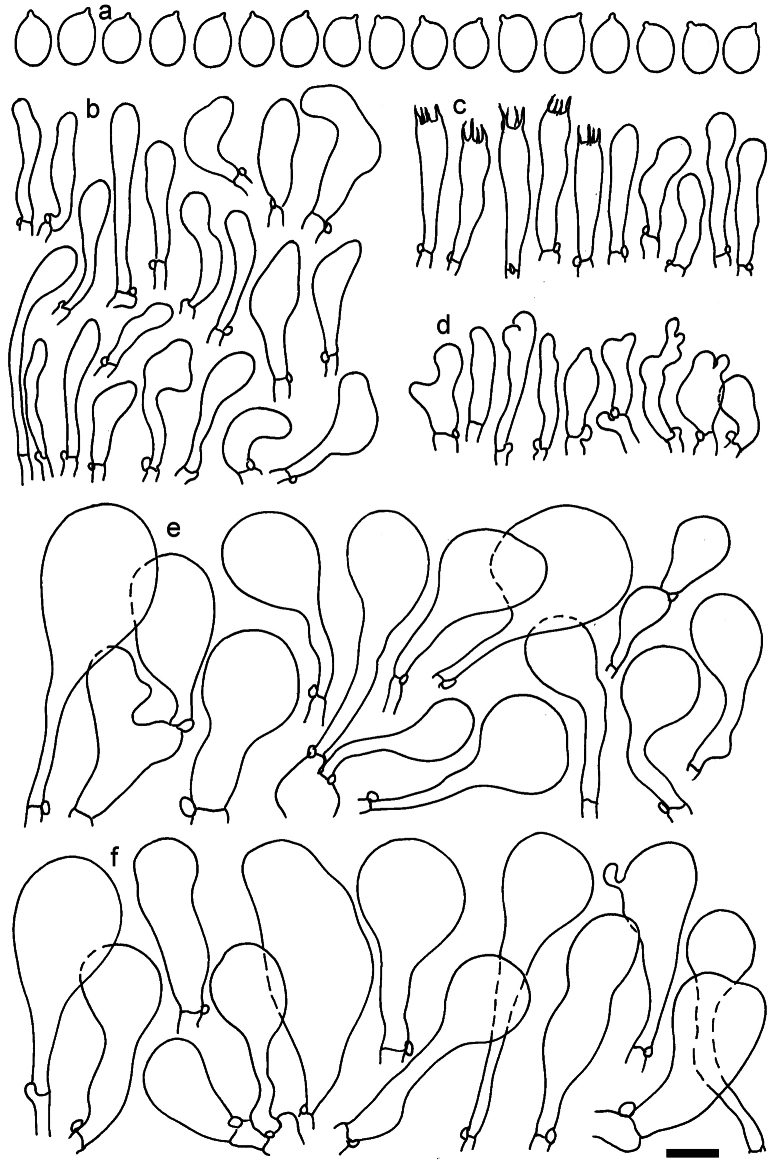
*Dermolomagriseobasale* (SAV F-4151), microscopic elements. **a** Spores; **b** caulocystidia; **c** basidia and basidioles; **d** marginal cells; **e** pileipellis elements near the pileus margin; **f** pileipellis elements near the pileus center. Scale bar: 5 µm for spores and 10 µm for other elements.

###### Distribution and ecology.

Widely distributed in Europe and probably relatively common, known from Austria, Croatia, France, Germany, Italy, Romania and Slovakia; in oligothrophic (non-fertilized) semi-natural grasslands, probably preferring calcareous soil.

###### Additional material studied.

Austria • Steiermark, Rabenstein S of Frohnleiten, elev. 481 m, coord. 47°14'56"N, 15°18'23"E, 26 Sep 2020, G. Friebes *GF20200173* (SAV F-23427). Croatia • Ličko Lešće, near Ramljani village, Podkoren area, coord. 44°45'31"N, 15°23'01"E, rocky semi-natural grassland with sparse shrubs of *Juniperuscommunis*, A. Mešić (CNF 5/287); • Zagreb, Črnomerec, coord. 45°50'04"N, 15°57'03"E, mowed grassland with short grass and mosses, 6 Nov 1998, A. Mešić (CNF 5/297); • Zagreb, Črnomerec, coord. 45°50'04"N, 15°57'03"E, grassland mowed twice a year, 25 Oct 2012, Z. Tkalčec (CNF 1/6490). France • Hautes-Pyrénées, Castet de Gerde, coord. 43°03'35"N, 00°09'54"E, Cynosurion, 20 Oct 2007, G. Corriol *GC07102002* (BBF, as *D.hygrophorus*); • ibid., 2 Nov 2010, G. Corriol *GC10110201* (BBF, as *D.pseudocuneifolium*); • Pyrénées Atlantiques, Portalet, coord. 42°48'60"N, 00°24'54"E, terrestrial in extensively grazed pasture, 7 Oct 2022, M. Caboň (SAV F-22284). Germany • Mittelherwigsdorf, Katzenlehne, coord. 50°55'29"N, 14°44'47"E, on loamy soil in tall grass on a sun-exposed, frequently mown ancient meadow, 2 Oct 2022, A. Karich *IHI-22Der03* (GLM-F137753). Italy • Genova, San Martino di Nuoto, Oliveto, 19 Nov 2011, F. Bocianolo *G2434* (GDOR, as *D.cuneifolium*); • Savona, Sassello, Periaschi, 2. Nov 2013, F. Bocianolo *G3183* (GDOR, as *D.phaeopodium*). Slovakia • Biele Karpaty Mts., 2.5 km NW of Zlatovce, coord. 48°54'37"N, 18°00'14"E, elev. 223 m, terrestrial in semi-natural grassland, 17 Sep 2014, V. Kautman (SLO 692); • ibid., 17 Sep 2014, V. Kautman (SLO 694); • Laborecká vrchovina Mts., 1.5 km NNE of Svetlice, elev. 390 m, coord. 49°11'03"N, 22°02'38"E, terrestrial in pasture, 23 Oct 2007, S. Adamčík (SAV F-4151).

###### Notes.

*Dermolomagriseobasale* has amyloid spores and belongs to D.subgenusAmylospora, section Atrobrunnea. Most cyllybioid members of the section together with D.griseobasale, form a well-supported clade of morphologically similar species (Fig. 2). Within this clade, *D.griseobasale* is placed in an isolated phylogenetic position. Morphologically, it is very similar to *D.confusum*, *D.phaeopodium* and other members of the clade and its identification requires special attention. We therefore recommend to use the heat map (Fig. 4), the chromatogram (Fig. 6), the key and other data available in this study, and in case of uncertainty, combine them with an ITS sequence. This species was included in the phylogenetic study by Sánchez-García et al. (2021) as “D.cf.pseudocuneifolium like”.

##### ﻿ Dermoloma huartii

Taxon classificationAnimaliaAgaricalesTricholomataceae

P.-A. Moreau & Corriol sp. nov.

D6D2B267-4F3C-5B0E-BD45-D3B38622E4FB

856341

Figs 31a, b , 32

###### Etymology.

Named in honor of the French mycologist Didier Huart, an expert in grassland fungi who provided three collections of this species including the holotype.

###### Holotype.

France • Pas-de-Calais, Neufchâtel-Hardelot, réserve naturelle du Mont-Saint-Frieux, coord. 50°36'39"N, 01°36'352"E calcareous grassland, 9 Nov 2014, D. Huart and P.-A. Moreau *PAM14110907* (LIP).

###### Diagnosis.

European species; basidiomata medium to large; pilei 20–30 mm in diameter; stipes usually 2–5 mm wide; lamellae pale ochraceous-gray to white; spores inamyloid, on average longer than 5.5 µm and often wider than 4 µm.

***Pileus*** 20–30(–50) mm; convex, soon expanding to plane, sometimes indistinctly umbonate, often lobate; margin usually not striate, indistinctly translucently striate up to half of the radius when wet, recurved when old; surface near margin smooth, sometimes radially rugulose or wrinkled near center, slightly hygrophanous; color when young dark brown (5F3, 8F5), when mature near margin brown (5E4), dark blond (5D4), grayish brown (5D3), ochraceous-gray (5B2, 6B2), brownish ochraceous (6C3), near center dark brown (5F5, 6F4), brown (5E4). ***Stipe*** (22–)32–50(–70) × 2–5(–8) mm; cylindrical, sometimes fusiform, narrowed towards the base, flexuous; surface finely longitudinally striate, granulose or flocculose near lamellae, towards the base finely fibrillose; color near lamellae ochraceous-gray (5B2) to almost white, near the base brownish gray (5C2) to grayish brown (5D3). ***Lamellae*** L = (24–)26–40(–45), l = 1–3(–7); 4–6 mm wide; adnate-emarginate and decurrent with tooth; color ochraceous-gray (5B2), towards edges almost white; edges slightly irregular. ***Context*** when young elastic, later fragile; odor farinaceous.

**Figure 31.**
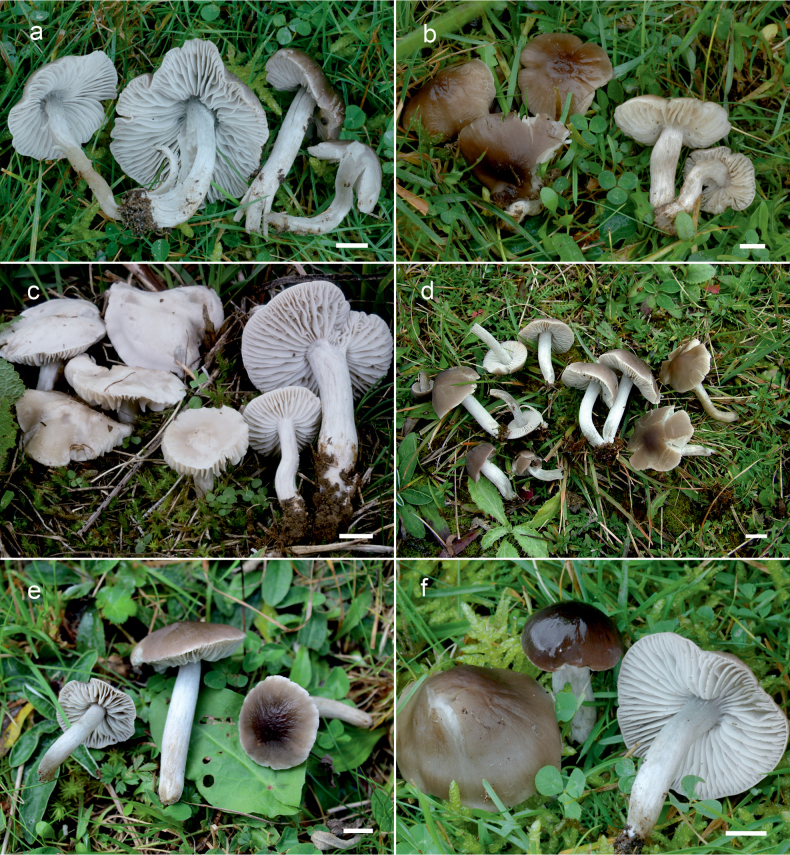
Basidiomata of *Dermoloma* in field appearance. **a***Dermolomahuartii* [LIP (*PAM14110907*), holotype], photo D. Huart; **b***Dermolomahuartii* [LIP (*PAM14110909*)], photo D. Huart; **c***Dermolomahygrophorus* [BBF (*GC01102501*)], photo G. Corriol; **d***Dermolomaintermedium* (SAV F-4270), photo S. Jančovičová; **e***Dermolomaintermedium* (SAV F-22209), photo S. Jančovičová; **f***Dermolomaintermedium* [LIP (*PAM14110905*)], epitype, photo P.-A. Moreau. Scale bar: 10 mm.

***Spores*** (5.2–)5.6–6.2–6.9(–8.2) × (3.5–)3.9–4.4–4.9(–5.4) μm; broadly ellipsoid to narrowly ellipsoid, Q = (1.28–)1.34–1.42–1.50(–1.62); walls inamyloid; hilar appendage ca. 1–1.5 μm long. ***Basidia*** (19–)23–26.4–30(–36.5) × (4.5–)6–6.4–7(–8) μm; clavate; usually with 2 sterigmata, occasionally with 1, 3 or 4 sterigmata. ***Basidioles*** first cylindrical, then clavate, ca. 2–6.5 μm wide. ***Marginal cells*** (7.5–)11.5–14.2–17(–19) × (3.5–)4–4.6–5.5(–6) μm; not well-differentiated, cylindrical or clavate, sometimes ellipsoid, often lobate. ***Pileipellis*** 57–70 μm deep; suprapellis of one or two layers of inflated cells, gradually passing to 23–33 μm deep subpellis of densely packed, irregularly oriented, puzzled, 3–10(–13) μm wide hyphae, not sharply delimited from horizontally oriented hyphae in trama; hyphal terminations with brownish yellow parietal pigments, walls thickened up to 1(–1.5) μm and occasionally with yellow-brown incrusted pigments especially near septa of terminal cells and in subpellis. Terminal cells near pileus margin (15.5–)32.3–43.6–55(–85) × (9–)14–21.1–28(–47) μm; usually obpyriform, clavate or sphaeropedunculate, rarely ellipsoid or fusiform; subterminal cells usually narrower and unbranched, clavate or obpyriform, often with lateral swellings. Terminal cells near pileus center (17–)32–42.5–52.5(–71) × (9.5–)17–23–28.5(–37.5) μm; similar to cells near margin but more frequently irregularly lobate; subterminal cells narrower or equally wide, often with lateral swellings or irregularly lobate. ***Caulocystidia*** (12.5–)25.5–35.7–45.8(–66) × (2–)4–6.1–8(–11) μm; clavate or cylindrical, often slightly flexuous, sometimes moniliform, often clustered in small ascending fascicules, sometimes individual and repent; usually with slightly thickened walls up to 0.5 μm, often with crystalline or granulose yellow incrustations. ***Clamp connections*** present.

**Figure 32.**
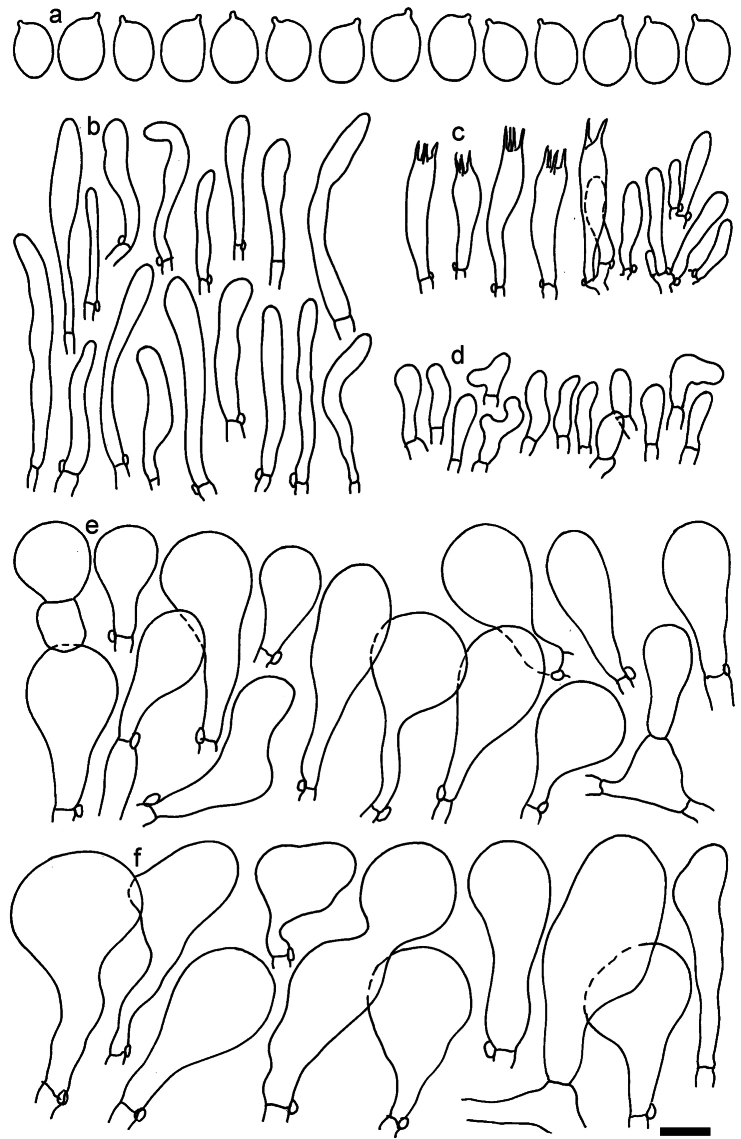
*Dermolomahuartii* [LIP (*PAM14110907*), holotype], microscopic elements. **a** Spores; **b** caulocystidia; **c** basidia and basidioles; **d** marginal cells; **e** pileipellis elements near the pileus margin; **f** pileipellis elements near the pileus center. Scale bar: 5 µm for spores and 10 µm for other elements.

###### Distribution and ecology.

Known from France, Slovakia and Wales (United Kingdom), in semi-natural grasslands on calcareous soil, once also collected in a calcareous beech forest.

###### Additional material studied.

France • Ariège, Ker de Massat, coord. 42°53'45"N, 01°18'57"E, calcareous beech forest with *Buxussempervirens*, 9 Oct 2017, C. Hannoire, *CH17100919* (BBF, as *D.cuneifolium*); • Pas-de-Calais, Neufchâtel-Hardelot, réserve naturelle du Mont-Saint-Frieux, coord. 50°36'39"N, 01°36'35"E, calcareous semi-natural grassland, 9 Nov 2014, D. Huart and P.-A. Moreau *PAM14110904* (LIP); • ibid., 9 Nov 2014, D. Huart and P.-A. Moreau *PAM14110909* (LIP). Slovakia • Strážovské vrchy Mts., motocross area 1 km W of Čelkova Lehota, elev. 460 m, 49°00'55"N, 18°31'12"E, grassland dominated by *Dactylisglomerata* and *Trifoliumarvense*, 6 Oct 2005, V. Kučera (SAV F-4149). United Kingdom • Wales, Powis Castle gardens, coord. 52°38'58"N, 03°09'34"E, terrestrial in lawn, 22 Oct 2014, D. Harries (SAV F-4378).

###### Notes.

With inamyloid spores, *D.huartii* belongs to D.subgenusDermoloma, section Dermoloma, and is closely related to type of the genus *D.cuneifolium* (Fig. 2). All species in the section are very similar and their identification requires special attention (see notes under *D.cuneifolium*). *Dermolomahuartii* is a medium-sized species that can be distinguished from *D.cuneifolium* by the spores longer than 5.5 μm and from the other large-capped species of the section by the stipe up to 5 mm wide. This species was included in the phylogenetic study by Sánchez-García et al. (2021) as “D.cf.cuneifolium”.

##### ﻿ Dermoloma hygrophorus

Taxon classificationAnimaliaAgaricalesTricholomataceae

Joss., Bull. mens. Soc. Linn. Lyon 39(1): 6. 1970.

4BD1056C-F2E4-5B62-8819-315373961716

312909

Figs 31c , 33

###### Holotype.

France • Ain, Quincieu, 5 Aug 1953, G. Lacombe and M. Josserand (G00127362).

###### Distinguishing characters.

European species; basidiomata large and robust, pale grayish, cream or almost white colors at all parts; pilei 25–45 mm in diameter; stipes 5–10 mm wide; lamellae up to 30 near the stipe attachment; spores amyloid.

***Pileus*** 25–45 mm; convex to plane, sometimes indistinctly umbonate, sometimes asymmetrical; margin straight, sometimes lobate, not striate; surface matt, fairly smooth to rugulose-subsquamulose, slightly hygrophanous; colors start to fade from center, pale grayish cream, becoming white when drying. ***Stipe*** 30–40 × 5–10 mm; fusiform or cylindrical, usually narrowed towards the base, flexuous; surface glabrous to finely longitudinally striate, sometimes weakly pruinose or squamulose; color marbled with white and pale gray. ***Lamellae*** L = 16–30, l = (0–)1–3(–7), occasionally forked to strongly interveined-anastomosed; 4–8 mm wide; adnate-emarginate; color white to very pale grayish; edges entire or slightly irregular. ***Context*** up to 3.5 mm wide at mid-radius, 8 mm wide at center; fragile; odor strongly farinaceous.

**Figure 33.**
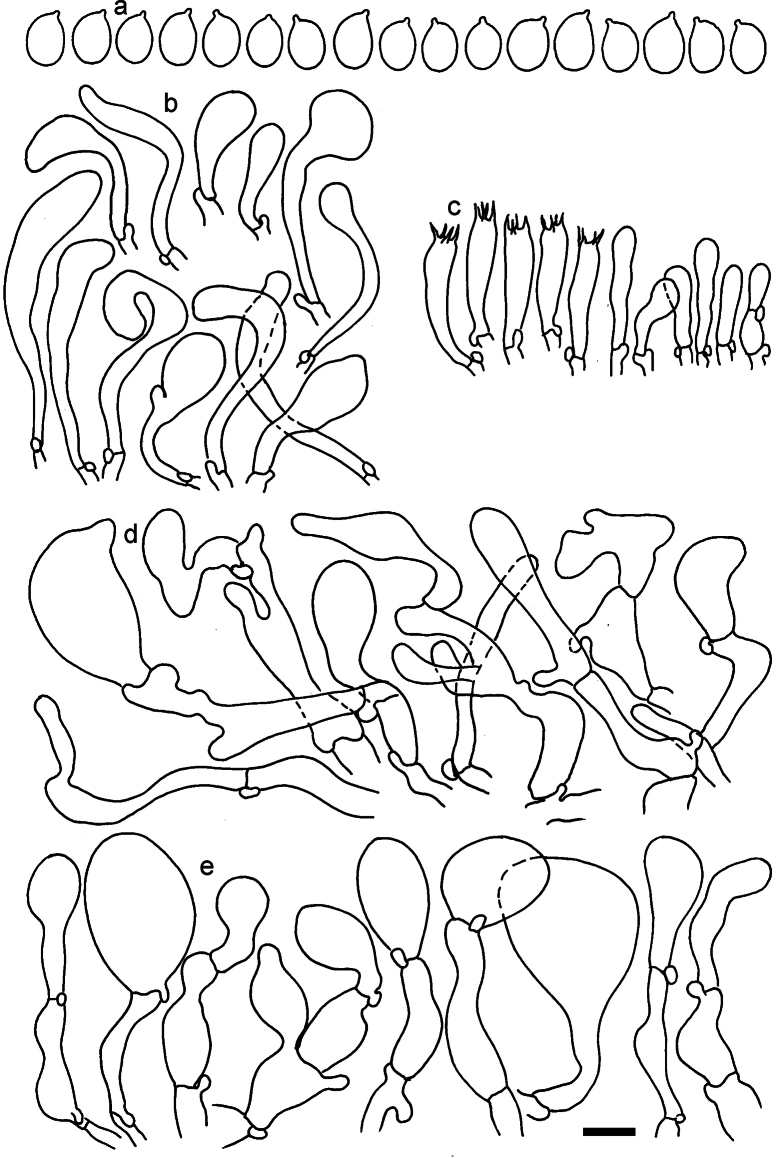
*Dermolomahygrophorus* (G00127362, holotype), microscopic elements. **a** Spores; **b** caulocystidia; **c** basidia and basidioles; **d** pileipellis elements near the pileus margin; **e** pileipellis elements near the pileus center. Scale bar: 5 µm for spores and 10 µm for other elements.

***Spores*** (4.8–)5.2–5.7–6.2(–7.1) × (3.6–)3.9–4.2–4.6(–5) μm; broadly ellipsoid to ellipsoid, Q = (1.08–)1.27–1.34–1.41(–1.53); walls amyloid; hilar appendage ca. 0.5–1 μm long. ***Basidia*** (20–)23–26.4–29(–32) × (4.5–)5.8–6.5–7.2(–8) μm; clavate; with 4 sterigmata. ***Basidioles*** first cylindrical, then clavate, ca. 3–6 μm wide. ***Marginal cells*** not observed. ***Pileipellis*** ca. 30–40 μm deep; suprapellis mainly of one or two layers of inflated, irregularly arranged cells; subpellis not well-differentiated, of densely packed, mainly horizontally oriented, intricate, 2.5–12 μm wide hyphae, sharply delimited from underlying hyphae of trama; hyphal terminations with brownish yellow parietal pigments, usually thin-walled. Terminal cells near pileus margin (17–)25–35.7–45.5(–67) × (4.5–)8.5–14.7–21(–30) μm; clavate obpyriform or fusiform, rarely cylindrical, usually irregularly lobate, often with lateral projections, often flexuous; subterminal cells usually narrower and subcylindrical, rarely clavate or fusiform, flexuous, often branched with lateral projections or lobate. Terminal cells near pileus center (13–)23–32.7–42.5(–62) × (6–)9.5–14.7–19.5(–28) μm; mainly clavate, often ellipsoid, obpyriform or fusiform, rarely sphaeropedunculate, less frequently lobate or flexuous, occasionally with lateral projections or nodules; subterminal cells ventricose or subcylindrical, often with lateral swellings or lobate. ***Caulocystidia*** (23–)32.5–45.1–58(–71) × (5.5–)6–8.3–10.5(–15) μm; clavate, sometimes sphaeropedunculate or obpyriform, apically obtuse, usually flexuous or twisted, repent with ascending tips or erect, often irregularly oriented, individual or in small to large fascicules; thin-walled, sometimes with thickened walls up to 0.5 with μm. ***Clamp connections*** present.

###### Distribution and ecology.

Known from two localities in France; in grasslands, probably preferring calcareous soil.

###### Additional material studied.

France • Ain, Quincieu, W of Grenoble, plain pasture with only a few *Crataegusoxyacantha*, G. Lacombe and M. Josserand, 14 Aug 1956 (G00260855); • ibid., 22 Aug 1956 (G00260854); • Dordogne, Thénon, coord. 45°08'13"N, 01°03'57"E, grassland on calcareous soil (*Mesobromion*), 25 Oct 2001, G. Corriol *GC01102501* (BBF).

###### Notes.

*Dermolomahygrophorus* is a member of D.subgenusAmylospora, section Atrobrunnea. It is among the most easily distinguishable species of the genus because of its large, fleshy and pale-colored basidiomata. The phylogenetically most closely related species is *D.parvisporum* (Fig. 2), which is characterized by the small mycenoid and dark colored basidiomata (see below). The morphologically most similar species is *Neodermolomacampestre*, but this is a North American species with more crowded lamellae and different elements in the pileipellis. The type sequence of *D.hygrophorus* was used by Sánchez-García et al. (2021) as the only sample of the species included in their phylogeny. The sequence is only partial and of poor quality which resulted in an unresolved position of the species within D.sectionAtrobrunnea. Recent collections, included in our study, allowed more precise placement of the species that is supported as sister to *D.parvisporum*.

##### ﻿ Dermoloma intermedium

Taxon classificationAnimaliaAgaricalesTricholomataceae

Bon, Doc. Mycol. 9(35): 42. 1979.

EA3234A0-C189-527B-90B2-BCAD7FB7F9CE

312910

Figs 31d–f , 34

Dermolomaalexandri Consiglio in Contu, Consiglio & Setti, Micol. Veg. Medit. 22(2): 84. 2008. Syn.

###### Holotype.

France • Somme, Airaines, Warlus, deciduous forest on limestone, Cephalantero-Fagion, Oct. 1967, M. Bon *71081* (LIP).

###### Epitype.

(designated here MBT10022921): France, Pas-de-Calais, Neufchâtel-Hardelot, réserve naturelle du Mont-Saint-Frieux, coord. 50°36'39"N, 01°36'35"E, calcareous grassland, 9 Nov 2014, D. Huart and P.-A. Moreau *PAM14110905* (LIP).

###### Distinguishing characters.

European species; basidiomata usually large; pilei 20–52 mm in diameter, distinctly hygrophanous; stipes 4–10 mm wide; lamellae 32–50 near the stipe attachment, gray to brownish gray; spores inamyloid.

***Pileus*** (13–)20–52(–65) mm; convex, later almost plane, indistinctly umbonate, rarely obtusely conical; margin usually not striate, indistinctly translucently striate to half of the radius when wet, recurved when old, when dry radially cracking; surface smooth, sometimes radially rugulose or wrinkled near center, matt, sometimes pruinose, hygrophanous; color when young dark brown (7E4), when mature near margin sometimes with white outline, dark brown (6F3, 6F4), brown (6E4), grayish brown (5E3, 6D3, 6E3), when dry brown (6E4), grayish brown (6D3, 6E3), brownish gray (5D2, 6D2), brownish ochraceous (6C3), near center dark brown (6F3, 6F4, 6F5, 6F7, 7F3, 7F4, 7F6) to black, rarely brown (6E5), when dry brown (5E3, 5E4, 6E4) to black, sometimes grayish brown (5D3). ***Stipe*** (20–)26–59(–80) × (3–)4–10(–13) mm; cylindrical, narrowed towards the base, usually flexuous, especially near the base; surface finely longitudinally striate, near lamellae finely granulose or pruinose, towards the base fibrillose or flocculose, with age and especially near the base silky and shiny; color near lamellae pale gray (B1 to C1) to almost white, sometimes brownish gray (6C2, 7C2), near the base brownish gray (5C2, 5D2, 5E2, 6C2, 6D2), grayish brown (5D3, 6E3), brownish ochraceous (5C3) or sometimes ochraceous-gray (5B2). ***Lamellae*** L = (28–)32–50(–52), l = (0–)1–3(–7); 5–12 mm wide; adnate-emarginate and decurrent with tooth; color gray (B1, C1), brownish gray (5C2, 6C2, 6D2); edges entire or slightly irregular, rarely serrulate. ***Context*** when young compact, later fragile; odor farinaceous.

**Figure 34.**
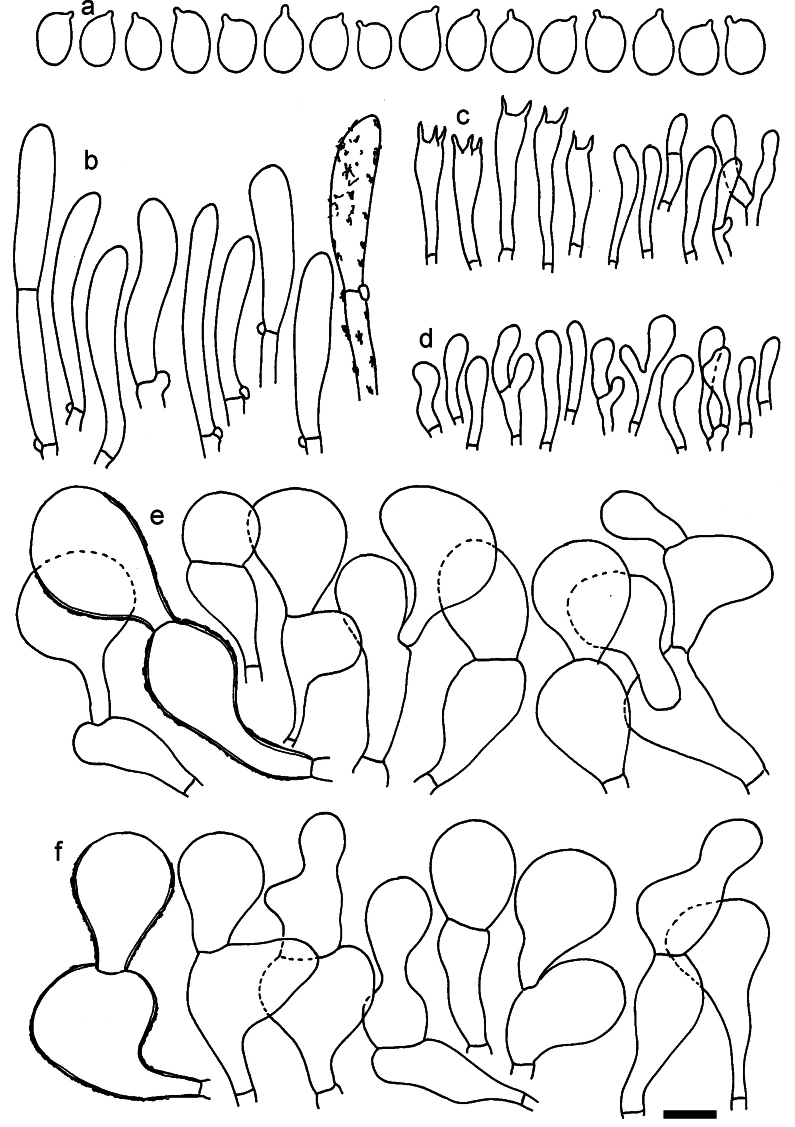
*Dermolomaintermedium* [LIP (*Bon 71081*), holotype], microscopic elements. **a** Spores; **b** caulocystidia; **c** basidia and basidioles; **d** marginal cells; **e** pileipellis elements near the pileus margin; **f** pileipellis elements near the pileus center. Scale bar: 5 µm for spores and 10 µm for other elements.

***Spores*** (4.8–)5.1–5.4–5.8(–6.3) × (3.4–)3.7–4–4.2(–4.8) μm; broadly ellipsoid to ellipsoid, Q = (1.15–)1.27–1.37–1.46(–1.62); walls inamyloid, sometimes thick-walled and dextrinoid; hilar appendage 0.7–1.3 μm long. ***Basidia*** (20–)23.5–26.7–29.7(–34) × (4.5–)6–6.4–7(–8) μm; clavate; usually with 4 sterigmata, but one collection (the type) with (1–)2–3 sterigmata. ***Basidioles*** first cylindrical, then clavate, ca. 3–5.5 μm wide. ***Marginal cells*** (5.5–)12–16.2–21(–24) × (3–)3.5–4.4–5(–5.5) μm; cylindrical or clavate, not well-differentiated from basidioles on lamellae sides but usually smaller. ***Pileipellis*** 60–70 μm deep; suprapellis of mainly one, occasionally two layers of inflated cells, gradually passing to 23–26 μm deep subpellis of densely packed, irregularly oriented, puzzled, 4­–10(–15) μm wide hyphae, not sharply delimited from horizontally oriented hyphae in trama; hyphal terminations with dark brown parietal pigments, thin-walled or occasionally thickened up to 1 μm and with dark incrusted pigments especially near septa of terminal cells. Terminal cells near pileus margin (15–)26–35.9–46(–75) × (10–)14.5–20.7–27(–44) μm; usually obpyriform or clavate, sometimes sphaeropedunculate, ellipsoid or subglobose; subterminal cells usually narrower and unbranched, clavate or obpyriform, often with lateral swellings. Terminal cells near pileus center (18–)28.5–39–49.5(–82) × (9–)14.5–20.9–27.5(–47) μm; usually obpyriform, ellipsoid or subglobose, rarely irregularly utriform or clavate-lageniform; subterminal cells narrower or equally wide, often with lateral swellings or irregularly lobate. ***Caulocystidia*** (16–)29.5–39.3–49.5(–65) × (3.5–)4.5–6.1–8(–11) μm; clavate or cylindrical, usually not or only slightly flexuous, often clustered in small ascending fascicules, sometimes individual and repent; usually with slightly thickened walls up to 0.5 μm, often with crystalline or granulose yellow incrustations. ***Clamp connections*** usually present, but absent in the type collection.

###### Distribution and ecology.

Widely distributed and rather frequent in Europe; in semi-natural grasslands, natural, dry “steppe-like” grasslands, maquis shrublands and forests on calcareous soil.

###### Additional material studied.

Austria • Burgenland, Galgenberg, 1.1 km E of Markt Neuhodis, elev. 336 m, coord. 47°18'01"N, 16°24'38"E, 30 Nov 2019, G. Friebes *GF20190143* (SAV F-23425); • Burgenland, Siegendorfer Puszta, Siegendorf, elev. 172 m, coord. 47°46'40"N, 16°34'53"E, 29 Oct 2017, G. Friebes *GF20170122* (SAV F-23424); • Steiermark, Pöllau, elev. 722 m, coord. 47°13'56"N, 15°22'18"E, 26 Oct 2014, G. Friebes *GF20140133A* (SAV F-23428). Croatia • Mljet island, Mljet National Park, 1.1 km E/E-SE of Goveđari village, coord. 42°46'54"N, 17°22'33"E, rocky footpath with maquis of *Arbutusunedo*, *Ericaarborea*, *Phillyrealatifolia*, *Pinushalepensis* and *Quercusilex*, 13 Nov 2010, A. Mešić (CNF 1/6124); • Mljet island, 250 m NW/W-NW of Prožurska luka village, coord. 42°43'51"N, 17°38'44"E, among mosses, hiking trail along maquis of *Arbutusunedo*, *Myrtuscommunis*, *Pinushalepensis* and *Pistacialentiscus*, 9 Nov 2009, Z. Tkalčec (CNF 1/5693); • Mljet island, 500 m NW of Prožurska luka village, coord. 42°43'57"N, 17°38'40"E, maquis of *Arbutusunedo*, *Myrtuscommunis*, *Pinushalepensis* and *Pistacialentiscus*, 9 Nov 2009, A. Mešić (CNF 1/5713); • near Krasno village, Velebit Mt., coord. 44°49'17"N, 15°02'01"E, pasture with young trees of *Piceaabies* and *Abiesalba*, on the border of *Fagussylvatica*, *Piceaabies* and *Abiesalba* forest, 30 Sep 2006, Z. Tkalčec (CNF 1/4218); • Zagreb, Črnomerec, coord. 42°49'58.91"N, 15°56'54.6"E, grassland, mowed twice a year, 13 Oct 2010, Z. Tkalčec (CNF 1/6496). Estonia • Saarnaki island near Hiiumaa island, 13 km SEE of Käina, elev. 5–10 m, coord. 58°48'7.7"N, 23°00'3.93"E, soil among mosses and low scattered grass, associated with *Juniperus* sp., 23 Sep 2008, S. Adamčík (SAV F-4147). Finland • Åland, Finström, Bergö, Husö Biological Station, coord. 60°16'33.6"N, 19°50'34.8"E, pasture, 19 Sep 1989, J. Vauras *FISAP654-13* (H6034917). France • Hautes-Pyrénées, Bagnères-de-Bigorre, Bizourtère, coord. 42°57'19"N, 00°05'19"E, acidophilous mountain grassland (*Nardion*), 8 Oct 2014, C. Hannoire *CH14100810* (BBF, as *D.fuscobrunneum*); • Pas-de-Calais, Neufchâtel-Hardelot, réserve naturelle du Mont-Saint-Frieux, 50°36'39"N, 01°36'35"E, calcareous grassland, 10 Nov 2014, D. Huart and P.-A. Moreau *PAM14111007* (LIP). Germany • Baden-Württemberg, Justingen, Schachenheide, coord. 48°24'35"N, 09°40'25"E, terrestrial in grassland, 2 Oct 2021, S. Adamčík (SAV F-20867); • ibid., 2 Oct 2021, S. Adamčík (SAV F-20868); • ibid., 2 Oct 2021, S. Adamčík (SAV F-20869); • ibid., 2 Oct 2021, S. Adamčík (SAV F-20870); • ibid., 2 Oct 2021, S. Adamčík (SAV F-20879); • ibid., 2 Oct 2021, S. Adamčík (SAV F-20880). Hungary • Heves, Mátra Mts., Parádfürdő, coord. 47°53'37"N, 20°03'15"E, terrestrial in semi-natural grassland, 11 Nov 2022, F. Németh *NF-2022-11-11-1* (ELTE). Italy • Emilia-Romagna, Boscone della Mesola, Mesola (FE), under *Quercusilex*, 12 Nov 2004, G. Consiglio, M. Panchetti, R. Bolletta and C. Orlandini *GC04317* (AMB 12794, holotype of *D.alexandri*); • Lazio, Ostia (RM), Tenuta presidenziale di Castelporziano, 2 Dec 2009, G. Consiglio, G. Perdisa, L. Perrone and L. Setti *GC09266* (AMB 12795). The Netherlands • Noord-Holland, Duinweide tegenover Koningshof, 2 Nov 1968, E. Kits van Waveren (L3988081, as D.cuneifoliumvar.punctipes). Norway • Telemark, Bamble, Kjerrvikodden, coord. 58°59'59"N, 09°43'55"E, in calcareous, coastal semi-natural grassland, 15 Oct 2012, T. Læssøe and A. Molia *NOBAS2437-16* (O-F-245550); • ibid., 16 Oct 2013, T. Læssøe and A. Molia *NOBAS3361-16* (O-F-22071). Romania • Vladeasa Mts., pasture 1.2 km N of Belis, elev. 1060–1085 m, coord. 46°41'47"N, 23°02'10"E, terrestrial in pasture, 1 Oct 2014, S. Adamčík (SAV F-4303); • ibid., 5 Oct 2014, M. Caboň (SAV F-4270); • ibid., 6 Oct 2014, S. Adamčík, (SAV F-4305). Slovakia • Laborecká vrchovina Mts., pasture 1 km NW of Vyšná Jablonka, elev. 400–450 m, coord. 49°09'31"N, 22°06'18"E, terrestrial, 21 Sep 2006, S. Adamčík (SAV F-4137). Spain • Aragon, Huesca prov., Canfranc, Río de la Canal Roya, coord. 42°46'26"N, 00°30'56"E, terrestrial on pasture, under *Buxus* sp. and *Pinussylvestris*, 3 Oct 2022, S. Adamčík (SAV F-22198); • ibid., 3 Oct 2022, S. Adamčík (SAV F-22209); • ibid., 6 Oct 2022, S. Adamčík (SAV F-22273); • ibid., 6 Oct 2022, S. Adamčík (SAV F-22274); • ibid., 6 Oct 2022, M. Caboň (SAV F-22275). United Kingdom • Wales, Pembrokeshire Coast National Park Offices, Pembroke Dock, coord. 51°41'55"N, 04°56'12"E, amenity grassland (lawn), 8 Oct 2019, D. Harries *DH 19-20* (SAV F-23433).

###### Notes.

*Dermolomaintermedium* has inamyloid spores and belongs to D.subgenusDermoloma, section Dermoloma, a group of closely related and morphologically similar species. Its phylogenetic position within the section is not resolved (Fig. 2), however, it is among the species with the largest basidiomata in the genus. It is a very common species that can be easily confused with another common species, *D.cuneifolium*, from which it differs by the distinctly hygrophanous pilei and slightly longer spores. Identification of *D.intermedium* requires careful attention and we recommend combining of the use of the heat map (Fig. 4) with other diagrams and data available in this study (see notes under *D.cuneifolium*). This species was included in the phylogenetic study by Sánchez-García et al. (2021) as *D.alexandri*. Here we assign the older name *D.intermedium* to it, based on the morphology of the type, because the holotype sequencing was not successful. It was originally described from France and defined as a species with large pilei (50–80 mm in diam.) and a depressed center, inamyloid spores and broadly adnate to decurrent lamellae (Bon 1979a). Spores from the type were on av. 5.3 × 4.1 μm in size, which fit only two species recognized in this study (Fig. 7). One of these, *D.atrocinereum*, has never been observed with a depressed pileus center. The only candidate matching the type of *D.intermedium* is the species previously identified as *D.alexandri* by a sequence from the type collection; thus, the latter name is here synonymised with *D.intermedium*. Our observations revealed that the pileus diameter of *D.intermedium* does not exceed 65 mm. However, there is no other *Dermoloma* species within the many samples that we examined that had larger pilei and we suspect that Bon (1979a) described his species based on a single collection with untypically large basidiomata. Together with *D.atrocinereum* and *D.cuneifolium*, *D.intermedium* might be the most common and widespread species of the genus in Europe.

##### ﻿ Dermoloma josserandii

Taxon classificationAnimaliaAgaricalesTricholomataceae

Dennis & P.D. Orton in P.D. Orton, Trans. Br. mycol. Soc. 43(2): 226. 1960.

452E901E-239E-5741-815B-69AC81F0EEB3

329824

Figs 35a, b , 36

###### Holotype.

United Kingdom • Somerset, Spaxton, Hawkridge, on soil, 15 Sep 1958, E. Marriage [K(M) 37580].

###### Epitype.

(designated here MBT10023010): Germany • Baden-Württemberg, Justingen, Schachenheide, coord. 48°24'35"N, 09°40'25"E, terrestrial in semi-natural grassland, 3 Oct 2021, leg. S. Adamčík (SAV F-20907).

###### Distinguishing characters.

European species; basidiomata medium sized; pilei 15–21 mm in diameter, near margin light brown at least when dry; stipes 2–5 mm wide; spores amyloid, > 4.3 µm wide.

***Pileus*** (10–)15–21(–24) mm; convex, plano-convex to plane, sometimes umbonate or weakly depressed, sometimes lobate; margin indistinctly translucently striate when wet; surface rugulose, pitted or rough, sometimes smooth, slightly hygrophanous; color when young dark brown (6F4, 6F5, 7F4), when mature near margin light brown (5D4, 5D5), to brown (6E4), when dry light brown (5D6, 6D4) or grayish brown (6C3), near center light brown (5D6, 6D4) to brown (6E4, 6E5, 6E6), when dry brownish ochraceous (5C5) or brown (6E5). ***Stipe*** 20–55 × 2–5 mm; cylindrical, narrowed towards the base, often flexuous especially near the base; surface distinctly longitudinally striate and finely fibrillose especially towards the base, pruinose near lamellae; color near lamellae ochraceous-gray (5B2) to brownish gray (5C2, 6C2), slightly darker near the base, brownish gray (5C2), brownish ochraceous (5C3), dark blond (5D4) to brown (6E4). ***Lamellae*** L = (20–)24–31(–38), l = 0–3(–7); 3–8 mm wide; adnate-emarginate and decurrent with tooth; color ochraceous-gray (5B2, 6B2); edges entire or slightly irregular. ***Context*** when young elastic, later fragile; odor farinaceous.

**Figure 35.**
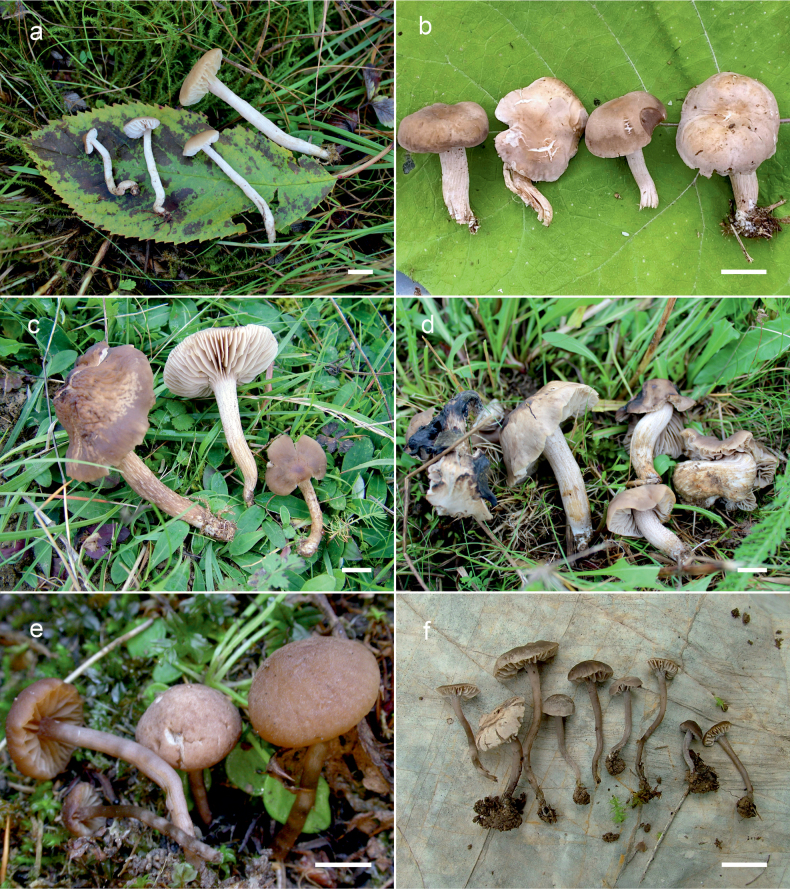
Basidiomata of *Dermoloma* in field appearance. **a***Dermolomajosserandii* (SAV F-20808, epitype), photo S. Jančovičová; **b***Dermolomajosserandii* (SAV F-20907, epitype), photo V. Shapkin; **c***Dermolomamagicum* (SAV F-4142, epitype), photo S. Jančovičová; **d***Dermolomamagicum* (SAV F-20618), photo S. Jančovičová; **e***Dermolomamurinellum* [LIP (*PAM06090203*)], photo P.-A. Moreau; **f***Dermolomaobscurum* (SAV F-20012, holotype), photo S. Jančovičová. Scale bar: 10 mm.

**Figure 36.**
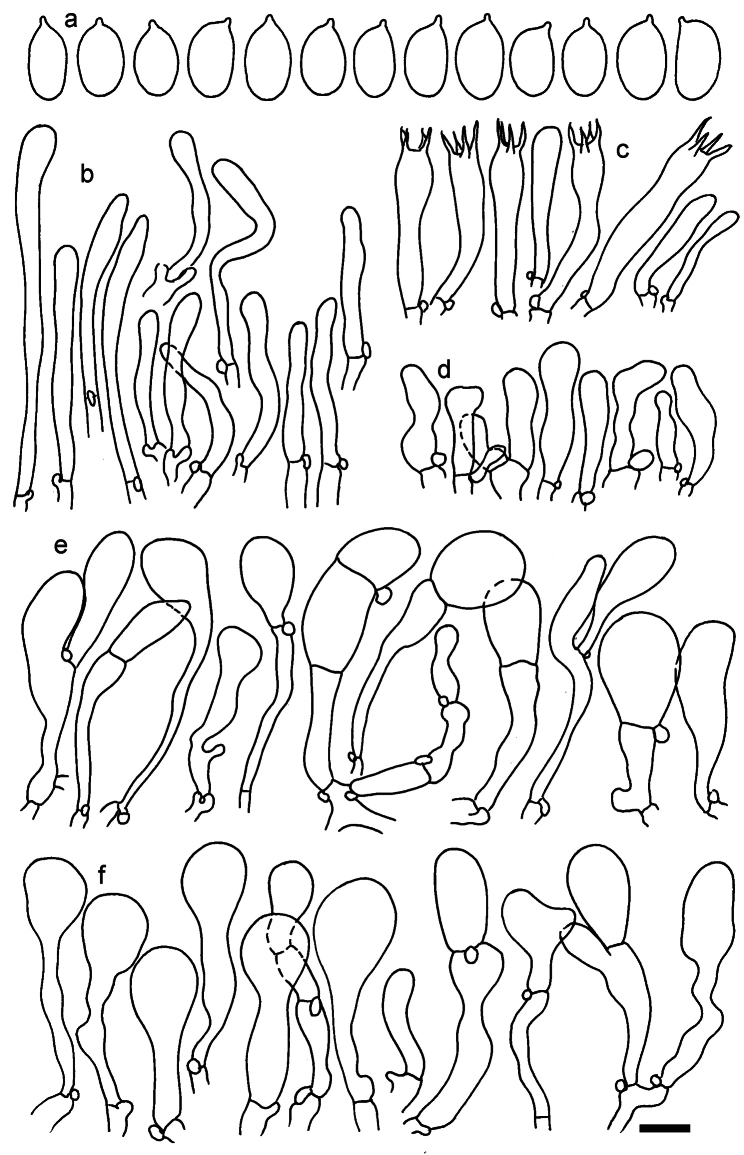
*Dermolomajosserandii* (SAV F-20849), microscopic elements. **a** Spores; **b** caulocystidia; **c** basidia and basidioles; **d** marginal cells; **e** pileipellis elements near the pileus margin; **f** pileipellis elements near the pileus center. Scale bar: 5 µm for spores and 10 µm for other elements.

***Spores*** (5.5–)6.1–6.7–7.4(–8.7) × (4–)4.3–4.6–4.9(–5.4) μm; ellipsoid to oblong, sometimes slightly amygdaloid, Q = (1.24–)1.39–1.53–1.66(–1.86); walls amyloid; hilar appendage 0.7–1.5 μm long. ***Basidia*** (24–)30.5–35.1–39.5(–47) × (6–)6.5–7.3–8(–8.5) μm; clavate; with 4 sterigmata. ***Basidioles*** first cylindrical, then clavate, ca. 3–7 μm wide. ***Marginal cells*** (14–)19–24.4–29.5(–38) × (3–)4.5–6–7.5(–10) μm; clavate or cylindrical, flexuous, occasionally slightly moniliform, nodulose or lobate, apically obtuse. ***Pileipellis*** 55–70 μm deep; suprapellis 40–50 μm deep, usually of one to three layers of inflated, densely arranged cells; subpellis not well-differentiated, ca. 10–20 μm deep, of densely packed, almost horizontally oriented, 2.5–12 μm wide hyphae, not sharply delimited from horizontally oriented hyphae in trama; hyphal terminations with brownish yellow parietal pigments, occasionally also dark brown incrusted pigments, walls thickened up to 0.5 μm, near septa of terminal cells and in subpellis up to 1 μm. Terminal cells near pileus margin (16–)24.5–34.2–43.5(–68) × (8–)11.5–15.7–20(–29) μm; usually clavate or obpyriform, occasionally sphaeropedunculate or subglobose, sometimes with narrowed flexuous basal part; subterminal cells occasionally branched, cylindrical or clavate, rarely inflated-ventricose, occasionally with branches or lobate. Terminal cells near pileus center (15–)29–39.8–50.5(–69) × (9–)13–17.4–21.5(–33) μm; clavate or obpyriform, occasionally also shaeropedunculate, subglogose or ellipsoid, rarely lobate, often with narrowed flexuous basal part; subterminal cells usually unbranched, mainly cylindrical, occasionally fusiform-ventricose, sometimes with lateral projections or branches. ***Caulocystidia*** (18–)22.5–32.3–42(–61) × (3–)4–5.3–6.5(–10) μm; mainly narrowly clavate, occasionally cylindrical, usually fasciculated, erect or ascending; thin-walled or slightly thickened near septa up to 0.5 μm, with brownish parietal pigments. ***Clamp connections*** present.

###### Distribution and ecology.

Known from France, Germany, Slovakia and United Kingdom; in semi-natural grasslands on calcareous soil, including one alpine site.

###### Additional material studied.

France • Pyrénées Atlantiques, Portalet, coord. 42°48'60"N, 00°24'54"E, terrestrial in pasture, 7 Oct 2022, S. Adamčík (SAV F-22283). Slovakia • Kremnické vrchy Mts., pasture 0.5 km W of Tajov, elev. 600 m, coord. 48°44'54"N, 19°03'31"E, terrestrial, 24 Oct 2020, S. Adamčík (SAV F-20808); • ibid., 25 Oct 2020, M. Caboň (SAV F-20849).

###### Notes.

*Dermolomajosserandii* is a member of D.subgenusAmylospora, section Atrobrunnea. Within the section, this species forms a distinct clade with two European and two North American taxa (Fig. 2). European members of the clade have a distinct morphology; their basidiomata are sturdy and reminiscent of small individuals of *Tricholoma*, but the pileus size is relatively small. The sister species to *D.josserandii* is *D.pseudojosserandii*, which is also very similar in morphology, as they both have pale-colored lamellae and stipes and also pale brown pilei when dry. This coloration distinguishes them from the related and similar *D.compactum*. *Dermolomajosserandii* is distinguished from its sister species by more distant lamellae (L < 30), stipes sometimes with darker bases, sometimes longer spores (> 6.5 μm), and marginal cells up to 8 μm wide. The concept of the name was assigned based on morphology. Despite being among the oldest published *Dermoloma* names (Orton 1960), the species was not included in any previous phylogenetic study. *Dermolomajosserandii* was the name intended to replace the invalid name “Tricholoma (Dermoloma) hygrophorus” published by Josserand (1958) based on French material, but the author of this new name selected his own British collection as the type (Orton 1960). Later, Josserand (1970) published *D.hygrophorus* validly, providing it with a Latin description and selecting his collection as the type. The types of these two names, however, do not represent the same species. Both species are relatively large and pale colored (Figs 5, 6). We note that *D.hygrophorus* produces larger and pale cream- to almost white-colored basidiomata, more reminiscent of *Neodermoloma* than other *Dermoloma* species. Spores of the type specimen of *D.josserandii* were amyloid and on av. 6.4 × 4.4 μm in size, which is similar to the spore sizes of several other *Dermoloma* species. These relatively sturdy, large and pale-colored basidiomata correspond to a clade of species that are unique within D.subgenusAmylospora. Within this clade we assigned the name *D.josserandii* to a species with distant lamellae, in agreement with its original description (Figs 2, 5). Because the type sequencing failed, and we managed to obtain only ITS from the French collection closest to the type collecting area (United Kingdom), we selected a German specimen SAV F-20907 as the epitype, which is provided by all six DNA regions and has multiple basidiomata.

##### ﻿ Dermoloma magicum

Taxon classificationAnimaliaAgaricalesTricholomataceae

Arnolds, Persoonia 17(4): 665. 2002.

66D2FF99-2340-518B-9CAD-DDBE044B937E

463376

Figs 35c, d , 37

###### Holotype.

Netherlands • Limburg, Epen, Cotessen, 21 Oct 1995, E. Arnolds *6701* (L).

###### Epitype

(designated here MBT10023011): Slovakia • Laborecká vrchovina Mts., pasture 1 km NW of Vyšná Jablonka, elev. 400–450 m, coord. 49°09'31"N, 22°06'18"E, terrestrial under *Pinussylvestris*, 21 Sep 2006, J. Terray (SAV F-4142).

###### Distinguishing characters.

European species; basidiomata large; context blackening where bruised; spores amyloid, narrowly ellipsoid to oblong, sometimes amygdaloid, 6.8–8 × 4.1–4.7 μm.

***Pileus*** (22–)29–43(–60) mm; convex to plane, indistinctly umbonate, sometimes lobate; margin indistinctly translucently striate when wet; surface smooth near margin, radially veined or rugulose near center, matt, hygrophanous; color near margin dark brown (6F5), brown (5E3) to grayish brown (5D3), when dry brownish ochraceous (5C3), brownish gray (5C2) to ochraceous-gray (5B2), near center dark brown (6F3, 6F5, 6F6), when dry brown (5E5), grayish ochraceous (5B3) to ochraceous-white (5A2). ***Stipe*** (35–)48–57(–70) × 4–12(–17) mm; cylindrical, attenuated towards the base, flexuous especially near the base; surface finely longitudinally striate, pruinose near lamellae, towards the base fibrillose or squamulose; color near lamellae grayish ochraceous (5B3), ochraceous-gray (5B2) to almost white, near the base brown (5E5–5E8) to dark brown (6F4). ***Lamellae*** L = 24–31(–44), l = 1–7; 4–8 mm wide; adnate-emarginate and decurrent with tooth; color ochraceous-white (5A2), ochraceous-gray (5B2) or brownish gray (5C2); edges irregular, sometimes with darker granules near stipe. ***Context*** turning black in pileus, inside of stipe turning apricot (5B6) to golden yellow (5B7); when young compact, later fragile; odor indistinctly farinaceous.

**Figure 37.**
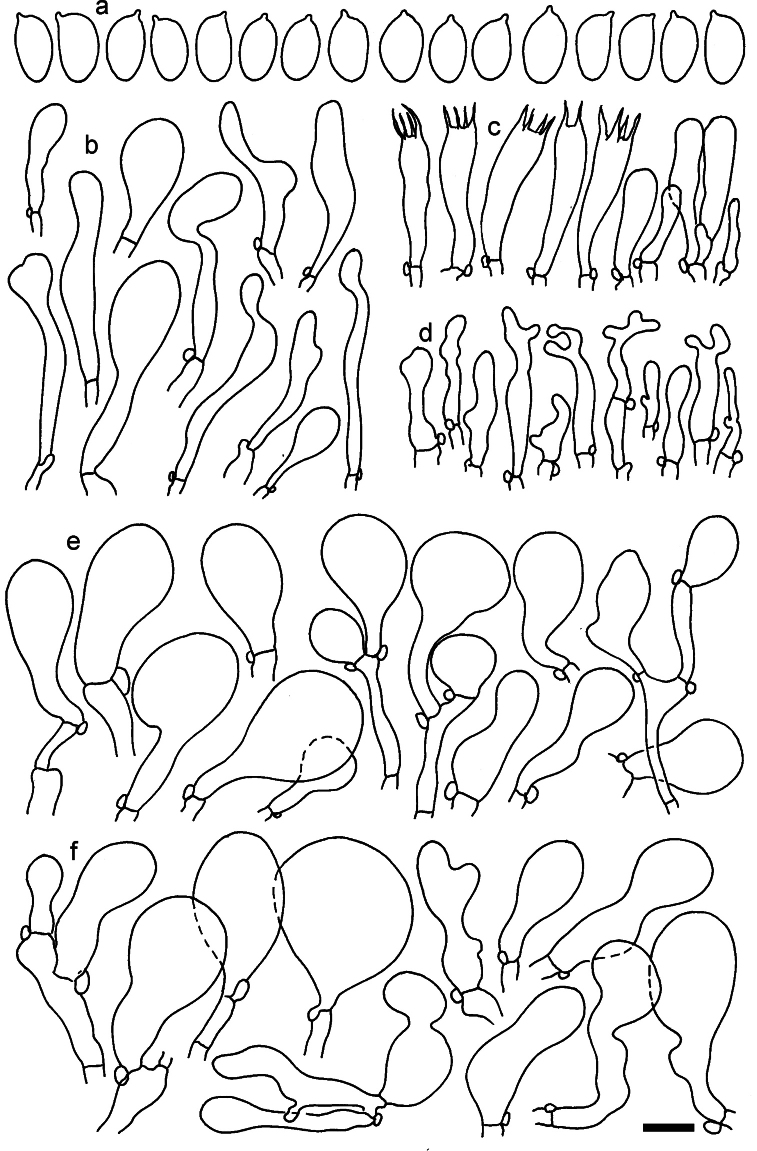
*Dermolomamagicum* (SAV F-4142, epitype), microscopic elements. **a** Spores; **b** caulocystidia; **c** basidia and basidioles; **d** marginal cells; **e** pileipellis elements near the pileus margin; **f** pileipellis elements near the pileus center. Scale bar: 5 µm for spores and 10 µm for other elements.

***Spores*** (6.2–)6.8–7.4–8(–9.4) × (3.6–)4.1–4.4–4.7(–5.3) μm; narrowly ellipsoid to oblong, sometimes amygdaloid, Q = (1.43–)1.57–1.69–1.80(–1.96); walls amyloid, sometimes dextrinoid; hilar appendage ca. 0.5–1.5 μm long. ***Basidia*** (25–)29–33.1–37(–42) × (6–)6.9–7.6–8.2(–9) μm; clavate, flexuous near the base; with 4 sterigmata, towards edges also with 1 or 2 sterigmata. ***Basidioles*** first cylindrical, then clavate, ca. 2.5–7 μm wide. ***Marginal cells*** (12–)16.5–21.7–27(–37) × (3–)3.5–4.5–5.5(–6.5) μm; cylindrical, lageniform or lageniform-clavate, apically often nodulose, diverticulate or furcate, flexuous. ***Pileipellis*** 45–70 μm deep; suprapellis of mainly one, occasionally two layers of inflated cells; subpellis 18–35 μm deep, hardly defined, of densely packed, irregularly oriented, intricate, 3–10(–15) μm wide hyphae, gradually passing to horizontally oriented hyphae in trama; hyphal terminations with brownish parietal pigments, thin-walled, hyphae in subpellis often with thickened walls up to 1 μm and locally with dark incrusted pigments. Terminal cells near pileus margin (14–)27–38.1–49(–78) × (8–)11.5–15.8–20.5(–35) μm; usually clavate, sphaeropedunculate or obpyriform, rarely ellipsoid, sometimes lobate; subterminal cells usually narrower, often branched, cylindrical and occasionally with lateral swellings or lobate. Terminal cells near pileus center (14–)24–35.6–47.5(–71) × (6–)9–13.1–17.5(–31) μm; usually clavate or sphaeropedunculate, rarely obpyriform, sometimes flexuous or lobate; subterminal cells narrower or occasionally equally wide, often branched, often with lateral swellings, projections or irregularly lobate. ***Caulocystidia*** (19–)27–35.9–45(–78) × (3–)5–7.1–9.5(–13) μm; sphaeropedunculate, clavate or cylindrical, flexuous, clustered in loose fascicules and repent but locally in dense fascicules and ascending or erect; usually with slightly thickened walls up to 0.5 μm, with brownish parietal pigments but locally also dark brown, towards septa usually with brownish but locally dark brown to black incrustations. ***Clamp connections*** present.

###### Distribution and ecology.

Known from France, Germany, Slovakia and United Kingdom; in semi-natural grasslands on calcareous soil.

###### Additional material studied.

Croatia • Crni Lug, Gorski kotar area, coord. 45°25'02"N, 14°42'22"E, mowed grassland mostly with short grass, 4 Oct 1998, A. Mešić (CNF 5/198). Slovakia • Oravské Beskydy Mts., Oravská Polhora, 2 km E of Slaná voda, around the peatbog, elev. 745–755 m, coord. 49°31'40"N, 19°28'23"E, terrestrial in semi-natural grassland, 21 Jul 2020, M. Caboň (SAV F-20618). United Kingdom • Wales, Pembrokeshire, Somerton farm, coord. 51°39'83"N, 04°59'49"E, 14 Oct 2019, pasture, D. Harries *DH 19-10* (SAV F-23434); • Wales, Plas Tirion, 22 Sep 2004, G. Griffith *GG220904* (ABS); • Wales, Powys, Gregynog grounds, coord. 52°34'04"N, 03°21'06"E, terrestrial in lawn, 19 Oct 2014, S. Adamčík (SAV F-4347).

###### Notes.

*Dermolomamagicum* has amyloid spores and basidiomata bruising black where handled, characters that define D.sectionNigrescentia. It is the only known member of the section and because of the unique blackening context it is easily distinguishable from the other *Dermoloma* species. The species was included in the phylogenetic study by Sánchez-García et al. (2021) and correctly labeled as the only member of D.sectionNigrescentia. Both multilocus phylogenies in this study do not support this species as a member of D.subgenusAmylospora. The species name *D.magicum* was assigned based on morphological match. During the preparation of the manuscript we managed to receive a partial ITS sequence of the type collection, which is not included in the ITS tree (Suppl. material 8), but does match other sequences assigned to the species and confirms the concept presented here. Spores of the type collections (av. 6.7 × 4.1 μm) were slightly smaller than in other more recent collections, but the range established by Arnolds (2002) perfectly matches our observations (Suppl. material 7). A Slovak collection was selected as epitype because it is perfectly documented and is the only collection represented by all six genes in the phylogeny.

##### ﻿ Dermoloma murinellum

Taxon classificationAnimaliaAgaricalesTricholomataceae

Horak, Sydowia 39: 110. 1987.

781DE58C-7DC4-5F57-85D1-5E964EFAFB4F

131362

Figs 35e , 38

###### Holotype.

Switzerland • Graubünden, N des Albulpasses (Terrassas), elev. 2450 m, 30 Aug 1982, E. Horak *ZT1573* (ZT Myc 42786).

###### Distinguishing characters.

European species; basidiomata small, mycenoid; pilei 8–22 mm in diameter; stipes 1–2 mm wide; lamellae ash-gray; spores amyloid, ellipsoid to oblong, 5.8–6.7 × 3.7–4.4 μm; caulocystidia narrowly clavate or narrowly fusiform, 4–6 μm wide.

***Pileus*** 8–22 mm; when young conical-campanulate, when mature plano-convex with broad, rounded umbo; margin shortly enrolled, entire to slightly crenulate; surface matt, rugulose, densely minutely wrinkled, strongly hygrophanous; color when young or wet very dark gray-brown to almost black, when mature fading to dull ochre-gray with some lilac shade at center. ***Stipe*** 15–35 × 1–2 mm; cylindrical, flexuous to undulate-bumped; surface densely pruinose when young, remaining pruinose only at apex when mature; color blackish gray all over, with age fading to ochraceous gray-brown from apex towards the base. ***Lamellae*** distant, L = 12–16, l = 1–3; adnate-emarginate; color ash-gray, when mature rather lighter yellowish ochre; edges truncate, concolorous, entire. ***Context*** whitish to somewhat flesh-colored in pileus, gray-brown in stipe; odor farinaceous.

**Figure 38.**
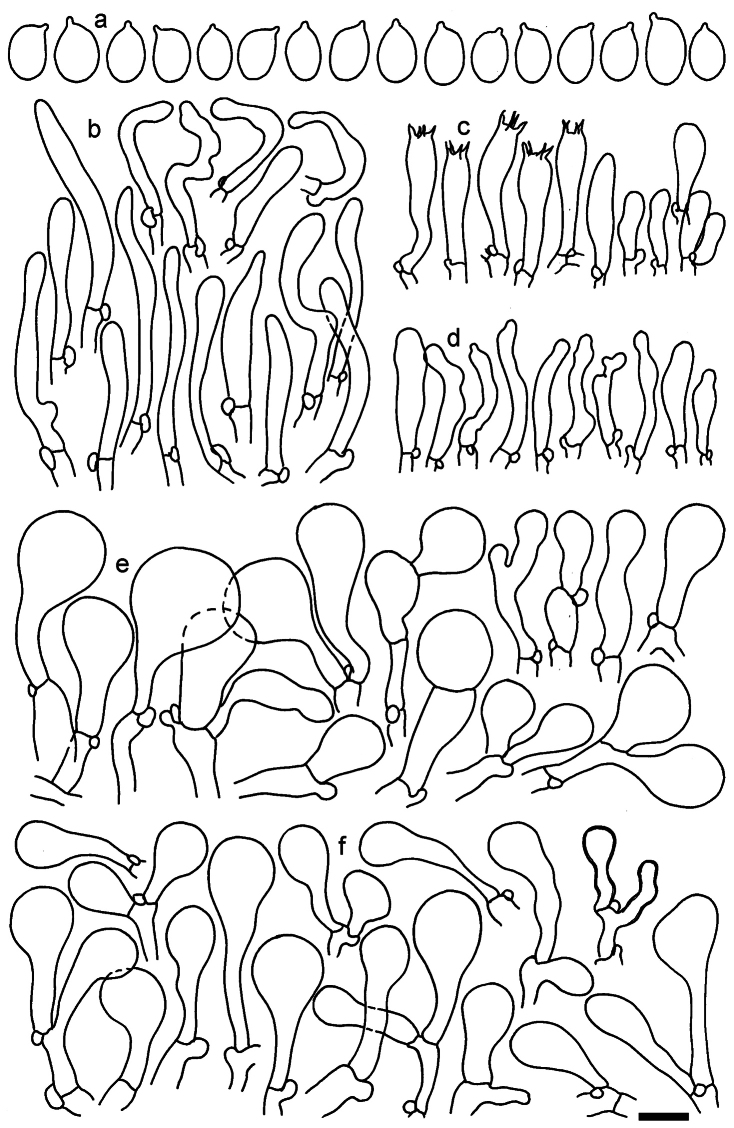
*Dermolomamurinellum* (ZT Myc 42786, holotype), microscopic elements. **a** Spores; **b** caulocystidia; **c** basidia and basidioles; **d** marginal cells; **e** pileipellis elements near the pileus margin; **f** pileipellis elements near the pileus center. Scale bar: 5 µm for spores and 10 µm for other elements.

***Spores*** (5.5–)5.8–6.2–6.7(–7.8) × (3.4–)3.7–4–4.4(–4.8) μm; ellipsoid to oblong, Q = (1.34–)1.41–1.55–1.69(–1.86); walls amyloid; hilar appendage ca. 0.5–1 μm long. ***Basidia*** (21–)25–28.7–32(–40) × 6–6.5–7.5 μm; clavate; with 2 or 4 sterigmata. ***Basidioles*** first cylindrical, then clavate, ca. 4–6.5 μm wide. ***Marginal cells*** (17–)20–24.7–29.5(–40) × (3–)3.5–4.8–6(–7) μm; clavate, cylindrical or fusiform, often flexuous, occasionally moniliform, sometimes diverticulate or nodulose, apically obtuse or constricted. ***Pileipellis*** 65–100 μm deep; suprapellis 40–65 μm deep, of one or two layers of inflated, densely arranged cells; subpellis not well-differentiated, 20–30 μm deep, of densely packed, horizontally oriented, intricate, 3–10 μm wide hyphae, gradually passing to horizontally oriented hyphae in trama; hyphal terminations with yellow-brown parietal pigments but on subterminal cells also incrusted pigments, with usually slightly or distinctly thickened walls up to 1 μm. Terminal cells near pileus margin (13–)25.5–36.6–47.5(–60) × (6–)10–20.3–30(–44) μm; obpyriform, sphaeropedunculate or clavate, occasionally lobate; subterminal cells narrower and subcylindrical or ventricose-fusiform, occasionally laterally swollen, often branched. Terminal cells near pileus center (14–)21.5–34.8–48(–73) × (7–)11.5–20.5–29.5(–45) μm; mainly clavate, occasionally sphaeropedunculate or obpyriform, towards septa often narrowed and flexuous; subterminal cells more frequently branched. ***Caulocystidia*** (26–)31.5–38.8–40(–53) × (3–)4–5–6(–6.5) μm; cylindrical, narrowly clavate, occasionally narrowly fusiform, apically obtuse or slightly acute or constricted, often flexuous, occasionally nodulose; thin-walled, with brownish yellow parietal pigments. ***Clamp connections*** present.

###### Distribution and ecology.

This species is only known from the type collecting area in Swiss Alps; probably restricted to alpine habitats, but insufficiently known.

###### Additional material studied.

Switzerland • Albulapass (Graubünden), alpine grassland under *Cirsiumspinosissimum*, elev. 2300 m, 2 Sep 2006, A. Leuchtmann and P.-A. Moreau *PAM06090203* (LIP).

###### Notes.

*Dermolomamurinellum* has amyloid spores and belongs to D.subgenusAmylospora, section Atrobrunnea. It has small mycenoid basidiomata but is placed within a clade of species with mainly collybioid basidiomata (Fig. 2). The species exhibits almost all characters with transitional stages between morphological groups defined in this study and is therefore very difficult to identify. The basidiomata are at the upper limit of the mycenoid size (pilei up to 15 mm, stipes up to 2 mm wide), the spores are of intermediate size and shape in D.sectionAtrobrunnea, the marginal cells are not well differentiated, and the caulocystidia are relatively slender. Morphological identification requires special attention; we therefore recommend to combine the heat map (Fig. 4), the key and other data available in this study to achieve a better probability of correct identification, and in case of uncertainty, an ITS sequence should be used for verification. *Dermolomamurinellum* is the only species of the genus described from the cold alpine habitat of the European Alps (Switzerland) (Horak 1987). Our collection LIP (PAM0690203) was included in the phylogenetic study by Sánchez-García et al. (2021). It is originating from the type collecting area and our morphological study confirmed that it matches the type of *D.murinellum*. It was collected in alpine grasslands, but according to our observations it seems that several species from lowlands can occur at higher elevation in the alpine habitat. For example, we collected *D.compactum*, *D.griseobasale* and *D.josserandii* in the Pyrenees at an elevation of ca. 1800 m. Furthermore, the Albulapass locality is not far from timberline and mixed alpine and subalpine influences with, e.g., *Amanitapantherina* and *Boletusedulis* associated with herbaceous vegetation (A. Leuchtmann, pers. comm.).

##### ﻿ Dermoloma obscurum

Taxon classificationAnimaliaAgaricalesTricholomataceae

Caboň & Jančovič. sp. nov.

8C771F66-B48C-5A1F-B496-FDA13C0FC4F0

856440

Figs 35f , 39a , 40

###### Etymology.

In reference to the basidiomata with dark colors, especially on the lamellae.

###### Holotype.

Italy • Toscana, 1.3 km SE of Frassine, elev. 260–270 m, coord. 43°06'15"N, 10°46'45"E, terrestrial, wooded pasture, 10 Nov 2016, S. Adamčík (SAV F-20012).

###### Diagnosis.

European species; basidiomata small, dark, gray and brown colored; pilei 7–12 mm in diameter; stipes 1–2 mm wide; lamellae brown to dark brown when young and fresh, pallescent with drying; spores amyloid, broadly ellipsoid to ellipsoid, 5.4–6.4 × 4.2–4.8 μm; caulocystidia up to 6.5 μm wide, clavate or cylindrical.

***Pileus*** (4–)7–12(–18) mm; young semiglobose, mature convex, expanding to plane, rarely lobate; margin translucently striate to half of the radius when wet; surface smooth, sometimes rugulose or rough near center, hygrophanous; color when young dark brown (7F3), when mature near margin dark brown (6F4, 6F5), grayish brown (6E3), when dry brown (6E4), grayish brown (6E3) to ochraceous-gray (6D2), near center dark brown (6F3, 6F4, 6F5) to almost black, when dry dark brown (6F4), brown (6E4) to grayish brown (5D3). ***Stipe*** (15–)20–33(–45) × 1–2 mm; cylindrical, sometimes narrowed towards the base, flexuous; surface indistinctly longitudinally striate, when young pruinose, later towards the base or completely smooth and shiny; color near lamellae brownish gray (6E2), grayish brown (6D3, 6E3) to dark brown (6F3, 6F4), near the base dark brown (6F4, 6F5, 7F5) to black. ***Lamellae*** L = (14–)17–22, l = (0–)1–3; 2–3 mm wide; adnate-emarginate and sometimes decurrent with tooth; color brown (6E3) to dark brown (6F3, 6F2), when old and dry ochraceous-gray (6C2) to yellowish gray (4B2); edges entire. ***Context*** when young elastic, later fragile; odor farinaceous.

**Figure 39.**
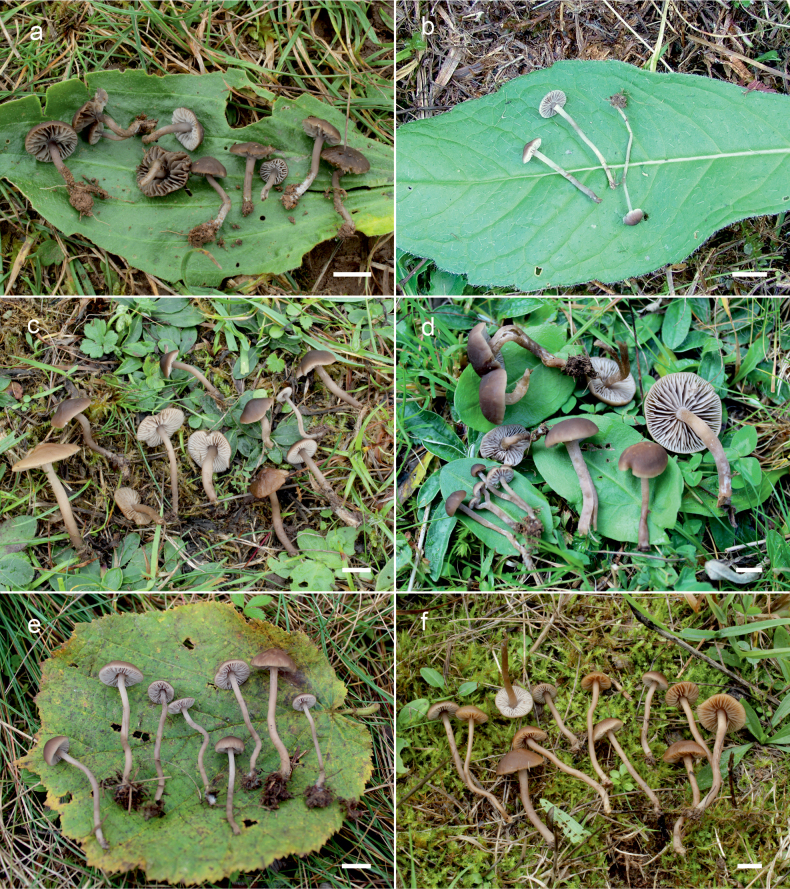
Basidiomata of *Dermoloma* in field appearance. **a***Dermolomaobscurum* (SAV F-20553), photo S. Jančovičová; **b***Dermolomaparvisporum* (SAV F-20229, holotype), photo S. Jančovičová; **c***Dermolomaphaeopodium* (SAV F-20546), S. Jančovičová; **d***Dermolomaphaeopodium* (SAV F-22208), photo S. Jančovičová; **e***Dermolomapruinosipes* (SAV F-20834, holotype), photo S. Jančovičová; **f***Dermolomapruinosipes* (SAV F-20504), photo S. Jančovičová. Scale bar: 10 mm.

**Figure 40.**
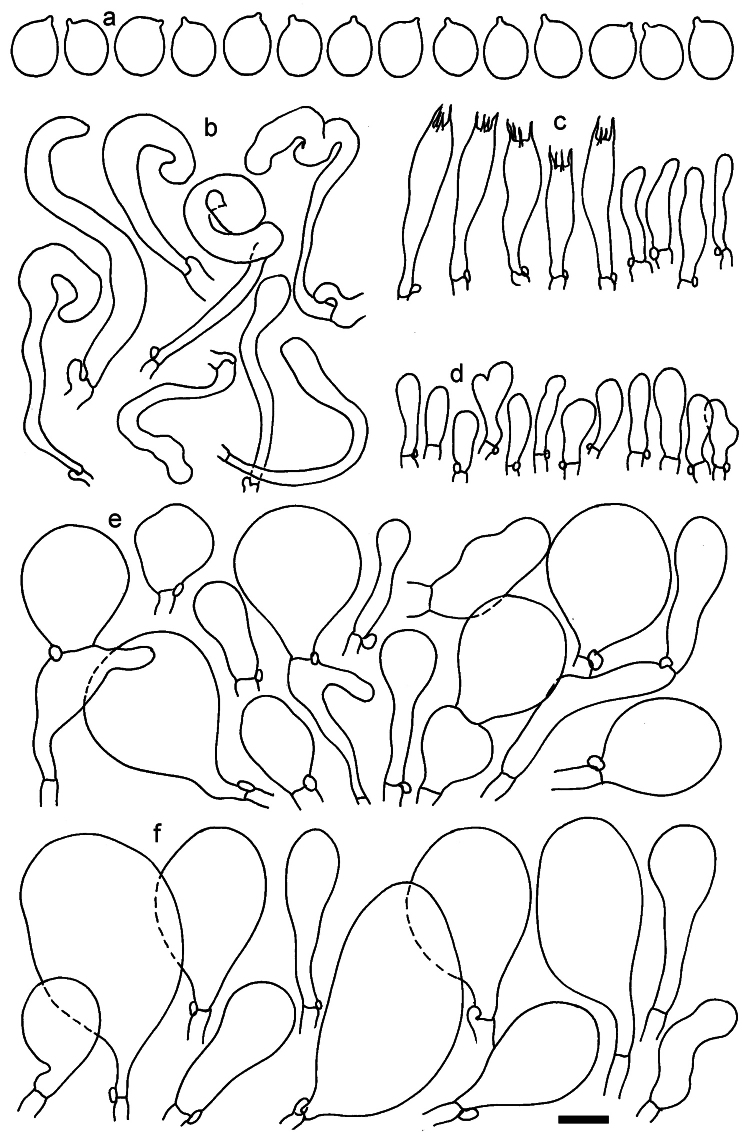
*Dermolomaobscurum* (SAV F-20013), microscopic elements. **a** Spores; **b** caulocystidia; **c** basidia and basidioles; **d** marginal cells; **e** pileipellis elements near the pileus margin; **f** pileipellis elements near the pileus center. Scale bar: 5 µm for spores and 10 µm for other elements.

***Spores*** (4.9–)5.4–5.9–6.4(–7.3) × (3.7–)4.2–4.5–4.8(–5.3) μm; broadly ellipsoid to ellipsoid, Q = (1.12–)1.20–1.31–1.42(–1.66); walls amyloid, sometimes thick-walled and dextrinoid; hilar appendage ca. 0.5–1.5 μm long. ***Basidia*** (20–)23.5–26.6–30(–39.5) × (4.5–)6–6.4–7(–8) μm; clavate; usually with 4 sterigmata, sometimes with both, 4 and 2 sterigmata, one collection (SAV F-20012) with only 2 sterigmata. ***Basidioles*** first cylindrical, then clavate, ca. 2–6.5 μm wide. ***Marginal cells*** (12–)14–19.7–29(–47) × (3.5–)4.5–6.4–8(–12) μm; not well-differentiated, clavate, rarely cylindrical. ***Pileipellis*** 70–85 μm deep; suprapellis 40–50 μm deep, of mainly one or two layers of inflated, densely arranged cells; subpellis well-differentiated, 30–45 μm deep, of densely packed, irregularly oriented, puzzled, 3–10(–15) μm wide hyphae gradually passing to horizontally oriented hyphae in trama; hyphal terminations with brownish yellow parietal pigments, locally also with brown incrusted pigments, usually with thick walls up to 1 μm, near septa of terminal cells and in upper part of subpellis with more thickened walls up to 2 μm. Terminal cells near pileus margin (15–)25.5–36.2–47(–82) × (6.5–)13–18.2–23.5(–31) μm; usually obpyriform or clavate, sometimes sphaeropedunculate, rarely lageniform, ellipsoid or subglobose; subterminal cells usually narrower and subcylindrical, occasionally inflated and fusiform, often branched. Terminal cells near pileus center (17–)26–36.6–46.9(–73) × (8–)12.5–17.9–23(–33.5) μm; similar to cells near margin; subterminal cells similar to cells near margin. ***Caulocystidia*** (15–)21–34.3–47.5(–70) × (2–)3.5–5.1–6.5(–9) μm; clavate or cylindrical, usually not or only slightly flexuous, repent, dispersed, individual or in small fascicules; often with slightly thickened walls up to 0.5 μm especially near septa, hyaline. ***Clamp connections*** present.

###### Distribution and ecology.

Known from Croatia, France, Germany, Italy, The Netherlands and Slovakia; in semi-natural grasslands on calcareous soil.

###### Additional material studied.

Croatia • 1.2 km NE of Gornja Supetarska Draga, Rab island, pasture with sheep, coord. 44°48'01"N, 14°44'40"E, edge of *Pinushalepensis* and *Quercusilex* forest, 7 Oct 2007, M. Čerkez (CNF 1/4830). France • Tarn-et-Garonne, military camp of Caylus, coord. 44°18'53"N, 01°43'47"E, calcareous dry semi-natural grassland, 14 Oct 2013, C. Hannoire *CH13101428* (BBF, as *D.phaeopodium*). Germany • Rheinland-Pfalz, Heimberg, elev. 265 m, coord. 49°48'37"N, 07°44'06"E, terrestrial in semi-natural grassland, 10 Nov 2019, S. Adamčík (SAV F-20549); • ibid., 10 Nov 2019, F. Hampe (SAV F-20553); • Rheinland-Pfalz, Horbach, elev. 370 m, coord. 49°49'45"N, 07°31'17"E, terrestrial in semi-natural grassland, 8 Nov 2019, S. Adamčík (SAV F-20519). Italy • Toscana, 1.3 km SE of Frassine, elev. 260–270 m, coord. 43°06'15"N, 10°46'45"E, terrestrial, wooded pasture, 10 Nov 2016, M. Caboň (SAV F-20013). The Netherlands • Pfalz, Duitsland, Ebenberg, poor grassland, 2 Oct 2013, E. Arnolds *Arnolds 13-21* (L, as *D.phaeopodium*). Slovakia • Biele Karpaty Mts., 1.5 km E of Nová Bošáca, Blažejová Natural Monument, elev. 415 m, coord. 48°52'33.3"N, 17°49'03.4"E, terrestrial among grass, 30 Jul 2005, V. Kautman (SAV F-4144).

###### Notes.

*Dermolomaobscurum* belongs to D.subgenusAmylospora, section Atrobrunnea. It has small mycenoid basidiomata and typically very dark gray to black colors with few brown components which distinguishes it from similar *Dermoloma* species. It forms a well-supported clade with other species with small basidiomata described or collected from the Mediterranean area, including *D.pusillum*, *D.clavicystis* and an unnamed *Dermoloma* species (Fig. 2). From the two described species it differs by the narrower caulocystidia. Unlike other closely related species, *D.obscurum* was collected both in the Mediterranean and temperate areas of Central Europe. The species was included in the phylogenetic study by Sánchez-García et al. (2021) as “D.cf.pusillum”.

##### ﻿ Dermoloma parvisporum

Taxon classificationAnimaliaAgaricalesTricholomataceae

Adamčík & Dima sp. nov.

BDBADB54-960B-52CD-B0CA-AE76A2FBDE5A

856441

Figs 39b , 41

###### Etymology.

Referring to the small spores.

###### Holotype.

Slovakia • Poloniny Mts., 4 km N of Stakčín, pastures above the water reservoir Starina, elev. 380–420 m, coord. 49°02'43"N, 22°14'56"E, terrestrial among grass, 25 Sep 2017, S. Adamčík (SAV F-20229).

###### Diagnosis.

European species; basidiomata small, mycenoid; pilei 6–8 mm in diameter, not striate near margin; stipes up to 1.5 mm wide; lamellae brown to dark brown; spores amyloid, small, 4.8–5.4 × 3.5–4 μm; marginal cells with attenuated, long, flexuous terminal parts; caulocystidia 8.5–13.5 μm wide, clavate, obpyriform, sphaeropedunculate, flexuous, nodulose-lobate.

***Pileus*** 6–8 mm; convex, sometimes indistinctly umbonate; margin not striate; surface smooth, matt, pruinose; color near margin dark brown (paler than 6F6), near center dark brown to black (7F4). ***Stipe*** 24–33 × 0.75–1.25 mm; cylindrical, flexuous especially near the base; surface finely longitudinally striate, near lamellae finely pruinose, bellow glabrous; color near lamellae brown (7E3, 7E4) to dark brown (7F5), near the base dark brown (7F5). ***Lamellae*** L = 17–25, l = 1–3; up to 1.5 mm wide; adnate; color brown (7E3) to dark brown (paler than 7F3); edges entire. ***Context*** fragile; odor indistinct.

**Figure 41.**
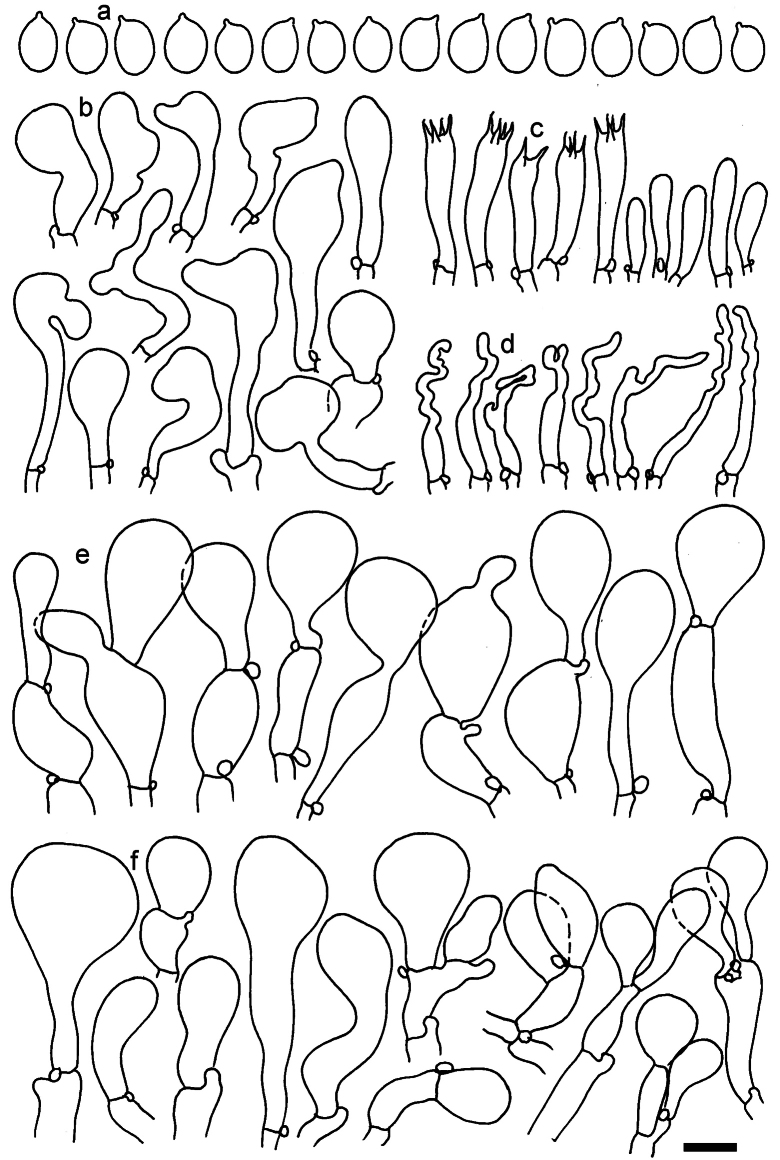
*Dermolomaparvisporum* (SAV F-20229, holotype), microscopic elements. **a** Spores; **b** caulocystidia; **c** basidia and basidioles; **d** pileipellis elements near the pileus margin; **e** pileipellis elements near the pileus center. Scale bar: 5 µm for spores and 10 µm for other elements.

***Spores*** (4.6–)4.8–5.1–5.4(–5.8) × (3.3–)3.5–3.8–4(–4.1) μm; broadly ellipsoid to ellipsoid, Q = (1.17–)1.29–1.36–1.44(–1.51); walls amyloid; hilar appendage ca. 0.5–1 μm long. ***Basidia*** (23–)25–26.6–28(–30) × (5.5–)6–6.2–6.5(–7) μm; clavate; mainly with 4, rarely with 2 sterigmata. ***Basidioles*** first cylindrical, then clavate, ca. 3–5.5 μm wide. ***Marginal cells*** (26–)27.5–36.4–45(–52) × 3.5–3.9–4.5(–5) μm; attenuated, with wider base, at apex with long, narrow (1.5–2 μm), flexuous or twisted, nodulose, intermingled or diverticulate projections, often with attached loose spores. ***Pileipellis*** 55–75 μm deep; suprapellis 35–45 μm deep, usually of one or two layers of inflated, densely arranged cells, subpellis not well differentiated, 20–30 μm deep, of densely packed, mainly horizontally oriented, intricate, 3–10 μm wide hyphae, gradually passing to horizontally oriented hyphae in trama; hyphal terminations with brownish parietal pigments, near septa of terminal cells and in subpellis with dark brown to black parietal and locally also incrusted pigments, walls only slightly thickened up to 0.5 μm especially in subpellis. Terminal cells near pileus margin (16–)20–27.8–35.5(–54) × (9.5–)12–15–18(–23) μm; sphaeropedunculate, obpyriform clavate or subglobose; subterminal cells usually narrower and rarely branched, fusiform-ventricose, sometimes with lateral swellings or lobate. Terminal cells near pileus center (15.5–)25–38.1–51.5(–68) × (9–)13.5–18.5–23.5(–32) μm; usually obpyriform or clavate, rarely sphaeropedunculate; subterminal cells usually narrower, usually narrowly fusiform or subcylindrical, often branched. ***Caulocystidia*** (19–)23–31.2–39.3(–52) × (6.5–)8.5–11–13.5(–18.5) μm; clavate, obpyriform, sphaeropedunculate, flexuous, nodulose-lobate, often clustered in small ascending fascicules, sometimes individual and repent; thin-walled, with faint brownish parietal pigments. ***Clamp connections*** present.

###### Distribution and ecology.

Known from two localities in Norway and Slovakia; in semi-natural or natural, dry grasslands; habitat preferences insufficiently known.

###### Additional material studied.

Norway • Oslo, Gressholmen, coord. 59°53'01"N, 10°43'16"E, in dry, natural, calcareous grassland, 7 Oct 2013, A. Molia and T. Læssøe *NOBAS2937-16* (O-F-21841).

###### Notes.

*Dermolomaparvisporum* belongs to D.subgenusAmylospora, section Atrobrunnea. It is easily distinguishable from similar species with small basidiomata due to its very small spores and marginal cells with attenuated and flexuous terminations that are often intermingled because of multiple projections that trap loose spores from the hymenium. We did not observe this in other *Dermoloma* species, but the phenomenon may be also present in species with projections on marginal cells like *D.pruinosipes*.

##### ﻿ Dermoloma phaeopodium

Taxon classificationAnimaliaAgaricalesTricholomataceae

P.D. Orton, Notes. R. bot. Gdn. Edinb. 28(2): 327. 1980.

9A09A00F-24DD-5259-B368-2E6BA3AFE5DE

111400

Figs 39c, d , 42

###### Holotype.

United Kingdom • Devon, Membury, Inter graminos, 28 Oct 1977, P. D. Orton *4905* (E16877).

###### Distinguishing characters.

European species; basidiomata medium to small, collybioid; pilei 8–25 mm in diameter, brown to dark brown when wet and light brown when dry; stipes 1–4 mm wide, towards bases darker brown; lamellae brownish gray, brownish ochraceous or grayish brown; spores amyloid, 5.6–6.5 × 3.5–4.2 μm; marginal cells mainly clavate and apically obtuse; caulocystidia 4.5–11.5 μm wide, variable in size and shape, often flexuous or twisted.

***Pileus*** (6–)8–25(–32) mm; convex to plano-convex, often broadly umbonate, sometimes weakly depressed or lobate; margin indistinctly translucently striate to half of the radius; surface smooth, often rugulose or rough near center, sometimes pitted, hygrophanous; color when young dark brown (7E4), near margin dark brown (6F5, 6F6, 6F7, 7F4, 8F3, 8F4), brown (6E4, 6E5), grayish brown (6E3), yellowish brown (5D5), when dry light brown (6D6), grayish brown (6D3), brownish ochraceous (6C3), grayish ochraceous (5B3, 5B4), near center darker or concolorous (5E5, 6E5, 6E7, 6F7, 7F4, 7F5, 7F6, 8F3) to black, when dry brown (6E5, 6E6), dark blond (5D4), brownish ochraceous (6C4). ***Stipe*** (20–)24–42(–46) × 1–4(–5) mm; cylindrical, narrowed towards the base, flexuous especially near the base; surface finely pruinose near lamellae, towards the base finely fibrillose or smooth; color near lamellae brownish ochraceous (5C3), grayish brown (5D3), dark blond (5D4), brownish gray (6C2), grayish brown (6D3, 6E3, 7E3), light brown (6D4), near the base grayish brown (6D3, 6E3), brown (6E4) to dark brown (6F4, 6F5, 7F4). ***Lamellae*** L = (15–)19–32, l = (0–)1–3(–7); 2–7 mm wide; adnexed or adnate-emarginate and decurrent with tooth; color brownish gray (6C2), brownish ochraceous (6C3), grayish brown (6D3, 6E3, 7D3, 7E3), near edges paler brownish gray (6C2, 7C2); edges entire. ***Context*** when young elastic, later fragile; odor farinaceous.

**Figure 42.**
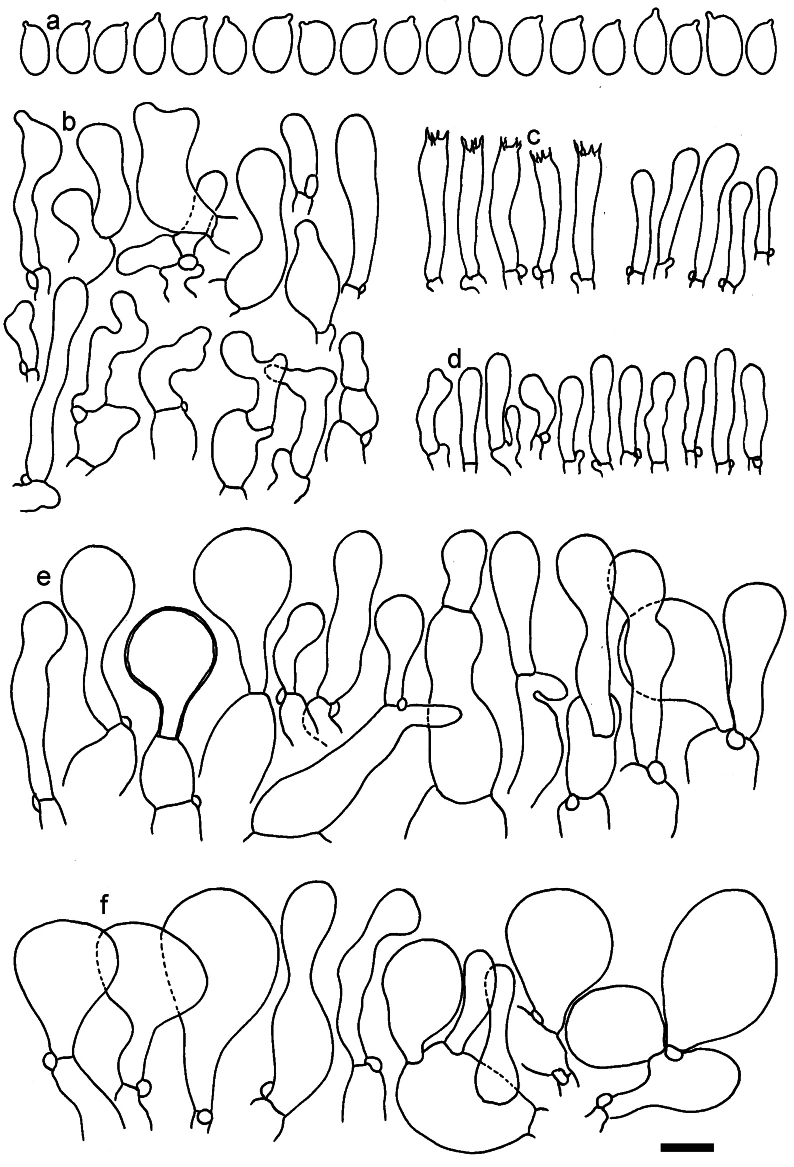
*Dermolomaphaeopodium* (E16877, holotype), microscopic elements. **a** Spores; **b** caulocystidia; **c** basidia and basidioles; **d** marginal cells; **e** pileipellis elements near the pileus margin; **f** pileipellis elements near the pileus center. Scale bar: 5 µm for spores and 10 µm for other elements.

***Spores*** (5–)5.6–6.1–6.5(–7.8) × (3.1–)3.5–3.9–4.2(–5.2) μm; ellipsoid to oblong, Q = (1.37–)1.46–1.57–1.68(–1.88); walls amyloid; hilar appendage ca. 0.5 μm long. ***Basidia*** (23–)25.5–28.3–31(–37) × (4.5–)5.5–6.3–7(–8) μm; clavate; with 4 sterigmata. ***Basidioles*** first cylindrical, then clavate, ca. 3–6 μm wide. ***Marginal cells*** (13–)16–21.1–26(–37) × (3.5–)4.5–6.2–8(–11) μm; clavate, occasionally cylindrical, occasionally flexuous or nodulose. ***Pileipellis*** 55–60 μm deep; suprapellis of mainly one, rarely two layers of inflated cells; subpellis well-defined, 20–25 μm deep, of densely packed, irregularly oriented, puzzled, 3–11 μm wide hyphae, not sharply delimited from horizontally oriented hyphae in trama; hyphal terminations with brownish parietal pigments, frequently with thickened walls up to 1 μm, in subpellis more thick-walled up to 1.3 μm, rarely with indistinct incrusted brownish pigments. Terminal cells near pileus margin (6.5–)27.5–35.5–43.5(–57) × (7.5–)13–18–23(–35) μm; usually clavate or sphaeropedunculate, rarely obpyriform, towards septa sometimes constricted, rarely with lateral projections; subterminal cells usually equally large or more voluminous (longer), ellipsoid or cylindrical, occasionally with lateral swellings or nodulous. Terminal cells near pileus center (11.5–)24.5–33.6–42.5(–60) × (7–)14–19.1–24(–32) μm; obpyriform, sphaeropedunculate, occasionally clavate ellipsoid or subglobose; subterminal cells narrower, equally wide or wider, cylindrical, obpyriform, ellipsoid, occasionally branched, often with lateral swellings. ***Caulocystidia*** (12–)22.5–32.7–42.5(–64) × (3–)4.5–7.9–11.5(–19) μm; very variable, clavate, fusiform, cylindrical, rarely lageniform, flexuous to curved or twisted, often moniliform, often nodulose or lobate, apically obtuse but occasionally constricted, often clustered in small ascending fascicules, sometimes individual and repent; usually with slightly thickened walls up to 0.5 μm, with brownish parietal pigments. ***Clamp connections*** present.

###### Distribution and ecology.

Known from Croatia, Germany, Italy, Slovakia and United Kingdom; in grasslands, preferably on alkaline soil.

###### Additional material studied.

Croatia • Mljet Island, Mljet National Park, 750 m SW/W-SW of Pomena village, coord. 42°47'02"N, 17°20'09"E, grassland with *Cistus* sp. and *Oleaeuropaea*, 14 Nov 2010, A. Mešić (CNF 1/6130). Germany • Baden-Württemberg, Justingen, Schachenheide, coord. 48°24'35"N, 09°40'25"E, terrestrial in semi-natural grassland, 2 Oct 2021, S. Adamčík (SAV F-20859); • ibid., 2 Oct 2021, S. Adamčík (SAV F-20865); • Rheinland-Pfalz, Heimberg, elev. 265 m, coord. 49°48'37"N, 07°44'06"E, terrestrial in semi-natural grassland, 10 Nov 2019, S. Adamčík (SAV F-20541); • ibid., 10 Nov 2019, S. Adamčík (SAV F-20545); • ibid., 10 Nov 2019, S. Adamčík (SAV F-20546); • ibid., 10 Nov 2019, S. Adamčík (SAV F-20548); • Gampenstein, Hainewalde, coord. 50°55'14"N, 14°43'22"E, loamy, ancient, semi-natural grassland, 27 Sep 2022, A.Karich and R. Ullrich *IHI-22Der02* (GLM-F137755). Italy • Prato, Piani et Pragho, 8 Oct 2013, F. Bocianolo *G3112* (GDOR). Slovakia • Zvolenská kotlina Basin, pasture E of Bečov, elev. 400–450 m, coord. 48°38'48"N, 19°14'49"E, terrestrial, 28 Aug 2014, S. Adamčík (SAV F-4249). Spain • Pyrénées Mts., Canfranc, Río de la Canal Roya, coord. 42°46'26"N, 00°30'56"E, terrestrial under *Buxus* sp. and *Pinussylvestris*, 3 Oct 2022, S. Adamčík (SAV F-22208).

###### Notes.

*Dermolomaphaeopodium* is a member of D.subgenusAmylospora, section Atrobrunnea. It belongs to an inclusive clade of species with mainly collyboid basidiomata such as *D.curvicystidiatum*, *D.griseobasale*, *D.confusum* and *D.pruinosipes* (Fig. 2). In the field it is very difficult to distinguish it from other members of this clade, and it is especially similar to *D.confusum* (see notes under to that species). We recommend to combine the key with the heat map (Fig. 4) and other data available in this publication to get a better probability of correct identification, or to verify it with sequence data of the ITS region. The concept of *D.phaeopodium* is based on the position of the type sequence and was already recognized correctly by Sánchez-García et al. (2021).

##### ﻿ Dermoloma pruinosipes

Taxon classificationAnimaliaAgaricalesTricholomataceae

Corriol & Jančovič. sp. nov.

11A53DA4-BA0B-5C5F-8E35-B3ECA09C62B4

856442

**Figure 43.**
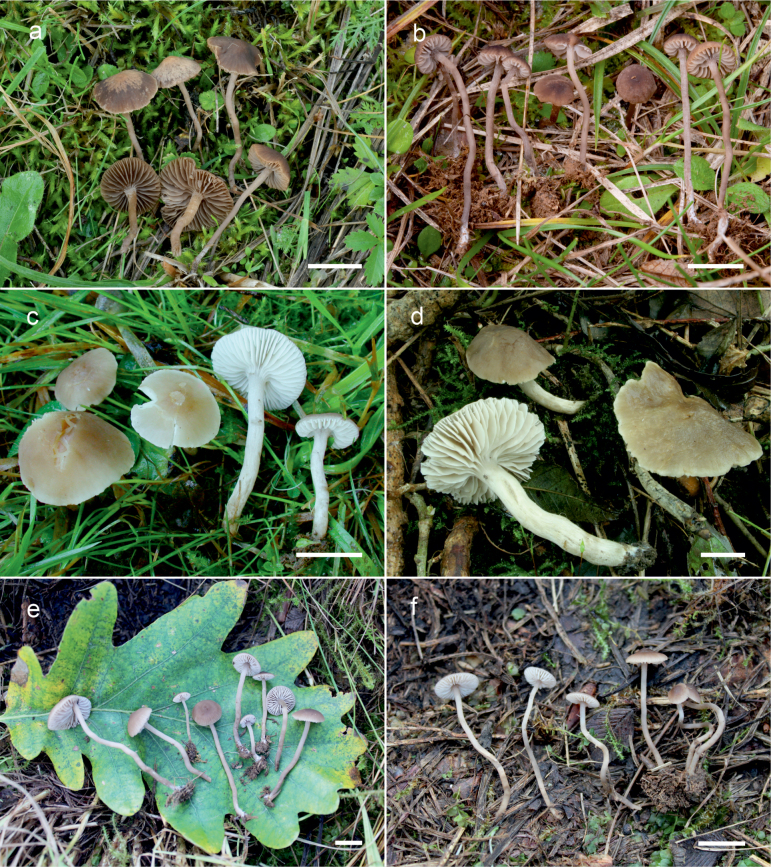
Basidiomata of *Dermoloma* in field appearance. **a***Dermolomapruinosipes* (GLM-F137757), photo A. Karich; **b**Dermolomaaff.pruinosipes, (SAV F-23429), photo G. Friebes; **c***Dermolomapseudojosserandii* [BBF (*CH17100504*)], holotype, photo C. Hannoire; **d***Dermolomapseudojosserandii* [BBF (*GC17092306*)], photo G. Corriol; **e***Dermolomarostratum* (SAV F-20811, holotype), photo S. Jančovičová; **f***Dermolomarostratum* (SAV F-20817), photo S. Jančovičová. Scale bar: 10 mm.

**Figure 44.**
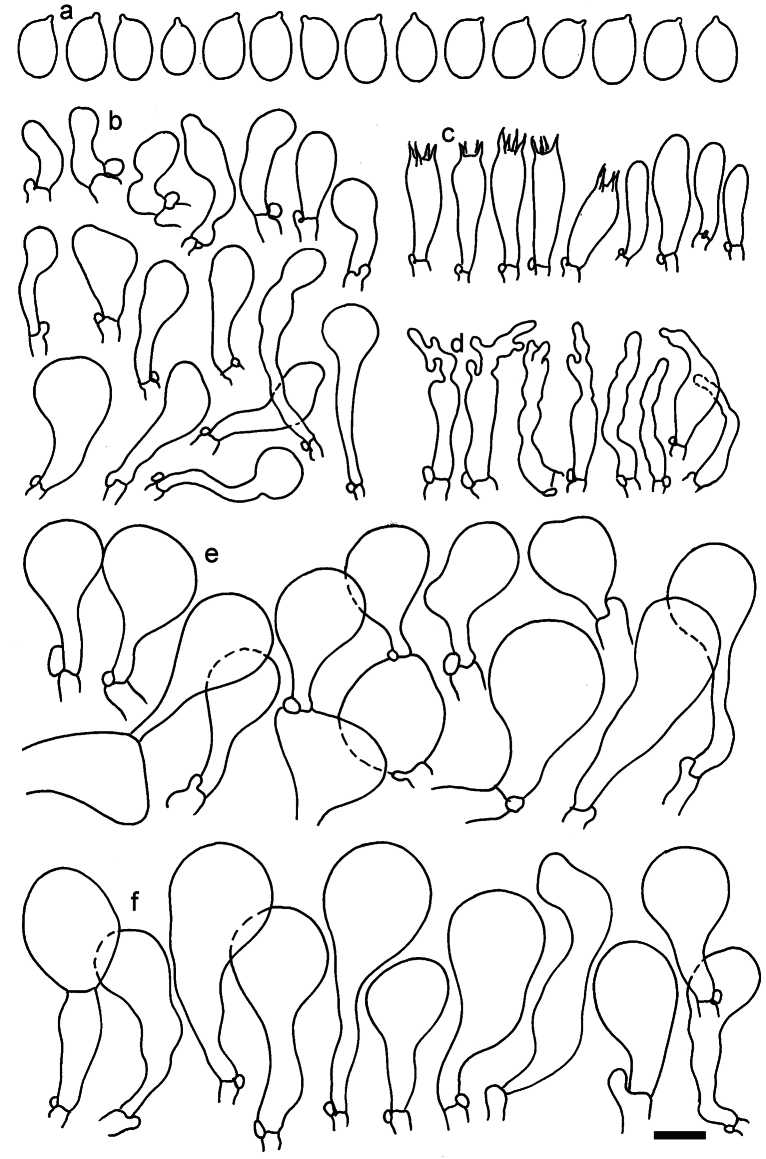
*Dermolomapruinosipes* (SAV F-20230), microscopic elements. **a** Spores; **b** caulocystidia; **c** basidia and basidioles; **d** marginal cells; **e** pileipellis elements near the pileus margin; **f** pileipellis elements near the pileus center. Scale bar: 5 µm for spores and 10 µm for other elements.

Figs 39e, f , 43a , 44

###### Etymology.

In reference to the distinctly pruinose stipe.

###### Holotype.

Slovakia • Kremnické vrchy Mts., pasture 0.5 km W of Tajov, elev. 600 m, coord. 48°44'54"N, 19°03'31"E, terrestrial, 25 Oct 2020, S. Jančovičová (SAV F-20834).

###### Diagnosis.

European species; basidiomata small, mycenoid or collybioid; pilei 9–20 mm, when wet translucently striate near margin and brown to dark brown, when dry light brown; stipes 1–3 mm wide, when young entirely pruinose; lamellae brownish ochraceous, brownish gray or grayish brown; spores amyloid, 6–7.1 × 3.9–4.4 μm; marginal cells lageniform, with long, narrow, flexuous, nodulose or diverticulate apical projections; caulocystidia, 5–18 μm wide, very variable in shape and size and often flexuous.

***Pileus*** (7–)9–20(–30) mm; convex, later plano-convex, sometimes indistinctly umbonate or broadly conical; margin translucently striate to half of the radius when wet; surface smooth near margin, usually radially rugulose or rough near center, hygrophanous and starting to discolor from center; color when young dark brown (6F4, 6F5), when mature near margin dark brown (6F4, 6F5, 6F6), sometimes grayish brown (6D3), when dry light brown (5D4), brownish ochraceous (5C3, 5C4, 6C4), grayish brown (5D3), grayish ochraceous (5B4), near center dark brown (6F4, 6F6, 7F3), rarely brown (6E4), when dry brown (6E6), light brown (5D5, 5D4), grayish brown (5D3), brownish ochraceous (6C4). ***Stipe*** (22–)25–40(–50) × 1–3(–4) mm; cylindrical, narrowed towards the base, flexuous; surface finely longitudinally striate, first entirely pruinose, then pruinose near lamellae, towards the base glabrous and shiny; color near lamellae brownish ochraceous (5C3, 6C3, 6C4), brownish gray (5D2), grayish brown (5D3, 6D3, 6E3) to brown (6E4), near the base light brown (6D4), grayish brown (6E3), brown (6E4), dark brown (6F3, 6F4, 6F5, 7F4) ***Lamellae*** L = (13–)17–28, l = (0–)1–3(–5); 2.5–4 mm wide; adnexed or adnate-emarginate; color ochraceous-gray (5B2), brownish ochraceous (5C4, 6C4), brownish gray (6C2, 6D2), grayish brown (6D3); edges entire. ***Context*** elastic to fragile; odor indistinct.

***Spores*** (5.2–)6–6.5–7.1(–8) × (3.6–)3.9–4.1–4.4(–5) μm; narrowly ellipsoid to oblong, Q = (1.32–)1.45–1.59–1.72(–2.00); walls amyloid; hilar appendage 0.5–1 μm long. ***Basidia*** (20–)26–29.2–32(–34) × (5.5–)6.5–7–7.5(–9) μm; clavate; with 4 sterigmata. ***Basidioles*** first cylindrical, then clavate, ca. 3–6 μm wide. ***Marginal cells*** (18–)21.5–28.5–35.5(–53) × (3.5–)4–5–6(–8.5) μm; lageniform, with long, narrow, flexuous, nodulose or diverticulate apical projections. ***Pileipellis*** 48–60 μm deep; suprapellis 28–35 μm deep, usually of one or three layers of inflated, densely arranged cells; subpellis well-differentiated, 20–30 μm deep, of densely packed, puzzled, 2.5–10 μm wide hyphae, gradually passing to horizontally oriented hyphae in trama; hyphal terminations with brownish yellow parietal pigments, in subpellis near center also with incrusted brownish yellow pigments, walls thickened up to 0.5 μm, in subpellis up to 1.5 μm. Terminal cells near pileus margin (18–)28.5–37.7–47(–67) × (10–)13.5–18.8–24(–35) μm; usually obpyriform, sphaeropedunculate or clavate; subterminal cells usually unbranched, fusiform or clavate and ventricose, rarely cylindrical, often with lateral swellings or branches. Terminal cells near pileus center (20–)27.5–38.3–49(–72) × (7–)13.5–19.1–24.5(–42) μm; similar to cells near margin; subterminal cells usually narrower, narrowly fusiform. ***Caulocystidia*** (15–)18.5–26.8–35(–48) × 5–11.5–18(–42) μm; very variable in shape and size, some small and clavate, others large and obpyriform, sphaeropedunculate, often flexuous, sometime with projections or lobate, repent and in small or larger clusters, often intermingled, subterminal cells often with lateral projections or branches; usually with slightly thickened walls up to 0.5 μm, but near septa and on subterminal cells up to 1 μm, with brownish yellow parietal pigments. ***Clamp connections*** present.

###### Distribution and ecology.

Known from Austria, Croatia, Germany, The Netherlands, Norway and Slovakia; in semi-natural grasslands on calcareous soil.

###### Additional material studied.

Austria • Burgenland, Oberwart, Rechnitz, Galgenberg, elev. 344 m, coord. 47°17'52"N, 16°25'09"E, semi-dry grassland, soil among moss and grass, 17 Nov 2019, G. Friebes *GF20190130* (SAV F-23423). Croatia • Kamenjak peninsula, 3.5 km S-SE of Premantura, near Pula, coord. 44°46'17"N, 13°55'06"E, grassland with *Rosmarinus* sp., 20 Nov 2005, M. Čerkez (CNF 1/3789). France • Hautes-Pyrénées, Arrens-Marsous, Caytivère, coord. 42°58'51"N, 00°14'53"E, acidophilic mountain grazed grassland (*Nardion*), 4 Oct 2016, C. Hannoire *CH16100418* (BBF, as *D.phaeopodium*); • Hautes-Pyrénées, Campan, vallon du Garet, coord. 42°55'06"N, 00°12'35"E, neutrophilous mountain grassland, 8 Oct 2016, G. Corriol *GC16100816* (BBF, as D.pseudocuneifoliumvar.obscurum); • Puy-de-Dôme, Orcines, Puy de la Charité, coord. 45°46'17"N, 03°01'42"E, lawn on volcanic soil (*Koelerio-Phleion*), 16 Oct 1999, G. Corriol *GC99101603* (BBF, as *D.phaeopodium*). Germany • Altranft, Hütelandschaft, coord. 52°45'19"N, 14°03'42"E, on loamy alkaline soil in extensively grazed (sheep) semi-natural grassland, 6 Nov 2022, A. Karich, R. Ullrich and R. Jarling *IHI-22Der05* (GLM-F137757); • Baden-Württemberg, Justingen, Schachenheide, coord. 48°24'35"N, 09°40'25"E, terrestrial in semi-natural grassland, 2 Oct 2021, S. Adamčík (SAV F-20894); • ibid., 3 Oct 2021, S. Adamčík (SAV F-20914). The Netherlands • Beilen, nature area Schepping, in poor grassland on calcareous loam, 1 Oct 2004, E. Arnolds *Arnolds 04-110* (L, as *D.rancidum* n. prov.). Norway • Akershus, Jevnaker, Rustad, semi-natural, calcareous grassland, 16 Aug 2023, T. E. Brandrud *TEB332-23* (O). Slovakia • Kremnické vrchy Mts., pasture 0.5 km W of Tajov, elev. 600 m, coord. 48°44'54"N, 19°03'31"E, terrestrial, 24 Oct 2020, S. Adamčík (SAV F-20815); • ibid., 25 Oct 2020, M. Caboň (SAV F-20845); • Poloniny Mts., 4 km N of Stakčín, pastures above the water reservoir Stariná, elev. 380–420 m, coord. 49°02'431"N, 22°14'56"E, terrestrial, 25 Sep 2017, S. Adamčík (SAV F-20230).

###### Notes.

*Dermolomapruinosipes* is a member of D.subgenusAmylospora, section Atrobrunnea. It belongs to an inclusive clade of species with mainly collyboid basidiomata, all of which are morphologically similar. For more comments and recommendations, see the notes under the previous species. Compared to other members of the clade it is relatively distinct, as the stipes are typically distinctly pruinose along their entire length and the marginal cells have long attenuated apical projections. The species cluster of *D.pruinosipes* includes 14 samples from seven countries, but it was represented only by a single sample in the previous phylogeny by Sánchez-García et al. (2021) and labelled as “*Dermoloma* sp. 8”. It is represented here by three distinct sequence variants in the ITS tree (Suppl. material 8), but the multilocus phylogeny (Fig. 2) did not support to distinguish them as three separate taxa and we did not find distinct morphological differences to distinguish them.

##### ﻿ Dermoloma aff. pruinosipes

Taxon classificationAnimaliaAgaricalesTricholomataceae

CD3BD0D4-A124-598D-8610-18A81656E846

Figs 43b , 45

###### Description.

***Pileus*** 5–9 mm; convex to plano-convex, rarely broadly umbonate; margin when fresh striate to 1/3 to 1/2 of the cap (not distinctly); surface smooth, almost velvety, hygrophanous; color dark brown in center (7F6), towards margin usually lighter, yellowish brown (5E6). ***Stipe*** 15–40 × 1–2 mm; cylindrical, flexuous; surface smooth or usually white pruinose, especially at apex, sometimes finely longitudinally striate; color at apex usually grayish brown (7E3), often concolorous throughout but sometimes dark brown towards the base (7F5), with white basal mycelium. ***Lamellae*** L = 11–18, l = 1–3; 1–3 mm wide; adnexed to adnate-emarginate; color similar when young and mature, olive brown (4E3); edges entire. ***Context*** elastic to fragile; odor slightly farinaceous.

***Spores*** (5.1–)5.7–6.1–6.4(–6.7) × (3.5–)3.7–3.9–4(–4.3) μm; narrowly ellipsoid or oblong, Q = (1.38–)1.48–1.57–1.65(–1.72); walls amyloid; hilar appendage 0.5–1 μm long. ***Basidia*** (19–)22–24.7–28(–30) × (5.5–)6–6.2–7 μm; clavate; with 4 sterigmata. ***Basidioles*** first cylindrical, then clavate, ca. 3.5–8 μm wide. ***Marginal cells*** (9–)12–16.7–21.5(–27) × (3.5–)4–4.9–6(–6.5) μm; not well-differentiated and similar to basidioles, cylindrical or clavate, occasionally also fusiform or lageniform. ***Pileipellis*** 45–50 μm deep; suprapellis 27–33 μm deep, usually of one layer of inflated and relatively loose cells; subpellis hardly differentiated, ca. 12–23 μm deep, of densely packed, mainly horizontally oriented, 3–10(–15) μm wide hyphae, gradually passing to horizontally oriented and intricate hyphae in trama; hyphal terminations with brownish yellow parietal pigments, in subpellis darker yellow-brown and sometimes slightly incrusted pigments, walls of terminal cells thickened up to 0.5 μm, in subpellis up to 1 μm. Terminal cells near pileus margin (22–)26.5–34.4–42.5(–50) × (11–)14–17.7–21.5(–25) μm; usually obpyriform, clavate, occasionally sphaeropedunculate, occasionally lobate near septa; subterminal cells mainly branched, fusiform or cylindrical, usually not inflated, occasionally lobate. Terminal cells near pileus center (24–)31.5–38.3–45(–52) × (11–)14–17.1–20.5(–28) μm; clavate, obpyriform or sphaeropedunculate; subterminal cells similar to cells near margin. ***Caulocystidia*** (26–)33.5–53.5–61(–85) × (4–)4.5–6.6–8.5(–12) μm; clavate or subcylindrical, occasionally fusiform, usually flexuous or twisted, sometimes slightly moniliform, occasionally nodulose or lobate, repent or ascending, individual or in small to larger clusters, often intermingled; thin-walled, near septa often slightly thickened up to 0.5 μm, with brownish yellow parietal pigments. ***Clamp connections*** present.

**Figure 45.**
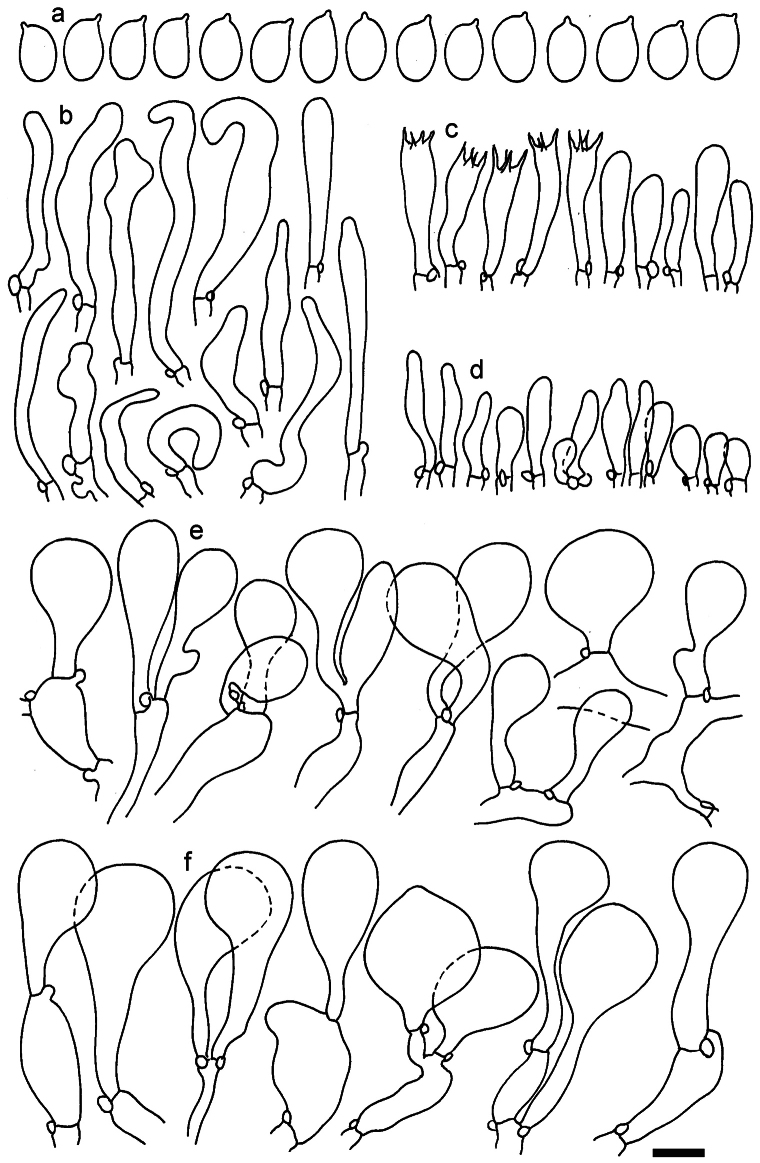
Dermolomaaff.pruinosipes (SAV F-23429), microscopic elements. **a** Spores; **b** caulocystidia; **c** basidia and basidioles; **d** pileipellis elements near the pileus margin; **e** pileipellis elements near the pileus center. Scale bar: 5 µm for spores and 10 µm for other elements.

###### Distribution and ecology.

Known from a single locality in Austria.

###### Material studied.

Austria • Steiermark, Leibnitz, Heimschuh, Fastlkogel, Plesch, elev. 396 m, coord. 46°47'07"N, 15°28'51"E, semi-dry grassland, soil among mosses and grass, 15 Nov 2018, G. Friebes *GF20180313* (SAV F-23429).

###### Notes.

This collection is not described formally as a new species because it is represented by a single sample. It probably represents a well-defined species with amyloid spores belonging to D.subgenusAmylospora, section Atrobrunnea. It is well-supported by the multilocus phylogeny (Fig. 2) as closely related to *D.pruinosipes* and *D.curvicystidiatum*. It differs from both of these by the smaller basidiomata and poorly differentiated marginal cells (see above). Its delimitation from other species with small basidiomata requires more observations to understand the variability of its morphological characters.

##### ﻿ Dermoloma pseudojosserandii

Taxon classificationAnimaliaAgaricalesTricholomataceae

Corriol & Hannoire sp. nov.

39EA9D55-5936-5AEA-A94F-29FC8E57E3AD

856443

Figs 43c, d , 46

###### Etymology.

Species closely related and similar to *D. josserandii*.

###### Holotype.

France • Hautes-Pyrénées, Lau-Balagnas, Barderou, grazed grassland, 5 Oct 2017, C. Hannoire *CH17100504* (BBF, as *D.phaeopodium*).

###### Diagnosis.

European species; basidiomata moderately large, collybioid, pale colored; from similar *D.josserandii* different in usually more than 30 lamellae near the stipe attachment; stipes with evenly pale colors along entire length; spores < 6.5 µm long; marginal cells > 8 µm wide.

***Pileus*** 15–35 mm; convex, soon plane, sometimes lobate; margin indistinctly translucently striate when wet; surface rough, rugulose or pitted, smooth near margin, indistinctly hygrophanous, fading from the center; color when mature near margin grayish brown (5D3, 6D3) to brown (6E4, 6E5), when dry grayish brown (6C3) or ochraceous-gray (5B2), near center light brown (6D4), grayish brown (6D3) to brown (6E4), when dry grayish ochraceous (5B3). ***Stipe*** 16–50 × 2.5–5.5 mm; cylindrical, sometimes flexuous, grooved, narrowed towards the base; surface pruinose near lamellae, towards the base longitudinally fibrillose-striate; color yellowish gray (3B2) to ochraceous-gray (6B2), slightly darker near the base. ***Lamellae*** L = 30–37, l = 1–7; 3–7 mm wide; adnate-emarginate and decurrent with tooth; color ochraceous-gray (5B2); edges entire or slightly irregular, paler. ***Context*** in pileus up to 2.5 mm wide; elastic, later fragile; odor farinaceous.

**Figure 46.**
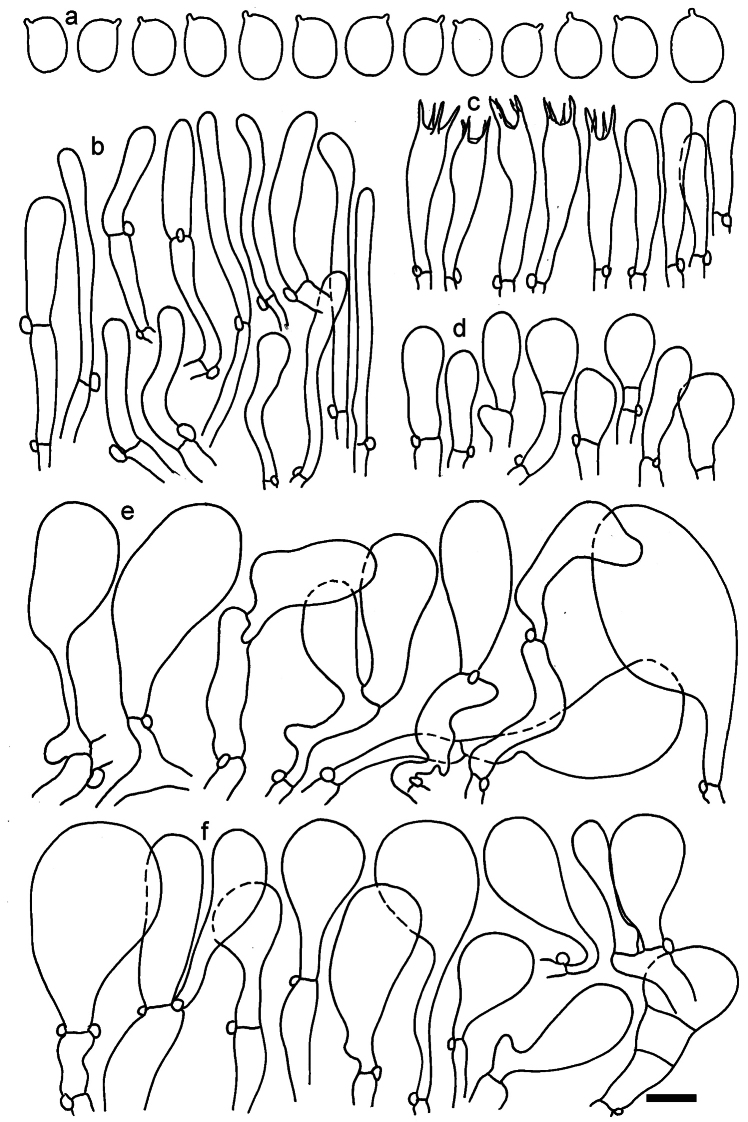
*Dermolomapseudojosserandii* (SAV F-20908), microscopic elements. **a** Spores; **b** caulocystidia; **c** basidia and basidioles; **d** marginal cells; **e** pileipellis elements near the pileus margin; **f** pileipellis elements near the pileus center. Scale bar: 5 µm for spores and 10 µm for other elements.

***Spores*** (5.1–)5.8–6.2–6.6(–7.2) × (3.5–)4.1–4.5–4.8(–5.2) μm; ellipsoid, Q = (1.18–)1.3–1.39–1.47(–1.55); walls amyloid; hilar appendage 0.7–1.5 μm long. ***Basidia*** (24–)26–29.3–32.5(–36) × (6–)6.5–7.1–7.5(–9) μm; clavate; with 4 sterigmata. ***Basidioles*** first cylindrical, then clavate, ca. 3–7 μm wide. ***Marginal cells*** (12–)15.5–20.6–26(–35) × (5–)6.5–9.1–11.5(–13) μm; clavate, obpyriform or ellipsoid, occasionally flexuous, rarely nodulose or lobate, apically obtuse. ***Pileipellis*** 48–60 μm deep; suprapellis of one or two (rarely three) layers of inflated, densely arranged cells; subpellis not well-differentiated, hardly 10 μm deep, of densely packed, almost horizontally oriented, 3–10 μm wide hyphae, gradually passing to trama; hyphal terminations with brownish yellow parietal pigments, walls thickened up to 0.5 μm, near septa of terminal cells and in subpellis up to 1 μm. Terminal cells near pileus margin (23–)29.5–40.1–51(–76) × (12–)15.5–19.9–24.5(–33) μm; usually clavate or sphaeropedunculate, rarely obpyriform or ellipsoid, frequently with narrowed, flexuous basal part; subterminal cells frequently branched, usually narrower, irregularly flexuous and lobate, rarely inflated-ventricose. Terminal cells near pileus center (18–)28–38.4–49(–66) × (7–)14.5–20.5–26.5(–45) μm; clavate or obpyriform, occasionally ellipsoid; subterminal cells usually unbranched, cylindrical, occasionally fusiform-ventricose, occasionally nodulose or with lateral swellings. ***Caulocystidia*** (13–)21–33.5–46(–63) × 4–5.6–7(–9) μm; mainly narrowly clavate, rarely cylindrical, sometimes fasciculated, erect or ascending, near lamellae forming continuous trichoderm structure; thin-walled or slightly thickened up to 0.5 μm near septa, with brownish yellow parietal pigments. ***Clamp connections*** present.

###### Distribution and ecology.

Known from three localities in France and Germany; in semi-natural grasslands or deciduous forests, perhaps on calcareous soil, but insufficiently known.

###### Additional material studied.

France • Hautes-Pyrénées, Ossen, coord. 43°04'05"N, 00°03'36"E, broadleaf eutrophic forest, 23 Sep 2017, G. Corriol *GC17092306* (BBF, as *D.nitens*). Germany • Baden-Württemberg, Justingen, Schachenheide, coord. 48°24'35"N, 09°40'25"E, terrestrial among grass, 3 Oct 2021, M. Caboň (SAV F-20908); • ibid., 3 Oct 2021, S. Adamčík (SAV F-20912).

###### Notes.

*Dermolomapseudojosserandii* is a member of D.subgenusAmylospora, section Atrobrunnea. It is closely related to *D.josserandii*. For details about its morphological delimitation see the notes referring to the latter species.

##### ﻿ Dermoloma pusillum

Taxon classificationAnimaliaAgaricalesTricholomataceae

Contu, in Contu, Consiglio & Setti, Micol. Veg. Medit. 22(2): 105. 2008.

14920CBD-2D93-5A5D-997F-4E7460D63247

533165

Fig. 47

###### Holotype.

Italy • Sardinia, near Olbia, Pittulongu, 19 Dec 2006, F. Bocianolo *30-XII-2006* (AQUI).

###### Distinguishing characters.

European species; basidiomata small, mycenoid; spores amyloid, broadly ellipsoid to narrowly ellipsoid, up to 6.5 μm long; caulocystidia large, 25.5–46 × 9.5–15.5 μm, clavate, sphaeropedunculate or obpyriform, known only from Mediterranean areas of Europe.

**Basidiomata** not collected by authors of this study, for description see original description (Contu et al. 2008).

***Spores*** (5.4–)5.7–6–6.4(–6.7) × (3.7–)4.3–4.6–4.8(–5.3) μm; broadly ellipsoid to narrowly ellipsoid, Q = (1.19–)1.25–1.32–1.39(–1.51); walls amyloid; hilar appendage ca. 1–1.5 μm long. ***Basidia*** (25–)26.5–28.5–30.5(–34) × (5–)5.5–6.2–7(–8) μm; clavate, cylindrical or fusiform; with 4 sterigmata. ***Basidioles*** first cylindrical, then clavate, ca. 3.5–6 μm wide. ***Marginal cells*** (10–)15–18.6–22(–25) × (2–)4–6.1–8.5(–11) μm; clavate or cylindrical, flexuous, often lobate, apically mainly obtuse but often also constricted. ***Pileipellis*** 40–58 μm deep; suprapellis 30–40 μm deep, mainly of one layer of inflated, densely arranged cells; subpellis not well-differentiated, 16–20 μm deep, of densely packed, mainly horizontally oriented, intricate, branched, 2–5(–10) μm wide hyphae gradually passing to horizontally oriented hyphae in trama; hyphal terminations with brownish yellow parietal pigments, usually thin-walled or with slightly thickened walls up to 0.7 μm. Terminal cells near pileus margin (20–)29.5–38.1–46.5(–58) × (9–)15–19.5–24(–30) μm; mainly sphaeropedunculate, occasionally obpyriform or clavate, near septa flexuous, and sometimes nodulose; subterminal cells usually narrower and subcylindrical, occasionally clavate or fusiform, often branched or nodulose, flexuous. Terminal cells near pileus center (24–)33–43.3–54(–71) × (10–)15.5–21–26.5(–30) μm; mainly clavate, often also sphaeropedunculate, more distinctly clavate and nodulose towards septa; subterminal cells similar to cells near margin. ***Caulocystidia*** (24–)25.5–35.8–46(–78) × (7–)9.5–12.5–15.5(–20) μm; clavate, sometimes sphaeropedunculate or obpyriform, apically obtuse, repent with ascending tips or erect, often irregularly oriented, individual or in small to large fascicules; thin-walled, with brownish yellow parietal pigments. ***Clamp connections*** present.

**Figure 47.**
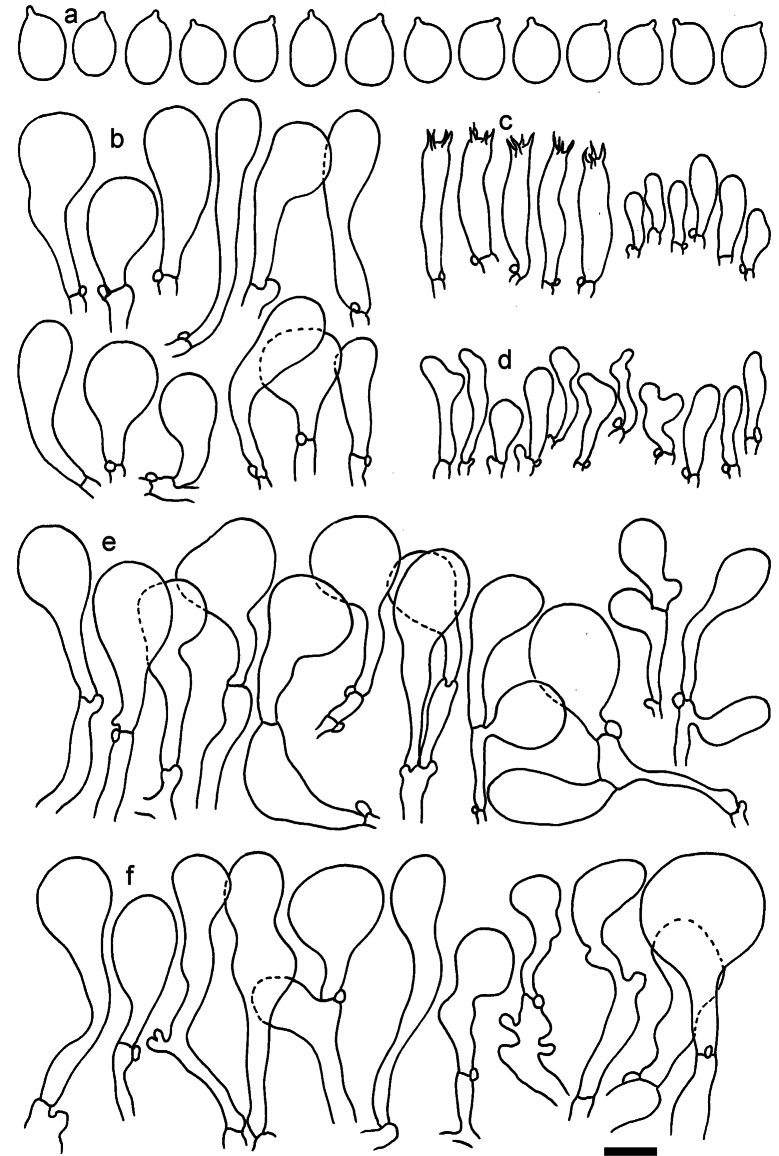
*Dermolomapusillum* (AQUI 30.XII.2006, holotype), microscopic elements. **a** Spores; **b** caulocystidia; **c** basidia and basidioles; **d** marginal cells; **e** pileipellis elements near the pileus margin; **f** pileipellis elements near the pileus center. Scale bar: 5 µm for spores and 10 µm for other elements.

###### Distribution and ecology.

Known only from Sardinia (Italy); possibly an exclusively Mediterranean species.

###### Additional material studied.

Italy • Sardinia, near Olbia, Pittulongu, 11 Jan 2008, M. Contu *11.I.2008* (AQUI).

###### Notes.

Two collections representing the type of *D.pusillum* and other authentic material were included in the study by Sánchez-García et al. (2021). The species was not collected by the authors of this study. It has amyloid spores and belongs to D.subgenusAmylospora, section Atrobrunnea. It forms a well-supported clade with *D.obscurum*, *D.clavicystis* and an unclarified *Dermoloma* species (Fig. 2; for morphological differences see notes under *D.obscurum*). The most similar species is probably *D.vestigium*, due to its very small basidiomata and similar spores. They differ by narrower, attenuated and flexuous marginal cells and shorter caulocystidia in the latter species.

##### ﻿ Dermoloma rostratum

Taxon classificationAnimaliaAgaricalesTricholomataceae

Mešić, Tkalčec, Brandrud & Dima sp. nov.

26C55321-708C-5B20-842D-D194A73BACA2

856444

**Figure 48.**
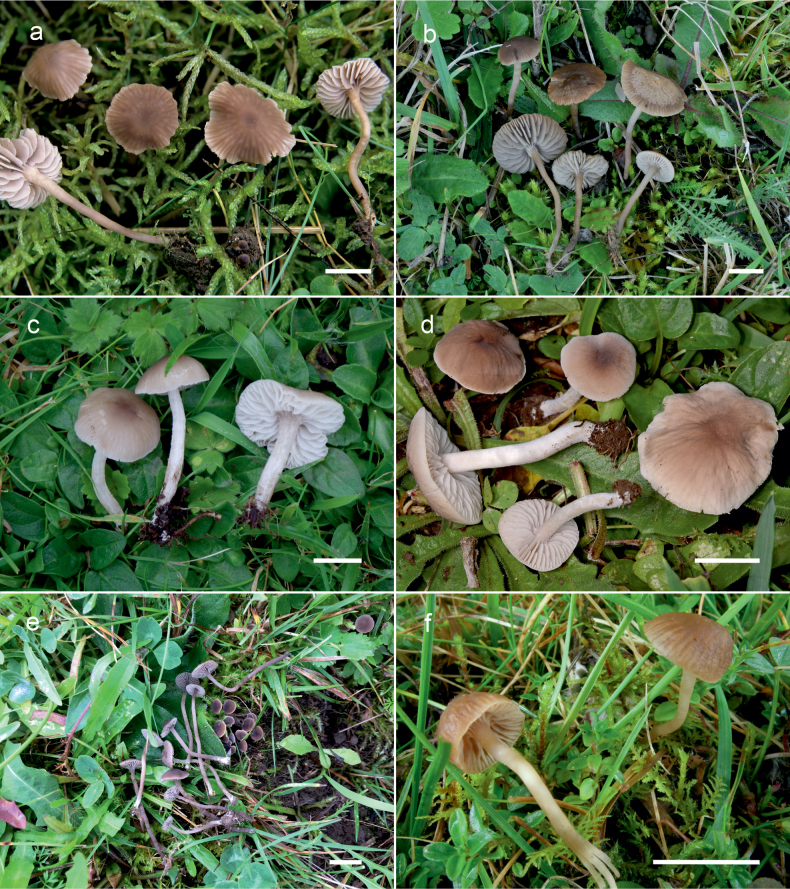
Basidiomata of *Dermoloma* in field appearance. **a***Dermolomarostratum* (CNF 5/209), photo A. Mešić; **b***Dermolomarostratum* (GLM-F137760), photo A. Karich; **c***Dermolomasimile* (SAV F-4421, holotype), photo D. Harries; **d***Dermolomasimile* (CNF 1/5620), photo A. Mešić; **e***Dermolomavestigium* (SAV F-20662, holotype), photo S. Jančovičová; **f***Dermolomavestigium* [BBF (*GC14101103*)], photo G. Corriol. Scale bar: 10 mm.

**Figure 49.**
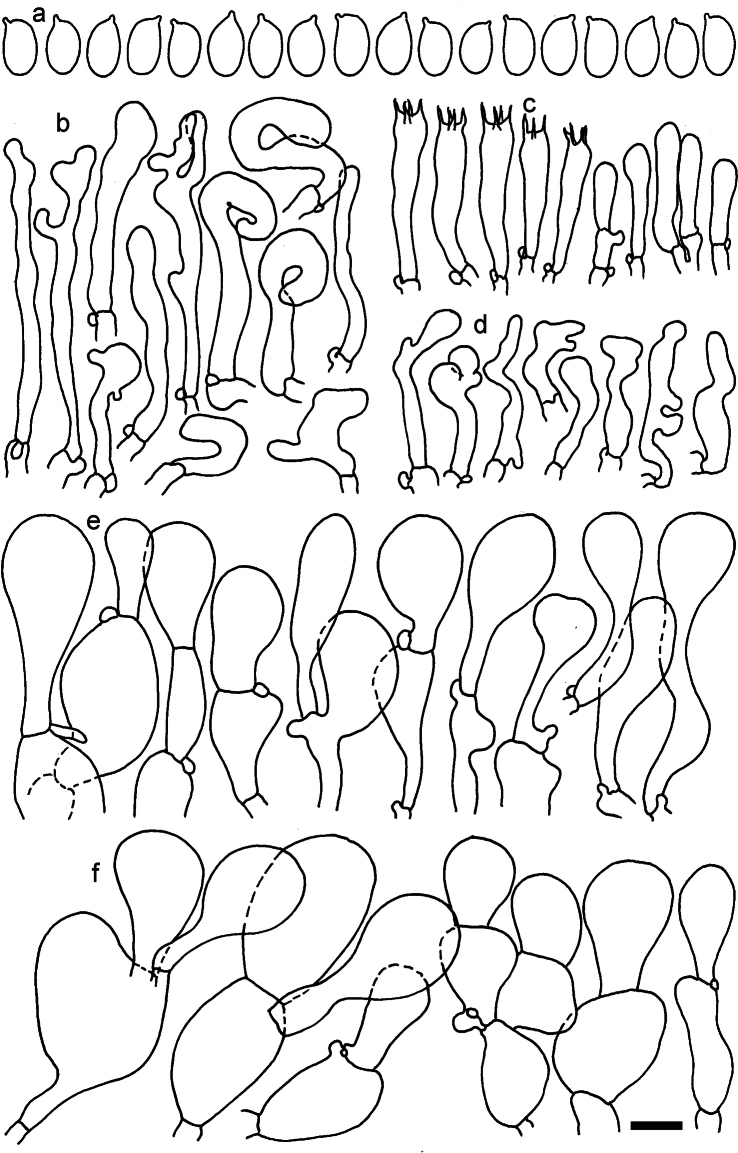
*Dermolomarostratum* (SAV F-20811, holotype), microscopic elements. **a** Spores; **b** caulocystidia; **c** basidia and basidioles; **d** marginal cells; **e** pileipellis elements near the pileus margin; **f** pileipellis elements near the pileus center. Scale bar: 5 µm for spores and 10 µm for other elements.

Figs 43e, f , 48a, b , 49

###### Etymology.

Caulocystidia and marginal cells have curved beak-like terminations.

###### Holotype.

Slovakia • Kremnické vrchy Mts., pasture 0.5 km W of Tajov, elev. 600 m, coord. 48°44'54"N, 19°03'31"E, terrestrial, 24 Oct 2020, M. Caboň (SAV F-20811).

###### Diagnosis.

European species; basidiomata small, mycenoid; pilei 4–20 mm in diameter with distinctly translucently striate margin; stipes 0.5–2 mm wide; lamellae brownish gray or grayish brown; spores amyloid, 5–6 × 3.2–3.7 μm, narrowly ellipsoid to oblong; caulocystidia 4.5–8 μm wide, usually flexuous, often lobate, nodulose or diverticulate.

***Pileus*** 4–20(–22) mm; conico-convex, later almost plane, sometimes indistinctly umbonate; margin translucently striate to half of the radius, sometimes indistinctly crenulate; surface smooth, near center sometimes rough or rugolose, hygrophanous; color near margin grayish brown (5D3, 5E3) to brown (6E4), near center dark brown (6F3, 7F4, 7F5) to almost black, when dry margin pale grayish brown, center ochre-brown. ***Stipe*** (8–)16–37(–48) × 0.5–2(–3) mm; cylindrical, flexuous, slightly tapering towards the base; surface when young finely pruinose all over, later almost glabrous; color near lamellae light brown (7D3), grayish brown (5E3) to brown (5E4), near the base dark brown (6F4, 7F4). ***Lamellae*** L = 16–28(–38), l = 1–3; 1–3 mm wide; adnexed to adnate-emarginate, sometimes decurrent with tooth; color brownish gray (7C2) or grayish brown (6D3); edges entire, concolorous. ***Context*** fragile; odor farinaceous or indistinct.

***Spores*** (4.3–)5–5.5–6(–6.7) × (2.9–)3.2–3.5–3.7(–4.3) μm from collections with 4-spored basidia and (5.4–)5.7–6.7–7.5(–8.1) × (3.4–)3.5–3.9–4.2(–4.5) μm from collections with 2-spored basidia; narrowly ellipsoid to oblong, Q = (1.30–)1.45–1.59–1.73(–1.91) from collections with 4-spored basidia and (1.40–)1.47–1.72–1.94(–2.03) from collections with 2-spored basidia; walls amyloid, sometimes thick-walled and dextrinoid; hilar appendage ca. 0.5–1.5 μm long. ***Basidia*** (20–)23–26.3–29.5(–34) × (5–)6–6.1–7(–7.5) μm; clavate; usually with only 4 sterigmata, some collections with predominantly or exclusively 2 sterigmata. ***Basidioles*** first cylindrical, then clavate, ca. 3.5–6.5 μm wide. ***Marginal cells*** (16–)20.5–27.1–34(–40) × (4.5–)5–6–7(–7.5) μm; irregularly shaped, cylindrical, clavate, lageniform, fusiform, lobate, flexuous, nodulose. ***Pileipellis*** 60–80 μm deep; suprapellis 40–55 μm deep, usually of one to three layers of inflated, densely arranged cells; subpellis not well differentiated, ca. 15–35 μm deep, of densely packed, puzzled, almost horizontally oriented, 3.5–10(–15) μm wide hyphae, not sharply delimited from horizontally oriented hyphae in trama; hyphal terminations with brownish yellow parietal pigments, walls thickened up to 0.5 μm, near septa of terminal cells and in subpellis up to 1 μm. Terminal cells near pileus margin (21–)29.5–39–48.5(–70) × (10–)13–16.8–20.5(–32) μm; obpyriform or clavate, often flexuous towards septa; subterminal cells narrower or equally wide, often branched, ventricose, often lobate. Terminal cells near pileus center (20–)28.5–40.7–53(–87) × (8–)13–18.5–24(–38) μm; pyriform or clavate, rarely sphaeropedunculate; subterminal cells cylindrical to broadly ventricose, often with lateral swellings or irregularly lobate. ***Caulocystidia*** (11–)18.5–40.9–63.5(–93) × (4–)4.5–6–8(–14) μm; clavate, rarely cylindrical, fusiform or obpyriform, usually flexuous, often lobate, nodulose or diverticulate, sometimes moniliform, in small or large clusters, loosely arranged, irregularly oriented, interwoven; usually with slightly thickened walls up to 0.5 μm, with brownish yellow parietal and occasionally slightly incrusted pigments. ***Clamp connections*** present.

###### Distribution and ecology.

Widely distributed, from the Mediterranean to boreal regions. Known from Austria, Croatia, Denmark, Germany, The Netherlands, Norway and Slovakia; in semi-natural grasslands, natural, dry grasslands or forests, usually on calcareous soil.

###### Additional material studied.

Austria • Steiermark, Graz-Umgebung, Frohnleiten, Pöllagraben, elev. 588 m, coord. 47°17'08"N, 15°14'55"E, soil among mosses and grass, 26 Sep 2020, G. Friebes *GF20200166* (SAV F-23430). Croatia • Mljet National Park, Mljet Island, 1 km W/W-SW of Goveđari village, coord. 42°46'52"N, 17°21'03"E, forest of *Ericaarborea*, *Phillyrealatifolia*, *Pinushalepensis*, *Pistacialentiscus* and *Quercusilex*, 12 Nov 2010, A. Mešić (CNF 1/6117); • Mljet Island, 250 m NW/W-NW of Prožurska luka village, coord. 42°43'51"N, 17°38'442"E, hiking trail along maquis of *Arbutusunedo*, *Myrtuscommunis*, *Pinushalepensis* and *Pistacialentiscus*, among mosses, 9 Nov 2009, Z. Tkalčec (CNF 1/5694); • Zagreb, Črnomerec, coord. 45°49'57"N, 15°56'58"E, mowed grassland with short grass and mosses, 7 Oct 1998, A. Mešić (CNF 5/209). Denmark • Møn, Kongsbjerg, in natural, calcareous grassland, 17 Oct 2015, T. Læssøe and T. Smidth, *DMS-725350* (C). Germany • Altranft, Hütelandschaft, coord. 52°45'19"N, 14°03'42"E, in slightly calcareous extensively grazed (sheep) grassland, 6 Nov 2022, A. Karich, R. Ullrich and R. Jarling *IHI-22Der06* (GLM-F137760); • Hainewalde, Gampenstein, coord. 50°55'14"N, 14°43'22"E, loamy meadow, 23 Sep 2021, A. Karich and R. Ullrich *IHI-21Der02* (GLM-F137759, as *D.josserandii*). The Netherlands • Beilen, nature area Schepping, in poor grassland on calcareous loam, 24 Sep 2013, E. Arnolds *Arnolds 13-6* (L, as D.cf.phaeopodium). Norway • Akershus, Asker, Elnestangen SV, 59°48'03"N, 10°29'53"E, in calcareous meadow/lawn, 18 Sept 2015, T. E. Brandrud, B. Dima *DB5815* (ELTE), duplicate *TEB417-15* (O); • Oslo, Oslo, Nakholmen, coord. 59°53'22"N, 10°41'40"E, in semi-natural, dry grassland close to sea, 6 Oct 2013, A. Molia and T. Læssøe *NOBAS2933-16* (O-F-21833); • Sør-Trøndelag, Frøya, Uttian, Nordstaulvika, coord. 19°51'47"N, 07°05'12"E, calcareous sheep pasture, semi-natural meadow, 16 Sept 2021, G. Gaarder and S. M. G. Nyjordet *GG7975* (O); • Telemark, Bamble, Eikstrand, coord. 59°01'51"N, 09°43'26"E, in semi-natural, dry grassland close to sea, 30 Aug 2012, A. Molia and T. Læssøe *NOBAS4369-17* (O-F-245572). Slovakia • Kremnické vrchy Mts., pasture 0.5 km W of Tajov, elev. 600 m, coord. 48°44'54"N, 19°03'30.6"E, terrestrial, 24 Oct 2020, S. Adamčík (SAV F-20817).

###### Notes.

*Dermolomarostratum* has amyloid spores and belongs to D.subgenusAmylospora, section Atrobrunnea. The species is included in a phylogeny for the first time in this study, but it seems that it is rather widespread and probably overlooked due to the small size and basidiomata resembling species of the genus *Psathyrella*.

##### ﻿ Dermoloma simile

Taxon classificationAnimaliaAgaricalesTricholomataceae

Adamčík & Jančovič. sp. nov.

83E37245-DCC6-530F-BABC-B220C11A35D2

856445

Figs 48c, d , 50

###### Etymology.

Similar to the generic type, *Dermoloma cuneifolium*.

###### Holotype.

United Kingdom • Wales, Pembrokeshire, Upton Castle, coord. 51°42'22"N, 04°51'57"E, terrestrial in lawn, 26 Oct 2014, D. Harries (SAV F-4421).

###### Diagnosis.

European species; basidiomata small to medium-sized; pilei 8–22 mm in diameter, with margins indistinctly striate when wet; stipes and lamellae predominantly grayish-ochraceous or grayish brown; spores inamyloid, on average up to 3.6 µm wide and/or Q > 1.4; caulocystidia 3–6 µm wide.

***Pileus*** (6–)8–22(–28) mm; convex, expanding to plane, sometimes indistinctly umbonate, sometimes lobate; margin not striate, indistinctly translucently striate to half of radius when wet; surface smooth near margin, rough, radially rugulose, sometimes wrinkled or veined near center, weakly hygrophanous; color when young near margin grayish brown (6E3, 7E3) to brown (7E4), near center dark brown (7F4) to black, when mature near margin brown (5E4, 6E4, 6E5), grayish brown (5D3, 6E3), light brown (6D5), brownish gray (6C2), when dry grayish brown (5D3, 6D3), brownish ochraceous (5C3, 6C3), ochraceous-gray (5B2, 6B2), near center dark brown (6F3, 6F4, 6F5) to almost black, brown (6E46E5), grayish brown (6D3, 6E3), when dry sometimes paler brown (5E4) to brownish ochraceous (6C3). ***Stipe*** (15–)21–39(–52) × 1–4 mm; cylindrical, narrowed towards the base, flexuous; surface finely longitudinally striate, when young pruinose along all length, when mature towards the base finely fibrillose or silky and shiny; color grayish ochraceous (5B3), ochraceous-gray (5B2) to almost white, near the base concolorous or darker, especially with age, brownish ochraceous (5C3, 6C3, 7C3), grayish brown (5D3, 6D3) or brownish gray (6C2). ***Lamellae*** L = (20–)23–38, l = 0–3; 2–5.5 mm wide; adnate-emarginate and decurrent with tooth; color ochraceous-gray (5B2, 6B2) to almost white, brownish gray (5C2, 6C2, 7C2), grayish brown (5D3), grayish ochraceous (5B3); edges entire. ***Context*** fragile; odor farinaceous.

**Figure 50.**
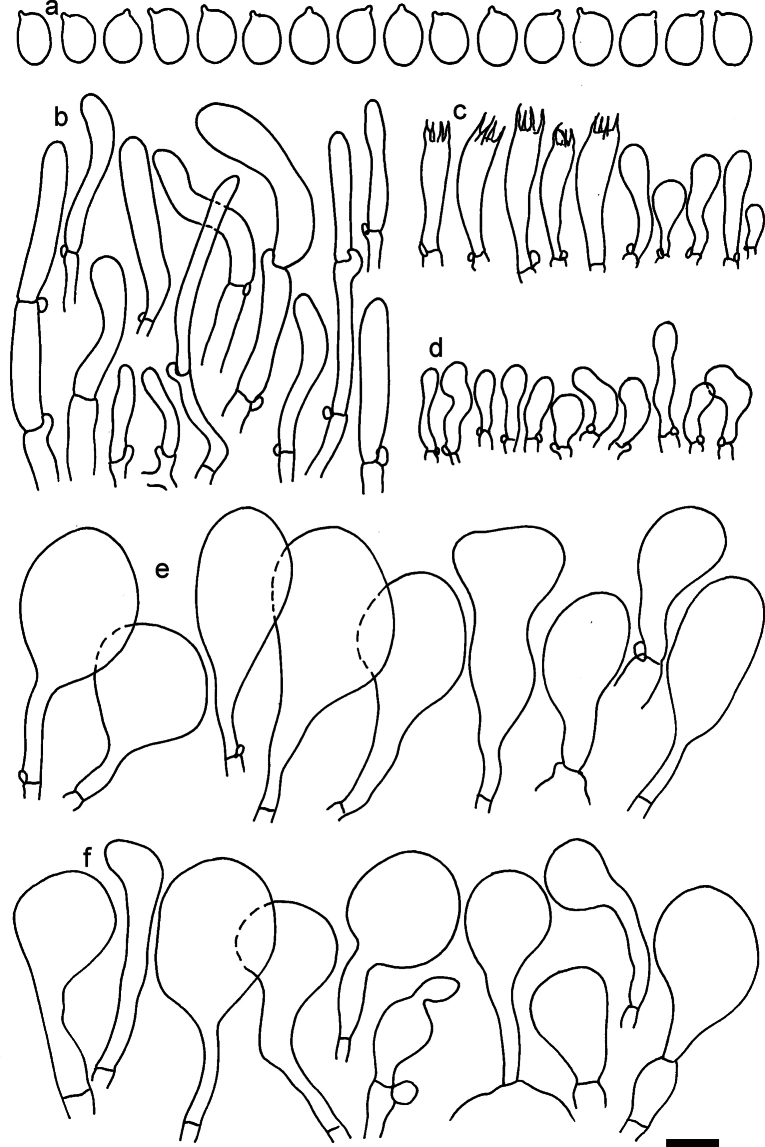
*Dermolomasimile* (SAV F-4359), microscopic elements. **a** Spores; **b** caulocystidia; **c** basidia and basidioles; **d** marginal cells; **e** pileipellis elements near the pileus margin; **f** pileipellis elements near the pileus center. Scale bar: 5 µm for spores and 10 µm for other elements.

***Spores*** (3.9–)4.7–5.1–5.4(–6.1) × (2.9–)3.3–3.5–3.8(–4.6) μm; ellipsoid to narrowly ellipsoid, Q = (1.15–)1.31–1.43–1.55(–1.91); walls inamyloid, sometimes thick-walled and dextrinoid; hilar appendage ca. 0.5–1.5 μm long. ***Basidia*** (17–)22–25.4–28.6(–34) × (4.5–)5.5–6.1–7(–8.5) μm; clavate; with 4 sterigmata. ***Basidioles*** first cylindrical, then clavate, ca. 3–6.5 μm wide. ***Marginal cells*** (10–)11–14.5–18(–24.5) × (3.5–)4–4.9–6(–6.5) μm; not well-differentiated, clavate, rarely cylindrical or ellipsoid, often flexuous. ***Pileipellis*** 65–105 μm deep; suprapellis 45–55 μm deep, usually of two or three layers of inflated, densely arranged cells; subpellis well-differentiated, 20–50 μm deep, of densely packed, irregularly oriented, intricate, 3–10(–18) μm wide hyphae, gradually passing to horizontally oriented hyphae in trama; hyphal terminations with brownish parietal pigments, in subpellis locally also with dark brown incrusted pigments, walls thickened up to 1 μm especially in subpellis. Terminal cells near pileus margin (22.5–)31.5–41.3–51(–75) × (7–)14.5–19.7–25(–36) μm; sphaeropedunculate, obpyriform or clavate; subterminal cells clavate or fusiform and usually equally wide, sometimes also narrower and almost cylindrical, usually unbranched. Terminal cells near pileus center (18.5–)31–40.8–51(–70) × (7–)12–18.6–25(–36) μm; similar to cells near margin; subterminal cells narrower and subcylindrical, more frequently with dark brown incrustations. ***Caulocystidia*** (16.5–)23–32.6–41(–59.5) × (2.5–)3–4.6–6(–10) μm; clavate or cylindrical, usually not or only slightly flexuous, often clustered in repent or ascending fascicules, sometimes individual and repent; usually slightly thickened up to 0.5 μm, often with crystalline or granulose yellow incrustations. ***Clamp connections*** present.

###### Distribution and ecology.

Widely distributed, mainly in temperate regions, but also found in boreal-montane sites. Known from Austria, Croatia, Finland, Germany, Slovakia and United Kingdom; in semi-natural grasslands, once in a woodland meadow, and once in a rich deciduous forest; probably preferring calcareous soil.

###### Additional material studied.

Austria • Steiermark, Graz-Umgebung, Frohnleiten, Rabenstein, elev. 466 m, coord. 47°14'59"N, 15°18'25"E, on soil among mosses and grass, 2 Oct 2021, G. Friebes *GF20210511* (SAV F-23431). Croatia • podr. Hunjka/Rauchova lugarnica, ca. 400 m NW/N-NW, pl. Medvednica, coord. 45°55'03"N, 15°58'13"E, pasture near forest of *Fagussylvatica*, *Pinussylvestris*, *Betulapendula* and *Populustremula*, 27 Sep 2009, A. Mešić (CNF 1/5620). Finland • Etelä-Häme, Nokia, Sarpatti, Maatialanharju, coord. 61°29'02"N, 23°33'18"E, on dry meadow under *Pinus* sp., *Betula* sp., *Sorbus* sp., 29 Sep 2004, U. Salo and P. Salo *FISAP656-13* (H6016609). Germany • Baden-Württemberg, Justingen, Schachenheide, coord. 48°24'35"N, 09°40'25"E, terrestrial in semi-natural grassland, 2 Oct 2021, M. Caboň (SAV F-20858); • ibid., 3 Oct 2021, S. Adamčík (SAV F-20906). Slovakia • Javorie Mts., Slatinské Lazy, Jombíkovci, meadow near Matúšov hájik cottage, elev. 460 m, coord. 48°29'47"N, 19°19'08"E, terrestrial, 22 Oct 2020, M. Caboň (SAV F-20758); • ibid., 22 Oct 2020, leg. M. Caboň (SAV F-20759); • Kremnické vrchy Mts., pasture 0.5 km W of Tajov, elev. 600 m, coord. 48°44'54"N, 19°03'31"E, terrestrial, 25 Oct 2020, M. Caboň (SAV F-20836); • Laborecká vrchovina Mts., 1.5 km NNE of Svetlice, elev. 390 m, coord. 49°11'03"N, 22°02'38"E, terrestrial on pasture, 21 Sep 2006, S. Adamčík (SAV F-4140); • ibid., 23 Oct 2007, S. Adamčík (SAV F-4141); • ibid., 23 Oct 2007, S. Adamčík (SAV F-4152); • Poloniny Mts., 4 km N of Stakčín, pastures above the water reservoir Starina, elev. 380–420 m, coord. 49°02'43"N, 22°14'56"E, terrestrial, 25 Sep 2017, M. Caboň (SAV F-20228); • Zvolenská kotlina Basin, pasture E of Bečov, elev. 400–450 m, coord. 48°38'48"N, 19°14'49"E, terrestrial, 17 Sep 2020, S. Adamčík (SAV F-20661). United Kingdom • England, Shropshire, Shrewsbury – cemetery, coord. 52°41'46"N, 02°45'32"E, terrestrial in lawn, 21 Oct 2014, S. Adamčík (SAV F-4359); • Wales, Pembrokeshire, Upton Castle, coord. 51°42'22"N, 04°51'57"E, terrestrial in lawn, 26 Oct 2014, D. Harries (SAV F-4414).

###### Notes.

*Dermolomasimile* is a member of D.subgenusDermoloma, section Dermoloma. It is sister to *D.carpathicum* (Fig. 2) which is also the most similar species. For the morphological circumscription of *D.simile*, see the notes under *D.carpathicum*. The species was included in the phylogenetic study by Sánchez-García et al. (2021) and labeled as “*Dermoloma* sp. 1” and “*Dermoloma* sp. 2”. Our multilocus phylogeny did not support two species and the support in the previous study was probably based on different ITS sequence variants of *D.simile*.

##### ﻿ Dermoloma vellingae

Taxon classificationAnimaliaAgaricalesTricholomataceae

Adamčík & Matheny sp. nov.

C3F1E990-FAAA-5313-9E45-62819DD354D1

856446

Figs 51a , 52

###### Etymology.

Named in honor of the collector, Dr. Else Vellinga, a distinguished Dutch and North American mycologist.

###### Holotype.

USA • Tennessee, Cocke County, Great Smoky Mountains National Park, Maddron Bald Trail, on acidic soil in mixed forest dominated by *Tsuga*, *Quercus*, *Betula*, *Rhododendron*, 10 Oct 2010, E. C. Vellinga *ECV4208* (TENN-F-065324).

###### Diagnosis.

North American species; basidiomata small to moderately large; pilei 11–22 mm in diameter, mainly brown to dark brown; stipes 2.5–3 mm wide; spores amyloid, 4.3–4.8 μm wide; basidia < 25 µm long and < 6.5 µm wide.

**Figure 51.**
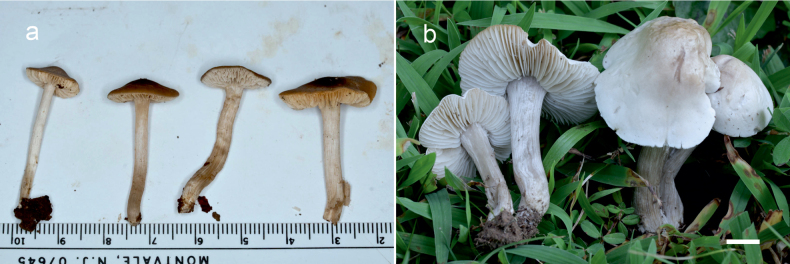
Basidiomata of *Dermoloma* in field appearance. **a***Dermolomavellingae* (TENN-F-065324, holotype), photo E. C. Vellinga; **b***Neodermolomacampestre* (TENN-F-074505, holotype), photo P. B. Matheny. Scale bar: 10 mm.

**Figure 52.**
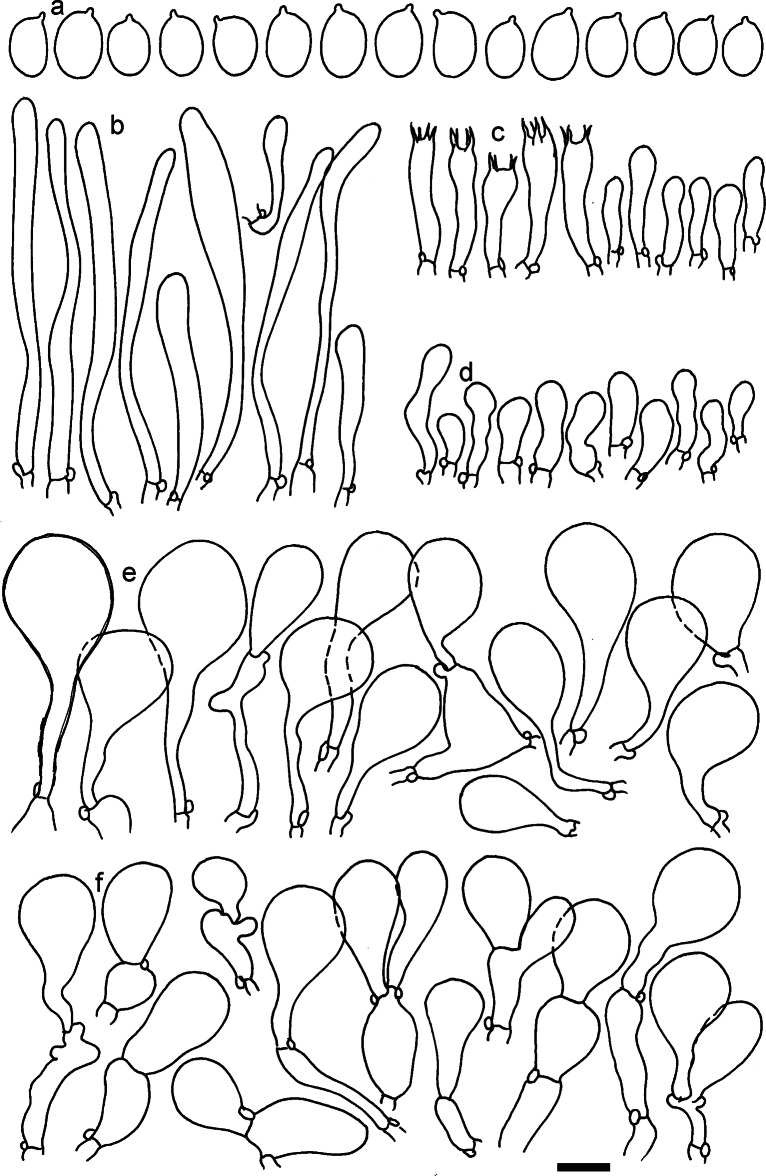
*Dermolomavellingae* (TENN-F-065324, holotype), microscopic elements. **a** Spores; **b** caulocystidia; **c** basidia and basidioles; **d** marginal cells; **e** pileipellis elements near the pileus margin; **f** pileipellis elements near the pileus center. Scale bar: 5 µm for spores and 10 µm for other elements.

***Pileus*** 11–22 mm; plano-convex, umbonate; margin not striate; surface smooth, hygrophanous; color dark brown (6F5) to brown (5F7), paler at margin. ***Stipe*** 20–30 × 2.5–3 mm; cylindrical; surface finely longitudinally striate, finely granulose near lamellae; color pale grayish, towards the base brownish gray when wounded or old. ***Lamellae*** L = 27–30, l = 3; up to 4 mm wide; emarginate; color pale gray; edges entire. ***Context*** when young elastic, later fragile; odor slightly sweetish like apple.

***Spores*** (5.4–)6.1–6.5–6.8(–7.1) × (4.2–)4.3–4.5–4.8(–5.1) μm; ellipsoid to narrowly ellipsoid, Q = (1.23–)1.36–1.43–1.51(–1.67); walls amyloid; hilar appendage ca. 0.5–1 μm long. ***Basidia*** (19–)20.5–23.4–26(–27) × (5–)5.5–6.4–7 μm; clavate, flexuous near the base; with 4 sterigmata. ***Basidioles*** first cylindrical, then clavate, ca. 3.5–6 μm wide. ***Marginal cells*** (11–)12–15.9–19.5(–27) × (4.5–)5–5.9–6.5(–7.5) μm; mainly clavate, often slightly flexuous or moniliform. ***Pileipellis*** 40–65 μm deep; suprapellis 30–45 μm deep, of one or two layers of inflated, densely arranged cells; subpellis 12–17 μm deep, hardly defined, of densely packed, horizontally oriented, 2–7(–10) μm wide hyphae, gradually passing to horizontally oriented hyphae in trama; hyphal terminations with brownish parietal pigments but near septa darker brown and incrusted pigments, thin-walled or with slightly thickened walls up to 1 μm, hyphae in subpellis often with thickened walls up to 1.5 μm. Terminal cells near pileus margin (25–)30.5–40–49.5(–58) × (10.5–)13.5–17.1–20.5(–26) μm; mainly sphaeropedunculate and with very narrow basal part (constricted to 2.5–4 μm), occasionally obpyriform or clavate, sometimes lobate especially towards septa; subterminal cells usually narrower, branched and implemented in subpellis, inflated and fusiform-ventricose, often lobate. Terminal cells near pileus center (20–)24–29.4–35(–38) × (11–)13.5–15.9–18.5(–22) μm; usually obpyriform or clavate, less frequently sphaeropedunculate, sometimes slightly flexuous towards bases; subterminal cells narrower or equally wide, less frequently branched, often with lateral swellings, projections or irregularly lobate. ***Caulocystidia*** (25.5–)43.5–64.4–85(–105) × (3–)3.5–5.2–6.5(–8.5) μm; mainly clavate, rarely subcylindrical, flexuous, individual or clustered in small fascicules, repent and usually with ascending tips; thin-walled, with brownish yellow parietal pigments. ***Clamp connections*** present.

###### Distribution and ecology.

Known from a single locality in Tennessee, USA.

###### Notes.

*Dermolomavellingae* is a member of D.subgenusAmylospora, section Atrobrunnea. It is a member of a distinct clade that includes *D.josserandii* and other taxa with relatively sturdy collybioid and pale-colored basidiomata. The North American species *D.appalachianum* and *D.hymenocephalum* are also members of this clade. *Dermolomaappalachianum* differs from *D.vellingae* by the longer spores. However, *D.hymenocephalum* is very similar and its morphological delimitation will require more observations, including new collections. *Dermolomavellingae* was included in the phylogenetic study by Sánchez-García and Matheny (2017) as *Dermoloma* sp. and later in Sánchez-García et al. (2021) as “*Dermoloma* sp. 9”. The ITS region was very difficult to sequence, probably due to complex secondary structure, and this is probably the reason why this species has not appeared in any public sequence database. Because the collection presented here is well-documented and morphologically distinct, we describe it as new.

##### ﻿ Dermoloma vestigium

Taxon classificationAnimaliaAgaricalesTricholomataceae

Adamčík & Corriol sp. nov.

2F5FD5BF-DE27-5CAA-96C1-A8B28B1C8985

856447

Figs 48e, f , 53

###### Etymology.

Type collected in hoof prints of cows at a pasture entrance.

###### Holotype.

Slovakia • Zvolenská kotlina Basin, pasture E of the village Bečov, elev. 400–450 m, coord. 48°38'48"N, 19°14'49"E, terrestrial at the entrance to the pasture on soil disturbed by cattle hoof prints, 17 Sep 2020, S. Adamčík (SAV F-20662).

###### Diagnosis.

European species; basidiomata formed by this species are the smallest known in the genus; pilei up to 10 mm in diameter; stipes up to 1.5 mm wide; lamellae light brown to dark brown; spores amyloid 5.3–6.2 × 3.5–4.4 μm; caulocystidia on average < 30 µm long and > 8 µm wide.

***Pileus*** (2–)4–­8(­10) mm; when young semiglobose, soon convex to plano-convex, often indistinctly umbonate; margin translucently striate to half of the radius when wet; surface usually smooth, sometimes rugulose, slightly hygrophanous; color near margin brown (5E4, 6E4, 5F7) to dark brown (6F4, 6F5, 7F3, 7F4, 7F4), near center dark brown (6F2, 6F3, 6F4, 6F5) to black, when dry uniformly grayish brown (5D3, 6D3, 6E3), light brown (5D4) to brown (6E4, 6E5). ***Stipe*** (8­–)10­–25(­–30) × 0.5­–1.5 mm; cylindrical, flexuous, grooved; surface finely pruinose near lamellae, glabrous and shiny towards the base; color near lamellae grayish brown (5D3), light brown (5D5), brown (5E5, 6E4, 6E5), near the base dark brown (6F4, 6F5, 8F4). ***Lamellae*** L = (10–­)17–20, l = (0–­)1–­3; 0.5­–2 mm wide; adnate-emarginate and decurrent with tooth; color grayish brown (5D3, 6E3, 7E3), light brown (5D4), brown (6E4, 6E5) to dark brown (6F4, 7F3); edges entire. ***Context*** elastic or fragile; odor farinaceous, sometimes weakly.

**Figure 53.**
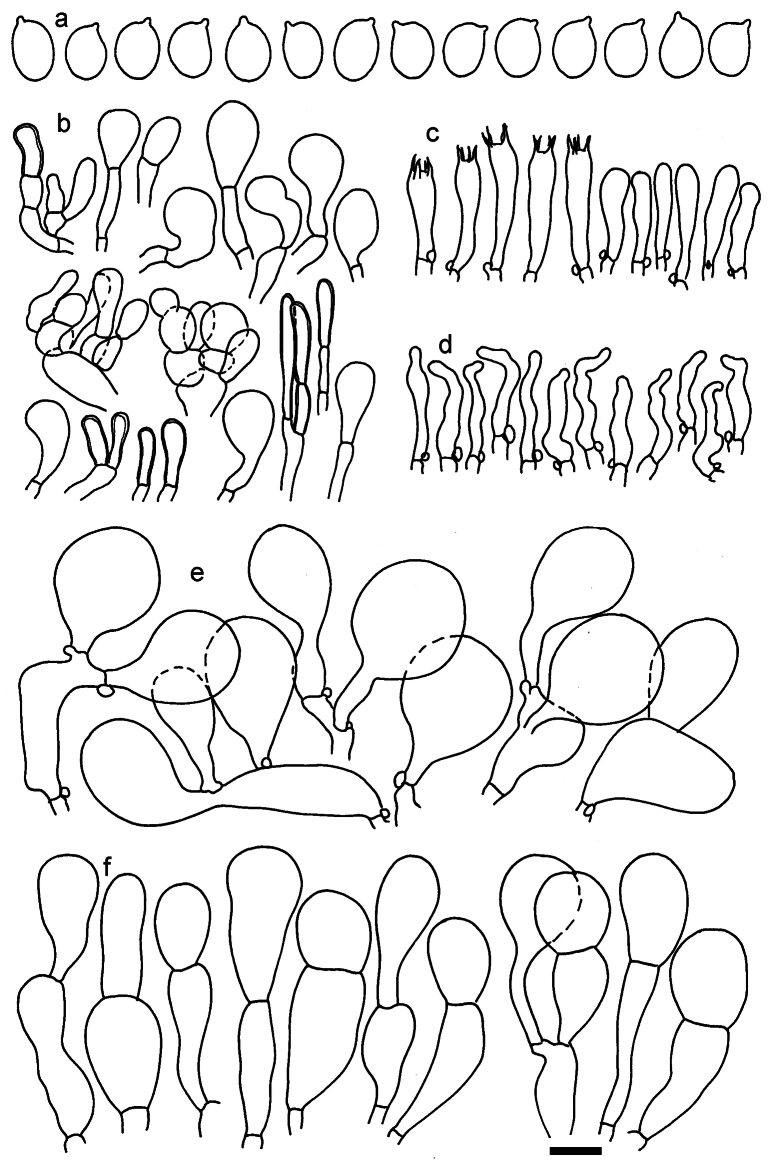
*Dermolomavestigium* (SAV F-4102), microscopic elements. **a** Spores; **b** caulocystidia; **c** basidia and basidioles; **d** marginal cells; **e** pileipellis elements near the pileus margin; **f** pileipellis elements near the pileus center. Scale bar: 5 µm for spores and 10 µm for other elements.

***Spores*** (4.3–)5.3–5.7–6.2(–6.8) × (3.1–)3.5–4–4.4(–5.1) μm; ellipsoid to oblong, Q = (1.22–)1.35–1.45–1.56(–1.89); walls amyloid, few dispersed spores thick-walled and dextrinoid; hilar appendage 0.5–0.8 μm long. ***Basidia*** (17–)18.5–20.8–23(–27) × (4.5–)5–6–7(–8.5) μm; clavate; with 2 sterigmata or with 2 and 4 sterigmata, occasionally also with 1 or 3 sterigmata. ***Basidioles*** first cylindrical, then clavate, ca. 3–6 μm wide. ***Marginal cells*** (11–)17–20.1–23(–30) × (2.5–)3–4–4.5(–7) μm; mainly cylindrical or lageniform, occasionally narrowly fusiform, flexuous, occasionally nodulose or slightly moniliform, apically often constricted or with an appendage. ***Pileipellis*** 70–90 μm deep; suprapellis 35–45 μm deep, usually of one or two layers of inflated, densely arranged cells; subpellis well-differentiated, 35–55 μm deep, of densely packed, puzzled, irregularly oriented, 2.5–10 μm wide hyphae, sharply delimited from horizontally oriented hyphae in trama; hyphal terminations with yellow-brown parietal pigments, sometimes also incrusted and locally with darker brown pigments, walls thickened up to 0.5 μm, near septa of terminal cells and in subpellis up to 1 μm. Terminal cells near pileus margin (13–)23.5–32.7–42(–54) × (9–)11.5–14.7–18(–22.5) μm; usually obpyriform or sphaeropedunculate, occasionally clavate or subglobose; subterminal cells often branched, cylindrical, fusiform or clavate, often inflated-ventricose, often with lateral swellings, branches or lobate. Terminal cells near pileus center (17–)24.5–31.8–39(–49) × (9–)11.5–15.4–19.5(–30) μm; subglogose, obpyriform, clavate or ellipsoid; subterminal cells often branched, mainly ellipsoid or fusiform-ventricose, often lobate. ***Caulocystidia*** (6–)12.5–19.3–26(–38) × (3.5–)5.5–8.6–11.5(–16) μm; variable, cylindrical, clavate, obpyriform, sphaeropedunculate, subglobose, ellipsoid, usually in dense clusters, erect or ascending, subterminal cells often inflated; thin-walled or slightly thickened up to 0.5 μm, with brown parietal and sometimes also incrusted pigments. ***Clamp connections*** present.

###### Distribution and ecology.

Known from Finland, France, Germany and Slovakia; in semi-natural grasslands on acidic to calcareous soil, once found in a calcareous forest. Several collections, including the holotype, grew on disrupted soil in or around hoof prints.

###### Additional material studied.

Finland • Laatokan Karjala, Saari, church yard, coord. 61°39'11"N, 29°44'46"E, in open site in forest with *Piceaabies*, *Pinussylvestris*, *Betula*, *Salixcaprea* and *Salix* sp., on calcareous ground, gravel soil, 24 Sept 2004, J. Vauras *FISAP923-14* (TUR170268). France • Hautes-Pyrénées, Bagnères-de-Bigorre, Cérétou, coord. 42°59'23"N, 00°05'44"E, acidophilic grazed grassland (*Nardion*), 11 Oct 2014, G. Corriol *GC14101103* (BBF, as *D.pseudocamarophyllopsis*). Germany • Rheinland-Pfalz, Horbach, elev. 370 m, coord. 49°49'44"N, 07°31'17"E, terrestrial on grassland entrance, 8 Nov 2019, S. Adamčík (SAV F-20505). Slovakia • Laborecká vrchovina Mts., pasture 1 km SSE of Osadné, elev. 400 m, coord. 49°08'07"N, 22°09'10"E, terrestrial, 12 Aug 2011, S. Adamčík (SAV F-4102); • Zvolenská kotlina Basin, pasture E of the village Bečov, elev. 400–450 m, coord. 48°38'48"N, 19°14'49"E, terrestrial, 17 Sep 2020, S. Adamčík (SAV F-20663); • ibid., 17 Sep 2020, S. Adamčík (SAV F-20664).

###### Notes.

*Dermolomavestigium* is a member of D.subgenusAmylospora, section Atrobrunnea. It is among the smallest *Dermoloma* species known. Unique characters of this species are the short and relatively broad caulocystidia contrasting with the narrow apically constricted marginal cells. The sister species *D.rostratum* (Fig. 2) also has small basidiomata, but it is clearly distinguished by the narrower caulocystidia and often nodulose marginal cells. The species was included in the phylogenetic study by Sánchez-García and Matheny (2017) as “*Dermoloma* sp.” but was incorrectly labelled as SAV4103. Later, the same sample was correctly labelled as SAV F-4102 in Sánchez-García et al. (2021) as “*Dermoloma* sp. 6”. The holotype is selected with respect to the best set of DNA regions and sufficient amount of basidiomata.

##### ﻿ Neodermoloma

Taxon classificationAnimaliaAgaricalesTricholomataceae

Sánchez-García, Matheny & Adamčík gen. nov.

78085B15-D48E-54B1-8299-DD0B5E9F2905

856448

###### Etymology.

Similar and related to the genus *Dermoloma*, the prefix *neo* refers to the origin of the type species in the New World, which was used as popular name for the Americas.

###### Type species.

*Neodermolomacampestre*

###### Diagnosis.

Basidiomata agaricoid; hyphae with clamp connections; spores hyaline, smooth, amyloid; pileipellis an epithelium composed of inflated cells; similar and related to *Dermoloma* from which it differs in pale gray colors and smaller, inflated elements in the pileipellis.

##### ﻿ Neodermoloma campestre

Taxon classificationAnimaliaAgaricalesTricholomataceae

Sánchez-García, Matheny & Adamčík sp. nov.

9B0BCC05-A67C-57EB-97AB-5825BFEEB77A

856449

Figs 51b , 54

###### Etymology.

In reference to occurrences in grassy lawns.

###### Holotype.

USA • Tennessee, Maynardville, Union Co., Big Ridge State Park, on soil in a lawn, 11 Aug 2018, P. B. Matheny & S. R. Warwick *PBM4177* (TENN-F-074505).

###### Diagnosis.

North American species; basidiomata relatively large, pale colored; color prevailingly ochraceous-gray to grayish brown; pilei 20–45 mm in diameter; stipes 4–11 mm wide; lamellae 42–48 near the stipe; pileipellis with similar cellular structure as found in *Dermoloma* but with terminal elements mainly < 12 µm wide.

***Pileus*** 20–45 mm; obtusely conical, campanulate, expanding to plane, at times with a low obtuse umbo; margin decurved to straight, not striate; surface smooth, near center rough to bumpy, dry, dull, not noticeably hygrophanous; color near margin ochraceous-gray (6B2) or light grayish drab, towards center brown (6E4), often with a bluish-gray tone. ***Stipe*** 40–65 × 4–11 mm; cylindrical and slightly tapering towards the base, equal, not bulbous, solid or stuffed; surface dry, veil absent, finely fibrillose, easily splitting with age; color streaked gray-drab with white areas, overall light brownish gray (5D2) to grayish brown (5E3), whitish at extreme point of attachment. ***Lamellae*** L = 42–48, l = 1–3; up to 11 mm wide; sinuate to adnate; color yellowish white ochraceous-gray (6B2) to brown (6E4); edges entire, concolorous. ***Context*** compact, flesh grayish, not changing color when bruised; odor unpleasant like a dirty dish rag, taste farinaceous. Negative reaction with KOH and PDAB.

**Figure 54.**
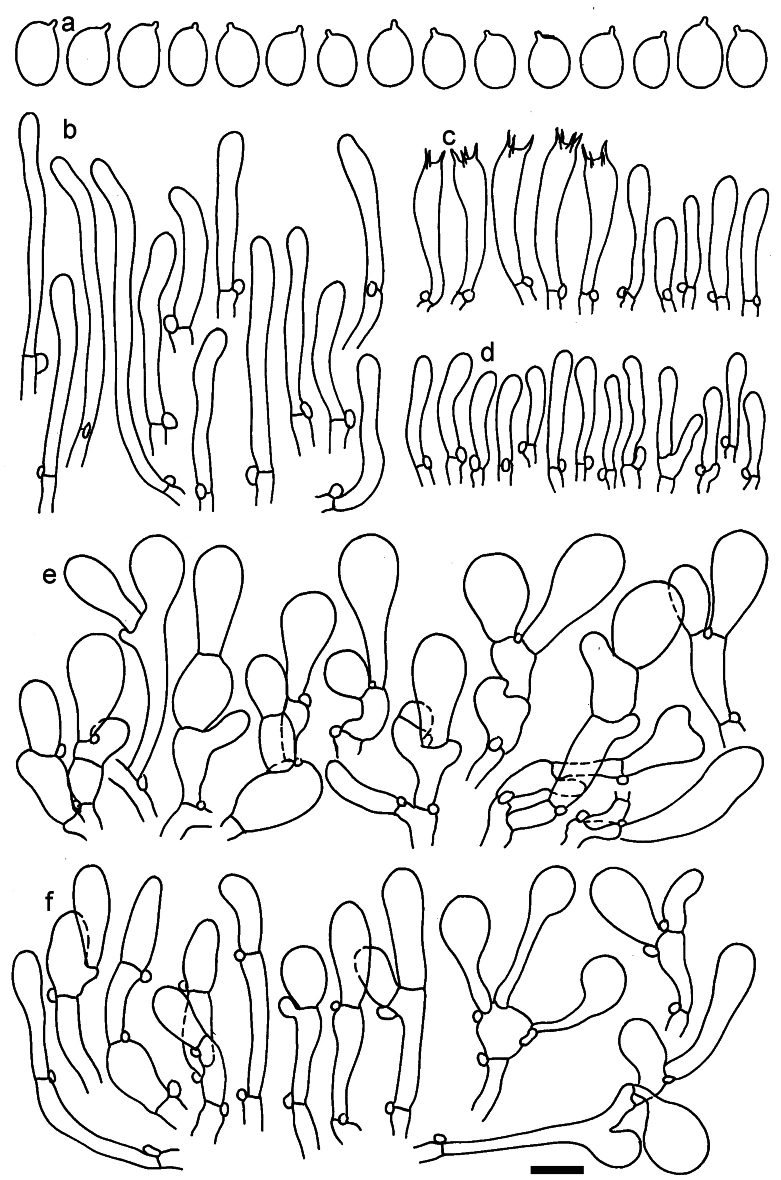
*Neodermolomacampestre* (TENN-F-066899), microscopic elements. **a** Spores; **b** caulocystidia; **c** basidia and basidioles; **d** marginal cells; **e** pileipellis elements near the pileus margin; **f** pileipellis elements near the pileus center. Scale bar: 5 µm for spores and 10 µm for other elements.

***Spores*** (4.4–)4.7–5.2–5.7(–6.8) × (3.5–)3.8–4–4.3(–4.7) μm; broadly ellipsoid to ellipsoid, Q = (1.15–)1.21–1.30–1.38(–1.58); walls amyloid; hilar appendage 0.7–1 μm long. ***Basidia*** (20–)22.5–27.1–31.5(–39) × (5–)5.5–6.4–7(–8) μm; clavate; with 4 sterigmata, occasionally with 2 sterigmata, rarely 1 or 3 sterigmata. ***Basidioles*** first cylindrical, then clavate, ca. 3–7 μm wide. ***Marginal cells*** (13–)15.5–18.6–21.5(–25) × (2.5–)3.5–4.4–5.5(–7) μm; not well-differentiated and similar to Basidioles but narrower, mainly cylindrical or clavate, apically obtuse, often slightly flexuous. ***Pileipellis*** 50–77 μm deep; suprapellis 47–57 μm deep, usually of multiple layers of inflated and densely arranged but near surface loose cells; subpellis not well defined, 14–23 μm deep, of densely packed, horizontally oriented, intricate, narrow, 3–6 μm wide hyphae, sharply delimited from horizontally oriented and intricate hyphae in trama; hyphal terminations with brownish yellow parietal, in subpellis also incrusted but not darker pigments, walls thickened up to 0.5 μm. Terminal cells near pileus margin (12–)17–24.8–32.5(–45) × (6–)9–10.9–12.5(–15.5) μm; usually obpyriform, occasionally clavate or ellipsoid, rarely sphaeropedunculate; subterminal cells branched or not, ventricose or cylindrical, occasionally inflated, often lobate or with lateral swellings. Terminal cells near pileus center (11–)15–23.1–31.5(–47) × (4.5–)7.5–9.2–11(–13) μm; clavate, obpyriform or sphaeropedunculate, occasionally fusiform; subterminal cells mainly cylindrical or narrowly clavate, occasionally ventricose. ***Caulocystidia*** (21–)26.5–37.4–48.5(–72) × (4–)4.5–7.2–10(–14) μm; narrowly clavate or subcylindrical, occasionally slightly flexuous, repent or with ascending tips, dispersed, individual or in small clusters; thin-walled, with pale brownish yellow parietal pigments, on subterminal cells also incrusted pigments. ***Clamp connections*** present.

###### Additional material studied.

USA • South Carolina, Weinnsboro, on soil in a lawn, 27 Aug 1966, C. Lyles *RHP29387* (TENN-F-029387, as *Dermolomahymenocephalum*); • Tennessee, Maynardville, Union Co., Big Ridge State Park, on soil in a lawn, 26 Sep 2011, B. Williams *BW8* (TENN-F-066899).

###### Notes.

The North American species *N.campestre* is the only known member of the genus. It is characterized by a pileipellis structure similar to that seen in *Dermoloma*, but with terminal cells that are significantly narrower (up to 12 μm) than those observed in any species of the latter genus. The relatively large and robust, pale colored basidiomata strikingly resemble those of *D.hygrophorus*, which differs by more distant lamellae (L < 30). The species was included in the phylogenetic study by Sánchez-García and Matheny (2017) and Sánchez-García et al. (2021) as “*Dermoloma* sp.”.

### ﻿﻿Excluded and dubious names

Among the 23 validly published *Dermoloma* names from Europe and North America at species and lower rank prior to this study, we were able to obtain ITS sequences from type collections of eleven taxa. Sánchez-García et al. (2021) demonstrated that the sequence from the type of D.intermediumvar.coniferarum Bon does not belong to the genus *Dermoloma*. For the remaining 11 *Dermoloma* species (all from Europe) no sequence data from type specimens were available.

Based on morphological observations of type specimens, six species names were assigned to phylogenetically defined species recognized here. The following names are treated as accepted: *D.bellerianum*, *D.fuscobrunneum*, *D.intermedium*, *D.josserandii*, *D.magicum* and *D.murinellum*. In order to stabilize these species concepts, we chose recently collected and sequenced collections as epitypes for each of them.

We were unable to match our recent material with a number of *Dermoloma* species based on our observations on type specimens. Among these were *D.aposcenum* Singer, *D.atrobrunneum* (Dennis) Singer ex Bon, *D.hymenocephalum* and *D.pragense* Kubička, which were probably not represented amongst the samples collected or examined during the present study, but they probably represent distinct *Dermoloma* species.

***Dermolomaaposcenum*** Singer described from a tropical rainforest site in Mexico has inamyloid spores measuring 5.8–7 × 4–4.5 μm, according to the protologue (Singer 1989). The habitat alone and spore size compared to other North American species serve to distinguish it, although we did not study the type.

***Dermolomaatrobrunneum*** (Dennis) Singer ex Bon was described from Trinidad (Dennis 1951) and it is the type species of D.sectionAtrobrunnea, which includes species with amyloid spores. Sequencing of the type specimen failed, but its morphology clearly confirmed the presence of amyloid spores and a cellular pileipellis typical for the D.subgenusAmylospora. The species differs from other amyloid North American taxa by the small broadly ellipsoid spores measuring on av. 5.0 × 4.0 μm, Q = 1.26, and by the diverticulate, branched or coralloid, and narrow (ca. 3–4 μm wide) marginal cells.

***Dermolomahymenocephalum*** (A.H. Smith) Singer was the only species described from temperate North America prior to our study. It was originally placed in the genus *Collybia* by Smith (1941) and later hesitantly combined in *Dermoloma* by Singer (1962). Singer (1975) classified the species in D.sectionAtrobrunnea with some hesitation. Based on his doubts and suggestions, Redhead (1984) combined this species into the genus *Hydropus*. Recently, Sánchez-García et al. (2021) confirmed that the species clearly belongs in *Dermoloma* after molecular annotation of the type. Results of our phylogenetic analyses are congruent with the latter study. *Dermolomahymenocephalum* is clearly different from the four North American species described as new here and is clustered with four more collections from Smith’s herbarium. Based on our type study, *D.hymenocephalum* differs from the other two North American *Dermoloma* species with amyloid spores described here by the shorter spores. However, spores and also other macro- and micromorphological characters of *D.vellingae* are very similar and these two species belong to the *D.josserandii* clade, so their morphological delimitation requires more observations.

***Dermolomapragense*** Kubička described from Czechia (former Czechoslovakia), was originally recognized by the amyloid and relatively small spores. There was nomenclatural confusion about the validity of the name because it appeared in a key without a detailed description (Kubička 1975). However, the diagnostic characters of the species are described in Latin as “Sporae amyloideae: Sp. 5–6 × 3.5–4.5 μm” and there is a reference to the type specimen (PRM611173) which complies with the requirements for valid publication (Turland et al. 2018, Art. 39.1 of the Code). Bon (1986) later intended to validate the name at varietal rank as D.pseudocuneifoliumvar.pragense Bon. He, however, selected his own collection as the type. Ballero and Contu (1988) combined Bon’s variety at the species rank and their name is a heterotypic homonym of Kubička’s name. Our type study is based on the type specimen designated by Kubička (1975) and previously reported by Svrček (1966) as *D.cuneifolium*. The type has very small spores (on av. 4.8 × 3.8 μm, Q = 1.25) which agrees only with the spore dimensions of *D.parvisporum*, but *D.pragense* differs in having much larger basidiomata (pilei 30 mm in diam., stipes 5–6 mm wide) according to Svrček (1966). In our opinion, *D.pragense* may represent a taxon that is not represented among our recent collections.

Based on available sequences from type collections, our observations on type specimens, or based on original diagnoses, some species have dubious concepts or are not members of the genus *Dermoloma. Dermolomacuneifolium* var. punctipes Arnolds and *D.pseudocuneifolium* Herink ex Bon are names of dubious concepts, previously misinterpreted in the literature, while *D.longibasidiatum* Consiglio, Contu & Setti is probably a later synonym of *D.atrocinereum*. In addition, *D.hybridum* (Kühner) Bon, *D.inconspicuum* Dennis and D.intermediumvar.coniferarum Bon are not members of the genus *Dermoloma*.

***Dermolomacoryleti*** Singer & Clémençon was described from the Czech Republic as a species associated with *Corylus* with inamyloid spores similar to *D.atrocinereum*, but its spores are supposedly narrower (7.2–9.7 × 3.3–3.7 μm) (Singer and Clémençon 1971). We were unable to access the type specimen from the Field Museum of Natural History (Chicago, USA). However, the spores of *D.coryleti* are longer than we observed in any other species of D.subgenusDermoloma which are seldom longer than 7 μm on average (Fig. 7). The species with the longest spores in Europe (longer than 7 μm) is *D.magicum*, which is also reminiscent of *D.coryleti* in its field appearance, but it differs in having amyloid spores on average wider than 4 μm and also in the blackening of the context after handling. However, some other information in the original diagnosis, for instance the odorless context suggests that this species is probably not a member of the genus *Dermoloma*. Because of its distinctive spores, *D.coryleti* was accepted by many authors, but after its original description, this species has only been reported twice, by Kubička (1975) from the Czech Republic and by Ricek (1989) from Austria.

**Dermolomacuneifoliumvar.punctipes** Arnolds was originally recognized from the type variety by darker punctuations of the stipe and darker incrusted pigments on the caulocystidia (Arnolds 1992). We did not observe these characters in any collections identified as D.cuneifolium from sequence data, thus var. punctipes probably corresponds to another *Dermoloma* species. Stipes with darker granulations were observed in several species with inamyloid spores, including *D.atrocinereum*, *D.carpathicum* and *D.fusipes*, but these darker dots were usually near the stipe base. Sequencing of the type of var. punctipes failed, and sequences of collections identified by authors of this study as var. punctipes resulted in matches with *D.atrocinereum*, *D.fusipes* and *D.intermedium*. Spores of the type specimen were on av. 6 × 4.4 μm in size according to our observations, which was the best match for *D.atrocinereum* among species with darker punctuations on the stipes. We did not decide about the taxonomic status of this variety, but it is clear that the taxonomic concept based only on darker dots on the stipe corresponds to more than one species.

***Dermolomahybridum*** (Kühner) Bon was described as *Tricholomahybridum*Kühner (1947) and defined by pilei 70–80 mm in diam., context with no odor and a suprapellis (referred in the original description as epicutis) of cylindrical hyphae. Bon (1979b) combined this species in *Dermoloma*, although these characters clearly contradict the definition of the genus. DNA extraction of the type specimen failed, but our type study confirmed that the pileipellis structure is a cutis composed of chains of ellipsoid inflated cells, which is more typical for other *Tricholomataceae* members, including the genus *Tricholoma*. Accordingly, *T.hybridum* is cited as synonym of *T.terreum* (Schaeff.) P. Kumm. in a French check-list published by Moreau et al. (2023).

***Dermolomainconspicuum*** Dennis, described from Venezuela, was the first member of the genus included in a phylogenetic study and placed close to *Lepiota* (Pers.) Gray in the family *Agaricaceae* Chevall (Kropp 2008). Our phylogeny confirmed the previous results of Sánchez-García et al. (2021) that the majority of studied *Dermoloma* species, including the type species, belong to the family *Tricholomataceae* and that *D.inconspicuum* is not a member of this genus. The species was placed in the genus *Dermoloma* based on a hymeniderm pileipellis composed of relatively narrowly clavate, 10–12 μm wide terminal cells (Dennis 1961). The morphology of the type specimen also strongly suggests that this is not a member of the genus *Dermoloma*, because of relatively narrow, on av. only 7–9 μm wide terminal cells in the pileipellis with frequent appendages that are often branched.

**Dermolomaintermediumvar.coniferarum** Bon was shown not be a member of the genus *Dermoloma*. Previous sequencing of the type indicated that it was identical with *Pseudolaccariapachyphylla* (Fr.) Vizzini & Contu (Sánchez-García et al. 2021). Our morphological observations of the type specimen revealed the presence of coralloid hyphal terminations in the pileipellis mixed with large, incrusted and inflated elements, which demonstrated that this taxon has a very different pileipellis structure compared to *Dermoloma*.

***Dermolomalongibasidiatum*** Contu, Consiglio & Setti described from Italy, was defined morphologically as a species similar to *D.atrocinereum* but distinguished by longer basidia (Contu et al. 2008). Type sequencing failed, but according to our morphological type examination, the length of the basidia fell within the range of *D.atrocinereum*, and thus we treat *D.longibasidiatum* as a later synonym of this species.

***Dermolomapseudocuneifolium*** Herink ex Bon was first introduced first by Herink (1958) as an invalid name (no Latin description) and later adopted by Bon (1986). His concept was based on a misapplication of *D.cuneifolium* as a species with amyloid spores by Josserand (1943) based on French material. Bon (1986) designated his collection as the type, and the protologue as well as Bon’s notes attached to the type specimen both describe the spores as amyloid, 7.5–9 × 4–5 μm. Our type sequencing failed, but the type specimen (a single basidiome) had bisporic basidia without clamp connection and inamyloid, narrow spores, on av. 5.2 × 3.5 μm, Q = 1.49. These spores clearly match the species labeled in our study as D.aff.bellerianum, represented by a single collection (see notes under this species). The name *D.pseudocuneifolium* was intended to be used as a nomenclatural replacement for a taxon similar to *D.cuneifolium* but with amyloid spores. However, inamyloid spores of its type specimen are contrary to the current name use for a species with amyloid spores (Wilhelm 1992; Arnolds 1993, 1995; Contu et al. 2008; Sánchez-García et al. 2021). Therefore, we consider it here a *nomen dubium*.

## ﻿﻿Discussion

### ﻿﻿Phylogeny

Our multilocus phylogenetic analysis using the *Tricholomataceae* dataset (Fig. 3) placed *Dermoloma* into *Tricholomataceae* s.s., which corresponds to its previous placement by Sánchez-García and Matheny (2017) and Vizzini et al. (2024, 2025). The majority of species with typical *Dermoloma* morphology (i.e. gray-brown agarics with lamellate hymenium, fragile context, farinaceous odor, small and smooth, thin-walled spores and a hymeniderm to epithelium pileipellis composed of inflated cells) formed a monophyletic group in our phylogeny, which corresponds to the previous phylogenetic definition of the genus (Sánchez-García et al. 2021). A single species with *Dermoloma* morphology, placed as sister to *Pseudotricholoma*, was already recognized in the phylogeny of Sánchez-García et al. (2021) and is formally described here as a single member of the new genus *Neodermoloma*. Despite *Neodermoloma* and *Dermoloma* being morphologically very similar, the former deserves the status of an independent genus because, based on our phylogeny, it is related to *Pseudotricholoma* and both genera form a supported clade that is sister to *Dermoloma*. Both *Neodermoloma* and *Pseudotricholoma*, also occupy long branches in our tree (Fig. 2), which indicates that they possibly group based on long-branch attraction. Other related *Tricholomataceae* genera, *Pseudotricholoma* and *Pseudoporpoloma* Vizzini & Consiglio have pileipellis structure of a cutis type, which make them morphologically very different from *Dermoloma* and *Neodermoloma* (Sánchez-García et al. 2014; Vizzini et al. 2016).

Infrageneric classification into two subgenera (*Dermoloma* and *Amylospora*), supported by our phylogeny, also corresponds to Sánchez-García et al. (2021), with the exception of *D.magicum*, whose placement is not resolved within D.subgenusAmylospora (Figs 2, 3). It is possible that the lack of the support for the latter species is due to poor taxon sampling outside Europe and North America. *Dermoloma* is also known from Africa, South America, Asia, Australia and Oceania (Sánchez-García et al. 2021), therefore, taxon sampling within the genus is not complete. However, some species described from tropical areas might actually represent members of other genera or families. This was demonstrated by Kropp (2008) who placed *D.inconspicuum* into the *Agaricaceae*, which also corresponds to our observations on its pileipellis morphology that differs from *Dermoloma* s.s. According to our type study of *D.atrobrunneum*, described from Trinidad, it is a typical member of *Dermoloma* with amyloid spores, and its classification as the type species of what is recognized here as D.sectionAtrobrunnea is justified. Our phylogeny also confirmed that the phylogenetic distance between the *D.magicum* and *D.bellerianum* clades is distinct and appropriate for ranking them as sections, as proposed by Sánchez-García et al. (2021).

We used genealogical concordance to test species hypotheses. In the majority of cases, species were supported across the phylogenetic analyses of six individual DNA regions. In some cases, support of the initial ITS phylogeny was not confirmed. For example, the previous phylogeny published by Sánchez-García et al. (2021) recognized two species labeled as “*Dermoloma* sp. 1” and “*Dermoloma* sp. 2”, but these species were not supported by the new multilocus phylogeny. Furthermore, no morphological differences could be detected to separate them. Thus, in our current study they are presented as a single species, *D.simile*. The ITS analysis of *Dermoloma* usually showed more supported clades than multilocus analyses, and we did not observe any species without support in ITS analysis but supported by other gene phylogenies, as has been shown for other *Basidiomycota* (Badotti et al. 2017). This indicates that the ITS region is a reliable tool for *Dermoloma* barcoding in most instances. High intraspecific variability in some regions or lack of support in other regions contribute at times to low support for recognition of phylogenetic species in our multilocus analysis. In case of low support, we applied approaches of integrative taxonomy using morphological, ecological and biogeographical traits to evaluate a species hypothesis (Stengel et al. 2022). *Dermolomajosserandii* and *D.pseudojosserandii* are two species that did not receive strong support in the multilocus phylogeny. However, morphological differences corresponded to clusters in the tree. In contrast, two clades recognized in the multilocus phylogeny are treated as the single species *D.pruinosipes*. In this case, we were unable to recognize any morphological differences between the two clades of *D.pruinosipes* and not all gene trees could distinguish them.

### ﻿﻿Morphology and *Dermoloma* species richness in Europe and North America

In the most recent species-level identification keys, the species richness of *Dermoloma* in different parts of Europe is estimated between three (Vesterholt 2012) and 18 species (Contu et al. 2008; a specific study on *Dermoloma*). In our study, 30 European species are presented, but this does not include another two species described prior to this study that were not collected by us (*D.clavicystis* and *D.pragense*) and the undescribed species *Dermoloma* sp. represented by a single collection CNF 1/6187 from Croatia. This number of 33 species in Europe is even higher than the highest expectations (Contu et al. 2008), and the existence of even more undescribed species is to be expected due to limited sampling in some areas of Europe (Mediterranean, boreal regions, Baltic and East Europe).

Despite the relatively low number of species previously recognized by European authors, several species names are recognized here as synonyms. The presence of synonyms and dubious species concepts are due to a range of reasons, which include the lack of type studies, taxa described only briefly in the older literature, material restricted to one or few species collected from a limited area, lack of sequence data, lack of accurate morphological data or overemphasis of some morphological characters. The present study on character variation has clearly shown that most *Dermoloma* species are morphologically differentiated, although a few are not completely distinguishable and therefore require sequence barcoding for identification. Because of obscure species concepts in the literature, which do not allow unambiguous species identifications, we focused primarily on type and authentic material to solve *Dermoloma* nomenclature, but we did not study concepts of described species reported by other mycologists. Among overemphasised characters in the literature, the most frequently used was color, as some species are even both named and defined by colors. For example, *D.fuscobrunneum* is distinguished by a dark brown pileus and *D.phaeopodium* by dark (stipe) colors (Orton 1980). However, the color often differs between the margin and the center of pileus or between fresh and dry conditions. Our chromatic analysis (Fig. 6) demonstrated that this character can be used only with some caution, but we found that the best areas showing differences between species were surfaces of lamellae and the upper part of the stipe. Basidiome size was also considered to be important for species identifications. We demonstrated that dimensions of basidiomata are crucial and efficient for the species identification in the field.

In this study, we provided for the first time all described species with observations on marginal cells near the lamellar edges, caulocystidia and pileipellis structure. While the latter character did not show differences between species, caulocystidia and marginal cells are in some cases used in our study for diagnostic identification. For example, *D.rostratum* differs from similar species by lobate and diverticulate caulocystidia and marginal cells. The most recent species described prior to our study was *D.clavicystis*, supposedly differing from other species by the presence of clavate cheilocystidia (Voto 2022). It is true that our phylogenetic analysis showed that the ITS sequence of this species is unique and close to *D.pusillum*, but the presence of cheilocystidia (in our study these structures are called marginal cells) was observed in the majority of D.subgenusAmylospora to which this species belongs. Thus, based on the original description, we were unable to identify differences between *D.clavicystis* and other species described there.

Studies of similar genera with brown agaricoid basidiomata and high species richness, for instance *Conocybe* (Song and Bau 2023), *Hebeloma* (Beker et al. 2016), *Inocybe* (Matheny et al. 2023) and *Psathyrella* (Örstadius et al. 2015; Örstadius 2023), also make a significant effort to standardize, code and use microscopic characters for species identifications. Unlike *Dermoloma*, these genera are characterized by additional features such as variation in developmental traits (veils), hymenial cystidia and pileipellis structure. More similar to *Dermoloma* is *Hodophilus* which lacks veils and hymenial cystidia, has a white spore deposit and exhibits almost no differences in the pileipellis structure. Studies on *Hodophilus* also rigorously used precisely defined morphology and statistically supported differences, because the morphological characters are sometimes variable and need to be considered with special care (Adamčík et al. 2016, 2017a, 2017b, 2018, 2020).

Because similarities in basidiomata morphology do not necessarily reflect phylogenetic relationships, morphological characteristics have lost some of their former significance in taxonomy (Kües and Navarro-González 2015). However, some morphological characters may represent adaptations linked to functional traits. These traits may represent early evolutionary responses to adaptation to certain changing environmental factors and may be important functional traits that are not yet understood properly (Halbwachs et al. 2016). Fungi exhibit frequent transformations and convergences in their morphological characteristics as response to ecological or trophic adaptations (Nagy et al. 2011; Virágh et al. 2022). Understanding these evolutionary processes requires implementation of integrative taxonomy analyzing phylogenetic/phylogenomics, morphological and ecological data using both bioinformatic and modelling approaches (Stengel et al. 2022; Miller et al. 2023). However, our study on *Dermoloma* is pioneering, and we did not link taxonomic data with soil, climate or other ecological preferences to recognize morphological adaptations of individual species. Understanding evolutionary adaptations may also help to understand importance of morphological characters for species delimitation (Manz et al. 2025).

Almost all morphological structures used for species circumscription in the genus *Dermoloma* show overlapping variation for the majority of species. Therefore, they can be used for species identification only in combination with other characters. We believe that our key to *Dermoloma* and *Neodermoloma* provides in most cases well defined morphological species delimitations, but in some related taxa these limits are critical and identifications should be confirmed by sequence data (ITS region). Such examples would be the species pairs *D.simile* and *D.carpathicum*, *D.intermedium* and *D.fusipes* or *D.josserandii* and *D.pseudojosserandii*. Limited sampling in some studied species and undiscovered species may change our understanding of morphological species limits and we expect the emergence of more cryptic or semi-cryptic species.

### ﻿﻿Ecology and distribution

Our phylogeny reinforces intra-continental scale endemism, which agrees with observations on other agaricoid CHEGD genera, i.e. *Hodophilus* (Adamčík et al. 2016, 2018), *Hygrocybe* s.l. (Lodge et al. 2014) and EntolomasubgenusNolanea (Reschke et al. 2022). We detected no inter-continental distributions among species of *Dermoloma* These studies also reported contrasting ecological preferences of CHEGD fungi in Europe where they are typically found in oligotrophic (‘ancient’) semi-natural grasslands, and in America where they occur in rich mull-soil forests, often dominated by non-ectomycorrhizal tree species. In our study, the European species are frequently documented by collections from (calcareous) forest sites, indicating that their association with open grassland is preferential, but not strict. In Europe, some *Dermoloma* species showed contrasting regional patterns. For example, *D.pusillum* and *D.clavicystis* were only reported from Mediterranean areas (Contu et al. 2008; Voto 2022), while *D.parvisporum* and *D.angustisporum* were only found in humid areas of Central and Northern Europe (this study). We also observed some differences in *Dermoloma* diversity in similar grasslands in temperate areas between West and East Europe. *Dermolomacuneifolium* is very frequent in the United Kingdom, north of France and Sweden, collected frequently as the only *Dermoloma* species in less pristine and more managed grasslands (e.g. cemeteries), but it was collected only at a single location in Slovakia. In contrast, the closely related *D.carpathicum* is frequent in Slovakia and Romania in semi-natural grasslands rich in CHEGD fungi, but aside from these two countries we studied only a single collection from Germany. These observations might represent hints for climate preferences, but the majority of *Dermoloma* species seem to be widely distributed across Europe.

During our study we have seen much more frequent occurrence of *Dermoloma* in European grasslands than was reported in earlier mycological field surveys (Griffith et al. 2012; Hay et al. 2018). At several sites we collected more than five species of *Dermoloma* in a single day. At least some metabarcoding studies, analyzing eDNA from European grasslands, reported not only multiple species in soil samples, but also a high relative abundance of the genus. For example, three large tricholomatoid clades labelled as “Tricholom 8, 9 and 10” retrieved from Mediterranean grassland in Northern Italy by Voyron et al. (2017) correspond to three *Dermoloma* species.

The occurrence of *Dermoloma* in North America (USA) is very different from Europe. We collected *Dermoloma* in presence of other CHEGD fungi but often also with other *Tricholomataceae* like *Pseudotricholoma* and *Dennisiomyces*. Both of these genera are associated with CHEGD fungi in our experience, but they may be even more frequent than *Dermoloma* in North America in sites inhabited by other CHEGD species and may be competitors of *Dermoloma* causing lower vacant niche spaces and reduced resource availability. In our experience, *Dermoloma* and CHEGD fungi are rare or absent in temperate grasslands in North America, and at least members of D.subgenusDermoloma, which are frequent in Europe, are very rare in the USA. More observations are needed to prove if our preliminary conclusions are correct, and especially more attention is necessary to collect *Dermoloma* in a wide range of habitats in North America. *Dennisiomyces* is very rare in Europe (Corriol and Jargeat 2019), and although *Pseudotricholomametapodium* is relatively frequently reported in Europe (Griffith et al. 2013), this might be due to its larger conspicuous basidiomata and also because some collections were confused with *Dermolomamagicum* in the past (Arnolds 2002).

Species of *Dermoloma* have a similar isotopic signal of δ^13^C and δ^15^N to two other CHEGD groups, *Hygrocybe* s.l. and *Clavariaceae*, which suggests that these fungi are biotrophic but not ectomycorrhizal. Similarly, other *Tricholomataceae* members which were not included in such analyses, for instance *Pseudotricholoma* and *Pseudobaeospora*, may also share the same trophic strategy (Caboň et al. 2021). This suggests that the trophic strategy of CHEGD fungi evolved independently in multiple *Tricholomataceae* lineages similarly to independent shifts of the ectomycorrhizal habit (Sánchez-García and Matheny 2017). It seems that the interaction of CHEGD fungi with their potential plant partners is mutualistic, because they preferably occur in oligotrophic grasslands and their diversity decreases with higher level of available organic material (Griffith et al. 2013; Halbwachs et al. 2018; Caboň et al. 2021).

In Europe, we occasionally observed occurrences of sister species at a single site. For example, *D.carpathicum* and *D.simile* were collected in a single day, 22 Oct 2020, at Zvolenská Slatina, Slovakia and 3 Oct 2021 at Schachenheide, Germany. We did not observe any pattern of co-occurrence of *Dermoloma* spp. with plants suggesting host specialisation. Sympatry is known as a common phenomenon in fungi, but for symbiotic fungi with no host specialisation (which is the probable trophic mode of *Dermoloma*), the drivers of speciation are not well understood (Hernández-Hernández et al. 2021). Ecological assortment, differentiation of resources and reproductive barriers are proposed as the most important factors facilitating sympatric speciation (Thibert-Plante and Hendry 2011; Débarre 2012). In particular, soil heterogeneity may play a key role in promoting fungal diversity and niche differentiation (Mujic et al. 2016). Based on our observations, we hypothesize that: i) *Dermoloma* engages in symbiotic, possibly mutualistic, interactions with plants; ii) because none of the species were observed repeatedly in association with a single plant species, *Dermoloma* species are probably not host specific; iii) multiple *Dermoloma* species were collected in small microhabitats suggesting that microhabitat adaptation not always plays the major role for speciation; iv) a possible explanation of sympatric speciation even at microhabitat scale is functional specialisation for different services provided for the interacting plants (Nagy et al. 2017).

## ﻿﻿Conclusions

The present study is the result of more than 15 years of concentrated research, conducted by an international European and North American mycological team to get consolidated data on 34 documented species accepted in this contribution. Among them, only 11 species are provided with previously published names, 20 are newly described in this study. Three species are fully described without a name assigned because they were represented by only a single collection and were closely related to other species described here; their delimitation will require a better understanding of their genetic and morphological variability. This means that the previous 17 studies publishing nomenclatural and taxonomic novelties (Fig. 1) failed to reach half of genus diversity described in this study. Brief and insufficient descriptions, limited sampling of only a few collections or restricted collecting areas, poor quality of type material and lack of type studies resulted in saturation of problems that hampered the description of new species. Since 2008, no new species have been described from Europe and North America based on morphology alone. More recently, Voto (2022) used the sequence data published by Sánchez-García et al. (2021) to identify a new species based on a unique ITS sequence but failed to define any unique distinguishing morphological characters. The older described species are problematic because of their uncertain concepts and it was necessary to request loans to study them all in order to be able to fully define even the most common of the previously described species. Despite the low number of published species prior to this study, many of these appeared to be synonyms, often misidentified multiple times, and several are not even members of the genus *Dermoloma*. For future studies on agarics, we recommend to: i) focus on morphologically well-defined groups at least at a section level; ii) gather multiple collections per species and preferably from distant locations within the distribution area of the group; iii) collect standardized and statistically supported data about morphology; iv) include the type studies of previously published taxa; and v) avoid reliance on ITS and ribosomal DNA genes alone and to confirm species concepts by multilocus phylogeny and genealogical concordance. Our analysis of stable isotopes showed that species of *Dermoloma* have different nitrogen and carbon isotopic signals from both ectomycorrhizal and saprotrophic fungi, similar to other CHEGD agarics, suggesting how little we know about trophic interactions, ecological roles and evolutionary processes affecting these fungi.

## Supplementary Material

XML Treatment for Dermoloma

XML Treatment for Dermoloma angustisporum

XML Treatment for Dermoloma appalachianum

XML Treatment for Dermoloma applanatum

XML Treatment for Dermoloma atrocinereum

XML Treatment for Dermoloma bellerianum

XML Treatment for Dermoloma aff. bellerianum

XML Treatment for Dermoloma carpathicum

XML Treatment for Dermoloma compactum

XML Treatment for Dermoloma confusum

XML Treatment for Dermoloma cuneifolium

XML Treatment for Dermoloma curvicystidiatum

XML Treatment for Dermoloma fumosidiscum

XML Treatment for Dermoloma fuscobrunneum

XML Treatment for Dermoloma fusipes

XML Treatment for Dermoloma aff. fusipes

XML Treatment for Dermoloma griseobasale

XML Treatment for Dermoloma huartii

XML Treatment for Dermoloma hygrophorus

XML Treatment for Dermoloma intermedium

XML Treatment for Dermoloma josserandii

XML Treatment for Dermoloma magicum

XML Treatment for Dermoloma murinellum

XML Treatment for Dermoloma obscurum

XML Treatment for Dermoloma parvisporum

XML Treatment for Dermoloma phaeopodium

XML Treatment for Dermoloma pruinosipes

XML Treatment for Dermoloma aff. pruinosipes

XML Treatment for Dermoloma pseudojosserandii

XML Treatment for Dermoloma pusillum

XML Treatment for Dermoloma rostratum

XML Treatment for Dermoloma simile

XML Treatment for Dermoloma vellingae

XML Treatment for Dermoloma vestigium

XML Treatment for Neodermoloma

XML Treatment for Neodermoloma campestre

## ﻿﻿Additional information

### Adherence to national and international regulations

All the fungal strains used in this study have been legally obtained, respecting the Convention on Biological Diversity (Rio Convention).

### Author ORCIDs

Katarína Adamčíková https://orcid.org/0000-0001-5407-5233

Munazza Kiran https://orcid.org/0000-0002-3671-9039

Miroslav Caboň https://orcid.org/0000-0002-2255-3816

Brandon P. Matheny https://orcid.org/0000-0003-3857-2189

Marisol Sánchez-García https://orcid.org/0000-0002-0635-6281

Michaela Caboňová https://orcid.org/0000-0002-2207-6183

Gilles Corriol https://orcid.org/0000-0002-2207-6183

Bálint Dima https://orcid.org/0000-0003-2099-3903

Gernot Friebes https://orcid.org/0000-0002-0996-3634

Gareth W. Griffith https://orcid.org/0000-0001-6914-3745

Django Grootmyers https://orcid.org/0000-0002-1042-4902

David Harries https://orcid.org/0000-0002-5477-0938

Alexander Karich https://orcid.org/0009-0005-9295-4163

Armin Mešić https://orcid.org/0000-0002-4479-600X

Martin Mihaljevič https://orcid.org/0000-0002-4875-9345

Pierre-Arthur Moreau https://orcid.org/0000-0003-4783-3643

Ana Pošta https://orcid.org/0000-0003-1715-1609

Vasilii Shapkin https://orcid.org/0000-0002-8394-3009

Zdenko Tkalčec https://orcid.org/0000-0002-1148-9425

Alfredo Vizzini https://orcid.org/0000-0003-2791-3160

Lenka Vondrovicová https://orcid.org/0000-0001-8390-6446

Slavomir Adamčík https://orcid.org/0000-0003-2156-5767

Soňa Jančovičová https://orcid.org/0000-0002-9959-3314

### Data availability

Sequences are deposited in Genbank and listed in the supplementary material, the alignments are submitted in Figshare (https://figshare.com/s/903834519a46fcd6190b).

## Supplementary materials

﻿Supplementary material 1 List of sequences used in phylogenetic analyses Data type xlsx Explanation note List of sequences used in phylogenetic analyses. T! – holotype, lectotype or neotype, E! – epitype. Sequences generated in this study are in bold. https://binary.pensoft.net/file/1370829This dataset is made available under the Open Database License (http://opendatacommons.org/licenses/odbl/1.0/). The Open Database License (ODbL) is a license agreement intended to allow users to freely share, modify, and use this Dataset while maintaining this same freedom for others, provided that the original source and author(s) are credited.Katarína Adamčíková, Munazza Kiran, Miroslav Caboň, Brandon P. Matheny, Marisol Sánchez-García, Eef Arnolds, Michaela Caboňová, Gilles Corriol, Bálint Dima, Gernot Friebes, Gareth W. Griffith, Django Grootmyers, David Harries, Alexander Karich, Armin Mešić, Martin Mihaljevič, Pierre-Arthur Moreau, Ana Pošta, Vasilii Shapkin, Zdenko Tkalčec, Alfredo Vizzini, Lenka Vondrovicová, Slavomir Adamčík, Soňa Jančovičová

﻿Supplementary material 2 *Dermoloma* multilocus alignment Data type fas Explanation note Multilocus alignment of ITS, LSU, rpb1, rpb2, mcm7 and tef1a regions used for phylogenetic analysis of *Dermoloma*-like species. https://binary.pensoft.net/file/1370830This dataset is made available under the Open Database License (http://opendatacommons.org/licenses/odbl/1.0/). The Open Database License (ODbL) is a license agreement intended to allow users to freely share, modify, and use this Dataset while maintaining this same freedom for others, provided that the original source and author(s) are credited.Katarína Adamčíková, Munazza Kiran, Miroslav Caboň, Brandon P. Matheny, Marisol Sánchez-García, Eef Arnolds, Michaela Caboňová, Gilles Corriol, Bálint Dima, Gernot Friebes, Gareth W. Griffith, Django Grootmyers, David Harries, Alexander Karich, Armin Mešić, Martin Mihaljevič, Pierre-Arthur Moreau, Ana Pošta, Vasilii Shapkin, Zdenko Tkalčec, Alfredo Vizzini, Lenka Vondrovicová, Slavomir Adamčík, Soňa Jančovičová

﻿Supplementary material 3 *Tricholomataceae* multilocus alignment Data type fas Explanation note Multilocus alignment of ITS, LSU, rpb1, rpb2 and SSU regions used for phylogenetic analysis of *Tricholomataceae* members. https://binary.pensoft.net/file/1370831This dataset is made available under the Open Database License (http://opendatacommons.org/licenses/odbl/1.0/). The Open Database License (ODbL) is a license agreement intended to allow users to freely share, modify, and use this Dataset while maintaining this same freedom for others, provided that the original source and author(s) are credited.Katarína Adamčíková, Munazza Kiran, Miroslav Caboň, Brandon P. Matheny, Marisol Sánchez-García, Eef Arnolds, Michaela Caboňová, Gilles Corriol, Bálint Dima, Gernot Friebes, Gareth W. Griffith, Django Grootmyers, David Harries, Alexander Karich, Armin Mešić, Martin Mihaljevič, Pierre-Arthur Moreau, Ana Pošta, Vasilii Shapkin, Zdenko Tkalčec, Alfredo Vizzini, Lenka Vondrovicová, Slavomir Adamčík, Soňa Jančovičová

﻿Supplementary material 4 ITS alignment Data type fas Explanation note Sequence alignment of ITS region. https://binary.pensoft.net/file/1370832This dataset is made available under the Open Database License (http://opendatacommons.org/licenses/odbl/1.0/). The Open Database License (ODbL) is a license agreement intended to allow users to freely share, modify, and use this Dataset while maintaining this same freedom for others, provided that the original source and author(s) are credited.Katarína Adamčíková, Munazza Kiran, Miroslav Caboň, Brandon P. Matheny, Marisol Sánchez-García, Eef Arnolds, Michaela Caboňová, Gilles Corriol, Bálint Dima, Gernot Friebes, Gareth W. Griffith, Django Grootmyers, David Harries, Alexander Karich, Armin Mešić, Martin Mihaljevič, Pierre-Arthur Moreau, Ana Pošta, Vasilii Shapkin, Zdenko Tkalčec, Alfredo Vizzini, Lenka Vondrovicová, Slavomir Adamčík, Soňa Jančovičová

﻿Supplementary material 5 Form used for annotating Dermoloma collections in field Data type pdf Explanation note Applicable options were labelled among pre-printed descripting characteristics. https://binary.pensoft.net/file/1370833This dataset is made available under the Open Database License (http://opendatacommons.org/licenses/odbl/1.0/). The Open Database License (ODbL) is a license agreement intended to allow users to freely share, modify, and use this Dataset while maintaining this same freedom for others, provided that the original source and author(s) are credited.Katarína Adamčíková, Munazza Kiran, Miroslav Caboň, Brandon P. Matheny, Marisol Sánchez-García, Eef Arnolds, Michaela Caboňová, Gilles Corriol, Bálint Dima, Gernot Friebes, Gareth W. Griffith, Django Grootmyers, David Harries, Alexander Karich, Armin Mešić, Martin Mihaljevič, Pierre-Arthur Moreau, Ana Pošta, Vasilii Shapkin, Zdenko Tkalčec, Alfredo Vizzini, Lenka Vondrovicová, Slavomir Adamčík, Soňa Jančovičová

﻿Supplementary material 6 Macromorphological characteristics used for species descriptions Data type xlsx Explanation note Macromorphological characteristics used for species descriptions. T! – holotype, lectotype or neotype, E! – epitype. https://binary.pensoft.net/file/1370834This dataset is made available under the Open Database License (http://opendatacommons.org/licenses/odbl/1.0/). The Open Database License (ODbL) is a license agreement intended to allow users to freely share, modify, and use this Dataset while maintaining this same freedom for others, provided that the original source and author(s) are credited.Katarína Adamčíková, Munazza Kiran, Miroslav Caboň, Brandon P. Matheny, Marisol Sánchez-García, Eef Arnolds, Michaela Caboňová, Gilles Corriol, Bálint Dima, Gernot Friebes, Gareth W. Griffith, Django Grootmyers, David Harries, Alexander Karich, Armin Mešić, Martin Mihaljevič, Pierre-Arthur Moreau, Ana Pošta, Vasilii Shapkin, Zdenko Tkalčec, Alfredo Vizzini, Lenka Vondrovicová, Slavomir Adamčík, Soňa Jančovičová

﻿Supplementary material 7 Microscopic observations Data type xlsx Explanation note Average values of microscopic observations (in μm) used for species descriptions. Q – spore length and width ratio, TC – terminal cell in pileipellis, T! – holotype or lectotype, E! – epitype. https://binary.pensoft.net/file/1370835This dataset is made available under the Open Database License (http://opendatacommons.org/licenses/odbl/1.0/). The Open Database License (ODbL) is a license agreement intended to allow users to freely share, modify, and use this Dataset while maintaining this same freedom for others, provided that the original source and author(s) are credited.Katarína Adamčíková, Munazza Kiran, Miroslav Caboň, Brandon P. Matheny, Marisol Sánchez-García, Eef Arnolds, Michaela Caboňová, Gilles Corriol, Bálint Dima, Gernot Friebes, Gareth W. Griffith, Django Grootmyers, David Harries, Alexander Karich, Armin Mešić, Martin Mihaljevič, Pierre-Arthur Moreau, Ana Pošta, Vasilii Shapkin, Zdenko Tkalčec, Alfredo Vizzini, Lenka Vondrovicová, Slavomir Adamčík, Soňa Jančovičová

﻿Supplementary material 8 Phylogenetic tree based on ITS nrDNA Data type pdf Explanation note Phylogram generated by Maximum Likelihood (RAxML) analysis based on ITS region. Maximum likelihood bootstrap support values greater than 50% are indicated above or below the nodes. Sequences originated from type collections are in red and bold. T! – holotype, lectotype or neotype, E! – epitype. https://binary.pensoft.net/file/1370836This dataset is made available under the Open Database License (http://opendatacommons.org/licenses/odbl/1.0/). The Open Database License (ODbL) is a license agreement intended to allow users to freely share, modify, and use this Dataset while maintaining this same freedom for others, provided that the original source and author(s) are credited.Katarína Adamčíková, Munazza Kiran, Miroslav Caboň, Brandon P. Matheny, Marisol Sánchez-García, Eef Arnolds, Michaela Caboňová, Gilles Corriol, Bálint Dima, Gernot Friebes, Gareth W. Griffith, Django Grootmyers, David Harries, Alexander Karich, Armin Mešić, Martin Mihaljevič, Pierre-Arthur Moreau, Ana Pošta, Vasilii Shapkin, Zdenko Tkalčec, Alfredo Vizzini, Lenka Vondrovicová, Slavomir Adamčík, Soňa Jančovičová

﻿Supplementary material 9 Density plots showing statistical differences in micromorphological characters Data type pdf Explanation note Density plots showing statistical differences in micromorphological characters observed on *Dermoloma* (D.) and *Neodermoloma* (N.). Yellow labelled are North American species, green European species with inamyloid spores and purple European species with amyloid spores. a. marginal cells length; b. marginal cells width; c. length of terminal cells in pileipellis near pileus margin; d. width of terminal cells in pileipellis near pileus margin; e. length of terminal cells in pileipellis near pileus centre; f. width of terminal cells in pileipellis near pileus centre; g. caulocystidia length. https://binary.pensoft.net/file/1370837This dataset is made available under the Open Database License (http://opendatacommons.org/licenses/odbl/1.0/). The Open Database License (ODbL) is a license agreement intended to allow users to freely share, modify, and use this Dataset while maintaining this same freedom for others, provided that the original source and author(s) are credited.Katarína Adamčíková, Munazza Kiran, Miroslav Caboň, Brandon P. Matheny, Marisol Sánchez-García, Eef Arnolds, Michaela Caboňová, Gilles Corriol, Bálint Dima, Gernot Friebes, Gareth W. Griffith, Django Grootmyers, David Harries, Alexander Karich, Armin Mešić, Martin Mihaljevič, Pierre-Arthur Moreau, Ana Pošta, Vasilii Shapkin, Zdenko Tkalčec, Alfredo Vizzini, Lenka Vondrovicová, Slavomir Adamčík, Soňa Jančovičová

﻿Supplementary material 10 Data used for analyses of stable isotopes Data type xlsx Explanation note Data used for analyses of stable isotopes δ13C and δ15N with references to published sources. Trophic mode categories: Bryophil – bryophilous fungi, CHEGD Clav – *Clavariaceae*, CHEGD Derm – *Dermoloma*, CHEGD Hygr – *Hygrocybe* s. lato, ECM – ectomycorrhizal fungi, SAP Grass – grassland saprothrophs, SAP – other saprothrophs. All *Dermoloma* measurements were obtained in this study. * – average values of measurents from single site and treatment in Halbwachs et al. (2018). https://binary.pensoft.net/file/1370838This dataset is made available under the Open Database License (http://opendatacommons.org/licenses/odbl/1.0/). The Open Database License (ODbL) is a license agreement intended to allow users to freely share, modify, and use this Dataset while maintaining this same freedom for others, provided that the original source and author(s) are credited.Katarína Adamčíková, Munazza Kiran, Miroslav Caboň, Brandon P. Matheny, Marisol Sánchez-García, Eef Arnolds, Michaela Caboňová, Gilles Corriol, Bálint Dima, Gernot Friebes, Gareth W. Griffith, Django Grootmyers, David Harries, Alexander Karich, Armin Mešić, Martin Mihaljevič, Pierre-Arthur Moreau, Ana Pošta, Vasilii Shapkin, Zdenko Tkalčec, Alfredo Vizzini, Lenka Vondrovicová, Slavomir Adamčík, Soňa Jančovičová

﻿Supplementary material 11 Results of ANOVA Tukey test on stable C and N isotopes Data type xlsx Explanation note Results of ANOVA Tukey test of δ13C (top right) and δ15N (bottom left) ratios among different taxonomic and trophic groups. Significant results differences (p < 0.05) are labelled by an Asterix (*), ns – not significant. Data are presented in Online resource 8. https://binary.pensoft.net/file/1370839This dataset is made available under the Open Database License (http://opendatacommons.org/licenses/odbl/1.0/). The Open Database License (ODbL) is a license agreement intended to allow users to freely share, modify, and use this Dataset while maintaining this same freedom for others, provided that the original source and author(s) are credited.Katarína Adamčíková, Munazza Kiran, Miroslav Caboň, Brandon P. Matheny, Marisol Sánchez-García, Eef Arnolds, Michaela Caboňová, Gilles Corriol, Bálint Dima, Gernot Friebes, Gareth W. Griffith, Django Grootmyers, David Harries, Alexander Karich, Armin Mešić, Martin Mihaljevič, Pierre-Arthur Moreau, Ana Pošta, Vasilii Shapkin, Zdenko Tkalčec, Alfredo Vizzini, Lenka Vondrovicová, Slavomir Adamčík, Soňa Jančovičová
