# Supplementary material for: ﻿A phylogenetic and morphological study of the genus Dermoloma (Agaricales, Tricholomataceae) in Europe and North America exposes inefficiency of opportunistic species descriptions
Source: IMA Fungus. 2025 Jul 10;16:e157337. doi: 10.3897/imafungus.16.157337 (PMC12272084; doi:10.3897/imafungus.16.157337)
Supplement: ﻿Supplementary material 5 — Form used for annotating Dermoloma collections in field [file imafungus-16-e157337-s005.pdf]

A phylogenetic and morphological study of the genus *Dermoloma* in Europe and North America exposes inefficiency of opportunistic species descriptions

Adamčíková K., Kiran M., Caboň M., Matheny P.B., Sánchez-García M., Arnolds E., Caboňová M., Corriol G., Dima B., Friebes G., Griffith G.W., Grootmyers D., Harries D., Karich A., Mešić A., Mihaljevič M., Moreau P.-A., Pošta A., Shapkin V., Tkalčec Z., Vizzini A., Vondrovicová L., Adamčík S.*, Jančovičová S.

*Corresponding author: Slovak Academy of Sciences, Bratislava, Slovakia; e-mail: slavomir.adamcik@savba.sk

Supplementary File 5 Form used for annotating *Dermoloma* collections in field. Applicable options were labelled among pre-printed descripting characteristics

Dermoloma
Country
location

Date Collector
Associated plants

Pileus size
Margin striated translucently at
Shape: convex, umbilicate, umbonate, weakly depressed, crenulate, lobate
Surface rugulose, veined, pitted, rough, smooth, shining, matt, rimulose, hygrophanous, fibrillose, squamulose, granulose, pruinose, cracked

Color near margin	dry
Color near centre	dry

Stipe size
Surface granulose, pruinose, squamulose

Color near gills
Color near base
Shape flexuous, narrowed on base

**Gills** width	Lamellulae
Number	Edge
Color	
**Flesh** Smell	
Consistency	Color
