# Supplementary material for: ﻿A phylogenetic and morphological study of the genus Dermoloma (Agaricales, Tricholomataceae) in Europe and North America exposes inefficiency of opportunistic species descriptions
Source: IMA Fungus. 2025 Jul 10;16:e157337. doi: 10.3897/imafungus.16.157337 (PMC12272084; doi:10.3897/imafungus.16.157337)

A phylogenetic and morphological study of the genus *Dermoloma* in Europe and North America exposes inefficiency of opportunistic species descriptions

IMA Fungus

Adamčíková K., Kiran M., Caboň M., Matheny P.B., Sánchez-García M., Arnolds E., Bálint D., Caboňová M., Corriol G., Friebes G., Griffith G.W., Grootmyers D., Harries D., Karich A., Mesić A., Mihaljevič M., Moreau P.-A., Pošta A., Shapkin V., Tkalčec Z., Vizzini A., Vondrovicová L., Adamčík S.*, Jančovičová S.

*Corresponding author: Slovak Academy of Sciences, Bratislava, Slovakia; e-mail: slavomir.adamcik@savba.sk

Supplementary File 8 Phylogram generated by Maximum Likelihood (RAxML) analysis based on ITS region. Maximum likelihood bootstrap support values greater than 50% are indicated above or below the nodes. Sequences originated from type collections are in red and bold. T! – holotype, lectotype or neotype, E! – epitype

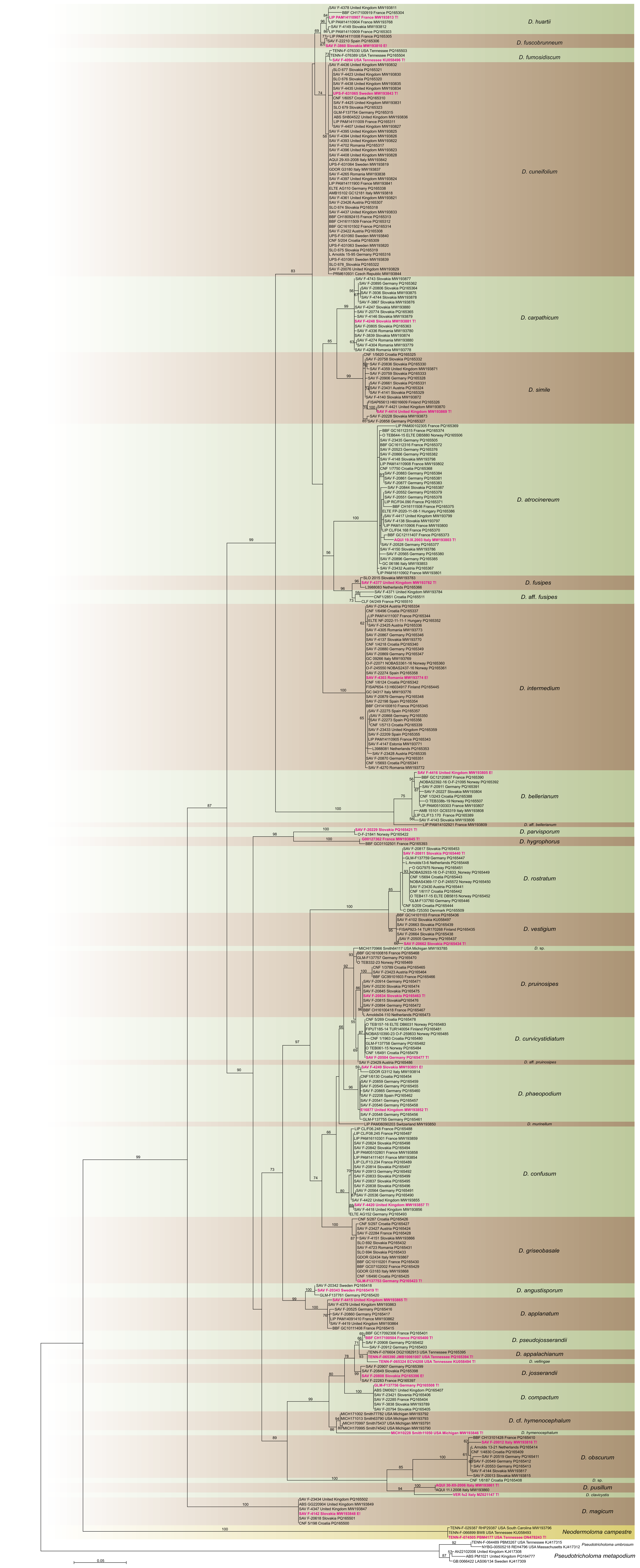

Supplement: ﻿Supplementary material 8 — Phylogenetic tree based on ITS nrDNA [file imafungus-16-e157337-s008.pdf]
