# Supplementary material for: ﻿A phylogenetic and morphological study of the genus Dermoloma (Agaricales, Tricholomataceae) in Europe and North America exposes inefficiency of opportunistic species descriptions
Source: IMA Fungus. 2025 Jul 10;16:e157337. doi: 10.3897/imafungus.16.157337 (PMC12272084; doi:10.3897/imafungus.16.157337)

A phylogenetic and morphological study of the genus *Dermoloma* in Europe and North America exposes inefficiency of opportunistic species descriptions

IMA Fungus

Adamčíková K., Kiran M., Caboň M., Matheny P.B., Sánchez-García M., Arnolds E., Caboňová M., Corriol G., Dima B., Friebes G., Griffith G.W., Grootmyers D., Harries D., Karich A., Mešić A., Mihaljevič M., Moreau P.-A., Pošta A., Shapkin V., Tkalčec Z., Vizzini A., Vondrovicová L., Adamčík S.*, Jančovičová S.

*Corresponding author: Slovak Academy of Sciences, Bratislava, Slovakia; e-mail: slavomir.adamcik@savba.sk

Supplementary file **9** Density plots showing statistical differences in micromorphological characters observed on *Dermoloma* (*D.*) and *Neodermoloma* (*N.*). Yellow labelled are North American species, green European species with inamyloid spores and purple European species with amyloid spores. a. marginal cells length; b. marginal cells width; c. length of terminal cells in pileipellis near pileus margin; d. width of terminal cells in pileipellis near pileus margin; e. length of terminal cells in pileipellis near pileus centre; f. width of terminal cells in pileipellis near pileus centre; g. caulocystidia length. Average values are presented in Supplementary file 7

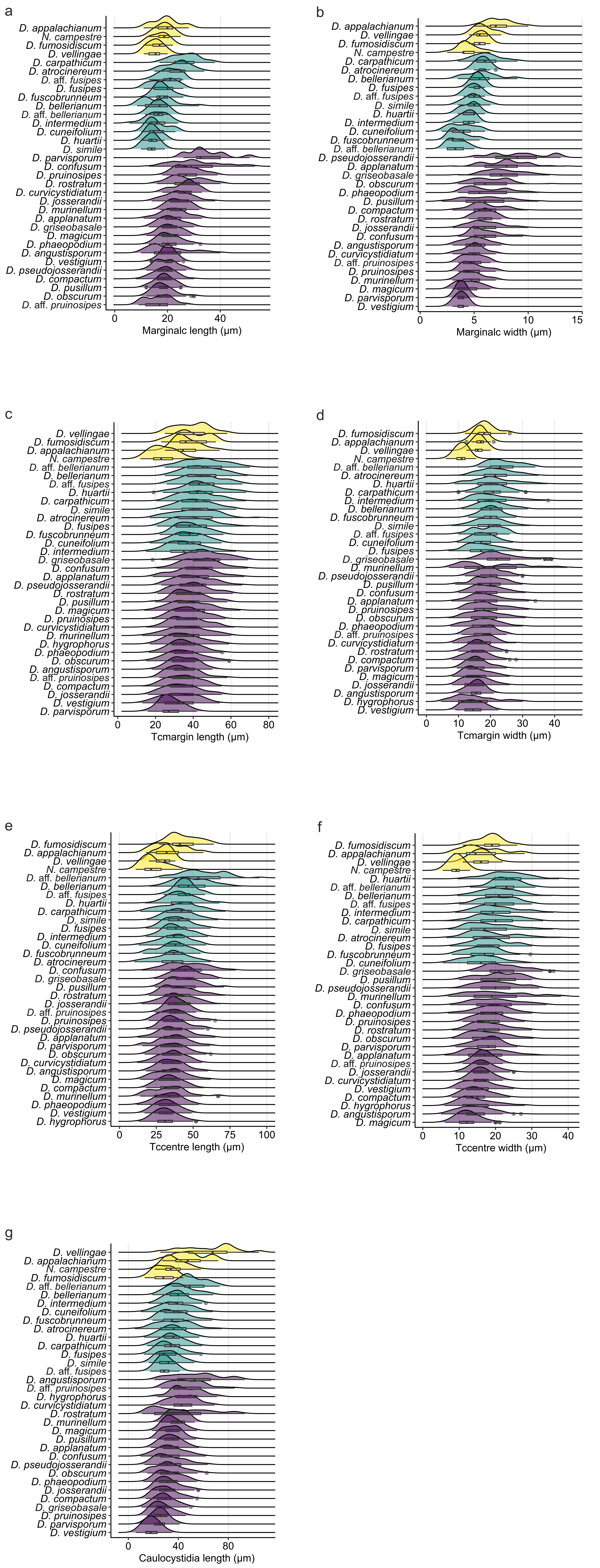

Supplement: ﻿Supplementary material 9 — Density plots showing statistical differences in micromorphological characters [file imafungus-16-e157337-s009.pdf]
